# The Parasporal Body of *Bacillus thuringiensis* subsp. *israelensis*: A Unique Phage Capsid-Associated Prokaryotic Insecticidal Organelle

**DOI:** 10.3390/biology12111421

**Published:** 2023-11-11

**Authors:** Sarah R. Rudd, Leticia Silva Miranda, Hannah R. Curtis, Yves Bigot, Mercedes Diaz-Mendoza, Robert Hice, Victor Nizet, Hyun-Woo Park, Gregor Blaha, Brian A. Federici, Dennis K. Bideshi

**Affiliations:** 1 Program in Biomedical Sciences, Department of Biological Sciences, California Baptist University, Riverside, CA 92504, USA; srudd@students.llu.edu (S.R.R.); leticiasilva.miranda@calbaptist.edu (L.S.M.); hannahcurtis@students.llu.edu (H.R.C.); hpark@calbaptist.edu (H.-W.P.); 2 School of Medicine, Loma Linda University, Loma Linda, CA 92350, USA; 3 Department of Pediatrics, School of Medicine, University of California at San Diego, La Jolla, CA 92093, USA; vnizet@health.ucsd.edu; 4 UMR CNRS7247, Centre INRA Val de Loire, 37380 Nouzilly, France; yves.bigot@inrae.fr; 5 Department of Biochemistry and Molecular Biology, Faculty of Chemical and Biological Sciences, University Complutense of Madrid, 28040 Madrid, Spain; mardia28@ucm.es; 6 Department of Entomology, University of California, Riverside, CA 92521, USA; robert.hice@gmail.com; 7 Department of Biochemistry, University of California, Riverside, CA 92521, USA; gregor.blaha@ucr.edu

**Keywords:** *Bacillus thuringiensis* subsp. *israelensis*, mosquitocidal toxins, bacterial organelle, prokaryote nanocompartments, prokaryotic insecticidal organelle, microcompartments, encapsulin, plasmid-coded phage capsid protein, Bti parasporal body targeting motif, transertion

## Abstract

**Simple Summary:**

*Bacillus thuringiensis* subsp. *israelensis* (Bti) is widely used for controlling disease-carrying mosquitoes. It achieves this by synthesizing four proteins (Cry4Aa1, Cry4Ba1, Cry11Aa1, and Cyt1Aa1), encoded by its pBtoxis plasmid, which target and kill mosquito larvae. These proteins are synthesized during sporulation and stored in three distinct compartments within the parasporal body (PB). As the spore and parasporal body originate from the same cell, they are in close proximity. When larvae ingest them, the four larvicidal proteins dissolve in the midgut lumen and bind to midgut cells, leading to the death of the larva. This creates a nutrient-rich environment for the germinated Bti spores to initiate vegetative growth. The compartments, along with the entire parasporal body, are encased in a net-like multilamellar fibrous matrix (MFM) that contains unique proteins, including Bt152 and Bt075, which are also encoded by pBtoxis. Bt075 shares similarities with encapsulin shell proteins, a class of proteins that encases various cargos. Our findings suggest the larvicidal proteins are packaged into their respective compartments of the parasporal body during translation. Although the parasporal body is larger and more complex than the compartments formed by encapsulins, they both create distinct encased compartments within the bacterial cell, thereby functioning as bacterial organelles.

**Abstract:**

The three most important commercial bacterial insecticides are all derived from subspecies of *Bacillus thuringiensis* (*Bt*). Specifically, *Bt* subsp. *kurstaki* (Btk) and *Bt* subsp. *aizawai* (Bta) are used to control larval lepidopteran pests. The third, *Bt* subsp. *israelensis* (Bti), is primarily used to control mosquito and blackfly larvae. All three subspecies produce a parasporal body (PB) during sporulation. The PB is composed of insecticidal proteins that damage the midgut epithelium, initiating a complex process that results in the death of the insect. Among these three subspecies of *Bt*, Bti is unique as it produces the most complex PB consisting of three compartments. Each compartment is bound by a multilaminar fibrous matrix (MFM). Two compartments contain one protein each, Cry11Aa1 and Cyt1Aa1, while the third contains two, Cry4Aa1/Cry4Ba1. Each compartment is packaged independently before coalescing into the mature spherical PB held together by additional layers of the MFM. This distinctive packaging process is unparalleled among known bacterial organelles, although the underlying molecular biology is yet to be determined. Here, we present structural and molecular evidence that the MFM has a hexagonal pattern to which Bti proteins Bt152 and Bt075 bind. Bt152 binds to a defined spot on the MFM during the development of each compartment, yet its function remains unknown. Bt075 appears to be derived from a bacteriophage major capsid protein (MCP), and though its sequence has markedly diverged, it shares striking 3-D structural similarity to the *Escherichia coli* phage HK97 Head 1 capsid protein. Both proteins are encoded on Bti’s pBtoxis plasmid. Additionally, we have also identified a six-amino acid motif that appears to be part of a novel molecular process responsible for targeting the Cry and Cyt proteins to their cytoplasmic compartments. This paper describes several previously unknown features of the Bti organelle, representing a first step to understanding the biology of a unique process of sorting and packaging of proteins into PBs. The insights from this research suggest a potential for future applications in nanotechnology.

## 1. Introduction

Because prokaryotic organisms lack organelles such as nuclei, mitochondria, and Golgi bodies, they were assumed to have a simple cell architecture. Moreover, they were believed to be devoid of any significant subcellular compartments. This notion has been challenged with the discovery of various sophisticated, subcellular compartments. These compartments are bound by either a membrane, a lipid monolayer, or a proteinaceous coat, and serve specific functions in many bacteria. They are now recognized as bacterial organelles [1].

We propose the insecticidal parasporal body (PB), found in the Gram-positive bacterium *Bacillus thuringiensis* (*Bt*), can be classified as a distinct type of organelle that stores proteins in crystalline form. Many of these proteins are insecticidal in nature and target specific groups of insects. These PBs are produced concurrently with spores toward the end of the vegetative growth phase. The genes responsible for PB formation are located on plasmids rather than on the chromosome. When ingested by insects, the PB dissolves in the alkaline conditions of the midgut, releasing and activating the proteins. These activated proteins target and destroy midgut epithelial cells, ultimately leading to the demise of the insect. The dead insects are colonized by the *Bt* bacteria that have germinated from the spores which were also ingested with the PBs [2,3,4,5,6].

The three most important commercial bacterial insecticides are all derived from subspecies of *Bt. Bt* subsp. *kurstaki* (Btk) and *Bt* subsp. *aizawai* (Bta) are used to control larval lepidopteran pests. In contrast, *Bt* subsp. *israelensis* (Bti) is effective against mosquito and blackfly larvae, important because the adults of many species are vectors of human diseases, such as malaria, filariasis, or viral encephalopathies [2,3,4,5,6,7,8]. The PBs of these subspecies are composed of four major proteins. In both Btk and Bta, all four proteins belong to the Cry family, sharing a similar mass of approximately 135 kDa. They co-crystallize to form a single PB in a bipyramidal form. In Bti, three proteins of the PBs belong to the Cry proteins, while one belongs to the Cyt proteins, specifically Cry4Aa1/Cry4Ba1, Cry11Aa1, and Cyt1Aa1, respectively. However, the mass of these proteins varies, with the three Cry proteins being 72 kDa for Cry11Aa1 and ~130 kDa for Cry4Aa1 and Cry4Ba1, and its one Cyt protein being 28 kDa for Cyt1Aa1 [3].

As sporulation progresses in *Bti*, separate crystals of Cyt1Aa1, Cry11Aa1, and Cry4Aa1/Cry4Ba1 form. Each crystal forms within its own compartment that is bound by a multilaminar fibrous matrix (MFM). These compartments then coalesce to create a spherical PB, held together by additional peripheral layers of MFM, ensuring the insect larva ingests all of the toxins. The genes responsible for Bti’s sophisticated PB are located on a single plasmid, pBtoxis, that encodes a total of 125 proteins [9].

The mechanisms underlying Cry toxicity to insect larvae have been studied extensively [4,10,11,12]. In summary, activated Cry proteins disrupt the membrane integrity by creating pores through interactions with midgut microvillar membrane receptors and binding to plasma membrane adhesion proteins. These receptors include cadherins, aminopeptidase N, alkaline phosphatases, and α-amylase. In contrast, Cyt1Aa1 exhibits a high lipophilicity with a preference for unsaturated fatty acids. It is thought its binding to lipids perturbs and destabilizes plasma membrane integrity [13,14,15].

While Cyt1Aa1 is less toxic than Cry4Aa1/Cry4Ba1 and Cry11Aa1, it synergistically interacts with these three Cry proteins against a wide range of mosquito and blackfly species. This allows Cyt1Aa1 to amplify the larvicidal activities of Bti’s Cry proteins. The synergistic effect of Cyt1Aa1 is not just limited to Cry proteins. Cyt1Aa1 also synergistically interacts with the unrelated Tpp1Aa1/Tpp2Aa1 binary toxin (formerly BinA/BinB) [6] of *Lysinibacillus sphaericus* against many mosquito species. This combined action hinders or delays the development of resistance to *L. sphaericus* in mosquito populations [3,16,17]. Although the precise mechanism remains poorly understood, Cyt1Aa1 appears also to be responsible for the absence of significant resistance to Bti in mosquito and blackfly populations, even after more than forty years of use.

## 2. Studies Supporting Classification of Bti’s PB as a Prokaryotic Insecticidal Organelle

### 2.1. Bti’s PB Is a Complex of Heterogenous Microcompartments Enveloped by a Well-Defined Multilamellar Fibrous Matrix (MFM)

**Figure 1:**
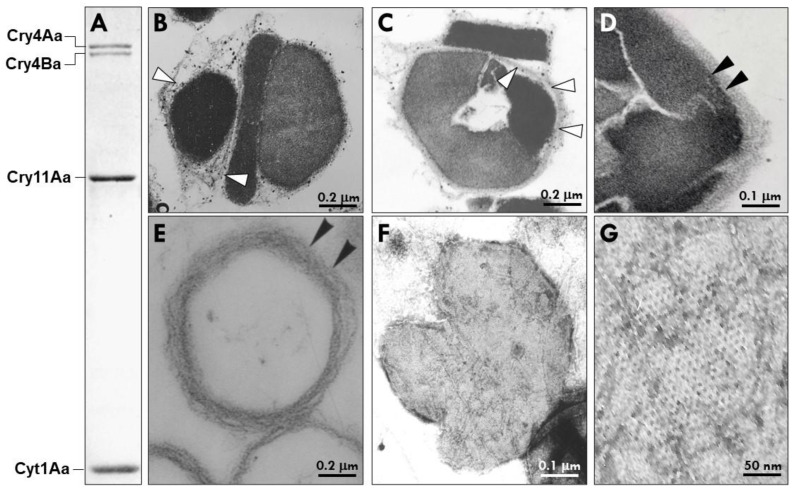
Protein profile and structural features of the parasporal body (PB) of *Bacillus thuringiensis* subsp. *israelensis* (Bti). (**A**) SDS-PAGE showing the major crystal proteins of the PB, i.e., Cry4Aa1 (135 kDa), Cry4Ba1 (128 kDa), Cry11Aa1 (65 kDa), and Cyt1Aa1 (27 kDa). (**B**,**C**) Representative transmission electron micrographs through two Bti parasporal bodies with the arrows pointing to the multilaminar fibrous matrix (MFM) that surrounds each individual crystal protein (white arrowheads indicates MFM inner layer). (**D**) Black arrowheads point to the tightly packed MFM surrounding the Bti PB after lysis of the cell at the end of sporulation. (**E**) Purified MFM after the insecticidal proteins have been dissolved in alkaline buffer. (**F**,**G**) Negatively stained MFM envelope after dissolution of the insecticidal proteins. Note the hexagonal pattern of the MFM.

Previous studies using both light and electron microscopy (EM) revealed the three crystals (Cry4Aa1/Cry4Ba1, Cry11Aa1, and Cyt1Aa1) within the PB are encased in thin layers of MFM [3,18]. Moreover, the mature spherical PB is further wrapped in additional layers of this matrix, as illustrated in Figure 1. Analysis of purified MFM preparations by negatively stained EM revealed a repetitive hexagonal pattern of the MFM. This MFM structure is also found in the PG-14 isolate of *B. thuringiensis* subsp. *morrisoni* (Btm PG-14) that is toxic to both dipteran (mosquito and blackfly) and lepidopteran larvae. Btm PG-14 packages a Cyt protein that differs from Cyt1Aa1 by one amino acid residue at position 82 (i.e., alanine rather than proline) and three other mosquitocidal proteins of similar size to Cry4Aa1, Cry4Ba1, and Cry11Aa1, as determined by SDS-PAGE analysis [19,20]. It is noteworthy that Btm PG-14 also contains a bipyramidal crystal that is also packaged in the MFM, which was later shown to have lepidopteran larvicidal activity.

### 2.2. Bti’s MFM Contains a Unique Collection of Proteins

While the integrity of the PB is crucial for its high larvicidal activity, our understanding of the MFM that holds the PB together remains limited. To address this, we previously performed mass spectrometric analysis of MFM-enriched samples [18]. These samples were prepared from purified PBs by dissolving the crystalline Cry and Cyt proteins under alkaline conditions. The analysis identified peptides originating from Cry4Aa1/Cry4Ba1, Cry11Aa1, and Cyt1Aa1, along with peptides from five novel proteins, i.e., Bt152, Bt148, Bt113, Bt075, and Bt073. The genes coding for these proteins are all localized on the pBtoxis plasmid [9], which also harbors the genes for the Cry and Cyt proteins. It is assumed most, if not all, of the proteins involved with the complex structures of the PB are encoded on pBtoxis. However, we cannot rule out the possibility that some genes involved in the formation of the PB are located on the chromosome of Bti.

### 2.3. Bt073, Bt113 and Bt148

At present, no predictive functions are known for Bt073 and Bt113 [9,18], whereas our comparative sequence analyses suggest Bt148 (95 residues, 10 kDa) is a putative transcription factor that contains well-conserved overlapping domains found in SpoVT_AbrB (COG2002, smart00966) and MazE antitoxin (pfam04014) of *Bacillus subtilis*. Collectively, these AbrB-like proteins belong to a family of small regulatory proteins with diverse functions. Most notable among these functions are the maintenance of low-copy number plasmids, reduction of protein synthesis, gene regulation, and retardation of cell growth under nutritional stress, spore formation, and cell survival. Further studies are required to determine if similar functions are associated with Bt148, as pBtoxis is a low-copy number plasmid [9] and Cry and Cyt crystal synthesis and PB maturation is sporulation-dependent.

### 2.4. Bt152 Tracks the Fusion of PB Compartments into a Single Parasporal Body

**Figure 2:**
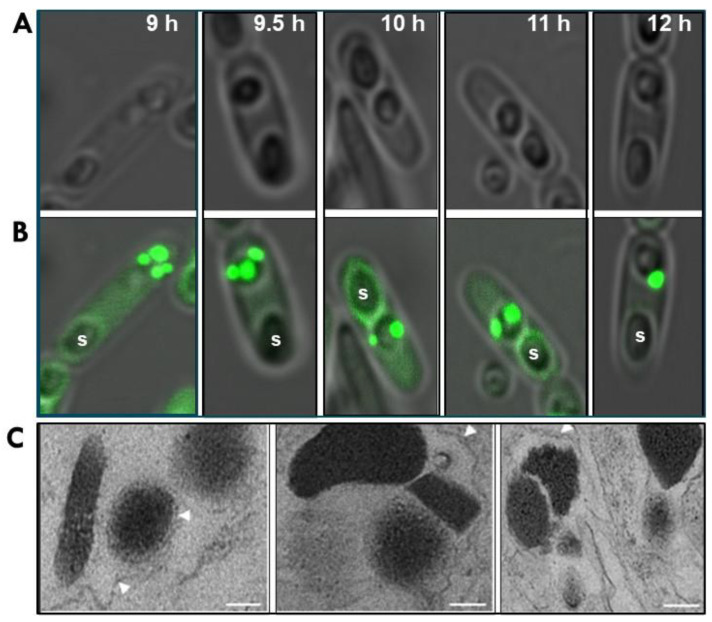
Microscopy showing progressive formation of Bti’s mature parasporal body (PB). Bti 4Q5 was transformed with a plasmid (pHT152-GFP) harboring a recombinant construct coding for the Bt152:GFP fusion protein, previously shown by Diaz-Mendoza et al. [18] to specifically bind to the multilaminar fibrous matrix. Confocal phase contrast (**A**) and fluorescence microscopy (**B**) of sporulating cells at 9–12 h. (**C**) Transmission electron micrographs of mutant 4Q5 strains deficient in Bt152 gene function showing crystalline inclusions did not aggregate to form intact parasporal bodies. Spores (S) and unorganized lamellar matrices (white arrowheads) are shown; bar = 0.2 μm.

We have demonstrated that Bt152 localizes specifically to the PB and plays a crucial role in holding together the intact PB [18]. Bt152 is a 54-kDa protein consisting of two domains: a metallophosphatase (MPP, cd00838) domain and a ricin-like beta-trefoil fold (pfam00652) domain. The protein localizes to a defined area of the MFM surrounding each of the developing PB. It does not extend to other cytoplasmic and structural components of the sporulating cell or the endospore (Figure 2).

The importance of Bt152 for the structural integrity of the PB was underscored by a mutant that lacked the protein [18]. Transmission EM of the PB of this mutant revealed the PB was unstable in the absence of Bt152. Unlike the tightly bound structure of the wild type PB, the compartments of the mutant’s PB were not firmly connected. The fibrillar layers of the MFM surrounding all compartments were only loosely connected (Figure 2).

Our recent work, using confocal microscopy indicates as early as 7 h after germination, Bt152-GFP localizes to at least three small but distinct cytoplasmic inclusions (Figure 2). These inclusions continue to grow, ultimately coalescing to a mature PB by the 12 h mark. Although more detailed studies are required, our findings suggest each crystal is enveloped by a thin MFM prior to assembly of the mature PB. This unique packaging process sets itself apart from all other known prokaryotic mechanisms for organelle formation.

### 2.5. Bt075 Is Structurally Similar to Encapsulin Shell Proteins

**Figure 3:**
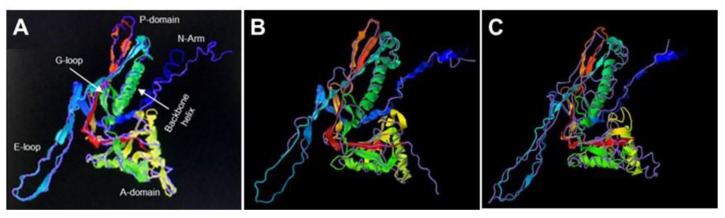
3-D modeling of plasmid-coded Bt075. In silico analysis performed with I-TASSER. (https://zhanggroup.org/I-TASSER/, accessed on 3 July 2022) showed Bt075 was structurally similar to *Escherichia coli* bacteriophage HK97 K169Y Head 1 capsid protein (pink overlay) (**A**), a feature shared by all known plasmid-coded shell proteins (Enc) of encapsulin organelles in bacteria and archaea, including the holo-Srpl encapsulin complex of *Synechococcus elongatus* PCC 7942 (PDB DOI: 10.2210/pdb6X8M/pdb, accessed on 4 August 2022; pink overlay) (**B**), and the closed pentamer of the *Haliangium ochraceum* encapsulin (PDB DOI: 10.2210/pdb7OE2/pdb, accessed on 4 August 2022; pink overlay) (**C**).

Initially, Bt075 (286 residues, 31 kDa) attracted our attention for further study, as it possesses no readily identifiable conserved sequence motifs or domains, based on BLAST searches. However, Bt075 shares ~32% identity and ~48% similarity with a phage major capsid protein found in *Planctomycetes* species (MCE9583169) and ~24% identity and ~40% similarity with head capsid proteins of bacteriophage ZY21 (UIS24576) and Xp15 (YP_239278) of *Pseudomonas* and *Xanthomonas*, respectively (Supplemental Materials). Moreover, though the amino acid sequences have diverged markedly among these proteins, molecular modeling revealed Bt075 may be structurally similar to the capsid shell proteins of encapsulins (Figure 3). All known encapsulin shell proteins share significant structural homology with the *Escherichia coli* bacteriophage HK97 Head 1 capsid protein, even though their amino acid sequences have diverged significantly. Encapsulins are polyhedral nanocompartment organelles ubiquitous among bacteria and archaea. They encase cargo inside a protein shell, with the major structural component being the encapsulin shell protein [21,22,23,24,25]. Interestingly, though structurally similar to phage capsid proteins, all known encapsulin shell proteins are encoded by plasmids. They self-assemble into icosahedral compartments, typically ranging from 25–42 nm in diameter.

Based on sequence analyses, four families (Families 1–4) of encapsulins have been identified and the cargos they encapsulate are known in at least 11 archaea and eubacteria species. The cargos include ferritin-like proteins (Flp), DyP-type peroxidases (DyP), iron-mineralizing encapsulin-associated Firmicute (IMEF), and FolB that is involved in biosynthesis of folate (vitamin B9), each encoded by plasmid-borne encapsulin operons [26,27,28]. Although specific functions of encapsulins in prokaryote biology are still being determined, knockout mutants of Flp-associated encapsulin in *Mycobacterium xanthus* have shown increased susceptibility to H_2_O_2_-induced oxidative stress. Given DyP-type peroxidases function in redox processes and possess antioxidant properties, it is conceivable they also play a role in oxidative stress responses [29]. More recently, a similar function for an encapsulin in *M. tuberculosis* has been demonstrated [30].

**Figure 4:**
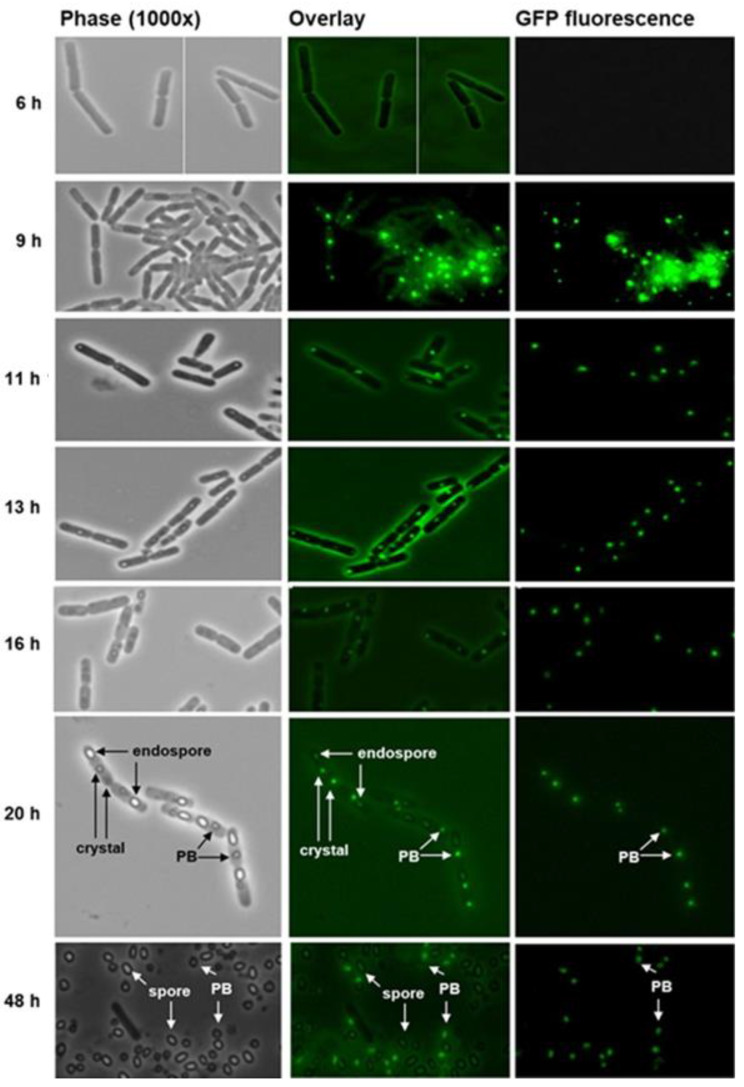
Bt075 specifically locates to the parasporal body (PB) of *Bacillus thuringiensis* subsp. *israelensis* strain 4Q5. Spores of Bti were cultured on nutrient agar at 30 °C and samples were collected at various intervals for microscopic examination. Bt075-GFP appeared at discrete cytoplasmic locations as early as 9 h before the appearance of crystals. Inclusions and well-formed crystals were detected as early as 11 h and 20 h, respectively. Endospores and PBs were clearly identifiable at 20 h. Greater than 99% of cells autolyzed by 48 h releasing spores and PBs. Note at 20–48 h, Bt075-GFP associates exclusively with the mature PB, similar to what was observed with Bt152 by Diaz-Mendoza et al. [18], based on the lack of fluorescence in other cellular structures, including the cell wall, endospore, and free spore.

Bt075 emerges at approximately 9 h after spore germination and appears as aggregates within the cytoplasm (Figure 4). These aggregates potentially associate with components of the MFM long before the visible formation of Cry4Aa1/Cry4Ba1, Cry11Aa1, and Cyt1Aa1 crystals. This suggests Bt075′s function is related to precursor events necessary for the synthesis of crystalline inclusions and the subsequent assembly of the PB, and perhaps is essential for maintaining the structural integrity of the composite PB. We have not been successful in isolating Bti 4Q5 mutants lacking Bt075, preventing us from definitively determining its requirement for PB stability.

Unlike encapsulin cargos, the insecticidal proteins that accumulate in several Bt subspecies in crystalline form but are not enveloped by a peripheral matrix are presumably not toxic to the bacterial cell in which they develop. The many different crystalline inclusions produced by *Bt* vary in composition. For example, the HD73 strain of *B. thuringiensis* subsp. *kurstaki* produces a bipyramidal inclusion composed of a single protein of molecular weight of ~135 kDa. Others, like the HD1 strain of the same subspecies, contains two distinct inclusions, one bipyramidal, formed by the co-crystalization of three 135 kDa proteins (Cry1Aa, Cry1Ab, Cry1Ac), and a second quasicuboidal inclusion containing only a 65 kDa protein (Cry2Aa). Similar crystalline inclusions occur in certain strains of *B. thuringiensis* subsp. *aizawai*, where four 135-kDa proteins (Cry1Aa, Cry1Ab, Cry1Ac, Cry1Da) co-crystallize to form a single bipyramidal inclusion; however, the crystals produced in these strains are not delimited by peripheral matrices [3].

With regards to Bti, and by extension strain PG14, it remains unclear whether the complex packaging of its larvicidal proteins prevents or reduces potential detrimental effects associated with the synthesis of the insecticidal proteins. Crystals of Cry4Aa1/Cry4Ba1 or Cry11Aa1 have been independently synthesized in an acrystalliferous strain of *Bt* and other bacteria without the need for accessory proteins. However, the synthesis of the lipophilic Cyt1Aa1 requires a 20-kDa protein, also encoded by pBtoxis, which prevents it from binding to lipids in the plasma membrane, averting host cell death [15,31,32,33]. Therefore, at least for Cyt1Aa1, its accumulation in the confines of the Bt152/Bt075-associated MFM confers an advantage to Bti, which otherwise would be toxic to the bacterium.

### 2.6. Signature Sequence for the Bti’s Insecticidal Proteins

The complex process involved in the formation of the Bti PB raises several intriguing questions. How do the individual compartments arise? Do the Cry and Cyt protein crystals start forming within the precursor MFM compartments? If so, how are the proteins directed to these compartments? Are there specific amino acid sequences that target the Cyt protein and each Cry protein to a specific MFM compartment? If such targeting sequences exist, additional proteins must be involved in this process, so that each protein type, e.g., Cyt1Aa1 versus Cry11Aa1, would be sorted to its specific MFM compartment. In fact, it is likely two or more unidentified proteins encoded by pBtoxis play a role in governing this remarkable sorting of proteins.

**Figure 5:**
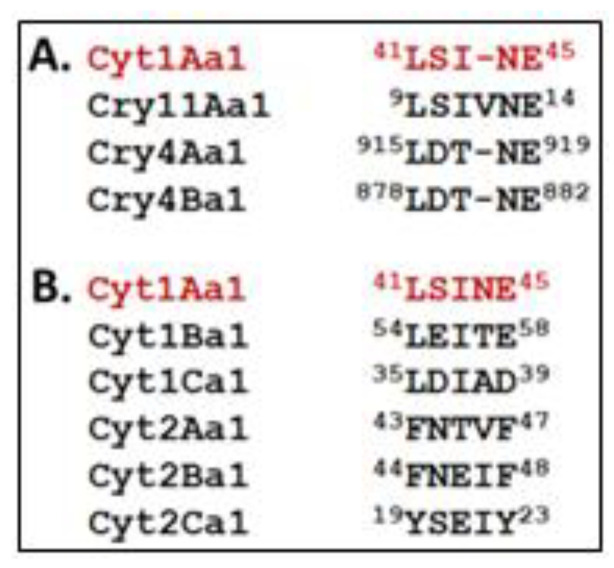
Putative signature motif “L(S/D)(I/T)NE” potentially involved in trafficking Cyt and Cry to the multilaminar fibrous matrix (MFM). (**A**) The motif is derived from Bti’s Cyt1Aa1 (red type) and is found in Cry11Aa1, Cry4Aa1, Cry4Ba1, each of which is directed to Bti’s MFM. (**B**) Cyt1B,C and Cyt2A,B,C proteins are more diverged at respective residues in the putative signature motif, particularly at the 4th and 5th (“NE”) positions; these protein are not trafficked to a fibrous matrix.

As a first step in unravelling this intriguing sorting process, we performed a comparative sequence analysis and identified a motif within Cry and Cyt proteins that may be involved in at least one component of the sorting process. A motif common to these proteins, is “L(S/D)(I/T)NE” (Figure 5). This motif is present in Bti larvicidal proteins (Cyt1Aa1, Cry11Aa1, Cry4Aa1, and Cry4Ba1), but not in closely related Cyt proteins, such as Cyt1Ba1, Cyt1Ca1, Cyt2Aa1, Cyt2Ba1, or Cyt2Ca1 [6,13], or in the overwhelming majority of other Cry proteins, especially those not encased in MFM layers within Bt. 

This motif is found in different regions of each Bti protein. In Cyt1Aa1 and Cry11Aa1, it is found in the N-terminal half, and in the C-terminal half in Cry4Aa1 and Cry4Ba1. These differences may reflect different stages of Bti PB evolution, as well as unknown complexities of the unique translation process. For example, the motif position may reflect the 3-D structure of the Bti protein bound to one or more other proteins that traffic its synthesis to each specific MFM compartment.

**Figure 6:**
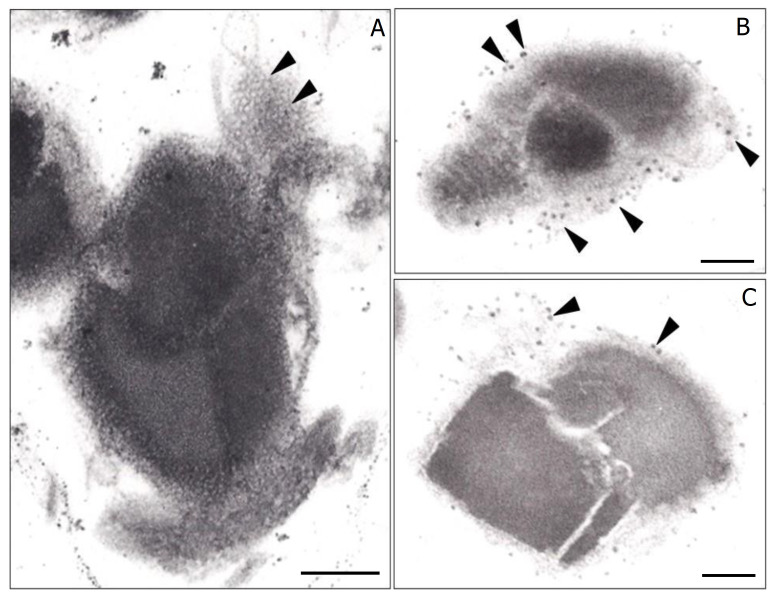
Transmission electron micrographs through purified parasporal bodies of *Bacillus thuringiensis* subsp. *israelensis.* (**A**) Grazing electron micrograph section through the multilaminar fibrous matrix (arrowheads). Note the hexagonal pattern of the MFM. (**B**,**C**) Electron micrographs showing what apparently are ribosomes (arrows) bound to the MFM. Bar, 0.2 μm.

Another unknown aspect of Bti’s PB development is how each of its different insecticidal proteins are sorted into their respective MFM compartment. One potential mechanism is the “transertion” process, in which transcription, translation, and insertion are tightly linked to RNA polymerase, ribosomes, and the translocon of the lipid membrane compartment [1,34]. In this process, multiple ribosomes attach to a single mRNA molecule encoding a polypeptide which associates with the membrane of the compartment. As the polypeptide is synthesized, it is simultaneously inserted through the membrane and undergoes folding as it enters the membrane-bound compartment. Applying this concept to PB formation in Bti, it is conceivable multiple ribosomes bind to each mRNA molecule coding for an insecticidal protein. These ribosomes would then facilitate the insertion of the developing polypeptide through the hexagonal structures (Figure 1G) that compose the MFM. This coordinated process, with high concentrations of the same protein within each specific insecticidal protein compartment, would likely facilitate their crystallization. Evidence suggesting such a process may occur during PB formation in Bti is found in electron micrographs of purified parasporal bodies (Figure 6). These micrographs reveal clusters of structures ~20 nm in size, consistent with the size of prokaryote ribosomes [35,36], bound to the surface of the MFM.

Answers to these and other questions will help provide critical insights into the biogenesis of Bti’s PB, including whether ribosomes specifically associate with the MFM, whether Cry and Cyt proteins are inserted into their respective compartments through molecular channels in the MFM, and the functional roles of Bt073, Bt113, and Bt148 within the MFM.

## 3. Is Bti’s PB a Novel Prokaryotic Organelle to Store Proteins?

Like other recognized organelles, Bti’s PB possesses well-defined subcellular boundaries and packages a cargo, in this case, insecticidal proteins. However, the intricate structure of Bti’s PB does not align neatly with any of the current classifications of known prokaryotic organelles [1]. For instance, prokaryotic organelles are categorized into several types based on their structural characteristics. These include membrane-bound, protein-lipid monolayer-bound, and protein-bound organelles. Examples of membrane-bound organelles include thylakoids and chromatophores, while protein-lipid monolayer-bound organelles encompass chlorosomes and lipid bodies. Protein-bound organelles are polyhedral microcompartments and nanocompartments. Bioinformatic surveys of their shell proteins and protein cargos have identified at least 68 types and subtypes of these microcompartments and include metabolosomes and carboxysomes [1,20,21,22,23]. Both membrane-bound and protein-lipid monolayer-bound organelles also include storage organelles, like the membrane-bound ferrosomes, magnetosomes, and nitrate vacuoles, or the monolayer-bound lipid bodies [1].

In addition, Bti’s composite PB is more than 20 times larger than encapsulin-based nanocompartments (~1000 nm vs. ~50 nm) and at least 2 times larger than non-encapsulin-based microcompartment organelles (<500 nm) [1,30]. Thus, we propose Bti’s PB represents a unique subcellular organelle delimited by a complex MFM that anchors important structural proteins, such as Bt075. Bt075 appears to be distributed throughout the MFM and may assist in reinforcing this matrix, thereby helping distribute the individual insecticidal proteins to their separate compartments within the PB. Specifically, during sporulation, this unique bacterial organelle selectively packages three mosquitocidal Cry proteins, along with the cytolytic Cyt1Aa1 protein, using a complex MFM. This results in the formation of a potent spherical inclusion that is readily ingested by Bti’s hosts, primarily the larvae of certain flies, notably mosquitoes. Following ingestion of PBs and spores, vegetative Bti cells invade the intoxicated and dead larvae, where they reproduce and form new endospores and PBs.

## 4. Conclusions

While studies on the structural biology, mechanisms of toxicity, synthetic manipulations of *cry* and *cyt* genes, and environmental impact are undoubtedly critical for the continued commercial success and practical applications of Bti, the novel characteristics of the MFM merit further investigation. The identification and characterization of newly discovered proteins such as Bt152 and Bt075 supports our concept that the Bti PB represents a unique prokaryotic insect larvicidal organelle, distinct from those found in other bacteria and archaea. In the foreseeable future, Bti will continue to be the most efficacious and widely used natural bacterial mosquito larvicide. Inquiry into the structural biology related to the biogenesis of Bti’s composite PB, and addressing the questions raised in this study, could prove valuable. These inquiries are likely to uncover novel mechanisms within basic prokaryotic cellular biology, including extending knowledge related to the sorting of proteins within bacterial cells. In addition, the potential to engineer other protein toxins or molecules for targeted delivery into the MFM lumen and PB could be of value to the biotechnology industry, including applications in drug delivery, as has been proposed for encapsulins and microcompartment organelles [37,38,39,40,41,42,43,44]. We hope these observations stimulate further interest and research into the structural organization and biogenesis of Bti’s PB.

## 5. Materials and Methods

### 5.1. Bacterial Strains and Propagation

*B. thuringiensis* subsp. *israelensis* (Bti) 4Q5 has been previously described [18]. Bti was routinely cultured and maintained on Nutrient Agar (NA) and Nutrient Broth (NB) (Difco). The pBtoxis from Bti was purified using the Nucleobond Plasmid Midi Kit (Clontech) and recombinant plasmids were propagated in *Escherichia coli* DH5a and purified using the Wizard Plus Miniprep kit (Promega), according to the manufacturers’ protocols.

### 5.2. In Vivo Localization of Bt075

To construct the Bt075-green fluorescent protein (GFP) chimera, the Bt075 gene sequence containing its native promoter and open reading frame (ORF) was amplified by PCR using the Expand Long Template PCR system (Boehringer GmnHm Mannheim, Germany) with pBtoxis as the template and the primer pair, DB075F 5′-CCCTCTAGACCGTTCACGAATGAGGTTTCTTTCAA-3′

DB075R 5′-GGGGGCGCCTATTGCTACATTTTTCACACTAGCAA-3′ with added Xba1 and Nar1 sites (underlined), respectively. The reaction was performed for 30 cycles as follows: 94 °C for 3 min, 55 °C for 30 s, 72 °C for 2 min, with a final 3 min extension step. The 1.54 kbp amplicon was digested with Xba1 and Nar1 and the 0.97 kbp Nar1 -> Sph1 fragment containing the *gfp* ORF in pHT-152-GFP (Diaz-Mendoza et al. 2012) were cloned in the Xba1 -> Sph1 site in pHT3101. The resulting plasmid, pHT-Bt075 was used to transform Bti 4Q5 by electroporation as described by Diaz et al. [18]. Transformants were selected on NA with erythromycin (25 mg/mL). Six transformants were initially screened for GFP expression, and as each showed localization of Bt075-GFP specifically to the parasporal body, one was chosen for further study. This recombinant (Bti/pHT-Bt075-GFP) was grown in 10 mL of NB with erythromycin (25 mg/mL) at 30 °C and 250 RPM for 72 h until > 99% of cells had sporulated and lysed. After, 0.5 mL of the culture was transferred to a 1.5 mL tube and incubated at 65 °C to destroy viable vegetative cells and 10 mL of the sample was spread on NA with erythromycin (25 mg/mL). Triplicate cultures were incubated at 30 °C and cells samples were acquired from the agar plates at various intervals for examination by phase and fluorescence microscopy at 1000× magnification (Zeiss Axio PhotoFluor LM-75-89 North). Images were collected with the SPOT Basic 5.6 digital system (Spot Imaging).

### 5.3. Sequence Analyses, Molecular Modeling and Phylogenetic Trees

The amino acid sequence (286 residues, 31 kDa) of Bt075 was analyzed using various online programs including BLAST (https://blast.ncbi.nlm.nih.gov/Blast.cgi, accessed on 3 July 2022). The theoretical 3-D model of Bt075 was obtained using the online I-TASSER program (https://zhanggroup.org/I-TASSER/, accessed on 4 August 2022) [45,46,47]. The 500 sequences of Bt075 orthologs (Supplemental Figures S1 and S2) were extracted from a psi-blast search results (https://blast.ncbi.nlm.nih.gov/Blast.cgi?PROGRAM=blastp&PAGE_TYPE=BlastSearch&LINK_LOC=blasthome, accessed on 3 July 2022) using three search rounds and default parameters and a minimal e-value of 0.001. The phylogenetic tree was constructed using facilities at https://ngphylogeny.fr/workflows/alacarte, accessed on 3 July 2022. The sequence alignment was made using the MAFFT program. Curation for informative positions in the sequence alignment was made using the BMGE program and the phylogenetic tree inference was calculated from the curated alignment using PhyML+SMS programs.

### 5.4. Confocal Microscopy

Spores of Bti 4Q5 transformed with the pHT-Bt152-GFP was grown on Nutrient Agar with erythromycin (25 mg/mL) at 30 °C, and samples were taken periodically (9 to 12 h) for imaging with the Leica SP5 system fitted with an argon laser that was activated to 20% (Microscopy and Imaging Core Facility, University of California, Riverside, Riverside, CA, USA, 92521). The images were taken using a 40× objective and a 2.5× digital zoom, following excitation at 488 nm and emission collection between 500 and 550 nm. The bright-field images were captured by simultaneous scanning.

### 5.5. Electron Microscopy

Sucrose gradient centrifugation was used to isolate and purify parasporal bodies, as described previously [18]. Briefly, Bti 4Q7 was grown in in 500 mL of Nutrient Broth + glucose (NBG) for 4 days until >99% of cells sporulated and lysed, as determined by phase-contrast microscopy. The spore-crystal mixture was harvested by centrifugation at 18,000 rpm (~39,800× *g*) for 30 min at 4 °C in a Beckman Sorvall SS-34 rotor. The pellet was washed five times with double-distilled water (ddH_2_O), resuspended in 10 mL of ddH_2_O, sonicated for 5 min (Ultrasonic Homogenizer 4710 series, Cole Palmer), and layered onto a discontinuous sucrose gradient (67–79%) for centrifugation at 20,000 rpm for 1 h at 4 °C using a Beckman SW-27 rotor. Bands containing parasporal bodies were extracted and taken through two additional rounds of gradient centrifugation to optimize purity. The isolated parasporal bodies were washed five times in ddH_2_O and collected each time by centrifugation using a Sorvall SS-34 rotor at 18,000 rpm for 30 min at 4 °C. Purified parasporal bodies from recombinant strains and enriched multilamellar fibrous matrix were examined by electron microscopy. Pellets were fixed in 3% glutaraldehyde in 0.1 M cacodylate buffer for 2 h, subsequently fixed in 1% OsO4 in the same buffer for 1 h, dehydrated in an ethanol series to propylene oxide, and embedded in Epon-Araldite. Ultrathin sections, prepared using a Sorvall model MT5 microtome equipped with a diamond knife, were stained with lead citrate and uranyl acetate and examined and photographed with a Hitachi 600 transmission electron microscope (TEM). Negative staining was also performed using samples treated bodies with 1% (wt/vol) phosphotungstic acid (K-PTS) in 0.1 M sodium phosphate (pH 5 to 7) adjusted with 1 N KOH.

## Data Availability

Data are contained within the article and supplementary materials.

## Supplementary Materials

The following supporting information can be downloaded at: https://www.mdpi.com/article/10.3390/biology12111421/s1, Figure S1: FASTA alignment file of 500 first Bt075 orthologs obtained with psi-BLAST at https://blast.ncbi.nlm.nih.gov/Blast.cgi?PROGRAM=blastp&PAGE_TYPE=BlastSearch&LINK_LOC=blasthome (accessed on 3 July 2022) and three search round using default parameters. The sequence alignment was made with MAFFT at https://ngphylogeny.fr/workflows/alacarte, accessed on 3 July 2022; Figure S2. Phylogeny of Bt075 orthologs. True phage capsid proteins are shown in blue. The two putative clades of exapted phage capsid proteins are shown in green. Inside the large clade to which Bt075 belongs (shown in purple), Bt075 protein relative in the genera *Planctomycetes*, *Nitrosomonas*, *Bacillus* and *Paenibacillus* species. and other bacterial species are respectively in grey, hypothetical proteins in red, sepia, brown, green and pink. Hypothetical proteins interspersed in the tree among capsid proteins are in red.
