# Supplementary material for: The Parasporal Body of Bacillus thuringiensis subsp. israelensis: A Unique Phage Capsid-Associated Prokaryotic Insecticidal Organelle
Source: Biology (Basel). 2023 Nov 11;12(11):1421. doi: 10.3390/biology12111421 (PMC10669011; doi:10.3390/biology12111421)
Supplement: Supplementary file 1 [file biology-2673757-supplementary_Figure S2_RUDD et al 2023.pdf › biology-2673757-supplementary_Figure S2_RUDD et al 2023.pdf]

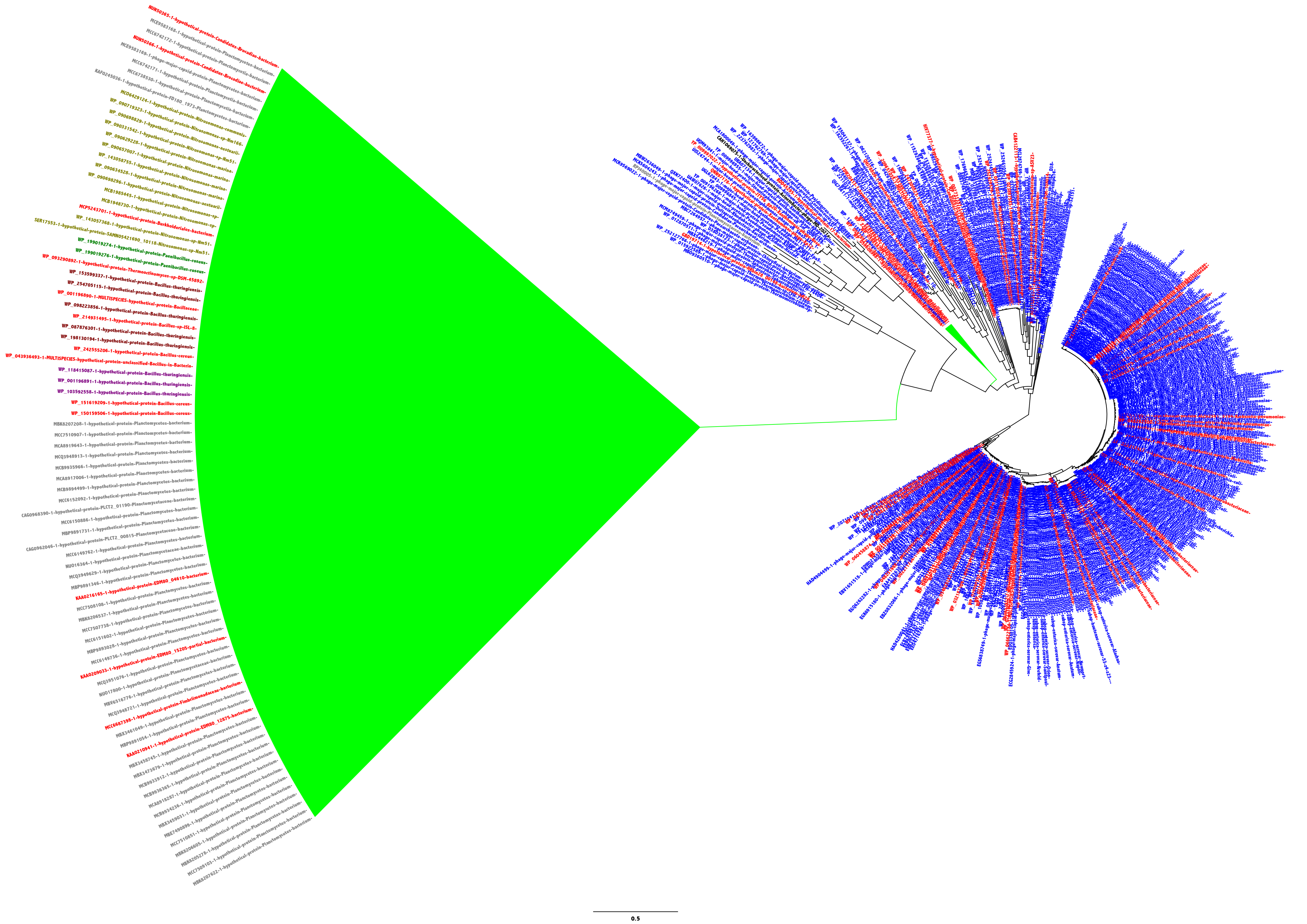

0.5
