# Supplementary material for: The Parasporal Body of Bacillus thuringiensis subsp. israelensis: A Unique Phage Capsid-Associated Prokaryotic Insecticidal Organelle
Source: Biology (Basel). 2023 Nov 11;12(11):1421. doi: 10.3390/biology12111421 (PMC10669011; doi:10.3390/biology12111421)
Supplement: Supplementary file 1 [file biology-12-01421-s001.zip › biology-2673757-supplementary_Figure S1_RUDD et al 2023.pdf]

1. WP_103592558.1 hypothetical protein [Bacillus thuringiensis]
2. WP_001196891.1 hypothetical protein [Bacillus thuringiensis]
3. WP_043936493.1 MULTISPECIES: hypothetical protein [unclassified Bacillus (in: Bacteria)]
4. WP_214931495.1 hypothetical protein [Bacillus sp. ISL-8]
5. WP_098223856.1 hypothetical protein [Bacillus thuringiensis]
6. WP_198130194.1 hypothetical protein [Bacillus thuringiensis]
7. WP_150159506.1 hypothetical protein [Bacillus cereus]
8. WP_001196890.1 MULTISPECIES: hypothetical protein [Bacillaceae]
9. WP_151619209.1 hypothetical protein [Bacillus cereus]
10. WP_087876301.1 hypothetical protein [Bacillus thuringiensis]
11. WP_242555206.1 hypothetical protein [Bacillus cereus]
12. WP_118415087.1 hypothetical protein [Bacillus thuringiensis]
13. WP_153599337.1 hypothetical protein [Bacillus thuringiensis]
14. WP_093290892.1 hypothetical protein [Thermoactinomyces sp. DSM 45892]
15. WP_090719323.1 hypothetical protein [Nitrosomonas sp. Nm166]
16. WP_090657007.1 hypothetical protein [Nitrosomonas marina]
17. WP_090629228.1 hypothetical protein [Nitrosomonas marina]
18. WP_090698829.1 hypothetical protein [Nitrosomonas aestuarii]
19. MCB1985445.1 hypothetical protein [Nitrosomonas sp.]
20. MCO6429124.1 hypothetical protein [Nitrosomonas communis]
21. SER17553.1 hypothetical protein SAMN05421690_10118 [Nitrosomonas sp. Nm51]
22. WP_143058755.1 hypothetical protein [Nitrosomonas marina]
23. WP_143057368.1 hypothetical protein [Nitrosomonas sp. Nm51]
24. MCP5243701.1 hypothetical protein [Burkholderiales bacterium]
25. WP_090698296.1 hypothetical protein [Nitrosomonas aestuarii]
26. WP_090634528.1 hypothetical protein [Nitrosomonas marina]
27. WP_090331542.1 hypothetical protein [Nitrosomonas sp. Nm51]
28. MCB1948730.1 hypothetical protein [Nitrosomonas sp.]
29. MBV6516776.1 hypothetical protein [Planctomycetes bacterium]
30. NUO17800.1 hypothetical protein [Planctomycetaceae bacterium]
31. MCC6151602.1 hypothetical protein [Planctomycetes bacterium]
32. MCC6149736.1 hypothetical protein [Planctomycetes bacterium]
33. MBP9893028.1 hypothetical protein [Planctomycetes bacterium]
34. MCA8919643.1 hypothetical protein [Planctomycetes bacterium]
35. MCQ3948913.1 hypothetical protein [Planctomycetes bacterium]
36. WP_254705115.1 hypothetical protein [Bacillus thuringiensis]
37. MCC7510907.1 hypothetical protein [Planctomycetes bacterium]
38. KAA0216165.1 hypothetical protein EDM80_04610 [bacterium]
39. WP_199019276.1 hypothetical protein [Paenibacillus roseus]
40. MBK8207208.1 hypothetical protein [Planctomycetes bacterium]
41. WP_199019274.1 hypothetical protein [Paenibacillus roseus]
42. MCC7507738.1 hypothetical protein [Planctomycetes bacterium]
43. MCC7508108.1 hypothetical protein [Planctomycetes bacterium]
44. MBK8206537.1 hypothetical protein [Planctomycetes bacterium]
45. MBP9891346.1 hypothetical protein [Planctomycetes bacterium]
46. MBK8206605.1 hypothetical protein [Planctomycetes bacterium]
47. MBE7490899.1 hypothetical protein [Planctomycetes bacterium]
48. MBK8207622.1 hypothetical protein [Planctomycetes bacterium]
49. MBK8205278.1 hypothetical protein [Planctomycetes bacterium]
50. MCC7509103.1 hypothetical protein [Planctomycetes bacterium]
51. MCB9934236.1 hypothetical protein [Planctomycetes bacterium]
52. KAA0210941.1 hypothetical protein EDM80_12875 [bacterium]
53. MCC6742171.1 hypothetical protein [Planctomycetia bacterium]
54. MCB9894499.1 hypothetical protein [Planctomycetes bacterium]
55. MCC6742172.1 hypothetical protein [Planctomycetia bacterium]
56. MCQ3951076.1 hypothetical protein [Planctomycetes bacterium]
57. MCC6687398.1 hypothetical protein [Fimbriimonadaceae bacterium]
58. MCB9935966.1 hypothetical protein [Planctomycetes bacterium]
59. NUN50366.1 hypothetical protein [Candidatus Brocadiae bacterium]
60. MBX3458745.1 hypothetical protein [Planctomycetes bacterium]
61. MCE9583168.1 hypothetical protein [Planctomycetes bacterium]
62. MCC7510851.1 hypothetical protein [Planctomycetes bacterium]
63. MBP9891054.1 hypothetical protein [Planctomycetes bacterium]
64. MCE9583169.1 phage major capsid protein [Planctomycetes bacterium]
65. NUN50365.1 hypothetical protein [Candidatus Brocadiae bacterium]
66. MCA8918287.1 hypothetical protein [Planctomycetes bacterium]
67. MBX3461049.1 hypothetical protein [Planctomycetes bacterium]
68. KAF0245036.1 hypothetical protein FD180_1973 [Planctomycetes bacterium]
69. MCC6738530.1 hypothetical protein [Planctomycetia bacterium]
70. MCC6149762.1 hypothetical protein [Planctomycetes bacterium]
71. MCQ3949629.1 hypothetical protein [Planctomycetes bacterium]
72. NUO16364.1 hypothetical protein [Planctomycetaceae bacterium]
73. CAG0962046.1 hypothetical protein PLCT2_00815 [Planctomycetaceae bacterium]
74. WP_002731357.1 hypothetical protein [Magnetospirillum molischianum]
75. MBX3459031.1 hypothetical protein [Planctomycetes bacterium]
76. MCQ3948721.1 hypothetical protein [Planctomycetes bacterium]
77. MCB9933912.1 hypothetical protein [Planctomycetes bacterium]
78. MBO6507501.1 phage major capsid protein [Roseibium sp.]

79. KAA0209033.1 hypothetical protein EDM80_15205, partial [bacterium]
80. WP_238616883.1 phage major capsid protein [Ectothiorhodospira shaposhnikovii]
81. NQZ53225.1 phage major capsid protein [Piscirickettsiaceae bacterium]
82. WP_199153549.1 hypothetical protein [Chromobacterium sp. ASV23]
83. TDW20413.1 hypothetical protein EV128_12543 [Rhizobium azibense]
84. WP_221103340.1 phage major capsid protein [Rhizobium bangladeshense]
85. WP_221122355.1 phage major capsid protein [Rhizobium lentis]
86. QIG69072.1 major capsid protein [Rhizobium phage RHph_N1_10]
87. MBP9891731.1 hypothetical protein [Planctomycetes bacterium]
88. MCA8917006.1 hypothetical protein [Planctomycetes bacterium]
89. QIG75611.1 major capsid protein [Rhizobium phage RHph_Y2_11]
90. WP_092498438.1 phage major capsid protein [Chelativorans sp. A52C2]
91. MCB9936365.1 hypothetical protein [Planctomycetes bacterium]
92. CAG0968390.1 hypothetical protein PLCT2_01190 [Planctomycetaceae bacterium]
93. MCC6150886.1 hypothetical protein [Planctomycetes bacterium]
94. WP_045991564.1 phage capsid protein [Halomonas sp. S2151]
95. WP_065784019.1 MULTISPECIES: phage capsid protein [unclassified Ensifer]
96. WP_144196640.1 phage major capsid protein [Cupriavidus campinensis]
97. WP_253399773.1 hypothetical protein [Methylorubrum extorquens]
98. YP_009620683.1 major capsid protein [Pseudomonas phage nickie]
99. AMB48270.1 phage capsid protein [Methylobacterium sp. AMS5]
100. UKL14918.1 major capsid protein [Pseudomonas phage hairong]
101. WP_026610715.1 hypothetical protein [Methylocaldum szegediense]
102. MCC6152092.1 hypothetical protein [Planctomycetes bacterium]
103. UIS24576.1 major capsid protein [Pseudomonas phage ZY21]
104. WP_247125312.1 phage major capsid protein [Acinetobacter baumannii]
105. WP_031986190.1 phage capsid protein [Acinetobacter baumannii]
106. MBP4651980.1 phage major capsid protein [Acinetobacter baumannii]
107. MCL2874769.1 phage major capsid protein [Betaproteobacteria bacterium]
108. WP_176966547.1 phage major capsid protein, partial [Acinetobacter baumannii]
109. HAV4743116.1 phage major capsid protein [Acinetobacter baumannii]
110. WP_001130341.1 MULTISPECIES: hypothetical protein [Acinetobacter]
111. WP_224446258.1 hypothetical protein [Cupriavidus metallidurans]
112. WP_252429529.1 hypothetical protein, partial [Acinetobacter baumannii]
113. MBQ0662298.1 phage major capsid protein [Acinetobacter baumannii]
114. EIU0976857.1 phage major capsid protein [Acinetobacter baumannii]
115. WP_115292648.1 phage major capsid protein [Pseudomonas mendocina]
116. DAP19794.1 TPA: MAG TPA: major capsid protein [Siphoviridae sp.]
117. WP_161289910.1 phage major capsid protein [Acinetobacter baumannii]
118. WP_001130337.1 hypothetical protein [Acinetobacter baumannii]
119. DAM63278.1 TPA: MAG TPA: major capsid protein [Siphoviridae sp.]
120. WP_063664455.1 phage major capsid protein [Comamonas thiooxydans]
121. WP_252436504.1 phage major capsid protein, partial [Acinetobacter baumannii]
122. WP_104915983.1 phage major capsid protein [Acinetobacter baumannii]
123. WP_151708812.1 phage major capsid protein [Acinetobacter brisouii]
124. WP_031980863.1 phage capsid protein [Acinetobacter baumannii]
125. WP_023897583.1 hypothetical protein [Acinetobacter baumannii]
126. HAV4899581.1 phage major capsid protein [Acinetobacter baumannii]
127. WP_042670060.1 phage capsid protein [Methylobacterium sp. B34]
128. WP_209651035.1 MULTISPECIES: hypothetical protein [Methylobacterium]
129. WP_124618513.1 phage major capsid protein [Burkholderia contaminans]
130. WP_059608968.1 hypothetical protein [Burkholderia anthina]
131. WP_059789217.1 hypothetical protein [Burkholderia sp. MSMB1072]
132. EGY2841718.1 phage major capsid protein [Acinetobacter baumannii]
133. MCK9569022.1 phage major capsid protein [Candidatus Pacearchaeota archaeon]
134. WP_059858205.1 hypothetical protein [Burkholderia cepacia]
135. WP_252414503.1 phage major capsid protein, partial [Acinetobacter baumannii]
136. DAS20049.1 TPA: MAG TPA: major capsid protein [Siphoviridae sp.]
137. WP_093294920.1 phage major capsid protein [Sphingomonas sp. NFR04]
138. WP_102064968.1 phage major capsid protein [Ralstonia pickettii]
139. WP_247300523.1 hypothetical protein [Bradyrhizobium sp. 179]
140. WP_047884360.1 hypothetical protein [Photobacterium ganghwense]
141. WP_187057131.1 phage major capsid protein [Dyella telluris]
142. WP_155041137.1 phage major capsid protein [Paracoccus litorisediminis]
143. WP_207104130.1 phage major capsid protein [Photobacterium ganghwense]
144. RWB95541.1 phage major capsid protein [Mesorhizobium sp.]
145. WP_150063515.1 phage major capsid protein [Roseospira marina]
146. HIV77377.1 hypothetical protein [Candidatus Sphingomonas excrementigallinarum]
147. WP_097566451.1 phage major capsid protein [Cronobacter sakazakii]
148. WP_166500133.1 phage major capsid protein [Cronobacter sakazakii]
149. WP_216296239.1 phage major capsid protein [Cronobacter sakazakii]
150. WP_080953189.1 phage capsid protein [Cronobacter sakazakii]
151. HAV7489798.1 phage major capsid protein [Escherichia coli]
152. WP_161580717.1 phage major capsid protein [Cronobacter muytjensii]
153. RWI35418.1 phage major capsid protein [Mesorhizobium sp.]
154. WP_130097775.1 MULTISPECIES: phage major capsid protein [Citrobacter]
155. EFG0346231.1 phage major capsid protein [Escherichia coli]
156. WP_029590302.1 MULTISPECIES: hypothetical protein [Cronobacter]
157. WP_085044976.1 phage capsid protein [Cronobacter sakazakii]
158. WP_052904762.1 MULTISPECIES: hypothetical protein [Enterobacteriaceae]

159. WP_014962239.1 MULTISPECIES: hypothetical protein [Enterobacteriaceae]
160. WP_052904335.1 hypothetical protein [Escherichia coli]
161. WP_059296125.1 phage capsid protein, partial [Acinetobacter baumannii]
162. MBE0438045.1 phage major capsid protein [Methylomicrobium sp.]
163. WP_052983289.1 hypothetical protein [Shigella sonnei]
164. WP_105875125.1 phage major capsid protein [Cronobacter malonaticus]
165. WP_080817813.1 phage capsid protein [Cronobacter sakazakii]
166. WP_029463964.1 MULTISPECIES: hypothetical protein [Cronobacter]
167. WP_209732192.1 phage major capsid protein [Cronobacter sakazakii]
168. WP_032969640.1 hypothetical protein [Cronobacter malonaticus]
169. WP_080781210.1 phage capsid protein [Cronobacter sakazakii]
170. WP_096095769.1 phage major capsid protein [Escherichia coli]
171. WP_076734789.1 phage capsid protein [Cronobacter sakazakii]
172. EFF6507864.1 phage major capsid protein [Escherichia coli]
173. HAL3311757.1 phage major capsid protein [Escherichia coli]
174. UTU32364.1 phage major capsid protein [Escherichia coli]
175. MCK2723972.1 phage major capsid protein [Escherichia coli]
176. EGS6838749.1 phage major capsid protein [Salmonella enterica subsp. enterica serovar Agona]
177. WP_244079899.1 phage major capsid protein [Escherichia coli]
178. EHC2975283.1 phage major capsid protein [Shigella sonnei]
179. WP_069127011.1 phage capsid protein [Klebsiella pneumoniae]
180. EDZ1273658.1 phage major capsid protein [Salmonella enterica]
181. WP_193125375.1 phage major capsid protein [Escherichia coli]
182. EJF8070929.1 phage major capsid protein [Escherichia coli]
183. WP_161620577.1 phage major capsid protein [Escherichia coli]
184. EFJ2838014.1 phage major capsid protein [Escherichia coli]
185. EJK5757188.1 phage major capsid protein [Escherichia coli]
186. EIH9371074.1 phage major capsid protein [Escherichia coli]
187. WP_001130339.1 MULTISPECIES: hypothetical protein [Enterobacteriaceae]
188. MHX32748.1 phage major capsid protein [Escherichia coli]
189. WP_072644904.1 phage capsid protein [Escherichia coli]
190. MBC7264957.1 phage major capsid protein [Chloroflexi bacterium]
191. HBL6848639.1 phage major capsid protein [Escherichia coli]
192. WP_062120516.1 phage major capsid protein [Aureimonas sp. AU40]
193. HAH5045117.1 phage major capsid protein [Escherichia coli]
194. WP_243540855.1 phage major capsid protein [Escherichia coli]
195. WP_112901748.1 phage major capsid protein [Escherichia coli]
196. EIT7775743.1 phage major capsid protein [Escherichia coli]
197. EFV7979351.1 phage major capsid protein [Shigella sonnei]
198. WP_042063131.1 hypothetical protein [Escherichia coli]
199. EEZ8393064.1 phage major capsid protein [Escherichia coli]
200. EEY7369300.1 phage major capsid protein [Escherichia coli]
201. EFG0309418.1 phage major capsid protein [Escherichia coli]
202. WP_182552267.1 phage major capsid protein [Phyllobacterium myrsinacearum]
203. WP_063091425.1 hypothetical protein [Escherichia coli]
204. CAB4122113.1 hypothetical protein UFOVP26_79 [uncultured Caudovirales phage]
205. WP_112043712.1 phage major capsid protein [Escherichia coli]
206. HBB7811611.1 phage major capsid protein [Escherichia coli]
207. WP_001130338.1 MULTISPECIES: hypothetical protein [Enterobacteriaceae]
208. HAJ7535281.1 phage major capsid protein [Escherichia coli]
209. WP_096165287.1 phage major capsid protein [Escherichia coli]
210. WP_085457360.1 phage capsid protein [Escherichia coli]
211. WP_032328869.1 hypothetical protein [Escherichia coli]
212. WP_053904528.1 MULTISPECIES: hypothetical protein [Escherichia]
213. MBB0544530.1 phage major capsid protein [Escherichia coli]
214. EFK7891705.1 phage major capsid protein [Escherichia coli]
215. EAB8604575.1 phage major capsid protein [Shigella sonnei]
216. WP_149556591.1 phage major capsid protein [Escherichia coli]
217. WP_149540218.1 phage major capsid protein [Escherichia albertii]
218. EFH9124402.1 phage major capsid protein [Escherichia coli]
219. WP_052988632.1 MULTISPECIES: hypothetical protein [Enterobacteriaceae]
220. WP_247150342.1 phage major capsid protein [Escherichia coli]
221. WP_094259633.1 phage major capsid protein [Escherichia coli]
222. WP_061158051.1 hypothetical protein [Escherichia coli]
223. WP_033803025.1 hypothetical protein [Escherichia coli]
224. MBZ8365671.1 phage major capsid protein [Escherichia coli]
225. WP_203054096.1 phage major capsid protein [Escherichia coli]
226. EFC1505940.1 phage major capsid protein [Escherichia coli]
227. WP_134894003.1 phage major capsid protein [Escherichia coli]
228. WP_251569595.1 hypothetical protein [Parasutterella sp. DSM 108034]
229. HBU8289600.1 phage major capsid protein [Escherichia coli]
230. WP_160334835.1 phage major capsid protein [Parasutterella sp. NM82_D38]
231. HBH5230159.1 phage major capsid protein [Escherichia coli]
232. WP_106889102.1 phage major capsid protein [Escherichia coli]
233. HAM5980914.1 phage major capsid protein [Escherichia coli]
234. WP_161623763.1 phage major capsid protein [Escherichia coli]
235. EFF1057382.1 phage major capsid protein [Escherichia coli]
236. EFZ7197477.1 phage major capsid protein [Shigella sonnei]
237. WP_231351989.1 phage major capsid protein [Escherichia coli]
238. WP_193119742.1 phage major capsid protein [Escherichia coli]

239. WP_252127766.1 phage major capsid protein [Thermomicrobium sp. CFH 73360]
240. EFW0034188.1 phage major capsid protein [Shigella sonnei]
241. EJE3861318.1 phage major capsid protein [Escherichia coli]
242. WP_085458157.1 MULTISPECIES: phage capsid protein [Escherichia]
243. WP_094337983.1 MULTISPECIES: phage major capsid protein [Escherichia]
244. WP_053886737.1 MULTISPECIES: hypothetical protein [Enterobacteriaceae]
245. MCN1174709.1 phage major capsid protein [Escherichia coli]
246. WP_152935148.1 phage major capsid protein [Escherichia coli]
247. EFX2756071.1 phage major capsid protein [Shigella sonnei]
248. EFC0466466.1 phage major capsid protein [Escherichia coli]
249. MCN4038342.1 phage major capsid protein [Escherichia coli]
250. EFD9355743.1 phage major capsid protein [Escherichia coli]
251. WP_097497792.1 phage major capsid protein [Escherichia coli]
252. EFA9209962.1 phage major capsid protein [Escherichia coli]
253. HBB1414416.1 phage major capsid protein [Escherichia coli]
254. HAO7436534.1 phage major capsid protein [Escherichia coli]
255. HAW3037422.1 phage major capsid protein [Escherichia coli]
256. HAW1565627.1 phage major capsid protein [Escherichia coli]
257. WP_032187309.1 MULTISPECIES: hypothetical protein [Enterobacteriaceae]
258. EEQ2490861.1 phage major capsid protein [Escherichia coli]
259. WP_053900371.1 hypothetical protein [Escherichia coli]
260. WP_109949435.1 phage major capsid protein [Escherichia coli]
261. HCC6220448.1 phage major capsid protein [Klebsiella pneumoniae]
262. HAN9820645.1 phage major capsid protein [Escherichia coli]
263. WP_046622184.1 MULTISPECIES: hypothetical protein [Enterobacter cloacae complex]
264. WP_039264878.1 MULTISPECIES: hypothetical protein [Enterobacteriaceae]
265. WP_113613520.1 phage major capsid protein [Klebsiella pneumoniae]
266. EEV7694734.1 phage major capsid protein [Escherichia coli]
267. WP_072124166.1 phage capsid protein [Klebsiella variicola]
268. WP_048292500.1 hypothetical protein [Klebsiella pneumoniae]
269. WP_247188155.1 phage major capsid protein [Escherichia coli]
270. DAE04111.1 TPA: MAG TPA: major capsid protein [Siphoviridae sp. ctmpG14]
271. WP_001405040.1 hypothetical protein [Escherichia coli]
272. WP_065304476.1 phage capsid protein [Escherichia coli]
273. HBZ3184842.1 phage major capsid protein [Klebsiella pneumoniae]
274. WP_119127007.1 phage major capsid protein [Klebsiella pneumoniae]
275. EIM7866883.1 phage major capsid protein [Escherichia coli]
276. WP_001130340.1 MULTISPECIES: hypothetical protein [Enterobacteriaceae]
277. WP_202979481.1 phage major capsid protein [Escherichia coli]
278. HAT3636238.1 phage major capsid protein [Raoultella ornithinolytica]
279. EFI9420290.1 phage major capsid protein [Escherichia coli]
280. WP_126646421.1 phage major capsid protein [Escherichia coli]
281. WP_252461054.1 phage major capsid protein, partial [Acinetobacter baumannii]
282. WP_221016063.1 phage major capsid protein [Klebsiella pneumoniae]
283. EFK0326126.1 phage major capsid protein [Escherichia coli]
284. HAJ7861025.1 phage major capsid protein [Escherichia coli]
285. EHH5028748.1 phage major capsid protein [Escherichia coli]
286. HAU5664456.1 phage major capsid protein [Citrobacter freundii]
287. EAB6775759.1 phage major capsid protein [Escherichia coli]
288. EBZ0832066.1 phage major capsid protein [Salmonella enterica subsp. enterica serovar Java]
289. WP_064343494.1 MULTISPECIES: hypothetical protein [Klebsiella]
290. WP_087507360.1 phage capsid protein [Escherichia coli]
291. WP_236525880.1 phage major capsid protein [Escherichia coli]
292. WP_096178288.1 phage major capsid protein [Escherichia coli]
293. WP_104074192.1 phage major capsid protein [Escherichia coli]
294. HAL2790855.1 phage major capsid protein [Escherichia coli]
295. HBR7729850.1 phage major capsid protein [Klebsiella pneumoniae]
296. WP_221012288.1 phage major capsid protein [Klebsiella pneumoniae]
297. HBV4481339.1 phage major capsid protein [Klebsiella pneumoniae]
298. EFF2501934.1 phage major capsid protein [Escherichia coli]
299. EIW9109236.1 phage major capsid protein [Klebsiella pneumoniae]
300. HAO3297438.1 phage major capsid protein [Escherichia coli]
301. WP_064165097.1 hypothetical protein [Klebsiella pneumoniae]
302. QJJ61945.1 phage major capsid protein [Klebsiella pneumoniae]
303. WP_089075232.1 phage major capsid protein [Escherichia coli]
304. WP_097307300.1 phage major capsid protein [Escherichia coli]
305. HBR4262047.1 phage major capsid protein [Klebsiella pneumoniae]
306. WP_004109857.1 MULTISPECIES: hypothetical protein [Enterobacteriaceae]
307. EEO4716749.1 phage major capsid protein [Salmonella enterica subsp. enterica serovar Tennessee]
308. HBT8335443.1 phage major capsid protein [Klebsiella pneumoniae]
309. EHK0743263.1 phage major capsid protein [Salmonella enterica]
310. WP_006812514.1 MULTISPECIES: hypothetical protein [Enterobacteriaceae]
311. WP_054625182.1 MULTISPECIES: hypothetical protein [Enterobacteriaceae]
312. WP_032217427.1 hypothetical protein [Escherichia coli]
313. EET8203310.1 phage major capsid protein [Escherichia coli]
314. WP_045339225.1 MULTISPECIES: hypothetical protein [Enterobacteriaceae]
315. EIH0829516.1 phage major capsid protein [Escherichia coli]
316. CAF2811462.1 hypothetical protein AI2925V1_5194 [Klebsiella pneumoniae]
317. EJG5508613.1 phage major capsid protein [Salmonella enterica]
318. WP_194797343.1 phage major capsid protein [Klebsiella pneumoniae]

319. WP_078207497.1 phage capsid protein [Escherichia coli]
320. WP_016607444.1 hypothetical protein [Yersinia pestis]
321. WP_204363305.1 phage major capsid protein [Klebsiella pneumoniae]
322. EBK5293162.1 phage major capsid protein [Salmonella enterica]
323. WP_063929200.1 hypothetical protein [Enterobacter hormaechei]
324. EEZ8608344.1 phage major capsid protein [Escherichia coli]
325. WP_096842503.1 phage major capsid protein [Escherichia coli]
326. HBX2495344.1 phage major capsid protein [Klebsiella pneumoniae]
327. WP_065519942.1 phage capsid protein [Klebsiella pneumoniae]
328. WP_086910796.1 MULTISPECIES: phage capsid protein [Klebsiella]
329. WP_040110313.1 MULTISPECIES: hypothetical protein [Enterobacteriaceae]
330. WP_194304202.1 phage major capsid protein [Enterobacter hormaechei]
331. WP_116431201.1 phage major capsid protein [Klebsiella pneumoniae]
332. WP_001130336.1 MULTISPECIES: hypothetical protein [Enterobacteriaceae]
333. WP_250209263.1 phage major capsid protein [Enterobacter cloacae]
334. WP_023316010.1 MULTISPECIES: hypothetical protein [Enterobacteriaceae]
335. ECB7446470.1 phage major capsid protein [Salmonella enterica subsp. enterica serovar Braenderup]
336. HAM5174158.1 phage major capsid protein [Escherichia coli]
337. EHR3333785.1 phage major capsid protein [Salmonella enterica subsp. enterica serovar Senftenberg]
338. HBV3788408.1 phage major capsid protein [Klebsiella pneumoniae]
339. EBS8359733.1 phage major capsid protein [Salmonella enterica]
340. WP_159115294.1 phage major capsid protein [Klebsiella pneumoniae]
341. WP_060458874.1 MULTISPECIES: hypothetical protein [Enterobacter cloacae complex]
342. EET8285689.1 phage major capsid protein [Escherichia coli]
343. WP_002211784.1 MULTISPECIES: hypothetical protein [Gammaproteobacteria]
344. WP_072105584.1 phage capsid protein [Enterobacter cloacae]
345. WP_150006605.1 phage major capsid protein, partial [Klebsiella pneumoniae]
346. WP_175617232.1 phage major capsid protein [Escherichia coli]
347. WP_192856648.1 phage major capsid protein, partial [Escherichia coli]
348. EHJ4216072.1 phage major capsid protein [Salmonella enterica]
349. WP_227899545.1 phage major capsid protein [Escherichia coli]
350. HCD2326535.1 phage major capsid protein [Enterobacter hormaechei]
351. WP_086374674.1 phage capsid protein, partial [Acinetobacter baumannii]
352. EDQ0380811.1 phage major capsid protein [Salmonella enterica subsp. enterica serovar Oslo]
353. BDD45295.1 hypothetical protein 17 [bacterium]
354. HBT7858040.1 phage major capsid protein [Klebsiella pneumoniae]
355. WP_060453932.1 MULTISPECIES: hypothetical protein [Enterobacteriaceae]
356. EAA4173638.1 phage major capsid protein [Salmonella enterica subsp. enterica]
357. HBQ8554378.1 phage major capsid protein [Klebsiella pneumoniae]
358. WP_247156820.1 phage major capsid protein [Escherichia coli]
359. HBV0452233.1 phage major capsid protein [Escherichia coli]
360. EDW0215574.1 phage major capsid protein [Salmonella enterica subsp. enterica]
361. WP_046083837.1 hypothetical protein [Escherichia fergusonii]
362. EIW8542850.1 phage major capsid protein [Klebsiella pneumoniae]
363. HAK0268282.1 phage major capsid protein [Salmonella enterica]
364. EGK6915180.1 phage major capsid protein [Salmonella enterica subsp. enterica serovar Enteritidis]
365. WP_071692149.1 phage capsid protein [Citrobacter portucalensis]
366. EBN2282108.1 phage major capsid protein [Salmonella enterica]
367. EDQ3388105.1 phage major capsid protein [Salmonella enterica subsp. houtenae]
368. ECT3984105.1 phage major capsid protein [Salmonella enterica subsp. houtenae serovar 53:z4,z23:-]
369. EBV1651518.1 phage major capsid protein [Salmonella enterica subsp. enterica serovar Bareilly]
370. WP_042863544.1 hypothetical protein [Enterobacter hormaechei]
371. WP_048231968.1 MULTISPECIES: hypothetical protein [Citrobacter freundii complex]
372. EEP8077587.1 phage major capsid protein [Salmonella enterica subsp. enterica serovar Braenderup]
373. WP_016587721.1 hypothetical protein [Yersinia pestis]
374. HAX9983652.1 phage major capsid protein [Escherichia coli]
375. WP_075191900.1 MULTISPECIES: phage capsid protein [Cronobacter]
376. HAY3994692.1 phage major capsid protein [Escherichia coli]
377. MBO9360510.1 phage major capsid protein [Thermomicrobium sp.]
378. HAD4280041.1 phage major capsid protein [Salmonella enterica subsp. enterica serovar Typhi str. CT18]
379. HBY0825155.1 phage major capsid protein [Klebsiella pneumoniae]
380. EBH6160155.1 phage major capsid protein [Salmonella enterica]
381. WP_023221754.1 hypothetical protein [Salmonella enterica]
382. MCK5604243.1 phage major capsid protein [Candidatus Pacearchaeota archaeon]
383. EHD3174008.1 phage major capsid protein [Salmonella enterica]
384. ECQ3861573.1 phage major capsid protein [Salmonella enterica]
385. EGQ6262282.1 phage major capsid protein [Salmonella enterica subsp. enterica serovar Enteritidis]
386. WP_114569653.1 phage major capsid protein [Escherichia coli]
387. WP_028449712.1 phage major capsid protein [Chitinibacter tainanensis]
388. ECC6545565.1 phage major capsid protein [Salmonella enterica]
389. MCM6396231.1 phage major capsid protein [Klebsiella pneumoniae]
390. RPJ40005.1 phage major capsid protein [Planctomycetaceae bacterium]
391. EAB0133809.1 phage major capsid protein [Salmonella enterica]
392. HCH9832956.1 phage major capsid protein [Salmonella enterica]
393. WP_169724898.1 phage major capsid protein [Enterobacter hormaechei]
394. ECG2845924.1 phage major capsid protein [Salmonella enterica subsp. enterica serovar Manhattan]
395. EBX1374166.1 phage major capsid protein [Salmonella enterica subsp. enterica serovar Newport]
396. HAX2345276.1 phage major capsid protein [Escherichia coli]
397. UQM93941.1 major head protein [Stenotrophomonas phage vB_Sm_QDWS359]
398. EEU2445140.1 phage major capsid protein [Escherichia coli]

399. EBS5432372.1 phage major capsid protein [Salmonella enterica subsp. enterica serovar Alachua]
400. EAX1817979.1 phage major capsid protein [Salmonella enterica]
401. EBR9946236.1 phage major capsid protein [Salmonella enterica subsp. enterica serovar Krefeld]
402. HBC0308464.1 phage major capsid protein [Salmonella enterica subsp. enterica serovar Napoli]
403. HBZ3547779.1 phage major capsid protein [Klebsiella pneumoniae]
404. MCB2982456.1 phage major capsid protein [Klebsiella pneumoniae]
405. EER4805513.1 phage major capsid protein [Escherichia coli]
406. EGO4917944.1 phage major capsid protein [Salmonella enterica]
407. HBV9739600.1 phage major capsid protein [Klebsiella pneumoniae]
408. HAD4904499.1 phage major capsid protein [Salmonella enterica subsp. enterica serovar Typhi str. CT18]
409. EAB5862671.1 phage major capsid protein [Salmonella enterica subsp. enterica]
410. WP_079825253.1 phage capsid protein [Salmonella enterica]
411. EBE2364545.1 phage major capsid protein [Salmonella enterica]
412. EBR8005981.1 phage major capsid protein [Salmonella enterica subsp. enterica serovar Saintpaul]
413. EFO0755060.1 phage major capsid protein [Escherichia coli]
414. WP_063592850.1 hypothetical protein [Salmonella enterica]
415. EDK9786262.1 phage major capsid protein [Salmonella enterica subsp. enterica serovar Give]
416. ECD3753536.1 phage major capsid protein [Salmonella enterica subsp. enterica serovar Orion]
417. HBN7443553.1 phage major capsid protein [Escherichia coli]
418. WP_252044140.1 phage major capsid protein [Enterobacter asburiae]
419. QHJ78809.1 hypothetical protein [Bacteriophage sp.]
420. EBC2961021.1 phage major capsid protein [Salmonella enterica]
421. EGS7371949.1 phage major capsid protein [Escherichia coli]
422. EEW0868939.1 phage major capsid protein [Escherichia coli]
423. HBQ4879992.1 phage major capsid protein [Escherichia coli]
424. EDZ5493145.1 phage major capsid protein [Salmonella enterica]
425. EFV9537430.1 phage major capsid protein [Shigella sonnei]
426. EFH0306860.1 phage major capsid protein [Escherichia coli]
427. EFW4078867.1 phage major capsid protein [Shigella sonnei]
428. EDU5118278.1 phage major capsid protein [Salmonella enterica subsp. enterica serovar Anatum]
429. EEV7166997.1 phage major capsid protein [Escherichia coli]
430. HBE0118361.1 phage major capsid protein [Shigella sonnei]
431. EAQ8609054.1 phage major capsid protein [Salmonella enterica]
432. QXN72400.1 major capsid protein [Rhodobacter phage RcSimone-Hastad]
433. EFO1692251.1 phage major capsid protein [Escherichia coli]
434. MBD7755531.1 phage major capsid protein [Klebsiella pneumoniae]
435. WP_102003656.1 phage major capsid protein [Klebsiella pneumoniae]
436. EEA1371212.1 phage major capsid protein [Salmonella enterica]
437. MBA8354127.1 phage major capsid protein [Escherichia coli]
438. EGI1978062.1 phage major capsid protein [Salmonella enterica]
439. DAJ05229.1 TPA: MAG TPA: major capsid protein [Siphoviridae sp.]
440. EJF9244945.1 phage major capsid protein [Escherichia coli]
441. WP_015922589.1 phage major capsid protein [Thermomicrobium roseum]
442. WP_181374643.1 phage major capsid protein [Pectobacterium carotovorum]
443. HCH7946627.1 phage major capsid protein [Escherichia coli]
444. WP_223762983.1 phage major capsid protein [Corallococcus sp. AS-1-6]
445. MBW2636049.1 phage major capsid protein [Deltaproteobacteria bacterium]
446. EET1013303.1 phage major capsid protein [Escherichia coli]
447. EIQ1087120.1 phage major capsid protein [Escherichia coli]
448. MCM8749459.1 phage major capsid protein [Thermomicrobiaceae bacterium CFH 74404]
449. EJA7649394.1 phage major capsid protein [Escherichia coli]
450. EIT1058465.1 phage major capsid protein [Escherichia coli]
451. GBD19714.1 hypothetical protein HRbin28_00148 [bacterium HR28]
452. MBX5446219.1 phage major capsid protein [Sphaerobacter sp.]
453. EJA5029925.1 phage major capsid protein [Salmonella enterica]
454. YP_009996955.1 major head protein [Xanthomonas phage Xoo-sp2]
455. WP_224020286.1 hypothetical protein [Burkholderia vietnamiensis]
456. HBQ7547743.1 phage major capsid protein [Klebsiella pneumoniae]
457. EFC1758968.1 phage major capsid protein [Escherichia coli]
458. EDU4451998.1 phage major capsid protein [Salmonella enterica subsp. enterica serovar Anatum]
459. WP_153283238.1 phage major capsid protein [Variovorax paradoxus]
460. HBB2595907.1 phage major capsid protein [Escherichia coli]
461. YP_009997022.1 hypothetical protein JTY56_gp20 [Xanthomonas phage Bosa]
462. EEZ6802210.1 phage major capsid protein [Escherichia coli]
463. MBX3473679.1 hypothetical protein [Planctomycetes bacterium]
464. EFW0729459.1 phage major capsid protein [Shigella sonnei]
465. EHL0287849.1 phage major capsid protein [Escherichia coli]
466. EER8844980.1 phage major capsid protein [Escherichia coli]
467. EFN9767071.1 phage major capsid protein [Escherichia coli]
468. WP_137447163.1 phage major capsid protein, partial [Escherichia coli]
469. MCJ8600995.1 phage major capsid protein [Klebsiella pneumoniae]
470. EFH8846498.1 phage major capsid protein [Escherichia coli]
471. UAW07626.1 major head protein [Pseudomonas phage AIIMS-Plu-RaNi]
472. WP_152881049.1 phage major capsid protein [Duganella sp. FT27W]
473. YP_009196268.1 major head protein [Pseudomonas phage PaMx11]
474. EIB9668287.1 phage major capsid protein [Escherichia coli]
475. HBL5398680.1 phage major capsid protein [Escherichia coli]
476. UIS24744.1 major head protein [Stenotrophomonas phage vB_SmaS-AXL_1]
477. CAH1069075.1 Uncharacterised protein [Acinetobacter phage MD-2021a]
478. WP_163998672.1 phage major capsid protein [Pyxidicoccus caerfyrddinensis]

479. URA07134.1 major capsid protein [Xanthomonas phage Mallos]
480. YP_009996845.1 major head protein [Pseudomonas phage Epa5]
481. EFC2304030.1 phage major capsid protein [Escherichia coli]
482. EHH4005454.1 phage major capsid protein [Escherichia coli]
483. WP_067370658.1 phage capsid protein [Morganella psychrotolerans]
484. EHW0876436.1 phage major capsid protein [Escherichia coli]
485. WP_012870511.1 phage major capsid protein [Sphaerobacter thermophilus]
486. WP_136134600.1 phage major capsid protein [Providencia sp. MGF014]
487. EDS1306404.1 phage major capsid protein [Salmonella enterica]
488. WP_099110294.1 phage major capsid protein [Xenorhabdus sp. KJ12.1]
489. WP_038234216.1 hypothetical protein [Xenorhabdus szentirmaii]
490. UGL62843.1 major head protein [Xanthomonas phage MET13-T1]
491. EIH9254667.1 phage major capsid protein [Escherichia coli]
492. WP_121762769.1 phage major capsid protein [Corallococcus sp. AB038B]
493. EFH8389121.1 phage major capsid protein [Escherichia coli]
494. WP_038038123.1 phage major capsid protein [Thermorudis peleae]
495. HBZ3723017.1 phage major capsid protein [Klebsiella pneumoniae]
496. WP_064173653.1 hypothetical protein, partial [Klebsiella pneumoniae]
497. MCA1800049.1 phage major capsid protein [Actinomycetia bacterium]
498. EFH8991876.1 phage major capsid protein [Escherichia coli]
499. WP_047963505.1 hypothetical protein [Xenorhabdus khoisanae]
500. QNN97178.1 hypothetical protein [Xanthomonas phage Xp12]

```
                10        20        30        40        50        60
1  ---------------------------------------------------MA---LNAQ 6
2  ---------------------------------------------------MA---LNAQ 6
3  ---------------------------------------------------MA---LNAQ 6
4  ---------------------------------------------------MA---LNAQ 6
5  ---------------------------------------------------MA---LNAQ 6
6  ---------------------------------------------------MA---LNAQ 6
7  ---------------------------------------------------MA---LNAQ 6
8  ---------------------------------------------------MA---LNAQ 6
9  ---------------------------------------------------MA---LNAQ 6
10 ---------------------------------------------------MA---LNAQ 6
11 ---------------------------------------------------MA---LNAQ 6
12 ---------------------------------------------------MA---LNAQ 6
13 ---------------------------------------------------MA---LNAQ 6
14 ---------------------------------------------------MP---LNQQ 6
15 ------------M--------------------------------------QP---IGNN 7
16 ------------M--------------------------------------QP---IGAN 7
17 ------------M--------------------------------------QP---IGAN 7
18 ------------M--------------------------------------QP---IGAN 7
19 ------------M--------------------------------------ST---IGEN 7
20 -----------MM--------------------------------------QP---IGNN 8
21 ------------M--------------------------------------ST---IGDN 7
22 ------------M--------------------------------------ST---IGDN 7
23 -----------MM--------------------------------------ST---IGDN 8
24 ------------M--------------------------------------ST---IGDN 7
25 ------------M--------------------------------------ST---IGEN 7
26 ------------M--------------------------------------ST---IGNN 7
27 ------------M--------------------------------------QP---IGAN 7
28 ------------M--------------------------------------ST---IGDN 7
29 ---------------------------------------------------MA---QLAN 6
30 ---------------------------------------------------MA---QLAN 6
31 ---------------------------------------------------MATNTSLQN 9
32 ---------------------------------------------------MATNTSLQN 9
33 ---------------------------------------------------MATNTSLQN 9
34 ---------------------------------------------------MA--TSPAN 7
35 ------------MG------------------------EARLHVHFSREQSMP--STPAN 22
36 ------------------------------------------------------------
37 ---------------------------------------------------MA--STPLN 7
38 ---------------------------------------------------MA----LTN 5
39 ---------------------------------------------------MS---LNQE 6
40 ---------------------------------------------------MA--TIPAN 7
41 ---------------------------------------------------MP---LLGE 6
42 ---------------------------------------------------MP----LAN 5
43 ---------------------------------------------------MP----VAN 5
44 ---------------------------------------------------MP----LAN 5
45 ---------------------------------------------------MA----LTN 5
46 ---------------------------------------------------MP----VTN 5
47 ---------------------------------------------------MP----VTN 5
48 ---------------------------------------------------MP----VTN 5
49 ---------------------------------------------------MP----VTN 5
50 ---------------------------------------------------MP----VTN 5
51 ---------------------------------------------------MP----LSN 5
52 ---------------------------------------------------MP----FVG 5
53 ---------------------------------------------------MP--LTNAQ 7
54 ------------------------------------------------------------
55 ---------------------------------------------------MP--LTNAQ 7
56 ---------------------------------------------------MA---QLAN 6
57 ---------------------------------------------------MP----LTN 5
58 ---------------------------------------------------MA--STPLN 7
59 ---------------------------------------------------MP--LSNTQ 7
60 ---------------------------------------------------MP----FSG 5
61 ---------------------------------------------------MP--LTNTQ 7
62 ---------------------------------------------------MP----VTN 5
63 ---------------------------------------------------MP----YVG 5
```

```
64   ---------------------------------------------------MP--LTNTQ  7
65   ---------------------------------------------------MP--LTNTQ  7
66   ---------------------------------------------------MS----LSN  5
67   ---------------------------------------------------MP----AQN  5
68   ---------------------------------------------------MP--LSPAQ  7
69   ------------MAN----------------------WQFRKENIEERER-MP--LSPAQ  23
70   ---------------------------------------------------MS---LGSF  6
71   MRLHNLRMVAKMMRNVFMILVMNQVDIFFLGGVDFYHIESPALSTFFVGVNMS---LGSF  57
72   -----MRMVAKMMRNVFMILVMNQVDIFFLGGVDFYHIESPALSTFFVGVNMS---LGSF  52
73   ---------------------------------------------------MS---LGGF  6
74   ---------------------------------------------------MP--LLRTT  7
75   ---------------------------------------------------MP----VTN  5
76   ------------------------------------------------------------
77   ---------------------------------------------------ML----FSG  5
78   ---------------------------------------------------MP--LLVTE  7
79   ---------------------------------------------------MA---QLAN  6
80   ---------------------------------------------------MP--LLKEE  7
81   ---------------------------------------------------MA--LLRVE  7
82   ---------------------------------------------------MP--LLREV  7
83   ---------------------------------------------------MP--LLMDE  7
84   ---------------------------------------------------MP--LLIEE  7
85   ---------------------------------------------------MP--LLIEE  7
86   ---------------------------------------------------MP--LLIDE  7
87   ---------------------------------------------------MP---TSG-  5
88   ------------------------------------------------------------
89   ---------------------------------------------------MP--LLIEE  7
90   ---------------------------------------------------MP--LLVEE  7
91   ------------------------------------------------------------
92   ---------------------------------------------------MP---TSG-  5
93   ---------------------------------------------------MP---TSG-  5
94   ---------------------------------------------------MP--LLVQE  7
95   ---------------------------------------------------MP--LLITE  7
96   ---------------------------------------------------MP--LLRVE  7
97   ---------------------------------------------------MP--LLVEE  7
98   ---------------------------------------------------MP--LLREV  7
99   ---------------------------------------------------MP--LLREE  7
100  ---------------------------------------------------MP--LLRTV  7
101  ---------------------------------------------------MG--LLRAD  7
102  ---------------------------------------------------MA--TLGSA  7
103  ---------------------------------------------------MP--LLREV  7
104  ---------------------------------------------------MP--LLREE  7
105  ---------------------------------------------------MP--LLREE  7
106  -------------------------------------------------------LLREE  5
107  ---------------------------------------------------MP--LLKVE  7
108  ---------------------------------------------------MP--LLREE  7
109  ---------------------------------------------------MP--LLREE  7
110  ---------------------------------------------------MP--LLREE  7
111  ---------------------------------------------------MP--LLKVE  7
112  -----------LL--------------------------------------MP--LLREE  9
113  ---------------------------------------------------MP--LLREE  7
114  ---------------------------------------------------MP--LLREE  7
115  ---------------------------------------------------MP--LMRDI  7
116  ---------------------------------------------------MP--LLRAE  7
117  ---------------------------------------------------MP--LLREE  7
118  ---------------------------------------------------MP--LLREE  7
119  ---------------------------------------------------MP--LLRAE  7
120  ---------------------------------------------------MP--LLKVE  7
121  ---------------------------------------------------MP--LLREE  7
122  ---------------------------------------------------MP--LLREE  7
123  ---------------------------------------------------MP--LLREE  7
124  ---------------------------------------------------MP--LLREE  7
125  ---------------------------------------------------MP--LLREE  7
126  ---------------------------------------------------MP--LLREE  7
127  ---------------------------------------------------MP--LLVEE  7
128  ---------------------------------------------------MP--LLVEE  7
129  ---------------------------------------------------MP--LLQAE  7
130  ---------------------------------------------------MP--LLQAE  7
```

```
131 --------------------------------------------------MP--LLQAE 7
132 --------------------------------------------------MP--LLREE 7
133 ------------M-------------------------------------AS--ITFVE 8
134 --------------------------------------------------MP--LLQAE 7
135 --------------------------------------------------MP--LLREE 7
136 --------------------------------------------------MP--LLKEE 7
137 --------------------------------------------------MP--LLRVE 7
138 --------------------------------------------------MP--LLRQE 7
139 --------------------------------------------------MP--LLETE 7
140 --------------------------------------------------MP--LLIDE 7
141 --------------------------------------------------MP--LLKVE 7
142 --------------------------------------------------MP--LLAAE 7
143 --------------------------------------------------MP--LLIDE 7
144 --------------------------------------------------MP--LLTTE 7
145 --------------------------------------------------MP--LLKVE 7
146 --------------------------------------------------MP--LLRVE 7
147 --------------------------------------------------MP--LLRDE 7
148 --------------------------------------------------MP--LLRDE 7
149 --------------------------------------------------MP--LLRDE 7
150 --------------------------------------------------MP--LLRDE 7
151 --------------------------------------------------MP--LLREE 7
152 --------------------------------------------------MP--LLRDE 7
153 --------------------------------------------------MP--LLTTE 7
154 --------------------------------------------------MP--LLREE 7
155 --------------------------------------------------MP--LLREE 7
156 --------------------------------------------------MP--LLRDE 7
157 --------------------------------------------------MP--LLRDE 7
158 --------------------------------------------------MP--LLREE 7
159 --------------------------------------------------MP--LLREE 7
160 --------------------------------------------------MP--LLREE 7
161 --------------------------------------------------MP--LLREE 7
162 --------------------------------------------------MP--LLIDE 7
163 --------------------------------------------------MP--LLREE 7
164 --------------------------------------------------MP--LLRDE 7
165 --------------------------------------------------MP--LLRDE 7
166 --------------------------------------------------MP--LLRDE 7
167 --------------------------------------------------MP--LLRDE 7
168 --------------------------------------------------MP--LLRDE 7
169 --------------------------------------------------MP--LLRDE 7
170 --------------------------------------------------MP--LLREE 7
171 --------------------------------------------------MP--LLRDE 7
172 --------------------------------------------------MP--LLREE 7
173 --------------------------------------------------MP--LLREE 7
174 --------------------------------------------------MP--LLREE 7
175 --------------------------------------------------MP--LLREE 7
176 --------------------------------------------------MP--LLRDE 7
177 --------------------------------------------------MP--LLREE 7
178 --------------------------------------------------MP--LLREE 7
179 --------------------------------------------------MP--LLREE 7
180 --------------------------------------------------MP--LLRDE 7
181 --------------------------------------------------MP--LLREE 7
182 --------------------------------------------------MP--LLREE 7
183 --------------------------------------------------MP--LLREE 7
184 --------------------------------------------------MP--LLREE 7
185 --------------------------------------------------MP--LLREE 7
186 --------------------------------------------------MP--LLREE 7
187 --------------------------------------------------MP--LLREE 7
188 --------------------------------------------------MP--LLREE 7
189 --------------------------------------------------MP--LLREE 7
190 --------------------------------------------------MP--LSKTE 7
191 --------------------------------------------------MP--LLREE 7
192 --------------------------------------------------MP--LLVEE 7
193 --------------------------------------------------MP--LLREE 7
194 --------------------------------------------------MP--LLREE 7
195 --------------------------------------------------MP--LLREE 7
196 --------------------------------------------------MP--LLREE 7
197 --------------------------------------------------MP--LLREE 7
```

```
198 ---------------------------------------------------MP--LLREE 7
199 ---------------------------------------------------MP--LLREE 7
200 ---------------------------------------------------MP--LLREE 7
201 ---------------------------------------------------MP--LLREE 7
202 ---------------------------------------------------MP--LLVEE 7
203 ---------------------------------------------------MP--LLREE 7
204 ---------------------------------------------------MA--LLRVE 7
205 ---------------------------------------------------MP--LLREE 7
206 ---------------------------------------------------MP--LLREE 7
207 ---------------------------------------------------MP--LLREE 7
208 ---------------------------------------------------MP--LLREE 7
209 ---------------------------------------------------MP--LLREE 7
210 ---------------------------------------------------MP--LLREE 7
211 ---------------------------------------------------MP--LLREE 7
212 ---------------------------------------------------MP--LLREE 7
213 ---------------------------------------------------MP--LLREE 7
214 ---------------------------------------------------MP--LLREE 7
215 ---------------------------------------------------MP--LLREE 7
216 ---------------------------------------------------MP--LLREE 7
217 ---------------------------------------------------MP--LLREE 7
218 ---------------------------------------------------MP--LLREE 7
219 ---------------------------------------------------MP--LLREE 7
220 ---------------------------------------------------MP--LLREE 7
221 ---------------------------------------------------MP--LLREE 7
222 ---------------------------------------------------MP--LLREE 7
223 ---------------------------------------------------MP--LLREE 7
224 ---------------------------------------------------MP--LLREE 7
225 ---------------------------------------------------MP--LLREE 7
226 ---------------------------------------------------MP--LLREE 7
227 ---------------------------------------------------MP--LLREE 7
228 ---------------------------------------------------MP--LLRAE 7
229 ---------------------------------------------------MP--LLREE 7
230 ---------------------------------------------------MP--LLRAE 7
231 ---------------------------------------------------MP--LLREE 7
232 ---------------------------------------------------MP--LLREE 7
233 ---------------------------------------------------MP--LLREE 7
234 ---------------------------------------------------MP--LLREE 7
235 ---------------------------------------------------MP--LLREE 7
236 ---------------------------------------------------MP--LLREE 7
237 ---------------------------------------------------MP--LLREE 7
238 ---------------------------------------------------MP--LLREE 7
239 ---------------------------------------------------MA--LTKAE 7
240 ---------------------------------------------------MP--LLREE 7
241 ---------------------------------------------------MP--LLRDE 7
242 ---------------------------------------------------MP--LLREE 7
243 ---------------------------------------------------MP--LLREE 7
244 ---------------------------------------------------MP--LLREE 7
245 ---------------------------------------------------MP--VLREE 7
246 ---------------------------------------------------MP--LLREE 7
247 ---------------------------------------------------MP--LLREE 7
248 ---------------------------------------------------MP--LLREE 7
249 ---------------------------------------------------MP--LLREE 7
250 ---------------------------------------------------MP--LLREE 7
251 ---------------------------------------------------MP--LLRDE 7
252 ---------------------------------------------------MP--LLREE 7
253 ---------------------------------------------------MP--LLREE 7
254 ---------------------------------------------------MP--LLREE 7
255 ---------------------------------------------------MP--LLREE 7
256 ---------------------------------------------------MP--LLRDE 7
257 ---------------------------------------------------MP--LLRDE 7
258 ---------------------------------------------------MP--LLREE 7
259 ---------------------------------------------------MP--LLREE 7
260 ---------------------------------------------------MP--LLREE 7
261 ---------------------------------------------------MP--LLREE 7
262 ---------------------------------------------------MP--LLRDE 7
263 ---------------------------------------------------MP--LLREE 7
264 ---------------------------------------------------MP--LLRDE 7
```

```
265 ---------------------------------------------------MP--LLREE 7
266 ---------------------------------------------------MP--LLREE 7
267 ---------------------------------------------------MP--LLREE 7
268 ---------------------------------------------------MP--LLREE 7
269 ---------------------------------------------------MP--LLRDE 7
270 ---------------------------------------------------MP--LLRAE 7
271 ---------------------------------------------------MP--LLREE 7
272 ---------------------------------------------------MP--LLREE 7
273 ---------------------------------------------------MP--LLREE 7
274 ---------------------------------------------------MP--LLREE 7
275 ---------------------------------------------------MP--LLRDE 7
276 ---------------------------------------------------MP--LLREE 7
277 ---------------------------------------------------MP--LLRDE 7
278 ---------------------------------------------------MP--LLREE 7
279 ---------------------------------------------------MP--LLREE 7
280 ---------------------------------------------------MP--LLREE 7
281 ---------------------------------------------------MP--LLREE 7
282 ---------------------------------------------------MP--LLREE 7
283 ---------------------------------------------------MP--LLREE 7
284 ---------------------------------------------------MP--LLREE 7
285 ---------------------------------------------------MP--LLREE 7
286 ---------------------------------------------------MP--LLRDE 7
287 ---------------------------------------------------MP--LLREE 7
288 ---------------------------------------------------MP--LLRDE 7
289 ---------------------------------------------------MP--LLREE 7
290 ---------------------------------------------------MP--LLRDE 7
291 ---------------------------------------------------MP--LLREE 7
292 ---------------------------------------------------MP--LLREE 7
293 ---------------------------------------------------MP--LLREE 7
294 ---------------------------------------------------MP--LLREE 7
295 ---------------------------------------------------MP--LLREE 7
296 ---------------------------------------------------MP--LLREE 7
297 ---------------------------------------------------MP--LLREE 7
298 ---------------------------------------------------MP--LLREE 7
299 ---------------------------------------------------MP--LLREE 7
300 -----------------------------------------------------------E 1
301 ---------------------------------------------------MP--LLREE 7
302 ---------------------------------------------------MP--LLREE 7
303 ---------------------------------------------------MP--LLRDE 7
304 ---------------------------------------------------MP--LLREE 7
305 ---------------------------------------------------MP--LLREE 7
306 ---------------------------------------------------MP--LLREE 7
307 ---------------------------------------------------MP--LLRDE 7
308 ---------------------------------------------------MP--LLREE 7
309 ---------------------------------------------------MP--LLRDE 7
310 ---------------------------------------------------MP--LLRDE 7
311 ---------------------------------------------------MP--LLRDE 7
312 ---------------------------------------------------MP--LLREE 7
313 ---------------------------------------------------MP--LLREE 7
314 ---------------------------------------------------MP--LLRDE 7
315 ---------------------------------------------------MP--LLREE 7
316 ---------------------------------------------------MP--LLREE 7
317 ---------------------------------------------------MP--LLRDE 7
318 ---------------------------------------------------MP--LLREE 7
319 ---------------------------------------------------MP--LLREE 7
320 ---------------------------------------------------MP--LLRDE 7
321 ---------------------------------------------------MP--LLREE 7
322 ---------------------------------------------------MP--LLRDE 7
323 ---------------------------------------------------MP--LLRDE 7
324 ---------------------------------------------------MP--LLREE 7
325 ---------------------------------------------------MP--LLREE 7
326 ---------------------------------------------------MP--LLREE 7
327 ---------------------------------------------------MP--LLREE 7
328 ---------------------------------------------------MP--LLREE 7
329 ---------------------------------------------------MP--LLRDE 7
330 ---------------------------------------------------MP--LLRDE 7
331 ---------------------------------------------------MP--LLREE 7
```

```
332 --------------------------------------------------MP--LLRDE 7
333 --------------------------------------------------MP--LLRDE 7
334 --------------------------------------------------MP--LLRDE 7
335 --------------------------------------------------MP--LLRDE 7
336 --------------------------------------------------MP--LLREE 7
337 --------------------------------------------------MP--LLRDE 7
338 --------------------------------------------------MP--LLREE 7
339 --------------------------------------------------MP--LLRDE 7
340 --------------------------------------------------MP--LLREE 7
341 --------------------------------------------------MP--LLRDE 7
342 --------------------------------------------------MP--LLREE 7
343 --------------------------------------------------MP--LLRDE 7
344 --------------------------------------------------MP--LLRDE 7
345 --------------------------------------------------MP--LLREE 7
346 --------------------------------------------------MP--LLRDE 7
347 --------------------------------------------------MP--LLREE 7
348 --------------------------------------------------MP--LLREE 7
349 --------------------------------------------------MP--LLRDE 7
350 --------------------------------------------------MP--LLRDE 7
351 -----------------------------------------------------------
352 --------------------------------------------------MP--LLRDE 7
353 ------------M-------------------------------------SS--VTLLE 8
354 -------------------------------------------------------LREE 4
355 --------------------------------------------------MP--LLRDE 7
356 --------------------------------------------------MP--LLRDE 7
357 --------------------------------------------------MP--LLREE 7
358 --------------------------------------------------MP--LLRDE 7
359 --------------------------------------------------MP--LLREE 7
360 --------------------------------------------------MP--LLRDE 7
361 --------------------------------------------------MP--LLREE 7
362 --------------------------------------------------MP--LLREE 7
363 --------------------------------------------------MP--LLRDE 7
364 --------------------------------------------------MP--LLRDE 7
365 --------------------------------------------------MP--LLRDE 7
366 --------------------------------------------------MP--LLRDE 7
367 --------------------------------------------------MP--LLREE 7
368 --------------------------------------------------MP--LLREE 7
369 --------------------------------------------------MP--LLRDE 7
370 --------------------------------------------------MP--LLRDE 7
371 --------------------------------------------------MP--LLRDE 7
372 --------------------------------------------------MP--LLRDE 7
373 --------------------------------------------------MP--LLRDE 7
374 --------------------------------------------------MP--LLRDE 7
375 --------------------------------------------------MP--LLRDE 7
376 --------------------------------------------------MP--LLREE 7
377 --------------------------------------------------MA--LTKVE 7
378 --------------------------------------------------MP--LLRDE 7
379 --------------------------------------------------MP--LLRDE 7
380 --------------------------------------------------MP--LLREE 7
381 --------------------------------------------------MP--LLREE 7
382 ------------M-------------------------------------AS--VTLAE 8
383 --------------------------------------------------MP--LLREE 7
384 --------------------------------------------------MP--LLREE 7
385 --------------------------------------------------MP--LLRDE 7
386 --------------------------------------------------MP--LLREE 7
387 --------------------------------------------------MP--LLQAV 7
388 --------------------------------------------------MP--LLREE 7
389 --------------------------------------------------MP--LLREE 7
390 ------------M-------------------------------------AS--ITFAE 8
391 --------------------------------------------------MP--LLREE 7
392 --------------------------------------------------MP--LLRDE 7
393 --------------------------------------------------MP--LLRDE 7
394 --------------------------------------------------MP--LLREE 7
395 --------------------------------------------------MP--LLREE 7
396 --------------------------------------------------MP--LLREE 7
397 ------------M-------------------------------------SS--VTLAE 8
398 --------------------------------------------------MP--LLREE 7
```

399 ---MP--LLRDE 7
400 ---MP--LLREE 7
401 ---MP--LLREE 7
402 ---MP--LLREE 7
403 ---MP--LLREE 7
404 ---MP--LLREE 7
405 ---MP--LLREE 7
406 ---MP--LLREE 7
407 ---MP--LLREE 7
408 ---MP--LLRDE 7
409 ---MP--LLREE 7
410 ---MP--LLREE 7
411 ---MP--LLREE 7
412 ---MP--LLREE 7
413 ---MP--LLREE 7
414 ---MP--LLREE 7
415 ---MP--LLREE 7
416 ---MP--LLREE 7
417 ---MP--LLREE 7
418 ---MP--LLRDE 7
419 ---MS--LLVEE 7
420 ---MP--LLREE 7
421 ---MP--LLREE 7
422 ---MP--LLREE 7
423 ---MP--LLREE 7
424 ---MP--LLREE 7
425 ---MP--LLREE 7
426 ---MP--LLREE 7
427 ---MP--LLREE 7
428 ---MP--LLREE 7
429 ---MP--LLREE 7
430 ---MP--LLREE 7
431 ---MP--LLREE 7
432 ------------M--PS--VTLTE 8
433 ---MP--LLREE 7
434 ---MP--LLREE 7
435 ---MP--LLREE 7
436 ---MP--LLREE 7
437 ---MP--LLREE 7
438 ---MP--LLREE 7
439 ---MP--LLRAE 7
440 ---MP--LLREE 7
441 ---MA--LTKVE 7
442 ---MP--LLREE 7
443 ---MP--LLREE 7
444 ------------M--PS--VILSE 8
445 ------------M--AS--VTLAE 8
446 ---MP--LLREE 7
447 ---MP--LLREE 7
448 ---MTKVE 5
449 ---MP--LLREE 7
450 ---MP--LLREE 7
451 ---MA--LTKVE 7
452 ---MA--LTKVE 7
453 ---MP--LLREE 7
454 ------------M--SS--VTLAE 8
455 ---MP--LLQAE 7
456 ---MP--LLREE 7
457 ---MP--LLREE 7
458 ---MP--LLREE 7
459 ---MP--LLKAT 7
460 ---MP--LLREE 7
461 ------------M--AS--VTLAE 8
462 ---MP--LLREE 7
463 --
464 ---MP--LLREE 7
465 ---MP--LLREE 7

```
466  ---------------------------------------------------MP--LLREE  7
467  ---------------------------------------------------MP--LLREE  7
468  ---------------------------------------------------MP--LLREE  7
469  ---------------------------------------------------MP--LLREE  7
470  ---------------------------------------------------MP--LLREE  7
471  ------------M--------------------------------------AS--VTLVE  8
472  ---------------------------------------------------MP--LLRTE  7
473  ------------M--------------------------------------AS--VTLVE  8
474  ---------------------------------------------------MP--LLREE  7
475  ---------------------------------------------------MP--LLRDE  7
476  ------------M--------------------------------------AS--VTLAE  8
477  ------------M--------------------------------------AT--ITLAE  8
478  ------------M--------------------------------------PS--NPLIE  8
479  ------------M--------------------------------------AS--VTLTE  8
480  ------------M--------------------------------------AS--VTLAE  8
481  ---------------------------------------------------MP--LLREE  7
482  ---------------------------------------------------MP--LLREE  7
483  ---------------------------------------------------MP--LLREE  7
484  ---------------------------------------------------MP--LLREE  7
485  ---------------------------------------------------MA--LTKVE  7
486  ---------------------------------------------------MP--LLREE  7
487  ---------------------------------------------------MP--LLREE  7
488  ---------------------------------------------------MP--LLREE  7
489  ---------------------------------------------------MP--LLREE  7
490  ------------M--------------------------------------AS--VTLAE  8
491  ---------------------------------------------------MP--LLREE  7
492  ------------M--------------------------------------PS--VILSE  8
493  ---------------------------------------------------MP--LLREE  7
494  ---------------------------------------------------MA--LTKLE  7
495  ---------------------------------------------------MP--LLREE  7
496  ---------------------------------------------------MP--LLREE  7
497  ------------M--------------------------------------AS--VTLAE  8
498  ---------------------------------------------------MP--LLREE  7
499  ---------------------------------------------------MP--LLREE  7
500  ------------M--------------------------------------AS--VTLAE  8
```

```
              70        80        90        100       110       120
1    AQFLSILELTKGLLENSPKRLQHLEVFPFHHVEGDSLRYVR------TPELTP----ASI  56
2    AQFLSILELTKGLLENSPKRLQHLEVFPFHHVEGDSLRYVR------TPELTP----ASI  56
3    AQFLSILELTKGLLENSPKRLQHLEVFPFHHVEGDSLRYVR------TPALTP----ASI  56
4    AQFLSILELTKGLLENSPKRLQHLEIFPFHHVEGDSLRYVR------TPELTP----ASI  56
5    AQFLSILELTKGLLENSPKRLQHLEIFPFHHVEGDSLRYVR------TPELTP----ASI  56
6    AQFLSILELTKGLLENSPKRLQHLEVFPFHHVEGDSLRYVR------TPELTP----ASI  56
7    AQFLSILELTKGLLENSPKRLQHLEIFPFHHVEGDSLRYVR------TPELTP----ASI  56
8    AQFLSILELTKGLLENSPKRLQHLEVFPFHHVEGDSLRYVR------TPELTP----ASI  56
9    AQFLSILELTKGLLENSPKRLQHLEIFPFHHVEGDSLRYVR------TPELTP----ASI  56
10   AQFLSILELTKGLLENSPKRLQHLEIFPFHHVEGDSLRYVR------TPELTP----ASI  56
11   AQFLSILELTKGLLENSPKRLQHLEVFPFHHVEGDSLRYVR------TPELTP----ASI  56
12   AQFLSILELTKGLLENSPKRLQHLEVFPFHHVEGDSLRYVR------TPELTP----ASI  56
13   AQFLSILELTKGLLENSPKRLQHLEIFPFHHVEGDSLRYVR------TPELTP----ASI  56
14   VQFLSIDALALGLIENAPKRMQYLPFFPFYSIKGSALRYVR------TPELVP----AEI  56
15   ARLLSNDVLAKWLLEDFPFRIQLSTAFPFLPVAGDALRYAT------TGPLIP----GIA  57
16   AQLLSNDALAKWLIEDFPFRIQLSSIFPFIPVSGHSLRYAA------TGALTP----GNA  57
17   AQLLSNDALAKWLVEDFPFRIQLSSIFPFIPVSGHSLRYAT------TDALMP----GNA  57
18   AQLLANDALAKWLIEDFPFRIQLSSIFPFLPVAGDSFRYAT------TGLLNP----GST  57
19   ARLLSNDVQAKWLIEDFPFRIQLASVLPFLPVAGDALRYTT------TPVLQP----GVT  57
20   ARLLSNDVLAKWLLEDFPFRIQLSTAFPFLPIAGDALRYAT------TGPLTP----GVT  58
21   ARLLSNDVQAKWLIEDFPFRIQLASVFPFYPVAGDALRYTT------TPVLQP----GVT  57
22   ARLLSNDVQAKWLIEDFPFRIQLASVFPFCPVAGDALRYTT------TPILQP----GVM  57
23   ARLLSNDVQAKWLIEDFPFRIQLASVFPFYPVAGDALRYTT------TPVLQP----GVT  58
24   ARLLSNDVQAKWLIEDFPFRIQLASALPFYPVAGDALRYTT------TPVLQP----GVT  57
25   ARLLSNDVQAKWLIEDFPFRIQLASVLPFLPVAGDALRYTT------TSVLQP----GVT  57
26   ARLLSNDVQAKWLIEDFPFRIQLASVFPFYPVAGDALRYTT------TPVLQP----GVT  57
```

27	AQLLSNDVLAKWLIEDFPFRIQLSSIFPFLSVSGHSLRYAT------TGPLIP----GAA	57
28	ARLLSNDVQAKWLIEDFPFRIQLASALPFYPVAGDALRYTT------TPVLQP----GVT	57
29	ADLLTLRDLWPWLVENFPWKIQIAHHWPFMGIDGGFLRVAR------TARFDQ----TDL	56
30	ADLLTLRDLWPWLVENFPWKIQIAHHWPFMGIDGGFLRVARTARFDQTARFDQ----TDL	62
31	ADRLVLTDQWQWLIENFPWKLQIARFWPFMGIYGGFLRVPR------TNQFDK----SEL	59
32	ADRLVLTDQWQWLIENFPWKLQIARFWPFTGIHGGFLRVAR------TNLIDK----SDL	59
33	ADRLVLTDQWQWLIENFPWKLQIARFWPFMGIHGGFLRVAR------TNQISN----ADL	59
34	STNLTQDQLAKWLIEDAPYKIQIARVWPFMGIQGGKISYAR------TAALESLANIDDM	61
35	AVNLTQEQLSKFLLETIEYKLQIMQIWPFMGVLGGKLRYER------TNQLEAIADINNM	76
36	--	
37	SINLTQDQLAKWLIEDMPYRIQIARIFPFMGIQGGKISYAR------TPALEAVADINDM	61
38	VSLLLQKLKQSWLIENFPYKLQIPRAWAWKGIEGTQVSFAR------TPAFNK----ASL	55
39	ANRLSTNHLLPGLQENAPKRLDILKALFLLQITGNALAVVR------TPALPD----ALA	56
40	SINLTQDQLAKWLIEDAPYKIQIARIWPFMGIQGGKISYAR------IADLTTGFSFDTV	61
41	AQYLSTDELLPGLLENWSKEIPGLECFPFYEIRGNALTAVA------TDALPD----AGT	56
42	VSRLLQLLRQAWLIENFPHKLQIPRAWVWKAIEGTQVAFAR------TNRMDP----AEL	55
43	VERFMQLLRQSWLVENFPHKLQIPRRWIWHGIAGTRVAFPR------TNRIDK----DNL	55
44	VSRLLQLLRQAWLIENFPHKLQIPRAWVWKAIEGTQVAFAR------INRMDP----AEL	55
45	VSMLLQKLKQSWLIENFPHKLQIPRAWAWKGIEGTQVSFAR------TPQLDK----ATL	55
46	ADLLSDDLLALWLVENAPIKIPFFQAWSWQQVAGKSLIYPR------TTALAP----ASV	55
47	ADLLADDLLALSLVENSPVKIPFFQAWIWQEVAGKSLTYAR------TPLLAP----ATE	55
48	ADLLSDDLLALWLVENAPIKIPFFQAWAWQQVAGKSLIYPR------TTALAP----AGV	55
49	ADLLSDDLLALWLVENAPIKIPFFQAWSWQQVAGKSLIYPR------TTALAP----AGV	55
50	ADLLSDDLLALWLVENAPIKIPFFQAWAWQQVAGKSLIYPR------TTTLSP----AGV	55
51	AFLLGDSPVARWLVENSPVAIPFLDYWAWQGVDGQSLTYPR------ATELTP----ASV	55
52	AANATTQQATRWLVENEPEIIQIHGHFPSQAVQGGFLTYAR------VPKLIE----ASP	55
53	AQLLSEEALLAGLVENDPKRIQLARRLPFYAVGGDRLAVPRV----TSGNLGT----AVW	59
54	-------MLSKFLLEELEYKLQIARIWPFMGVVGGKLRYER------TDQLETVANIDSM	47
55	ALLLSNDAFIKGLIEAEPKRIQMSHIFPFFEIDGDKLTVPRV----TSANLGT----AIF	59
56	ADLLTRRDLWPWLVENFPWKIQIARHWPFMGIDGGFLRVAR------TTRFDE----TDL	56
57	ASLLSENRRTRFIVENSPIKLQIARVWPWLGVDGSGNMYAR-----KNAQLTP----TPV	56
58	AVDLTQEQLVKTVIEKIRYKVEIANFWPMRGVIGGKLRYAR------TDQLSA-GDINAM	60
59	ASLLSDEQLLSNLVENEPKRIQFALRFPFYAIGGDRLTVPRV----TAANLGT----AVW	59
60	ASGTANTIATPHAVENEPRLIQIHRRMATKGVPGGTLVYPR------VPALKP----ASV	55
61	ALLLSNDAFLQGLVENDPKRIQMSNLLPFFEIDGDKLTIPRV----TNANLGT----AIW	59
62	ADLLSDDLLALWLVENAPIKIPFFQAWSWQQVAGKSLIYPR------TTALAP----AVM	55
63	AQNAVNQIATPWLVENDPEIIQVHGHWAWQGVPGGLLTYAR------VPKLIA----ASA	55
64	ALLLSNDALLAGLVENDPKRIQLSRRLPFYALAGDRLTVPRV----TNANLGT----ALW	59
65	ALLLSNDAFLQGLTENEPRRIQMSHVLEFFEIDGDKLTIPRV----TSANLGT----AIF	59
66	AYMLGESPVARWLIENSPINIAFYQAWAWQGVDGQSLTYPR------ATELSP----AAV	55
67	TQFLNSIPVARYAVERAPTRIQHLMLWSFQGVAGRELLYAQ------VPQLVA----SGQ	55
68	GLLLSNDFLVGRFIEDSPFVIQPAHFLPFFEVPGDSERWST------TAAILP----ASA	57
69	GLLLSNDFLVGRFIEDSPFVIQPAHFLPFFEVSGDSERWST------TVAIPP----ASP	73
70	AQALAEEKVAKWLLENWPVKLQFGLVCPITWVDGKSLKFNSLTD-PAGNPIPF----AYP	61
71	AQALAEEKITKWLLENWPVKLQFGLVCPITWVDGKSLKFNSLTD-AGGNPMPF----AYP	112
72	AQALAEEKITKWLLENWPVKLQFGLVCPITWVDGKSLKFNSLTD-AGGNPMPF----AYP	107
73	AQALAEEKVTRWLLENWPVKLQFGLVCPITWVDGKSLKFNSLTD-PDGNPIPF----AYP	61
74	AELLSQEQMERGIIEEIIDKDDLFAVLPFKQVVGKAYVYDR------ELTLSE----GDF	57
75	ADLLADDLLALWLVENSPVKIPFYQAWIWQEVAGKSLTYAR------TPLLAP----ATI	55
76	--	
77	APDTANIQAAKWFVENEPRRIQIHGVMPVFGVNGGFLVYPR------VPAIKP----AQV	55
78	AAKLSREQFERGVIEEIIDKDELFALFPFMKVEGKTYDYVR------EGTLSE----GDF	57
79	ADLLTLRDLWPWLVENFPWKIQIARHWPFMGINGGFLRVAR------TARFDE----TDL	56
80	AAKLSNNDLVAGVIEEVIDKEDLLAVLPFTETKGKAYVYNR------ENFISE----AVF	57
81	AEKLSNNQLVSGVIEEIIDKDELFALLPFMGVNGKAYVYNR------ENTISE----GDF	57
82	AETLTNNDLVAGIVEEIIERDDMFAILPFVHTEGKAYVYNR------EKTLAE----GVF	57
83	AAKLSQEDKARGVIEEIIDTESLFALMPFQHVNDKTFTYVR------EGTLSE----GEF	57
84	AAKLSQEDKARGVIEEIIDTESLFALMPFQHVTDKTFTYVR------EGTLSE----GEF	57
85	AAKLSQEDKARGVIEEIIDTESLFALMPFQHVTDKTFTYVR------EGTLSE----GEF	57
86	AAKLSQEDKARGVIEEIIDDESLFALMPFQHVTDKTFTYVR------EGTLSE----GEF	57
87	AENAGQNFLQPHIVETAPVPMQIARHMPVQFVNGRAKSFAVI------GKLEP----GVA	55
88	---M	1
89	ADKLSQEDKARGVIEEIIDTESLFALMPFQHVSDKTFTYVR------EGTLSE----GEF	57
90	AAKLSQEQMERGVIEEIIDTDELFALIPFMGVNGKAYVYNR------EDTLSE----GEF	57
91	------------------------------------MKYAR------SAVLAA----ATK	14
92	AENAGQNFLQPHIVETAPVPLQIARHMPVRYINGRALSFAVI------DAMAA----GVA	55
93	AENAGSDFLQPHIVETAPVPLQIARHMPVRYVNGRAMSFAVI------GKMQP----GIA	55

```
94   AAKLSEADLQRGIIEEIIDKEDLLAVLPFVRTDGKSYDYVR------EKTLSE----AEF  57
95   AEKLSQEDKARGVIEEIIDTESLFALMPFQHVNDKTFTYVR------EGTLSE----GEF  57
96   AERLSNNQLVAGIIEEIIERDDLFAILPFTGINGKAYVYNR------ENTLAG----ASW  57
97   AAKLSVNQLERGVIEEIIDVDELFALFPFMQVNGKAYDYIR------ESTLSE----GDF  57
98   AEKLSQEEMLRGVIEEIIDRDDLLALLPFARTEGKALVYIR------ELTNSE----GSF  57
99   AEKLSQDYLERGVIEEIIDQDEMFALVPFMFVNGKAYDYVR------EKELSE----GAF  57
100  AETLSQEDMLRGVIEEIIHRDDLLALLPFSRVDGKALVYIR------ELTNSE----GTF  57
101  AEKLSNNTLVQGIVEELIDRDELFSLLPFEQVNSKAYVYNR------EKTLSE----ADF  57
102  AQYLSRDALAMWLLETDPWRNELAAVFPYMPIDGPALKVAR------QTKVAI----GSL  57
103  AEKLSQEEMLRGVIEEIIDRDDLLALLPFARTEGKALVYIR------ELTNSE----GSF  57
104  AAKLSNNQLVSGVVEEIIDKDEMFAFLPFVGVKGKAYVYNR------EKTLAD----ADF  57
105  AEKLSNNQLVQGVVEEIIDRDDLFAILPFTQVNGKAYVYNR------EKTLGG----ANW  57
106  AEKLSNNQLVQGVVEEIIDRDDLFAILPFTQVNGKAYVYNR------EKTLGG----ANW  55
107  AEKLSNNQLVSGVIDEIIFRDDMFAILPFMGVNGKAYVYNR------ENTLGD----AEF  57
108  AEKLSNNQLVQGVVEEIIDRDDLFAILPFTQVNGKAYVYNR------EKTLGG----ANW  57
109  AEKLSNNQLVQGVVEEIIDRDDLFAILPFTQVNGKAYVYNR------EKTLGG----ANW  57
110  AEKLSNNQLVQGVVEEIIDRDDLFAILPFTQVNGKAYVYNR------EKTLGG----ANW  57
111  ADKLSNNQLERGVIEEVIERDELFALFPFMRVNSKAYVYNR------ENGLSG----AAF  57
112  AEKLSNNQLVQGVVEEIIDRDDLFAILPFTQVNGKAYVYNR------EKTLGG----ANW  59
113  AEKLSNNQLVQGVVEEIIDRDDLFAILPFTQVNGKAYVYNR------EKTLGG----ANW  57
114  AEKLSNNQLVQGVVEEIIDRDDLFAILPFTQVNGKAYVYNR------EKTLGG----ANW  57
115  AETLSQEDMVRGVIEEIVHKDDLMALMPFSRTDGKAYVYVR------EKTNSE----GSF  57
116  AEKLSNNQLLRGVITEIIDKDDMFAILPFTQVNGKAYVYNR------EGELAD----GQW  57
117  AAKLSNNQLVSGVVEEIIDKDEMFAFLPFVGVKGKAYVYNR------EKTLAD----ADF  57
118  AAKLSNNQLVSGVVEEIIDKDEMFAFLPFVGVKGKAYVYNR------EKTLAD----ADF  57
119  AEKLSNNQLLRGVITEIIDKDDMFAILPFTQVNGKAYVYNR------EGELAD----GQW  57
120  AEKLSQDQVVQGIVEEIFTTNEMYNMLPFTKVSGKAYLYKR------EGTLAT----VDY  57
121  AEKLSNNQLVQGVVEEIIDRDDLFAILPFTQVNGKAYVYNR------EKTLGG----ANW  57
122  AEKLSNNQLVQGVVEEIIDRDDLFAILPFTQVNGKAYVYNR------EKTLGG----ANW  57
123  AEKLSNNTLVAGVIDEIIERDDLFSVLPFVAVNSKAYVYNR------EATLAD----ADF  57
124  AEKLSNNQLVQGVVEEIIDRDDLFAVLPFVQVNGKAYVYNR------EKTLGG----ANW  57
125  AEKLSNNQLVQGVVEEIIDRDDLFAILPFTQVNGKAYVYNR------EKTLGG----ANW  57
126  AEKLSNNQLVQGVVEEIIDRDDLFAILPFTQVNGKAYVYNR------EKTLGG----ANW  57
127  AAKLSVDQLERGIIEEIIVQDDLFALFPFVHVNGKAYDYIR------EATLSE----GSF  57
128  AAKLSVDQLERGIIEEIIVQDDLFALFPFVHVNGKAYDYIR------EATLSE----GAF  57
129  AEKLSNNQLVAGVIEEIIERDELFALIPFIGINGKAYVYDR------ENSLPT----ADF  57
130  AEKLSNNQLVAGVIEEIIERDELFALIPFIGINGKAYVYDR------ENTLPT----ADF  57
131  AEKLSNNQLVAGVIEEIIERDELFALIPFIGINGKAYVYDR------ENTLPT----ADF  57
132  AEKLSNNQLVQGVVEEIIDRDDLFAILPFTQVNGKAYVYNR------EKTLGG----ANW  57
133  AAKLCQDELVSGLIENVITVNPFYNILPFDSIDGNGLTYNR------EDVLGD----VQV  58
134  AEKLSNNQLVAGVIEEIIERDELFALIPFIGINGKAYVYDR------ENSLPT----ADF  57
135  AEKLSNNQLVQGVVEEIIDRDDLFAILPFTQVNGKAYVYNR------EKTLGG----ANW  57
136  AAKLSNNQLEAGVITEIYTRDDLFAVLPFTRVNGKAYVYNR------ENELAT----ADW  57
137  AEKLSNNVLEQGVIEEIIDNDAMFAMLPFKRVVGKAYMYNR------EKTLSE----GEF  57
138  AETLSNNQLVAGVIDQIIDRDDLFAILPFERVNGKAYVYNR------EDTLGG----ADW  57
139  ANKLSESLMERGVIEEIIDREALFALFPFKTVDGKAYLYHR------ENTISE----GDF  57
140  AKKLSNNELEQGIIETIIDREELFAVLPFMKVNSKAYLYNR------EKELSE----ASF  57
141  ADKLSNNDLVRGVIEEVIDRDDLFAVLPFTRTDGKAYVYNR------ENGVVE----AEF  57
142  AAKLSIEDRQRGVIEEIIKDDALFALLPFVGVTDEVYSYVR------EATLAQ----GAF  57
143  AKKLSNNELEQGIIETIIDREELFAVLPFMKVNSKAYLYNR------EKELSE----ASF  57
144  AEKLSQEDMERGVIEEIIDKDELFALVPFMHVTDKTYTYVR------EGNLPS----AEF  57
145  AEKLSEEFMERGVIEEIIDRDELFQFLPFMGVDNKAYTYNR------EGTLSE----GQF  57
146  AEKLSNNILEQGVIEEIIDNDAMFALLPFKRIVGKAYVYNR------EKSLSE----GAF  57
147  AEKLSNNELEQGVIETIIDRDDLFAVLPFMKIDSKAYLYNR------EATLSE----ATF  57
148  AEKLSNNELEQGVIETIIDRDDLFAVLPFMKIDSKAYLYNR------EATLSE----ATF  57
149  AEKLSNNELEQGVIETIIDRDDLFAVLPFMKIDSKAYLYNR------EATLSE----ATF  57
150  AEKLSNNELEQGVIETIIDRDDLFAVLPFMKIDSKAYLYNR------EATLSE----ATF  57
151  AEKLSNNELEQGVIETIIDRDDLFAILPFMKINSKAYLYNR------EATLSE----ATF  57
152  AEKLSNNELEQGVIETIIDRDDLFAVLPFMKIDSKAYLYNR------EATLSE----ATF  57
153  AEKLSQEDMERGVIEEIIDKDELFALVPFMHVTDKTYTYVR------EGNLPS----AEF  57
154  AEKLSNNELEQGVIETIIDRDDLFAVLPFMKIDSKAYLYNR------EATLSE----ATF  57
155  AEKLSNNELEQGVIETIIDRDDLFAILPFMKINSKAYLYNR------EATLSE----ATF  57
156  AEKLSNNELEQGVIETIIDRDDLFAVLPFMKIDSKAYLYNR------EATLSE----ATF  57
157  AEKLSNNELEQGVIETIIDRDDLFAVLPFMKIDSKAYLYNR------EATLSE----ATF  57
158  AEKLSNNELEQGVIETIIDRDDLFAILPFMKINSKAYLYNR------EATLSE----ATF  57
159  AEKLSNNELEQGVIETIIDRDDLFAILPFMKINSKAYLYNR------EATLSE----ATF  57
160  AEKLSNNELEQGVIETIIDRDDLFAILPFMKINSKAYLYNR------EATLSE----ATF  57
```

161 AAKLSNNQLVSGVVEEIIDKDEMFAFLPFVGVKGKAYVYNR------EKTLAD----ADF 57
162 AKKLSNNQLEIGVVEEIIDKEDLFSVLPFMGINGKAYVYNR------EKTLSE----GDF 57
163 AEKLSNNELEQGVIETIIDRDDLFSILPFMKINSKAYLYNR------EATLSE----ATF 57
164 AEKLSNNELEQGVIETIIDRDDLFAVLPFMKIDSKAYLYNR------EATLSE----ATF 57
165 AEKLSNNELEQGVIETIIDRDDLFAVLPFMKIDSKAYLYNR------EATLSE----ATF 57
166 AEKLSNNELEQGVIETIIDRDDLFAVLPFMKIDSKAYLYNR------EATLSE----ATF 57
167 AEKLSNNELEQGVIETIIDRDDLFAVLPFMKIDSKAYLYNR------EATLSE----ATF 57
168 AEKLSNNELEQGVIETIIDRDDLFAVLPFMKIDSKAYLYNR------EATLSE----ATF 57
169 AEKLSNNELEQGVIETIIDRDDLFAVLPFMKIDSKAYLYNR------EATLSE----ATF 57
170 AEKLSNNELEQGVIETIIDRDDLFAILPFMKINSKAYLYNR------EKTLSE----ATF 57
171 AEKLSNNELEQGVIETIIDRDDLFAVLPFMKIDSKAYLYNR------EATLSE----ATF 57
172 AEKLSNNELEQGVIETIIDRDDLFAILPFMKINSKAYLYNR------EATLSE----ATF 57
173 AEKLSNNELEQGVIETIIDRDDLFAILPFMKINSKAYLYNR------EKTLSE----ATF 57
174 AEKLSNNELEQGVIETIIDRDDLFAILPFMKINSKAYLYNR------EKTLSE----ATF 57
175 AEKLSNNELEQGVIETIIDRDDLFAILPFMKINSKAYLYNR------EKTLSE----ATF 57
176 AEKLSNNELEQGVIETIIDRDDLFAVLPFMKIDSKAYLYNR------EATLSE----ATF 57
177 AEKLSNNELEQGVIETIIDRDDLFAILPFMKINSKAYLYNR------EATLSE----ATF 57
178 AEKLSNNELEQGVIETIIDRDDLFAILPFMKINSKAYLYNR------EATLSE----ATF 57
179 AEKLSNNELEQGVIETIIDRDDLFAILPFMKINSKAYLYNR------EKTLSE----ATF 57
180 AEKLSNNELEQGVIETIIDRDDLFAVLPFMKIDSKAYLYNR------EATLSE----ATF 57
181 AEKLSNNELEQGVIETIIDRDDLFAILPFMKINSKAYLYNR------EKTLSE----ATF 57
182 AEKLSNNELEQGVIETIIDRDDLFAILPFMKINSKAYLYNR------EKTLSE----ATF 57
183 AEKLSNNELEQGVIETIIDRDDLFAILPFMKINSKAYLYNR------EKTLSE----ATF 57
184 AEKLSNNELEQGVIETIIDRDDLFAILPFMKINSKAYLYNR------EKTLSE----ATF 57
185 AEKLSNNELEQGVIETIIDRDDLFAILPFMKINSKAYLYNR------EATLSE----ATF 57
186 AEKLSNNELEQGVIETIIDRDDLFAILPFMKINSKAYLYNR------EKTLSE----ATF 57
187 AEKLSNNELEQGVIETIIDRDDLFAILPFMKINSKAYLYNR------EKTLSE----ATF 57
188 AEKLSNNELEQGVIETIIDRDDLFAILPFMKINSKAYLYNR------EKTLSE----ATF 57
189 AEKLSNNELEQGVIETIIDRDDLFAILPFMKINSKAYLYNR------EKTLSE----ATF 57
190 AAKLTQDLLLRGVIEAIVKESAVFDYLPFMAITGSALKYNR------EATLGD----AEF 57
191 AEKLSNNELEQGVIETIIDRDDLFAILPFMKIDSKAYLYNR------EATLSE----ATF 57
192 AEKLSEIDLVRGVIEEIIHKDELFALLPWVRTEGKSYDYVR------ELTISE----GEF 57
193 AEKLSNNELEQGVIETIIDRDDLFAIMPFMKINSKAYLYNR------EKTLSE----ATF 57
194 AEKLSNNELEQGVIETIIDRDDLFAILPFMKINSKAYLYNR------EKTLSE----ATF 57
195 AEKLSNNELEQGVIETIIDRDDLFAILPFMKINSKAYLYNR------EKTLSE----ATF 57
196 AEKLSNNELEQGVIETIIDRDDLFAILPFMKINSKAYLYNR------EKTLSE----ATF 57
197 AEKLSNNELEQGVIETIIDRDDLFAILPFMKINSKAYLYNR------EKTLSE----ATF 57
198 AEKLSNNELEQGVIETIIDRDDLFAILPFMKINSKAYLYNR------EKTLSE----ATF 57
199 AEKLSNNELEQGVIETIIDRDDLFAILPFMKINSKAYLYNR------EKTLSE----ATF 57
200 AEKLSNNELEQGVIETIIDRDDLFAILPFMKINSKAYLYNR------EKTLSE----ATF 57
201 AEKLSNNELEQGVIETIIDRDDLFAILPFMKINSKAYLYNR------EKTLSE----ATF 57
202 AAKLSQEDMERGIIEEIIDRDELFALMPFLHVNDKVYTYNR------EKELSS----ATF 57
203 AEKLSNNELEQGVIETIIDRDDLFAILPFMKINSKAYLYNR------EKTLSE----ATF 57
204 AEKLSNNQLISGVIDQIITRDDLFAVLPFVGVNGKAYVYNR------ENTLPS----ADF 57
205 AEKLSNNELEQGVIETIIDRDDLFAILPFMKITSKAYLYNR------EKTLSE----ATF 57
206 AEKLSNNELEQGVIETIIDRDDLFAILPFMKINSKAYLYNR------EKTLSE----ATF 57
207 AEKLSNNELEQGVIETIIDRDDLFAILPFMKINSKAYLYNR------EKTLSE----ATF 57
208 AEKLSNNELEQGVIETIIDRDDLFAILPFMKINSKAYLYNR------EKTLSE----ATF 57
209 AEKLSNNELEQGVIETIIDRDDLFAILPFMKINSKAYLYNR------EKTLSE----ATF 57
210 AEKLSNNELEQGVIETIIDRDDLFAILPFMKINSKAYLYNR------EKTLSE----ATF 57
211 AEKLSNNELEQGVIETIIDRDDLFAILPFMKITSKAYLYNR------EKTLSE----ATF 57
212 AEKLSNNELEQGVIETIIDRDDLFAILPFMKINSKAYLYNR------EKTLSE----ATF 57
213 AEKLSNNELEQGVIETIIDRDDLFAILPFMKINSKAYLYNR------EKTLSE----ATF 57
214 AEKLSNNELEQGVIETIIDRDDLFAILPFIKINSKAYLYNR------EATLSE----ATF 57
215 AEKLSNNELEQGVIETIIDRDDLFAILPFMKINSKAYLYNR------EKTLSE----ATF 57
216 AEKLSNNELEQGVIETIIDRDDLFAILPFMKINSKAYLYNR------EKTLSE----ATF 57
217 AEKLSNNELEQGVIETIIDRDDLFAILPFMKINSKAYLYNR------EKTLSE----ATF 57
218 AEKLSNNELEQGVIETIIDRDDLFAILPFMKINSKAYLYNR------EKTLSE----ATF 57
219 AEKLSNNELEQGVIETIIDRDDLFAILPFMKINSKAYLYNR------EKTLSE----ATF 57
220 AEKLSNNELEQGVIETIIDRDDLFAILPFMKINSKAYLYNR------EKTLSE----ATF 57
221 AEKLSNNELEQGVIETIIDRDDLFAILPFMKINSKAYLYNR------EKTLSE----ATF 57
222 AEKLSNNELEQGVIETIIDRDDLFAILPFMKINSKAYLYNR------EKTLSE----ATF 57
223 AEKLSNNELEQGVIETIIDRDDLFAILPFMKITSKAYLYNR------EKTLSE----ATF 57
224 AEKLSNNELEQGVIETIIDRDDLFAILPFMKINSKAYLYNR------EKTLSE----ATF 57
225 AEKLSNNELEQGVIETIIDRDDLFAILPFMKINSKAYLYNR------EKTLSE----ATF 57
226 AEKLSNNELEQGVIETIIDRDDLFAILPFMKINSKAYLYNR------EKTLSE----ATF 57
227 AEKLSNNELEQGVIETIIDRDDLFAILPFMKINSKAYLYNR------EKTLSE----ATF 57

228	SEKLSNNQLISGVIDEIIERDDLFSVLPFTRVNGKAYVYNR------ENTLAG----ADW	57
229	AEKLSNNELEQGVIETIIDRDDLFAILPFMKINSKAYLYNR------EKTLSE----ATF	57
230	SEKLSNNQLISGVIDEIIERDDLFSVLPFTRVNGKAYVYNR------ENTLAG----ADW	57
231	AEKLSNNELEQGVIETIIDRDDLFAILPFMKINSKAYLYNR------EKTLSE----ATF	57
232	AEKLSNNELEQGVIETIIDRDDLFAILPFMKIDSKAYLYNR------ESTLSE----ATF	57
233	AEKLSNNELEQGVIETIIDRDDLFAILPFMKINSKAYLYNR------EKTLSE----ATF	57
234	AEKLSNNELEQGVIETIIDRDDLFAILPFMKINSKAYLYNR------EKTLSE----ATF	57
235	AEKLSNNELEQGVIETIIDRDDLFAILPFMKINSKAYLYNR------EKTLSE----ATF	57
236	AEKLSNNELEQGVIETIIDRDDLFAILPFMKINSKAYLYNR------EKTLSE----ATF	57
237	AEKLSNNELEQGVIETIIDRDDLFAILPFMKINSKAYLYNR------EKTLSE----ATF	57
238	AEKLSNNELEQGVIETIIDRDDLFAILPFMKINSKAYLYNR------EKTLSE----ATF	57
239	AAKLTNDLLLRGVIETIVMESPVLQRLPFMEVVGTGLTYNR------EATLPA----AEF	57
240	AEKLSNNELEQGVIETIIDRDDLFAILPFMKINSKAYLYNR------EATLSE----ATF	57
241	AEKLSNNELEQGVIETIIDRDDLFAVLPFMKINSKAYLYNR------EATLSE----ATF	57
242	AEKLSNNELEQGVIETIIDRDDLFAILPFMKITSKAYLYNR------EKTLSE----ATF	57
243	AEKLSNNELEQGVIETIIDRDDLFAILPFMKINSKAYLYNR------EKTLSE----ATF	57
244	AEKLSNNELEQGVIETIIDRDDLFAILPFMKINSKAYLYNR------EKTLSE----ATF	57
245	AEKLSNNELEQGVIETIIDRDDLFAILPFMKITSKAYLYNR------EKTLSE----ATF	57
246	AEKLSNNELEQGVIETIIDRDDLFAILPFMKINSKAYLYNR------EKTLSE----ATF	57
247	AEKLSNNELEQGVIETIIDRDDLFAILPFMKINSKAYLYNR------EKTLSE----ATF	57
248	AEKLSNNELEQGVIETIIDRDDLFAILPFMKINSKAYLYNR------EKTLSE----ATF	57
249	AEKLSNNELEQGVIETIIDRDDLFAILPFMKINSKAYLYNR------EKTLSE----ATF	57
250	AEKLSNNELEQGVIETIIDRDDLFAILPFMKITSKAYLYNR------EKTLSE----ATF	57
251	AEKLSNNELEQGVIETIIDRDDLFAILPFMKITSKAYLYNR------EATLSE----ATF	57
252	AEKLSNNELEQGVIETIIDRDDLFAILPFMKINSKAYLYNR------EKTLSE----ATF	57
253	AEKLSNNELEQGVIETIIDRDDLFAILPFMKINSKAYLYNR------EKTLSE----ATF	57
254	AEKLSNNELEQGVIETIIDRDDLFAILPFMKINSKAYLYNR------EKTLSE----ATF	57
255	AEKLSNNELEQGVIETIIDRDDLFAILPFMKINSKAYLYNR------EKTLSE----STF	57
256	AEKLSNNELEQGVIETIIDRDDLFAILPFMKINSKAYLYNR------EATLSE----ATF	57
257	AEKLSNNELEQGVIETIIDRDDLFAILPFMKINSKAYLYNR------EATLSE----ATF	57
258	AEKLSNNELEQGVIETIIDRDDLFAILPFMKINSKAYLYNR------EKTLSE----ATF	57
259	AEKLSNNELEQGVIETIIDRDDLFAILPFMKINSKAYLYNR------EKTLSE----ATF	57
260	AEKLSNNELEQGVIETIIDRDDLFAILPFMKINSKAYLYNR------EKTLSE----ATF	57
261	AEKLSNNELEQGVIETIIDRDDLFAVLPFMKINSKAYLYNR------EKTLSE----ATF	57
262	AEKLSNNELEQGVIETIIDRDDLFAILPFMKITSKAYLYNR------EKTLSE----ATF	57
263	AEKLSNNELEQGVIETIIDRDDLFAILPFMKIDSKAYLYNR------EATLSE----ATF	57
264	AEKLSNNELEQGVIETIIDRDDLFAVLPFMKINSKAYLYNR------EATLSE----ATF	57
265	AEKLSNNELEQGVIETIIDRDDLFAVLPFMKINSKAYLYNR------EKTLSE----ATF	57
266	AEKLSNNELEQGVIETIIDRDDLFAILPFMKITSKAYLYNR------EKTLSE----ATF	57
267	AEKLSNNELEQGVIETIIDRDDLFAVLPFMKINSKAYLYNR------EKTLSE----ATF	57
268	AEKLSNNELEQGVIETIIDRDDLFAVLPFMKINSKAYLYNR------EKTLSE----ATF	57
269	AEKLSNNELEQGVIETIIDRDDLFAILPFMKINSKAYLYNR------EATLSE----ATF	57
270	AERLSNNTLISGIITEIIDRDDLFSILPFVKVNSKAYVYNR------ENTLAG----ASW	57
271	AEKLSNNELEQGVIETIIDRDDLFAILPFMKITSKAYLYNR------EKTLSE----ATF	57
272	AEKLSNNELEQGVIETIIDRDDLFAILPFMKINSKAYLYNR------EKTLSE----ATF	57
273	AEKLSNNELEQGVIETIIDRDDLFAVLPFMKINSKAYLYNR------EKTLSE----ATF	57
274	AEKLSNNELEQGVIETIIDRDDLFAVLPFMKINSKAYLYNR------EKTLSE----ATF	57
275	AEKLSNNELEQGVIETIIDRDDLFAILPFMKINSKAYLYNR------EKTLSE----ATF	57
276	AEKLSNNELEQGVIETIIDRDDLFAILPFMKINSKAYLYNR------EKTLSE----ATF	57
277	AEKLSNNELEQGVIETIIDRDDLFAILPFMKINSKAYLYNR------EKTLSE----ATF	57
278	AEKLSNNELEQGVIETIIDRDDLFAVLPFMKINSKAYLYNR------EKTLSE----ATF	57
279	AEKLSNNELEQGVIETIIDRDDLFAILPFMKINSKAYLYNR------EKTLSE----ATF	57
280	AEKLSNNELEQGVIETIIDRDDLFAILPFMKINSKAYLYNR------EKTLSE----ATF	57
281	AEKLSNNQLVQGVVEEIIDRDDLFAILPFTQVNGKAYVYNR------EKTLGG----ANW	57
282	AEKLSNNELEQGVIETIIDRDDLFAVLPFMKINSKAYLYNR------EKTLSE----ATF	57
283	AEKLSNNELEQGVIETIIDRDDLFAILPFMKINSKAYLYNR------EKTLSE----ATF	57
284	AEKLSNNELEQGVIETIIDRDDLFAILPFMKINSKAYLYNR------EKTLSE----ATF	57
285	AEKLSNNELEQGVIETIIDRDDLFAILPFMKITSKAYLYNR------EKTLSE----ATF	57
286	AEKLSNNELEQGVIETIIDRDDLFAVLPFMKINSKAYLYNR------EATLSE----ATF	57
287	AEKLSNNELEQGVIETIIDRDDLFAILPFMKINSKAYLYNR------EKTLSE----ATF	57
288	AEKLSNNELEQGVIETIIDRDDLFAVLPFMKINSKAYLYNR------EATLSE----ATF	57
289	AEKLSNNELEQGVIETIIDRDDLFAVLPFMKINSKAYLYNR------EKTLSE----ATF	57
290	AEKLSNNELEQGVIETIIDRDDLFAILPFMKINSKAYLYNR------EKTLSE----ATF	57
291	AEKLSNNELEQGVIETIIDRDDLFAILPFMKITSKVYLYNR------EKTLSE----ATF	57
292	AEKLSNNELEQGVIETIIDRDDLFAILPFMKINSKAYLYNR------EKTLSE----ATF	57
293	AEKLSNNELEQGVIETIIDRDDLFAILPFMKINSKAYLYNR------EKTLSE----ATF	57
294	AEKLSNNELEQGVIETIIDRDDLFAILPFMKINSKAYLYNR------EKTLSE----ATF	57

```
295 AEKLSNNELEQGVIETIIDRDDLFAVLPFMKINSKAYLYNR------EKTLSE----ATF 57
296 AEKLSNNELEQGVIETIIDRDDLFAVLPFMKINSKAYLYNR------EKTLSE----ATF 57
297 AEKLSNNELEQGVIETIIDRDDLFAVLPFMKINSKAYLYNR------EKTLSE----ATF 57
298 AEKLSNNELEQGVIETIIDRDDLFAILPFMKINSKAYLYNR------EKTLSE----ATF 57
299 AEKLSNNELEQGVIETIIDRDDLFAVLPFMKINSKAYLYNR------EKTLSE----ATF 57
300 AEKLSNNELEQGVIETIIDRDDLFAILPFMKINSKAYLYNR------EKTLSE----ATF 51
301 AEKLSNNELEQGVIETIIDRDDLFAVLPFMKINSKAYLYNR------EKTLSE----ATF 57
302 AEKLSNNELEQGVIETIIDRDDLFAVLPFMKINSKAYLYNR------EKTLSE----ATF 57
303 AEKLSNNELEQGVIETIIDRDDLFAVLPFMKINSKAYLYNR------EATLSE----ATF 57
304 AEKLSNNELEQGVIETIIDRDDLFAILPFMKINSKAYLYNR------EKTLSE----ATF 57
305 AEKLSNNELEQGVIETIIDRDDLFAVLPFMKINSKAYLYNR------EKTLSE----ATF 57
306 AEKLSNNELEQGVIETIIDRDDLFAVLPFMKINSKAYLYNR------EKTLSE----ATF 57
307 AEKLSNNELEQGVIETIIDRDDLFAVLPFMKINSKAYLYNR------EATLSE----ATF 57
308 AEKLSNNELEQGVIETIIDRDDLFAVLPFMKINSKAYLYNR------EKTLSE----ATF 57
309 AEKLSNNELEQGVIETIIDRDDLFAVLPFMKINSKAYLYNR------EATLSE----ATF 57
310 AEKLSNNELEQGVIETIIDRDDLFAVLPFMKINSKAYLYNR------EATLSE----ATF 57
311 AEKLSNNELEQGVIETIIDRDDLFAVLPFMKINSKAYLYNR------EATLSE----ATF 57
312 AEKLSNNELEQGVIETIIDRDDLFAILPFMKINSKAYLYNR------EKTLSE----ATF 57
313 AEKLSNNELEQGVIETIIDRDDLFAILPFMKINSKAYLYNR------EKTLSE----ATF 57
314 AEKLSNNELEQGVIETIIDRDDLFAVLPFMKINSKAYLYNR------EATLSE----ATF 57
315 AEKLSNNELEQGVIETIIDRDDLFAILPFMKINSKAYLYNR------EKTLSE----ATF 57
316 AEKLSNNELEQGVIETIIDRDDLFAVLPFMKINSKAYLYNR------EKTLSE----ATF 57
317 AEKLSNNELEQGVIETIIDRDDLFAVLPFMKINSKAYLYNR------EATLSE----ATF 57
318 AEKLSNNELEQGVIETIIDRDDLFAVLPFMKINSKAYLYNR------EKTLSE----ATF 57
319 AEKLSNNELEQGVIETIIDRDDLFAILPFMKINSKAYLYNR------EKTLSE----ATF 57
320 AEKLSNNELEQGVIETIIDRDDLFAVLPFMKINSKAYLYNR------EATLSE----ATF 57
321 AEKLSNNELEQGVIETIIDRDDLFAVLPFMKINSKAYLYNR------EKTLSE----ATF 57
322 AEKLSNNELEQGVIETIIDRDDLFAVLPFMKINSKAYLYNR------EATLSE----ATF 57
323 AEKLSNNELEQGVIETIIDRDDLFAVLPFMKINSKAYLYNR------EATLSE----ATF 57
324 AEKLSNNELEQGVIETIIDRDDLFAILPFMKINSKAYLYNR------EKTLSE----ATF 57
325 AEKLSNNELEQGVIETIIDRDDLFAILPFMKINSKAYLYNR------EKTLSE----ATF 57
326 AEKLSNNELEQGVIETIIDRDDLFAVLPFMKINSKAYLYNR------EKTLSE----ATF 57
327 AEKLSNNELEQGVIETIIDRDDLFAVLPFMKINSKAYLYNR------EKNLSE----ATF 57
328 AEKLSNNELEQGVIETIIDRDDLFAVLPFMKINSKAYLYNR------EKTLSE----ATF 57
329 AEKLSNNELEQGVIETIIDRDDLFAVLPFMKINSKAYLYNR------EATLSE----ATF 57
330 AEKLSNNELEQGVIETIIDRDDLFAVLPFMKINSKAYLYNR------EATLSE----ATF 57
331 AEKLSNNELEQGVIETIIDRDDLFAVLPFMKINSKAYLYNR------EKTLSE----ATF 57
332 AEKLSNNELEQGVIETIIDRDDLFAVLPFMKINSKAYLYNR------EATLSE----ATF 57
333 AEKLSNNELEQGVIETIIDRDDLFAVLPFMKINSKAYLYNR------EATLSE----ATF 57
334 AEKLSNNELEQGVIETIIDRDDLFAILPFMKIDSKAYLYNR------EATLSE----ATF 57
335 AEKLSNNELEQGVIETIIDRDDLFAVLPFMKINSKAYLYNR------EATLSE----ATF 57
336 AEKLSNNELEQGVIETIIDRDDLFAILPFMKINSKAYLYNR------EKTLSE----ATF 57
337 AEKLSNNELEQGVIETIIDRDDLFAVLPFMKINSKAYLYNR------EATLSE----ATF 57
338 AEKLSNNELEQGVIETIIDRDDLFAVLPFMKINSKAYLYNR------EKTLSE----ATF 57
339 AEKLSNNELEQGVIETIIDRDDLFAVLPFMKINSKAYLYNR------EATLSE----ATF 57
340 AEKLSNNELEQGVIETIIDRDDLFAVLPFMKINSKAYLYNR------EKTLSE----ATF 57
341 AEKLSNNELEQGVIETIIDRDDLFAVLPFMKINSKAYLYNR------EATLSE----ATF 57
342 AEKLSNNELEQGVIETIIDRDDLFAILPFMKINSKAYLYNR------EATLSE----ATF 57
343 AEKLSNNELEQGVIETIIDRDDLFAVLPFMKINSKAYLYNR------EATLSE----ATF 57
344 AEKLSNNELEQGVIETIIDRDDLFAVLPFMKINSKAYLYNR------EATLSE----ATF 57
345 AEKLSNNELEQGVIETIIDRDDLFAVLPFMKINSKAYLYNR------EKTLSE----ATF 57
346 AEKLSNNELEQGVIETIIDRDDLFAILPFMKINSKAYLYNR------EKTLSE----ATF 57
347 AEKLSNNELEQGVIETIIDRDDLFAILPFMKINSKAYLYNR------EKTLSE----ATF 57
348 AEKLSNNELEQGVIETIIDRDDLFAILPFMKIDSKAYLYNR------EATLSE----ATF 57
349 AEKLSNNELEQGVIETIIDRDDLFAILPFIKINSKAYLYNR------EATLSE----ATF 57
350 AEKLSNNELEQGVIETIIDRDDLFAVLPFMKINSKAYLYNR------EATLSE----ATF 57
351 ------------VVEEIIDRDDLFAILPFTQVNGKAYVYNR------EKTLGG----ANW 38
352 AEKLSNNELEQGVIETIIDRDDLFAVLPFMKINSKAYLYNR------EATLSE----ATF 57
353 SAKLSQDQLVSGVIENVITVNKFYQHLPFLPIEGNALAYNR------ENALGD----VQF 58
354 AEKLSNNELEQGVIETIIDRDDLFAVLPFMKINSKAYLYNR------EKTLSE----ATF 54
355 AEKLSNNELEQGVIETIIDRDDLFAVLPFMKINSKAYLYNR------EATLSE----ATF 57
356 AEKLSNNELEQGVIETIIDRDDLFAVLPFMKINSKAYLYNR------EATLSE----ATF 57
357 AEKLSNNELEQGVIETIIDRDDLFAVLPFMKINSKAYLYNR------EKTLSE----ATF 57
358 AEKLSNNELEQGVIETIIDRDDLFAILPFMKINSKAYLYNR------EATLSE----ATF 57
359 AEKLSNNELEQGVIETIIDRDDLFAILPFMKINSKAYLYNR------EKTLSE----ATF 57
360 AEKLSNNELEQGVIETIIDRDDLFAVLPFMKINSKAYLYNR------EATLSE----ATF 57
361 AEKLSNNELEQGVIETIIDRDDLFAILPFMKINSKAYLYNR------EATLSE----AEF 57
```

362	AEKLSNNELEQGVIETIIDRDDLFAVLPFMKINSKAYLYNR------EKTLSE----ATF	57
363	AEKLSNNELEQGVIETIIDRDDLFAVLPFMKINSKAYLYNR------EATLSE----ATF	57
364	AEKLSNNELEQGVIETIIDRDDLFAVLPFMKINSKAYLYNR------EATLSE----ATF	57
365	AEKLSNNELEQGVIETIIDRDDLFAVLPFMKINSKAYLYNR------EATLSE----ATF	57
366	AEKLSNNELEQGVIETIIDRDDLFAVLPFMKINSKAYLYNR------EATLSE----ATF	57
367	AEKLSNNELEQGVIETIIDRDDLFAILPFMKIDSKAYLYNR------EATLSE----ATF	57
368	AEKLSNNELEQGVIETIIDRDDLFAILPFMKIDSKAYLYNR------EATLSE----ATF	57
369	AEKLSNNELEQGVIETIIDRDDLFAVLPFMKINSKAYLYNR------EATLSE----ATF	57
370	AEKLSNNELEQGVIETIIDRDDLFAVLPFMKINSKAYLYNR------EATLSE----ATF	57
371	AEKLSNNELEQGVIETIIDRDDLFAVLPFMKINSKAYLYNR------EATLSE----ATF	57
372	AEKLSNNELEQGVIETIIDRDDLFAVLPFMKINSKAYLYNR------EATLSE----ATF	57
373	AEKLSNNELEQGVIETIIDRDDLFAVLPFMKINSKAYLYNR------EATLSE----ATF	57
374	AEKLSNNELEQGVIETIIDRDDLFAILPFMKITSKAYLYNR------EATLSE----ATF	57
375	AEKLSNNELEQGVIETIIDRDDLFAVLPFMKINSKAYLYNR------EATLSE----ATF	57
376	AEKLSNNELEQGVIETIIDRDDLFAILPFMKINSKAYLYNR------EKTLSE----ATF	57
377	AAKLTDDMLLRGVIETIVMESPVLQRLPFMEVVGTGLTYNR------EATLPA----ASF	57
378	AEKLSNNELEQGVIETIIDRDDLFAVLPFMKINSKAYLYNR------EATLSE----ATF	57
379	AEKLSNNELEQGVIETIIDRDDLFAVLPFMKINSKAYLYNR------EATLSE----ATF	57
380	AEKLSNNELEQGVIETIIDRDDLFAILPFMKIDSKAYLYNR------EATLSE----ATF	57
381	AEKLSNNELEQGVIETIIDRDDLFAILPFMKIDSKAYLYNR------EATLSE----ATF	57
382	SAKLAQDTLITGLIESIITVNHMFQLLPFEGINGNALAYNR------ENALGD----VES	58
383	AEKLSNNELEQGVIETIIDRDDLFAILPFMKIDAKAYLYNR------EATLSE----ATF	57
384	AEKLSNNELEQGVIETIIDRDDLFAILPFMKIDAKAYLYNR------EATLSE----ATF	57
385	AEKLSNNELEQGVIETIIDRDDLFAVLPFMKINSKAHLYNR------EATLSE----ATF	57
386	AEKLSNNELEQGVIETIIDRDDLFAILPFMKINSKAYLYNR------EKTLSE----ATF	57
387	ADRLSATSVERGVVEEVIDRDALFALLPFHRIEGKAWVYNR------EGVLSE----GAF	57
388	AEKLSNNELEQGVIETIIDRDDLFAILPFMKIDSKAYLYNR------EATLSE----ATF	57
389	AEKLSNNELEQGVIETIIDRDDLFAVLPFMKINSKAYLYNR------EKTLSE----ATF	57
390	AAKLCQDELVSGLIENIITVNPFYQVFPFDSIEGNGLTYNR------EDVLGD----VQV	58
391	AEKLSNNELEQGVIETIIDRDDLFAILPFMKIDSKAYLYNR------EATLSE----ATF	57
392	AEKLSNNELEQGVIETIIDRDDLFAVLPFMKINSKAYLYNR------EATLSE----ATF	57
393	AEKLSNNELEQGVIETIIDRDDLFAVLPFMKINSKAYLYNR------EATLSE----ATF	57
394	AEKLSNNELEQGVIETIIDRDDLFAIMPFMKIDSKAYLYNR------EATLSE----ATF	57
395	AEKLSNNELEQGVIETIIDRDDLFAILPFMKIDSKAYLYNR------EATLSE----ATF	57
396	AEKLSNNELEQGVIETIIDRDDLFAILPFMKVNNKAYLYNR------EATLSE----ATF	57
397	SAKLAQDELVAGVIENVITVNELYELLPFDGIDGNALAYNR------ENALGD----VQM	58
398	AEKLSNNELEQGVIETIIDRDDLFAILPFMKINSKAYLYNR------EKTLSE----ATF	57
399	AEKLSNNELEQGVIETIIDRDDLFAVLPFMKINSKAYLYNR------EATLSE----ATF	57
400	AEKLSNNELEQGVIETIIDRDDLFAILPFMKIDSKAYLYNR------EATLSE----ATF	57
401	AEKLSNNELEQGVIETIIDRDDLFAILPFMKIDSKAYLYNR------EATLSE----ATF	57
402	AEKLSNNELEQGVIETIIDRDDLFAILPFMKIDSKAYLYNR------EATLSE----ATF	57
403	AEKLSNNELEQGVIETIIDRDDLFAVLPFMKINSKAYLYNR------EKTLSE----ATF	57
404	AEKLSNNELEQGVIETIIDRDDLFAVLPFMKINSKAYLYNR------EKTLSE----ATF	57
405	AEKLSNNELEQGVIETIIDRDDLFAILPFMKINSKAYLYNR------EKTLSE----ATF	57
406	AEKLSNNELEQGVIETIIDRDDLFAILPFMKIDSKAYLYNR------EATLSE----ATF	57
407	AEKLSNNELEQGVIETIIDRDDLFAVLPFMKINSKAYLYNR------EKTLSE----ATF	57
408	AEKLSNNELEQGVIETIIDRDDLFAVLPFMKINSKAYLYNR------EATLSE----ATF	57
409	AEKLSNNELEQGVIETIIDRDDLFAILPFMKIDSKAYLYNR------EATLSE----ATF	57
410	AEKLSNNELEQGVIETIIDRDDLFAILPFMKIDSKAYLYNR------EATLSE----ATF	57
411	AEKLSNNELEQGVIETIIDRDDLFAILPFMKIDSKAYLYNR------EATLSE----ATF	57
412	AEKLSNNELEQGVIETIIDRDDLFAILPFMKIDAKAYLYNR------EATLSE----ATF	57
413	AEKLSNNELEQGVIETIIDRDDLFAILPFMKINSKAYLYNR------EKTLSE----ATF	57
414	AEKLSNNELEQGVIETIIDRDDLFAILPFMKIDSKAYLYNR------EATLSE----ATF	57
415	AEKLSNNELEQGVIETIIDRDDLFAILPFMKIDSKAYLYNR------EATLSE----ATF	57
416	AEKLSNNELEQGVIETIIDRDDLFAILPFMKIDAKAYLYNR------EATLSE----ATF	57
417	AEKLSNNELEQGVIETIIDRDDLFAILPFMKVNNKAYLYNR------EATLSE----ATF	57
418	AEKLSNNELEQGVIETIIDRDDLFAVLPFMKINSKAYLYNR------EATLSE----ATF	57
419	AAKLSIEDKQRGVIEEIIDRDELFALVPFVRAKDKVYSYVR------EGELPT----GDW	57
420	AEKLSNNELEQGVIETIIDRDDLFAILPFMKIDSKAYLYNR------EATLSE----ATF	57
421	AEKLSNNELEQGVIETIIDRDDLFAILPFMKITSKAYLYNR------EKTLSE----ATF	57
422	AEKLSNNELEQGVIETIIDRDDLFAILPFMKINSKAYLYNR------EATLSE----ATF	57
423	AEKLSNNELEQGVIETIIDRDDLFAILPFMKVNNKAYLYNR------EATLSE----ATF	57
424	AEKLSNNELEQGVIETIIDRDDLFAILPFMKIDSKAYLYNR------EATLSE----ATF	57
425	AEKLSNNELEQGVIETIIDRDDLFAILPFMKINSKAYLYNR------EATLSE----ATF	57
426	AEKLSNNELEQGVIETIIDRDDLFAILPFMKINSKAYLYNR------EATLSE----ATF	57
427	AEKLSNNELEQGVIETIIDRDDLFAILPFMKINSKAYLYNR------EATLSE----ATF	57
428	AEKLSNNELEQGVIETIIDRDDLFAILPFMKIDSKAYLYNR------EATLSE----ATF	57

```
429  AEKLSNNELEQGVIETIIDRDDLFAILPFMKITSKAYLYNR------EKTLSE----ATF  57
430  AEKLSNNELEQGVIETIIDRDDLFAILPFMKINSKAYLYNR------EATLSE----ATF  57
431  AEKLSNNELEQGVIETIIDRDDLFAILPFMKIDSKAYLYNR------EATLSE----ATF  57
432  SAKLAQDELVAGVIENVITVNKMYQLLPFDGIDGNSLAYNR------ENVLGD----VQV  58
433  AEKLSNNELEQGVIETIIDRDDLFAILPFMKINSKAYLYNR------EKTLSE----ATF  57
434  AEKLSNNELEQGVIETIIDRDDLFAVLPFMKINSKAYLYNC------EKTLSE----ATF  57
435  AEKLSNNELEQGVIETIIDRDDLFAVLPFMKINSKAYLYNC------EKTLSE----ATF  57
436  AEKLSNNELEQGVIETIIDRDDLFAILPFMKIDSKAYLYNR------EATLSE----ATF  57
437  AERLSNNELEQGVIETIIDRDDLFAIMPFFKINSKAYLYNR------EKTLSE----AEF  57
438  AEKLSNNELEQGVIETIVDRDDLFAILPFMKIDSKAYLYNR------EATLSE----ATF  57
439  AERLSNNTLISGIITEIIDRDDLFSILPFVKVNSKAYVYNR------ENTLAG----ADW  57
440  AEKLSNNELEQGVIETIIDRDDLFAILPFMKINSKAYLYNR------EKTLSE----ATF  57
441  AAKLTDDMLLRGVIETIVMESPVLQRLPFMEVVGTGLTYNR------EATLPA----ATF  57
442  AEKLSNNTLEQGVIETIIDREDLFAVLPFMKVDSKAYLYNR------ESELSE----ASF  57
443  AEKLSNNELEQGVIETIIDRDDLFAILPFFKVNSKAYLYNR------EATLSE----AGF  57
444  AAKLSNNDLVAGIIENIVTVDRFFGVLPFEPVEGPAAVYNR------ENVLGD----VQM  58
445  SAKLCQDVLIQGVIESIVTVDDFYWMLPFEGIDGNALSYNR------ENALGD----VES  58
446  AEKLSNNELEQGVIETIIDRDDLFAILPFMKINSKAYLYNR------EKTLSE----ATF  57
447  AEKLSNNELEQGVIETIIDRDDLFAILPFMKINSKAYLYNR------EKTLSE----ATF  57
448  AAKLTNDLLLRGVIETIVKESSVLLMLPFMEVTGTALTYNR------EATMPQ----AQF  55
449  AEKLSNNELEQGVIETIIDRDDLFAILPFMKINSKAYLYNR------EKTLSE----ATF  57
450  AEKLSNNELEQGVIETIIDRDDLFAILPFMKINSKAYLYNR------EKTLSE----ATF  57
451  AAKLTDDLLLRGVIETIVMESPVLQRLPFMEVVGTGLTYNR------EGTLPA----TDF  57
452  AAKLTNDMLLRGVIETIVMESSVLTMLPFMNVTGTALTYNR------EKTMPA----AKF  57
453  AEKLSNNELEQGVIETIIDRDDLFAILPFMKIDSKAYLYNR------EATLSE----ATF  57
454  SAKLAQDELVAGVIENVITVNELYELLPFDGIDGNALAYNR------ENVLGD----VQM  58
455  AEKLSNNQLVAGVIEEIIERDELFALIPFIGINGKAYVYDR------ENSLPT----ADF  57
456  AEKLSNNELEQGVIETIIDRDDLFAVLPFMKINSKAYLYNR------EKTLSE----ATF  57
457  AEKLSNNELEQGVIETIIDRDDLFAILPFMKINSKAYLYNR------EKTLSE----ATF  57
458  AEKLSNNELEQGVIETIIDRDDLFAILPFMKIDSKAYLYNR------EATLSE----ATF  57
459  AEMLSQNDVVVGIIEEIITVNELFGLLPFVKTEGKAYVYHR------ENTLPS----VSY  57
460  AEKLSNNELEQGVIETIIDRDDLFAILPFMKINSKAYLYNR------EKTLSE----ATF  57
461  SAKLAQDELVAGVIENVITVNEIFEQLPFDGIDGNALAYNR------ENVLGD----VQM  58
462  AEKLSNNELEQGVIETIIDRDDLFAILPFMKINSKAYLYNR------EKTLSE----ATF  57
463  ------------------------------------------------------------
464  AEKLSNNELEQGVIETIIDRDDLFAILPFMKINSKAYLYNR------EATLSE----ATF  57
465  AEKLSNNELEQGVIETIIDRDDLFAILPFMKINSKAYLYNR------EKTLSE----ATF  57
466  AEKLSNNELEQGVIETIIDRDDLFAILPFMKINSKAYLYNR------EKTLSE----ATF  57
467  AEKLSNNELEQGVIETIIDRDDLFAILPFMKINSKAYLYNR------EKTLSE----ATF  57
468  AEKLSNNELEQGVIETIIDRDDLFAILPFMKITSKAYLYNR------EKTLSE----ATF  57
469  AEKLSNNELEQGVIETIIDHEDLFAVLPFHKINSKAYLYNR------EATLSE----AQF  57
470  AEKLSNNELEQGVIETIIDRDDLFAILPFMKINSKAYLYNR------EKTLSE----ATF  57
471  SAKLALDDLVAGVIENVITVNRMYEMIPFDGIAGNALAYNR------ENVLGD----VQV  58
472  AERLSANQMVQGVIEEIIERDDLFSILPFVQTEGKAYLYNR------EKTIGG----ADW  57
473  SAKLALDDLVAGVIENVITVNRMYEMIPFDGIAGNALAYNR------ENVLGD----VQV  58
474  AEKLSNNELEQGVIETIIDRDDLFAILPFMKINSKAYLYNR------EKTLSE----ATF  57
475  AEKLSNNELEQGVIETIIDRDDLFAILPFMKITSKAYLYNR------EATLSE----ATF  57
476  SAKLAQDELVAGVIENVITVNEIFEQLPFDGIDGNALAYNR------ENVLGD----VQM  58
477  SAKLSQNLLYAGIIESIVEVNPFFEILPFQEIEGNALSYNR------EKTLGD----IQF  58
478  AAKLSQDDLVSGIIANIVTVDRFYGLLPFEPVEGPAAVYNR------ENVLGD----VQM  58
479  SAKLAQDELVAGVIENVITVNEMFDLLPFDGIDGNALAYNR------ENVLGD----VQV  58
480  SAKLALDELVAGVIENVITVNRMFEVIPFDGISGNALAYNR------ENVLGN----VQV  58
481  AEKLSNNELEQGVIETIIDRDDLFAILPFMKINSKAYLYNR------EKTLSE----ATF  57
482  AEKLSNNELEQGVIETIIDRDDLFAILPFMKINSKAYLYNR------EKTLSE----ATF  57
483  AEKLSNNTLEQGVIETIIDREDLFAVLPFMKVDSKAYLYNR------ESELSE----AEF  57
484  AEKLSNNELEQGVIETIIDRDDLFAILPFMKINSKAYLYNR------EKTLSE----ATF  57
485  AAKLTNDMLLRGVIETIVMESSVLTLLPFMNVTGTALTYNR------EKTMPA----AQF  57
486  AEKLSNNTLEQGVIETIIDREDLFAVLPFMKVDSKAYLYNR------ESELSE----AAF  57
487  AEKLSNNELEQGVIETIIDRDDLFAILPFMKIDSKAYLYNR------EATLSE----ATF  57
488  AEKLSNNTLEQGVIETIIDREDLFAVLPFMKVDSKAYLYNR------ESELSE----AQF  57
489  AEKLSNNTLEQGVIETIIDREDLFAVLPFMKVDSKAYLYNR------ESELSE----ATF  57
490  SAKLAQDELVAGVIENVITVNEIFEQLPFDGIDGNALAYNR------ENVLGD----VQM  58
491  AEKLSNNELEQGVIETIIDRDDLFAILPFMKINSKAYLYNR------EKTLSE----ATF  57
492  AAKLSNNDLVAGIVENIVTVDRFFGVLPFEPVEGPAAVYNR------ENVLGD----VQM  58
493  AEKLSNNELEQGVIETIIDRDDLFAILPFMKINSKAYLYNR------EKTLSE----ATF  57
494  AAKLTNDLLLRGVVETIVRESSVLALLPFTEVTGTALTYVR------EATLPA----ASW  57
495  AEKLSNNELEQGVIETIIDRDDLFAVLPFMKINSKAYLYNR------EKTLSE----ATF  57
```

```
496  AEKLSNNELEQGVIETIIDRDDLFAVLPFMKINSKAYLYNR------EKTLSE----ATF  57
497  SAKLSQDLLLSGVIESIVDVNPIYEVFPFMGIEGNSLAYNR------ENVLGD----VQF  58
498  AEKLSNNELEQGVIETIIDRDDLFAILPFMKINSKAYLYNR------EKTLSE----ATF  57
499  AEKLSNNTLEQGVIETIIDREDLFAVLPFMKVDSKAYLYNR------ESELSE----AQF  57
500  SAKLAQDELVAGVIENVITVNEIFEQLPFDGIDGNALAYNR------ENVLGD----VQM  58
```

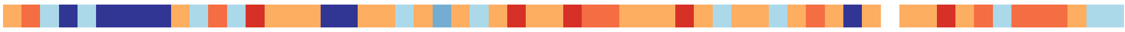

```
          130       140       150       160       170       180
1    IGFG-----DPIPDNTTIPDEPI-TFPMSELATSFLVAYKGQDIY-S-YTNDLVKLEEHL  108
2    IGFG-----DPIPDNTTIPDEPI-TFPMSELATSFLVAYKGQDIY-S-YTNDLVKLEEHL  108
3    IGFG-----DPIPDNTTIPDEPI-TFPMSELATSFLVAYKGQDIY-S-YTNDLVKLEEHL  108
4    IGFG-----DPIPDNTTIPDEPI-TFPMSELATSFLVAYKGQDIY-S-YTNDLVKLEEHL  108
5    IGFG-----DPIPDNTTIPDEPI-TFPMSELATSFLVAYKGQDIY-S-YTNDLVKLEEHL  108
6    IGFG-----DPIPDNTTIPDEPI-TFPMSELATSFLVAYKGQDIY-S-YTNDLVKLEEHL  108
7    IGFG-----DPIPDNTTIPDEPI-TFPVSELATSFLVAYKGQDIY-S-YTNDLVKLEDHL  108
8    IGFG-----DPIPDNTTIPDEPI-TFPMSELATSFLVAYKGQDIY-S-YTNDLVKLEEHL  108
9    IAFG-----DPIPDNTTIPDEPI-TFPVSELATSFLVAYKGQDIY-S-YTNDLVKLEDHL  108
10   IGFG-----DPIPDNTTIPDEPI-TFPMSELATSFLVAYKGQDIY-S-YTNDLVKLEEHL  108
11   IGFG-----DPIPDNTTIPDEPI-TFPMSELATSFLVAYKGQDIY-S-YTNDLVKLEEHL  108
12   IGFG-----DPISDNTTIPDEPI-TFSMSELATSFLVAYKGQDIY-S-YTNDLVKLEEHL  108
13   IGFG-----VPIPDNTTIPDEPI-TFPMSELATSFLVAYKVQDIY-S-YTNDLVKLEEHL  108
14   IGFG-----DAIEDQTSLPEEPV-IFPMTEFATQFVVSYTGQDVY-S-DTNNLLQVEHDS  108
15   IGQC-----GTIAEDTKQPNDTNRIYRFAEIATQFRVCYKAQDIFSS-NVNDQVAVQMAL  111
16   IGQN-----SPIPEDTKQPNDPNRVYRFAEIATHFRVSYKAQDIFSS-NANDQVAVQMAL  111
17   IGQN-----SPIPEDTKQPNDPNRVYRFAEIATHFRVSYKAQDIFSS-NANDQVAVQMAL  111
18   LGQC-----DTISEDTKQPNDLNRVHRFAEIATHFRVSYKAQDIFSS-NVNDQVAVQMAL  111
19   IGFE-----EGILEDTKMPNDQKRAFAFAEIATQFRVSYKAQDVFSS-NVNDQVSVQMAL  111
20   INQC-----DSIAADTKQPHDTNRIYRFAEIATQFRVCYKAQDIFSS-NVNDQAAVQMAL  112
21   IGFE-----EGIVEDTKMPNDASRVFSFAEIATQFRVSYKAQDIFSS-NVNDQVSVQMAL  111
22   IGFE-----EGIVEDTKMPNDPSRVFTFAEIATQFRVAYKAQDIFSS-NVNDQVSVQMAL  111
23   IGFE-----EGIVEDTKMPNDASRVFSFAEIATQFRVSYKAQDIFSS-NVNDQVSVQMAL  112
24   IGFE-----EGIVEDTKMPSDASRVFSFAEIATQFRVSYKAQDIFSS-NVNDQVSVQMAL  111
25   IGFE-----EGIMEDTKMPNDQKRNFTFAEIATHFRVSYKAQDVFSS-NVNDQVSVQMAL  111
26   IGFE-----EGIVEDTKMPNDASRVFSFAEIATQFRVSYKAQDIFSS-NVNDQVSVQMAL  111
27   IGQN-----DTIPEDTKQPADPNRVHRFAEIATHFRVSYKAQDIFSS-NVNDQVAVQMAL  111
28   IGFE-----EGIVEDTKMPSDASRVFSFAEIATQFRVSYKAQDIFSS-NVNDQVSVQMAL  111
29   ANLNS-EC-GPVTDRTSQPTDPEVVFGLANIITRYSVCFDDQDKF-S-NPNNLDRTEYVL  112
30   ANLNS-EC-GPVTDRTSQPTDPEVVFGLANIITRYSVCFDDQDKF-S-NPNNLDRTEYVL  118
31   ADFNS-SC-GQVTLHTDQP-DSTVTFGLANLITRYQVCFDDQDKF-E-IPNNLDETEYVL  114
32   ADFNS-SC-GQVTLHTDQP-DSTVTYGLANLITRYQVCFDDQDKF-E-IPNNLDETEYVL  114
33   ADFNS-SC-GQVTLHTDQP-DSTVTFGLANLITRYQVCFDDQDKF-E-IPNNLDETEYVL  114
34   ATILGDGATSGITVQTADPDNPNTTFTLGELATRYKIDYSSMDRF-K-VPNNLDAVESAL  119
35   ATILGDGTD--ITDKTANADTS-KTFTVGELATRYKVDYNAQDRY-R-F-QSIDAVEAVL  130
36   ---------------------------MSELATSFLVAYKGQDIY-S-YTNDLVKLEEHL  31
37   ATILGEGGTSGIAVQTADPAASNATFTLGELATRYKIEYSSMDRF-K-YPNNLDAVESAL  119
38   ADLSTGTCDSEVTERTDSP-DSEAVFELVSLMTRYQICMADLDRV-R-YPEQLKQIEFEL  112
39   NGFG-----TVIPDQTSMPDPQI-LFVLKQLATSYQVAPEAQDVD-S-AINNQKEIQDDL  108
40   ADILGDGDTA-VTEQANDPDASNTTFTIGALATRYFLDYASTDRF-K-YPNDFDAVESAL  118
41   IGYG-----SPLLDQTTIPNVPI-TYPFIDLATSFRISVKALDTC-S-GTNNLVLIQIKL  108
42   AEISDGGCTTEVPERTDQP-DSEAIFELVTIMTRFQICMADIDRI-R-DPELLKQVLGVL  112
43   ASVLT-GCSTEVTEHTDQP-DSEVVFELVTLMTRYQICMADLDRI-Q-DPELLKQVYWEL  111
44   AEISAGGCDTAVPERTDQP-DSEAIFELVTIITRFQICMADIDRI-R-DPELLKQVLGVL  112
45   ADLSTGSCDSEVSDRTDSP-SSEAIFELVSLMTRYQICMADLDRV-R-YPEQLKQIEFEL  112
46   MA----DC-ATVLEQLPDI--SQAEMTFLDYVTRWQACASDLDRY-R-NPQLLQAIMFAA  106
47   IT----AC-ANVPEQEAAV--SQAGFEFVDFATRYAICASDLDRF-R-HPQLLQSVFYAI  106
48   MA----DC-TTVEEQLPDI--SQAEMTFLDYVTRWQACASDLDRY-R-NPQLLQAIMFAA  106
49   MA----DC-TTVEEQLPDI--SQAEMTFLDYVTRWQACASDLDRY-R-NPQLLQAIMFAA  106
50   MA----DC-ATVLEQLPDI--SQAEMTFLDYVTRWQACASDLDRY-R-NPQLLQDIMFAA  106
51   KS----DC-TEVTEALPTV--SQATFSLLDFIARYAICGVDLDRV-Q-FPTQLDAARYAI  106
52   AG----AC-LPIKDHSDEA--TQAKFGFLEYATRLVICNADEDTL-R-VPNPPEEVEVEL  106
53   -DTGG----TAISDTSAVPSDPDVTFPLKLLVTSFKVNATAEYDM-S-NVNDQVEVQMVA  112
54   AQILGDNTPA-ITDKTADADTS-VTFTVGELATSYLLDYTAQDRF-R-Y-QSIDAVESVL  102
55   -DAGG----TAVGDTPAVPTSPDVTYALKLLISAVKSNYTAQYTM-S-NVNDQEQVQISA  112
56   ANLNS-EC-GPVTDRTSQPTDPEVVFGLANIITRYSVCFDDQDKF-S-SPNNLDHTEYVL  112
```

```
57   NT----GC-SNVAETADTL--ASITFPVVDFVVRKQICFTDADRY-H-LPNNPDEDEYLV  107
58   ATILADGAD--ITDQTAGVDET-VTFTCGELASRYKIDYNAQDRW-R-Y-QSIDNALAII  114
59   -DEGG----SAISDTSAVPSSPNVTFPLKLVATSFKVNDTAEALM-S-SVNDQVETQSVA  112
60   VD----PC-QSADDISTSV--TPARFGMVEYVARVPVCMADQDIF-Q-HPNDITDFNITL  106
61   -DAGG----TAVSDTPAVPTSPNATFSLKLLISAAKTNYTAQYDM-S-NVNDQEGVQINA  112
62   MT----AC-GTVDEQLPDL--SQAEMVFVDYATRYTICASDLDRY-R-NPLLLQAILFAV  106
63   AG----AC-LPIKDHSDEA--TQVQFGMVEYATRVVICNADEDTQ-R-GPNTPELNETVL  106
64   -DAGG----TALSDTPAVPNDPNVSFSLKLLATSFKVNYTAEYDM-S-NVNDQVEVQTVA  112
65   -DAGG----TAVSDTPAVPTSPNPTFSLKLLISMVKTNQTAQYTM-S-NVNNQEKVALAA  112
66   RN----DC-GEVIEQLPTV--SQAAFNLYDFIAHYSICSIDLDRV-Q-YPTQLDAAMFAI  106
67   VP----PC-DAVPDRSSVF--SQATFGMTYLNTRVRVCHANQDTF-R-TPITPEAAEYPL  106
68   LPF----C-APIADQTT--VPVARSYSFGELATSRIVCYGTQDLQ-S-NVNDQSAVQLEM  108
69   LPF----C-GPIADQTT--VPAARSYSFGEVATSRVVCYGTQDLQ-S-NVNDQAAVQIEM  124
70   LGQ----CKDYTDQSFD--SWQERVFDFGAPATMYQSCESAIDKYTS-SINDVIDAQYAK  114
71   LGQ----CEDYTDQSFD--SWQERVFSFGAPATMYQSCESAIDKYTS-SINDVIDVQYAK  165
72   LGQ----CEDYTDQSFD--SWQERVFSFGAPATMYQSCESAIDKYTS-SINDVIDVQYAK  160
73   LGQ----CEDYLDQSFD--SWQERVFDFGAPATLYQSCESAIDKYTS-SINDVIDVQFAK  114
74   IDV-N----DDVPEGAA--TFEEVVCRLARLAGDVDVDKFLDETM-S-DINDQKATQIAS  108
75   FT----SC-AGVPEQEATV--SQAEFQFVDFATRYAICASDLDRF-R-NPQLLQTVLYAI  106
76   -----------VTDRTSQPTDPEVVFGLANIITRYSVCFDDQDKF-S-NPNNLDRTEYVL  47
77   VA----PC-EPADDISTQV--TPARFGMVQYVARVPVCMADQAIY-R-HPTDIVDGNVAL  106
78   LDP-Y----EDVPEGAA--TFDPVSTRLRILAGQVLLDKFLIKTQ-S-DVNDQLAEQISA  108
79   ANFNS-EC-GPVTDRTSQPANPEVVFGLANIITRYSVCFDDQDKF-S-NPNNLDHAEYVL  112
80   LDP-N----EVVPEEAS--TFEEVVARLRILIGDVDVDKFLNTTM-D-DTNNQKAIQIAQ  108
81   LDPVN----DTVNEEAS--TFTEVTANLRVLAGDVDVDKFLDATM-G-DHNSQKAIQIAQ  109
82   LDP-N----EAIPESGS--DFDEVTAKLKILAGDVDVDKFLMETM-S-DVNSQLGLQLAA  108
83   LDA-Y----EAVPEGAA--TFDEVTTKLKVLAGDVDIDKFTAAVQ-S-NLNPQVAIQLAA  108
84   LDA-Y----EAVPEGAA--TFDEVTTKLKVLAGDVDIDKFTAAVQ-S-NLNPQIAIQLAA  108
85   LDA-Y----EAVPEGAA--TFDEVTTKLKVLAGDVDIDKFTAAVQ-S-NLNPQIAIQLAA  108
86   LDA-Y----EAVPEGAA--TFDEVTTKLKVLAGDVDIDKFTAAVQ-S-NLNPQIAIQLAA  108
87   LGA----CLDYTLQDYE--I-EQATYVYGELVSAFQTCSTTQD-ITG-GPNNLNNALRKK  106
88   AQVLGDNTPA-IADKTADADTS-VTFTVGELATSYLLDYTAKDRFSS-Y-QSIDNVESVL  57
89   LDA-Y----EAVPEGAA--TFDEVTTKLKVLAGDVDIDKFTAAVQ-S-NLNPQVAIQLAA  108
90   LDP-Y----EVVPEGAA--TFKEVMTKLRILAGDVDLDKFLITTQ-S-DTNDQLAIQIAS  108
91   VS----DC-SLVDEQVPTI--SEAEFALQEFITRYKICTFDLDSY-Q-FPNQLETVLYAL  65
92   LGA----CLDYTLQDYE--I-EQATYIYGELVSAFQTCSTTQD-ITG-GPNNLNNALRKK  106
93   LGA----CLDYTLQDYE--I-EQATYIYGELVSAFQTCSTTQD-ITG-GPNNLNNALRKK  106
94   LSP-Y----DPVPEGAA--TFDEVSTKLRILAGDVDIDKFTAETQ-S-DLNDQIAIQLQG  108
95   LDA-Y----EAVPEGAA--TFDEVTAKLKVLAGDVDIDKFTAAVQ-S-HLNPQIAIQLAA  108
96   LDP-N----DVVPEEAS--TFQEVVAKLRILAGDVDIDKFLQATM-S-DTNSQIVTQLAA  108
97   LDP-Y----EDIKEGAA--TFEEVQAKLRILAGHVDLDNFLVETQ-S-DTHDQLAIQLAA  108
98   LDV-N----EVVPEGTS--DVEEVVTKLRILAGDVDVDKFIDETM-S-DKNSQLALQIAM  108
99   LDP-Y----EDIPEGAA--TFDEVQAKLRILAGHVDLDNFILETQ-S-ETNPQLAIQLAA  108
100  LDV-N----EIVPEGAS--DVEEVVTKLRIIAGDVDVDNFLNETM-S-DTNSQLALQIAL  108
101  IDPVT----DTVPEGAA--TFESVTETLKVLIGDVDVDNFLRETM-S-DTNNQFETQIAA  109
102  AQPL--DYNAAIADSTMAP-SANVTFTPGVVASRYRIAYSALGRF-D-ANQDLDAVEAAL  112
103  LDV-N----EVVPEGTS--DVEEVVTKLRILAGDVDVDKFIDETM-S-DKNSQLALQIAM  108
104  LDP-N----EEIKESAS--TFEEVTAKLRILAGDVDIDKFLDSTM-D-DTNNQMAIQVTQ  108
105  LDP-N----EVIEEEGS--TFDEVTARLRILAGDVDVDKFLDSTM-G-DTNAQKAIQIKQ  108
106  LDP-N----EVIEEEGS--TFDEVTAHLRILAGDVDVDKFLDSTM-G-DTNAQKAIQIKQ  106
107  LDP-N----DPITESAA--TFTEVVAKLRILVGDVDVDKFLQATM-G-DTNDQMAIQIAK  108
108  LDP-N----EVIEEEGS--TFDEVTAHLRILAGDVDVDKFLDSTM-G-DTNAQKAIQIKQ  108
109  LDP-N----EVIEEEGS--TFDEVTAHLRILAGDVDVDKFLDSTM-G-DTNAQKAIQIKQ  108
110  LDP-N----EVIEEEGS--TFDEVTAHLRILAGDVDVDKFLDSTM-G-DTNAQKAIQIKQ  108
111  LDP-N----EEVPESAA--TFTPITVTLKIIAGDVDVDKFIQATE-S-DTNDQKAVQIAA  108
112  LDP-N----EVIEEEGS--TFDEVTAHLRILAGDVDVDKFLDSTM-G-DTNAQKAIQIKQ  110
113  LDP-N----EVIEEEGS--TFDEVTAHLRILAGDVDVDKFLDSTM-G-DTNAQKAIQIKQ  108
114  LDP-N----EVIEEEGS--TFDEVTAHLRILAGDVDVDKFLDSTM-G-DTNAQKAIQIKQ  108
115  LDV-N----EVVPEGAS--EVEEVVTKLRIIAGDVDVDKFLNETM-S-DTNQQLAIQIAM  108
116  LDP-N----DEVPESAS--KFVEVTTKLRILAGDVDVDKFLASTM-G-DTNPQKALQIAA  108
117  LDP-N----EEIKESAS--TFEEVTAKLRILAGDVDIDKFLGSTM-D-DTNNQMAIQVTQ  108
118  LDP-N----EEIKESAS--TFEEVTAKLRILAGDVDIDKFLDSTM-D-DTNNQMAIQVTQ  108
119  LDP-N----DEVPESAS--KFVEVTTKLRILAGDVDVDKFLASTM-G-DTNPQKALQIAA  108
120  LDV-N----EVVPENAS--TFKEVTTKLRILIGDVDVDKFMAETM-S-DTEDQLGVQIAL  108
121  LDP-N----EVIEEEGS--TFDEVTAHLRILAGDVDVDKFLDSTM-G-DTNAQKAIQIKQ  108
122  LDP-N----EVIEEEGS--TFDEVTAHLRILAGDVDVDKFLDSTM-G-DTNAQKAIQIKQ  108
123  LDP-N----DEITEGAS--TFTEVVAKLRILAGDVDIDKFLETTM-G-DTNSQMKIQISK  108
```

```
124  LDP-N----EVIEEEGS--TFDEVTAHLRILAGDVDVDKFLDSTM-G-DTNAQKAIQIKQ  108
125  LDP-N----EVIEEEGS--TFDEVTAHLRILAGDVDVDKFLDSTM-G-DTNAQKAIQIKQ  108
126  LDP-N----EVIEEEGS--TFDEVTAHLRILAGDVDVDKFLDSTM-G-DTNAQKAIQIKQ  108
127  LDP-Y----EDITEGAA--TFEEVQAKLRILAGHVDLDNFLVETQ-S-DTQDQLAVQLAA  108
128  LDP-Y----EDITEGAA--TFEEVQAKLRILAGHVDLDNFLVETQ-S-DTQDQLAVQLAA  108
129  LSP-N----DTVNEDAG--TFDEVVSKLRILAGDVDVDKFLQETD-S-DTNDQRATQIAM  108
130  LSP-N----DTVNEDAG--TFDEVVSKLRILAGDVDVDKFLQETD-S-DTNDQRATQIAM  108
131  LSP-N----DTVNEDAG--TFDEVVSKLRILAGDVDVDKFLQETD-S-DTNDQRATQIAM  108
132  LDP-N----EVIEEEGS--TFDEVTAHLRILAGDVDVDKFLDSTM-G-DTNAQKAIQIKQ  108
133  AGVGD----TITAKNAT--TFTSVTSVLTSILGDAEVNGLVQATQ-S-GETNQTAQQIAS  110
134  LSP-N----DTVNEDAG--TFDEVVSKLRILAGDVDVDKFLQETD-S-DTNDQRATQIAM  108
135  LDP-N----EVIEEEGS--TFDEVTAHLRILAGDVDVDKFLDSTM-G-DTNAQKAIQIKQ  108
136  LDP-N----DEIHESAS--KFTEVTTNLRILAGDVDVDKFLMATM-G-DTNDQLALQLAQ  108
137  LDP-Y----DTVPEGGA--EFDEVVAKLRILIGDVDIDNFLDETM-S-DTNDQTATQIAA  108
138  LDP-N----DPVNESAA--TFTEVVAKLRILAGDVDVDKFLQSTM-G-DTNDQMAIQIAK  108
139  LDP-Y----DPVPEGAA--TFTEVTTRLRILAGDVDMDKFLLSTQ-S-DKNPQLAIQIAA  108
140  LDP-N----DAVPEGAA--KFSEHVAKLRIIAGDVDVDKFIQTTM-S-DTSDQLATQVRL  108
141  LDP-N----EEVPEGAA--KFTEVTTALRVLAHDVDIDKFLASSE-S-DVNDQVALQIAA  108
142  VDP-Y----EVLEESAS--TFTSHSTKIRRFAGQVDIDNFMDEVQ-S-NLNPQTAIQLAS  108
143  LDP-N----DAVPEGAA--KFSEHVAKLRIIAGDVDVDKFIQTTM-S-DTSDQLATQVRL  108
144  MDP-Y----DDFEESAA--TFTEVSTKLKILGGDILLDKFTTATQ-S-ALNDQVGIQLQK  108
145  LDP-Y----DVVPEGAA--TFEEVVTRLRILAGDVDLDKFLMSTM-S-DANPQLAIQLAA  108
146  LDP-Y----DVVPEGGA--DFDEVVAKLRILIGDVDIDNFLDETM-S-DTNDQTAIQIAA  108
147  IDV-N----DTITEGAA--TFTEHVAKLRILAGDVDVDKFLATTM-S-DTNNQLAIQVRQ  108
148  IDV-N----DTITEGAA--TFTEHVAKLRILAGDVDVDKFLATTM-S-DTNNQLAIQVRQ  108
149  IDV-N----DTITEGAA--TFTEHVAKLRILAGDVDVDKFLATTM-S-DTNNQLAIQVRQ  108
150  IDV-N----DTITEGAA--TFTEHVAKLRILAGDVDVDKFLATTM-S-DTNNQLAIQVRQ  108
151  IDV-N----DTITEGAA--TFSEHVAKLRILAGDVDVDKFLATTM-S-DTNNQLAVQVRQ  108
152  IDV-N----DTITEGAA--TFTEHVAKLRILAGDVDVDKFLATTM-S-DTNNQLAIQVRQ  108
153  MDP-Y----DDFEESAA--TFQEVSTKLKILGGDILLDKFTTATQ-S-ALNDQVGIQLQK  108
154  IDV-N----DTITEGAA--TFTEHVAKLRILAGDVDVDKFLATTM-S-DTNNQLAVQVRQ  108
155  IDV-N----DTIAEGAA--TFSEHVAKLRILAGDVDVDKFLATTM-S-DTNNQLAVQVRQ  108
156  IDV-N----DTITEGAA--TFTEHVAKLRILAGDVDVDKFLATTM-S-DTNNQLAIQVRQ  108
157  IDV-N----DTITEGAA--TFTEHVAKLRILAGDVDVDKFLATTM-S-DTNNQLAIQVRQ  108
158  IDV-N----DTIAEGAA--TFSEHVAKLRILAGDVDVDKFLATTM-S-DTNNQLAVQVRQ  108
159  IDV-N----DTIAEGAA--TFSEHVAKLRILAGDVDVDKFLATTM-S-DTNNQLAVQVRQ  108
160  IDV-N----DTIAEGAA--TFSEHVAKLRILAGDVDVDKFLATTM-S-DTNNQLAVQVRQ  108
161  LDP-N----EEIKESAS--TFEEVTAKLRILAGDVDIDKFLDSTM-D-DTNNQMAIQVTQ  108
162  LDP-S----ETVNEGAA--TFEEIVTRLRILIGDVDVDKFMQGTL-S-DHNNQKAIQIAQ  108
163  IDV-N----DTIAEGAA--TFSEHVAKLRILAGDVDVDKFLATTM-S-DTNNQLAVQVRQ  108
164  IDV-N----DTITEGAA--TFTEHVAKLRILAGDVDVDKFLATTM-S-DTNNQLAIQVRQ  108
165  IDV-N----DTITEGAA--TFTEHVAKLRILAGDVDVDKFLTTTM-S-DTNNQLAIQVRQ  108
166  IDV-N----DTITEGAA--TFTEHVAKLRILAGDVDVDKFLATTM-S-DTNNQLAIQVRQ  108
167  IDV-N----DTITEGAA--TFTEHVAKLRILAGDVDVDKFLATTM-S-DTNNQLAIQVRQ  108
168  IDV-N----DTITEGAA--TFTEHVAKLRILAGDVDVDKFLATTM-S-DTNNQLAIQVRQ  108
169  IDV-N----DTITEGAA--TFTEHVAKLRILAGDVDVDKFLATTM-S-DTNNQLAIQVRQ  108
170  IDV-N----DTIPEGAA--TFEECVAKLRILAGDVDVDKFLATTM-A-DTNNQLAIQVRQ  108
171  IDV-N----DTITEGAA--TFTEHVAKLRILAGDVDVDKFLATTM-S-DTNNQLAIQVRQ  108
172  IDV-N----DTIAEGAA--TFSEHVAKLRILAGDVDVDKFLATTM-S-DTNNQLAVQVRQ  108
173  IDV-N----DTIPEGAA--TFEECVAKLRILAGDVDVDKFLATTM-A-DTNNQLAIQVRQ  108
174  IDV-N----DTIPEGAA--TFEEKVAKLRILAGDVDVDKFLATTM-A-DTNNQLAIQVRQ  108
175  IDV-N----DTIPEGAA--TFEEKVAKLRILAGDVDVDKFLATTM-A-DTNNQLAIQVRQ  108
176  IDV-N----DTIAEGAA--TFTEHVAKLRILAGDVDVDKFLATTM-S-DTNNQLAIQVRQ  108
177  IDV-N----DTIAEGAA--TFSEHVAKLRILAGDVDVDKFLATTM-S-DTNNQLAVQVRQ  108
178  IDV-N----DTIAEGAA--TFSEHVAKLRILAGDVDVDKFLATTM-S-DTNNQLAVQVRQ  108
179  IDV-N----DTIPEGAA--TFEECVAKLRILAGDVDVDKFLATTM-A-DTNNQLAIQVRQ  108
180  IDV-N----DTITEGAA--TFTEHVAKLRILAGDVDVDKFLATTM-S-DTNNQLAIQVRQ  108
181  IDV-N----DTIPEGAA--TFEEKVAKLRILAGDVDVDKFLATTM-A-DTNNQLAIQVRQ  108
182  IDV-N----DTIPEGAA--TFEECVAKLRILAGDVDVDKFLATTM-A-DTNNQLAIQVRQ  108
183  IDV-N----DTIPEGAA--TFEEKVAKLRILAGDVDVDKFLATTM-A-DTNNQLAIQVRQ  108
184  IDV-N----DTIPEGAA--TFEECVAKLRILAGDVDVDKFLATTM-A-DTNNQLAIQVRQ  108
185  IDI-N----DTIAEGAA--TFSEHVAKLRILAGDVDVDKFLATTM-S-DTNNQLAVQVRQ  108
186  IDV-N----DTIPEGAA--TFEEKVAKLRILAGDVDVDKFLATTM-A-DTNNQLAIQVRQ  108
187  IDV-N----DTIPEGAA--TFEECVAKLRILAGDVDVDKFLATTM-A-DTNNQLAIQVRQ  108
188  IDV-N----DTIPEGAA--TFEECVAKLRILAGDVDVDKFLATTM-A-DTNNQLAIQVRQ  108
189  IDV-N----DTIPEGAA--TFEECVAKLRILAGDVDVDKFLATTM-A-DTNNQLAIQVRQ  108
190  HAP-N----AEWGESAP--TFEQVTATLAILGDNADIDAFLQATY-A-SHNDLTAAVIAA  108
```

```
191  IDV-N----DTIAEGAA--TFSEHVAKLRILAGDVDVDKFLATTM-A-DTNNQLAIQVRQ  108
192  LSA-Y----EDVPEGAA--TFDEVSTKCKVLAGQILLDNFTTESM-S-DLNPQLGIQIRS  108
193  IDV-N----DTIPEGAA--TFEECVAKLRILAGDVDVDKFLATTM-A-DTNNQLAIQVRQ  108
194  IDV-N----DTIPEGAA--TFEECVAKLRILAGDVDVDKFLATTM-A-DTNNQLAIQVRQ  108
195  IDV-N----DTITEGAA--TFEEKVAKLRILAGDVDVDKFLATTM-A-DTNNQLAIQVRQ  108
196  IDV-N----DTIPEGAA--TFEECVAKLRILAGDVDVDKFLATTM-A-DTNNQLAIQVRQ  108
197  IDV-N----DTIPEGAA--TFEECVAKLRILAGDVDVDKFLATTM-A-DTNNQLAIQVRQ  108
198  IDV-N----DTIPEGAA--TFEECVAKLRILAGDVDVDKFLATTM-A-DTNNQLAIQVRQ  108
199  IDV-N----DTIPEGAA--TFEECVAKLRILAGDVDVDKFLSTTM-A-DTNNQLAIQVRQ  108
200  IDV-N----DTIPEGAA--TFEEKVAKLRILAGDVDVDKFLATTM-A-DTNNQLAIQVRQ  108
201  IDV-N----DTIPEGAA--TFEECVAKLRILAGDVDVDKFLATTM-A-DTNNQLAIQVRQ  108
202  LDP-Y----EDFTEGAA--TFDPVSTRLRVLGGDILMDKFTTTVQ-S-GINDQIGIQLQL  108
203  IDV-N----DTIPEGAA--TFEEKVAKLRILAGDVDVDKFLATTM-A-DTNNQLAIQVRQ  108
204  LDP-N----DTVNESAA--TFTEVVTKLRILAGDVDIDKFLQTTM-G-DTNDQMAIQIAK  108
205  IDV-N----DTIPEGAA--TFEECVAKLRILAGDVDVDKFLATTM-A-DTNNQLAIQVRQ  108
206  IDV-N----DTIPEGAA--TFEECVAKLCILAGDVDVDKFLATTM-A-DTNNQLAIQVRQ  108
207  IDV-N----DTIPEGAA--TFEECVAKLRILAGDVDVDKFLATTM-A-DTNNQLAIQVRQ  108
208  IDV-N----DTIPEGAA--TFEEKVAKLRILAGDVDVDKFLATTM-A-DTNNQLAIQVRQ  108
209  IDV-N----DTIPEGAA--TFEECVAKLRILAGDVDVDKFLATTM-A-DTNNQLAIQVRQ  108
210  IDV-N----DTITEGAA--TFEEKVAKLRILAGDVDVDKFLATTM-A-DTNNQLAIQVRQ  108
211  IDV-N----DTIPEGAA--TFEECVAKLRILAGDVDVDKFLATTM-A-DTNNQLAIQVRQ  108
212  IDV-N----DTIPEGAA--TFEECVAKLRILAGDVDVDKFLATTM-A-DTNNQLAIQVRQ  108
213  IDV-N----DTIPEGAA--TFEECVAKLRILAGDVDVDKFLATTM-A-DTNNQLAIQVRQ  108
214  IDV-N----DTIAEGAA--TFSEHVAKLRILAGDVDVDKFLATTM-S-DTNNQLAVQVRQ  108
215  IDV-N----DTITEGAA--TFEECVAKLRILAGDVDVDKFLATTM-A-DTNNQLAIQVRQ  108
216  IDV-N----DTITEGAA--TFEEKVAKLRILAGDVDVDKFLATTM-A-DTNNQLAIQVRQ  108
217  IDV-N----DTIPEGAA--TFEECVAKLRILAGDVDVDKFLATTM-A-DTNNQLAIQVRQ  108
218  IDV-N----DTIPEGAA--TFEECVAKLRILAGDVDVDKFLATTM-A-DTNNQLAIQVRQ  108
219  IDV-N----DTITEGAA--TFEECVAKLRILAGDVDVDKFLATTM-A-DTNNQLAIQVRQ  108
220  IDV-N----DTITEGAA--TFEECVAKLRILAGDVDVDKFLATTM-A-DTNNQLAIQVRQ  108
221  IDV-N----DTIPEGAA--TFEECIAKLRILAGDVDVDKFLATTM-A-DTNNQLAIQVRQ  108
222  IDV-N----DTIPEGAA--TFEECVAKLRILAGDVDVDKFLATTM-A-DTNNQLAIQVRQ  108
223  IDV-N----DTIPEGAA--TFEECVAKLRILAGDVDVDKFLATTM-A-DTNNQLAIQVRQ  108
224  IDV-N----DTITEGAA--TFEEKVAKLRILAGDVDVDKFLATTM-A-DTNNQLAIQVRQ  108
225  IDV-N----DTIPEGAA--TFEECVAKLRILAGDVDVDKFLATTM-A-DTNNQLAIQVRQ  108
226  IDV-N----DTIPEGAA--TFEECVAKLRILAGDVDVDKFLATTM-A-DTNNQLAIQVRQ  108
227  IDV-N----DTIPEGAA--TFEECVAKLRILAGDVDVDKFLATTM-A-DTNNQLAIQVRQ  108
228  LDP-N----EVVNESAS--TFTEVVAKLRILIGDVDVDKFLQTTQ-S-DHNNQLAIQIAK  108
229  IDV-N----DTITEGAA--TFEECVAKLRILAGDVDVDKFLATTM-A-DTNNQLAIQVRQ  108
230  LDP-N----EVVNESAS--TFTEVVAKLRILIGDVDVDKFLQTTQ-S-DHNNQLAIQIAK  108
231  IDV-N----DTIPEGAA--TFEECVAKLRILAGDVDVDKFLATTM-A-DTNNQLAIQVRQ  108
232  IDV-N----DTIAEGAA--TFSEHVAKLRILAGDVDVDKFLATTM-A-DTNNQLAIQVRQ  108
233  IDV-N----DTITEGAA--TFEECVAKLRILAGDVDVDKFLATTM-A-DTNNQLAIQVRQ  108
234  IDV-N----DTIPEGAA--TFEECVAKLRILAGDVDVDKFLATTM-A-DTNNQLAIQVRQ  108
235  IDV-N----DTIPEGAA--TFEEKVAKLRILAGDVDVDKFLATTM-A-DTNNQLAIQVRQ  108
236  IDV-N----DTITEGAA--TFEECVAKLRILAGDVDVDKFLATTM-A-DTNNQLAIQVRQ  108
237  IDV-N----DTIPEGAA--TFEECVAKLRILAGDVDVDKFLATTM-A-DTNNQLAIQVRQ  108
238  IDV-N----DTITEGAA--TFEEKVAKLRILAGDVDVDKFLATTM-A-DTNNQLAIQVRQ  108
239  YDV-G----DTWTESTP--TFEQVTTGLKILGGDADIDHFLQTY-R-DPNQLEAVVLQQ  108
240  IDV-N----DTIAEGAA--TFSEHVAKLRILAGDVDVDKFLATTM-S-DTNNQLAVQVRQ  108
241  IDV-N----DTITEGAA--TFSEHVAKLRILAGDVDVDKFLATTM-S-DTNNQLAIQVRQ  108
242  IDV-N----DTIPEGAA--TFEECVAKLRILAGDVDVDKFLATTM-A-DTNNQLAIQVRQ  108
243  IDV-N----DTITEGAA--TFEEKVAKLRILAGDVDVDKFLATTM-A-DTNNQLAIQVRQ  108
244  IDV-N----DTITEGAA--TFEECVAKLRILAGDVDVDKFLATTM-A-DTNNQLAIQVRQ  108
245  IDV-N----DTIPEGAA--TFEECVAKLRILAGDVDVDKFLATTM-A-DTNNQLAIQVRQ  108
246  IDV-N----DTITEGAA--TFEEKVAKLRILAGDVDVDKFLATTM-A-DTNNQLAIQVRQ  108
247  IDV-N----DTITEGAA--TFEECVAKLRILAGDVDVDKFLATTM-A-DTNNQLAIQVRQ  108
248  IDV-N----DTIPEGAA--TFEECVAKLRILAGDVDVDKFLATTM-A-DTNNQLAIQVRQ  108
249  IDV-N----DTIPEGAA--TFEECVAKLRILAGDVDVDKFLATTM-A-DTNNQLAIQVRQ  108
250  IDV-N----DTIPEGAA--TFEECVAKLRILAGDVDVDKFLATTM-A-DTNNQLAIQVRQ  108
251  IDV-N----DTITEGAA--TFTEHVAKLRILAGDVDVDKFLATTM-S-DTNNQLAIQVRQ  108
252  IDV-N----DTISEGAA--TFEECVAKLRILAGDVDVDKFLATTM-A-DTNNQLAIQVRQ  108
253  IDV-N----DTIPEGAA--TFEECVAKLRILAGDVDVDKFLATTM-A-DTNNQLAIQVRQ  108
254  IDV-N----DTIPEGAA--TFEECVAKLRILAGDVDVDKFLATTM-A-DTNNQLAIQVRQ  108
255  IDV-N----DTIPEGAA--TFEECVAKLRILAGDVDVDKFLATTM-A-DTNNQLAIQVRQ  108
256  IDV-N----DTITEGAA--TFSEHVAKLRILAGDVDVDKFLATTM-S-DTNNQLAIQVRQ  108
257  IDV-N----DTITEGAA--TFSEHVAKLRILAGDVDVDKFLATTM-S-DTNNQLAIQVRQ  108
```

```
258  IDV-N----DTIPEGAA--TFEECVAKLRILAGDVDVDKFLATTM-A-DTNNQLAIQVRQ  108
259  IDV-N----DTITEGAA--TFEECVAKLRILAGDVDVDKFLATTM-A-DTNNQLAIQVRQ  108
260  IDV-N----DTITEGAA--TFEEKVAKLRILAGDVDVDKFLATTM-A-DTNNQLAIQVRQ  108
261  IDV-N----DTITEGAA--TFEEKVAKLRILAGDVDVDKFLATTM-A-DTNNQLAIQVRQ  108
262  IDV-N----DTIPEGAA--TFEECVAKLRILAGDVDVDKFLATTM-A-DTNNQLAIQVRQ  108
263  IDV-N----DTISEGAA--TFTEHVAKLRILAGDVDVDKFLATTM-S-DTNSQLAIQVRQ  108
264  IDV-N----DTITEGAA--TFTEHVAKLRILAGDVDVDKFLATTM-S-DTNNQLAIQVRQ  108
265  IDV-N----DTITEGAA--TFEEKVAKLRILAGDVDVDKFLATTM-A-DTNNQLAIQVRQ  108
266  IDV-N----DTIPEGAA--TFEECVAKLRILAGDVDVDKFLATTM-A-DTNNQLAIQVRQ  108
267  IDV-N----DTITEGAA--TFEEKVAKLRILAGDVDVDKFLATTM-A-DTNNQLAIQVRQ  108
268  IDV-N----DTITEGAA--TFEEKVAKLRILAGDVDVDKFLATTM-A-DTNNQLAIQVRQ  108
269  IDV-N----DTITEGAA--TFSEHVAKLRILAGDVDVDKFLATTM-S-DTNNQLAIQVRQ  108
270  LDP-N----DTVTESAS--TFTEVVAKLRILIGDVDVDKFLQATM-S-DHNNQLAIQIAK  108
271  IDV-N----DTIPEGAA--TFEECVAKLRILAGDVDVDKFLATTM-A-DTNNQLAIQVRQ  108
272  IDV-N----DTITEGAA--TFEEKVAKLRILAGDVDVDKFLATTM-A-DTNNQLAIQVRQ  108
273  IDV-N----DTITEGAA--TFEEKVAKLRILAGDVDVDKFLATTM-A-DTNNQLAIQVRQ  108
274  IDV-N----DTITEGAA--TFEEKVAKLRILAGDVDVDKFLATTM-A-DTNNQLAIQVRQ  108
275  IDV-N----DTITEGAA--TFEECVAKLRILAGDVDVDKFLATTM-A-DTNNQLAIQVRQ  108
276  IDV-N----DTITEGAA--TFEEKVAKLRILAGDVDVDKFLATTM-A-DTNNQLAIQVRQ  108
277  IDV-N----DTITEGAA--TFEECVAKLRILAGDVDVDKFLATTM-A-DTNNQLAIQVRQ  108
278  IDV-N----DTITEGAA--TFEEKVAKLRILAGDVDVDKFLATTM-A-DTNNQLAIQVRQ  108
279  IDV-N----DTIPEGAA--TFEECVAKLRILAGDVDVDKFLATTM-A-DTNNQLAIQVRQ  108
280  IDV-N----DTITEGAA--TFEEKVAKLRILAGDVDVDKFLATTM-A-DTNNQLAIQVRQ  108
281  LDP-N----EVIEEEGS--TFDEVTAHLRILAGDVDVDKFLDSTM-G-DTNAQKAIQIKQ  108
282  IDV-N----DTITEGAA--TFEEKVAKLRILAGDVDVDKFLATTM-A-DTNNQLAIQVRQ  108
283  IDV-N----DTIPEGAA--TFEECVAKLRILAGDVDVDKFLATTM-A-DTNNQLAIQVRQ  108
284  IDV-N----DTIPEGAA--TFEECVAKLRILAGDVDVDKFLATTM-A-DTNNQLAIQVRQ  108
285  IDV-N----DTIPEGAA--TFEECVAKLRILAGDVDVDKFLATTM-A-DTNNQLAIQVRQ  108
286  IDV-N----DTITEGAA--TFTEHVAKLRILAGDVDVDKFLATTM-S-DTNNQLAIQVRQ  108
287  IDV-N----DTITEGAA--TFEEKVAKLRILAGDVDVDKFLATTM-A-DTNNQLAIQVRQ  108
288  IDV-N----DTITEGAA--TFTEHVAKLRILAGDVDVDKFLATTM-S-DTNNQLAIQVRQ  108
289  IDV-N----DTITEGAA--TFEEKVAKLRILAGDVDVDKFLATTM-A-DTNNQLAIQVRQ  108
290  IDV-N----DTITEGAA--TFEECVAKLRILAGDVDVDKFLATTM-A-DTNNQLAIQVRQ  108
291  IDV-N----DTIPEGAA--TFEECVAKLRILAGDVDVDKFLATTM-A-DTNNQLAIQVRQ  108
292  IDV-N----DTIPEGAA--TFEECVAKLRILAGDVDVDKFLATTM-A-DTNNQLAIKVRQ  108
293  IDV-N----DTITEGAA--TFEEKVAKLRILAGDVDVDKFLATTM-A-DTNNQLAIQVRQ  108
294  IDV-N----DTITEGAA--TFEEKVAKLRILAGDVDVDKFLATTM-A-DTNNQLAIQVRQ  108
295  IDV-N----DTITEGAA--TFEEKVAKLRILAGDVDVDKFLATTM-A-DTNNQLAIQVRQ  108
296  IDV-N----DTITEGAA--TFEEKVAKLRILAGDVDVDKFLATTM-A-DTNNQLAIQVRQ  108
297  IDV-N----DTITEGAA--TFEEKVAKLRILAGDVDVDKFLATTM-A-DTNNQLAIQVRQ  108
298  IDV-N----DTIPEGAA--TFEECVAKLRILAGDVDVDKFLATTM-A-DTNNQLAIQVRQ  108
299  IDV-N----DTITEGAA--TFEEEVAKLRILAGDVDVDKFLATTM-A-DTNNQLAIQVRQ  108
300  IDV-N----DTIPEGAA--TFEECVAKLRILAGDVDVDKFLATTM-A-DTNNQLAIQVRQ  102
301  IDV-N----DTITEGAA--TFEEKVAKLRILAGDVDVDKFLATTM-A-DTNNQLAIQVRQ  108
302  IDV-N----DTITEGAA--TFEEKVAKLRILAGDVDVDKFLATTM-A-DTNNQLAIQVRQ  108
303  IDV-N----DTIAEGAA--TFTEHVAKLRILAGDVDVDKFLATTM-S-DTNNQLAIQVRQ  108
304  IDV-N----DTITEGAA--TFEEKVAKLRILAGDVDVDKFLATTM-A-DTNNQLAIQVRQ  108
305  IDV-N----DTITEGAA--TFEEKVAKLRILAGDVDVDKFLDTTM-A-DTNNQLAIQVRQ  108
306  IDV-N----DTITEGAA--TFEEKVAKLRILAGDVDVDKFLATTM-A-DTNNQLAIQVRQ  108
307  IDV-N----DTITEGAA--TFTEHVAKLRILAGDVDVDKFLATTM-S-DTNNQLAIQVRQ  108
308  IDV-N----DTITEGAA--TFEEKVAKLRILAGDVDIDKFLATTM-A-DTNNQLAIQVRQ  108
309  IDV-N----DTITEGAA--TFTEHVAKLRILAGDVDVDKFLATTM-S-DTNNQLAIQVRQ  108
310  IDV-N----DTITEGAA--TFTEHVAKLRILAGDVDVDKFLATTM-S-DTNNQLAIQVRQ  108
311  IDV-N----DTITEGAA--TFTEHVAKLRILAGDVDVDKFLATTM-S-DTNNQLAIQVRQ  108
312  IDV-N----DTITEGAA--TFEEKVAKLRILAGDVDVDKFLATTM-A-DTNNQLAIQVRQ  108
313  IDV-N----DTIQEGAA--TFEECVAKLRILAGDVDVDKFLATTM-A-DTNNQLAIQVRQ  108
314  IDV-N----DTITEGAA--TFTEHVAKLRILAGDVDVDKFLATTM-S-DTNNQLAIQVRQ  108
315  IDV-N----DTIPEGAA--TFEECVAKLRILAGDVDVDKFLATTM-A-DTNNQLAIQVRQ  108
316  IDV-N----DTITEGAA--TFEEKVAKLRILAGDVDVDKFLATTM-A-DTNNQLAIQVRQ  108
317  IDV-N----DTITEGAA--TLTEHVAKLRILAGDVDVDKFLATTM-S-DTNNQLAIQVRQ  108
318  IDV-N----DTITEGAA--TFEEKVAKLRILAGDVDVDKFLATTM-A-DTNNQLAIQVRQ  108
319  IDV-N----DTITEGAA--TFEEKVAKLRILAGDVDVDKFLATTM-A-DTNNQLAIQVRQ  108
320  IDV-N----DTITEGAA--TFTEHVAKLRILAGDVDVDKFLATTM-S-DTNNQLAIQVRQ  108
321  IDV-N----DTITEGAA--TFEERVAKLRILAGDVDVDKFLATTM-A-DTNNQLAIQVRQ  108
322  IDV-N----DTITEGAA--TFTEHVAKLRILAGDVDVDKFLATTM-S-DTNNQLAIQVRQ  108
323  IDV-N----DTITEGAA--TFTEHVAKLRILAGDVDVDKFLATTM-S-DTNNQLAIQVRQ  108
324  IDV-N----DTIPEGAA--TFEECVAKLRILAGDVDVDKFLATTM-A-DTNNQLAIQVRQ  108
```

```
325 IDV-N----DTIPEGAA--TFEECVAKLRILAGDVDVDKFLATTM-A-DTNNQLAIQVRQ 108
326 IDV-N----DTITEGAA--TFEEKVAKLRILAGDVDVDKFLATTM-A-DTNNQLAIQVRQ 108
327 IDV-N----DTITEGAA--TFEEKVAKLRILAGDVDVDKFLATTM-A-DTNNQLAIQVRQ 108
328 IDV-N----DTITEGAA--TFEEKVAKLRILAGDVDVDKFLATTM-A-DTNNQLAIQVRQ 108
329 IDV-N----DTITEGAA--TFTEHVAKLRILAGDVDVDKFLATTM-S-DTNNQLAIQVRQ 108
330 IDV-N----DTITEGAA--TFTEHVAKLRILAGDVDVDKFLATTM-S-DTNNQLAIQVRQ 108
331 IDV-N----DTITEGAA--TFEEKVAKLRILAGDVDVDKFLATTM-A-DTNNQLAIQVRQ 108
332 IDV-N----DTITEGAA--TFTEHVAKLRILAGDVDVDKFLATTM-S-DTNNQLAIQVRQ 108
333 IDV-N----DTITEGAA--TFTEHVAKLRILAGDVDVDKFLATTM-S-DTNNQLAIQVRQ 108
334 IDV-N----DTITEGAA--TFTEHVAKLRILAGDVDVDKFLATTM-S-DTNSQLAIQVRQ 108
335 IDV-N----DTIAEGAA--TFTEHVAKLRILAGDVDVDKFLATTM-S-DTNNQLAIQVRQ 108
336 IDV-N----DTITEGAA--TFEEKVAKLRILAGDVDVDKFLATTM-A-DTNNQLAIQVRQ 108
337 IDV-N----DNITEGAA--TFTEHVAKLRILAGDVDVDKFLATTM-S-DTNNQLAIQVRQ 108
338 IDV-N----DTITEGAA--TFEEKVAKLRILAGDVDVDKFLATTM-A-DTNNQLAIQVRQ 108
339 IDV-N----DTIAEGAA--TFTEHVAKLRILAGDVDVDKFLATTM-S-DTNNQLAIQVRQ 108
340 IDV-N----DTITEGAA--TFEEKVAKLRILAGDVDVDKFLATTM-A-DTNNQLAIQVRQ 108
341 IDV-N----DTITEGAA--TFTEHVAKLRILAGDVDVDKFLATTM-S-DTNNQLAIQVRQ 108
342 IDV-N----DTIAEGAA--TFSEHVAKLRILAGDVDVDKFLATTM-S-DTNNQLAVQVRQ 108
343 IDV-N----DTITEGAA--TFTEHVAKLRILAGDVDVDKFLATTM-S-DTNNQLAIQVRQ 108
344 IDV-N----DTITEGAA--TFTEHVAKLRILAGDVDVDKFLATTM-S-DTNNQLAIQVRQ 108
345 IDV-N----DTITEGAA--TFEEKVAKLRILAGDVDVDKFLATTM-A-DTNNQLAIQVRQ 108
346 IDV-N----DTITEGAA--TFEECVAKLRILAGDVDVDKFLATTM-A-DTNNQLAIQVRQ 108
347 IDV-N----DTIPEGAA--TFEECVAKLRILAGDVDVDKFLATTM-A-DTNNQLAIQVRQ 108
348 IDV-N----DTISEGAA--TFTEHVAKLRILAGDVDVDKFLATTM-S-DTNSQLAIQVRQ 108
349 IDV-N----DTITEGAA--TFSEHVAKLRILAGDVDVDKFLATTM-S-DTNNQLAIQVRQ 108
350 IDV-N----DTITEGAA--TFTEHVAKLRILAGDVDVDKFLATTM-S-DTNNQLAIQVRQ 108
351 LDP-N----EVIEEEGS--TFDEVTAHLRILAGDVDVDKFLDSTM-G-DTNAQKAIQIKQ 89
352 IDV-N----DTIAEGAA--TFTEHVAKLRILAGDVDVDKFLATTM-S-DTNNQLAIQVRQ 108
353 AGVGT----TITAKSPA--TFTQVTSSLTSLLGDAEVNGLIQATR-S-NIQDQKAIQVAS 110
354 IDV-N----DTITEGAA--TFEEKVAKLRILAGDVDVDKFLATTM-A-DTNNQLAIQVRQ 105
355 IDV-N----DTITEGAA--TFTEHVAKLRILAGDVDVDKFLATTM-S-DTNSQLAIQVRQ 108
356 IDV-N----DTIAEGAA--TFTEHVAKLRILAGDVDVDKFLATTM-S-DTNNQLAIQVRQ 108
357 IDV-N----DTITEGAA--TFEEKVAKLRILAGDVDVDKFLATTM-A-DTNNQLAIQVRQ 108
358 IDV-N----DTITEGAA--TFSEHVAKLRILAGDVDVDKFLATTM-S-DTNNQLAIQVRQ 108
359 IDV-N----DTITEGAA--TLEEKVAKLRILAGDVDVDKFLATTM-A-DTNNQLAIQVRQ 108
360 IDV-N----DTITEGAA--TFTEHVAKLRILAGDVDVDKFLATTM-S-DTNNQLAIQVRQ 108
361 IDV-N----DTITEGAA--TFTEHVAKLRILAGDVDVDKFLATTM-A-DTNNQLAIQVRQ 108
362 IDV-N----DTITEGAA--TFEEKVAKLRILAGDVDVDKFLATTM-A-DTNNQLAIQVRQ 108
363 IDV-N----DTITEGAA--TFTEHVAKLRILAGDVDVDKFLATTM-S-DTNNQLAIQVRQ 108
364 IDV-N----DTITEGAA--TFTEHVAKLRILAGDVDVDKFLATTM-S-DTNNQLAIQVRQ 108
365 IDV-N----DTITEGAA--TFTEHVAKLRILAGDVDVDKFLATTM-S-DTNSQLAIQVRQ 108
366 IDV-N----DTITEGAA--TFTEHVAKLRILAGDVDVDKFLATTM-S-DTNNQLAIQVRQ 108
367 IDV-N----DTISEGAA--TFTEHVAKLRILAGDVDVDKFLATTM-S-DTNSQLAIQVRQ 108
368 IDV-N----DTIAEGAA--TFTEHVAKLRILAGDVDVDKFLATTM-S-DTNSQLAIQVRQ 108
369 IDV-N----DTITEGAA--TFTEHVAKLRILAGDVDVDKFLATTM-S-DTNNQLAIQVRQ 108
370 IDV-N----DTIIEGAA--TFTEHVAKLRILAGDVDVDKFLATTM-S-DTNNQLAIQVRQ 108
371 IDV-N----DTISEGAA--TFTEHVAKLRILAGDVDVDKFLATTM-S-DTNNQLAIQVRQ 108
372 IDV-N----DTIAEGAA--TFTEHVAKLRILAGDVDVDKFLATTM-S-DTNNQLAIQVRQ 108
373 IDV-N----DTITEGAA--TFIEHVAKLRILAGDVDVDKFLATTM-S-DTNNQLAIQVRQ 108
374 IDV-N----DTITEGAA--TFTEHVAKLRILAGDVDVDKFLATTM-S-DTNNQLAIQVRQ 108
375 IDV-N----DTIAEGAA--TFTEHVAKLRILAGDVDVDKFLATTM-S-DTNNQLAIQVRQ 108
376 IDV-N----DTITEGAA--TFEEKVAKLRILAGDVDVDKFLATTM-A-DTNNQLAIQVRQ 108
377 YEV-G----DTWTESTP--TFTQVTTGLKILGGDADIDHFLQTY-R-DPNELEAIVIQS 108
378 IDV-N----DTITEGAA--TFTEHVAKLRILAGDVDVDKFLATTM-S-DTNNQLAIQVRQ 108
379 IDV-N----DTITEGAA--TFTEHVAKLRILAGDVDVDKFLATTM-A-DTNNQLAIQVRQ 108
380 IDV-N----DTISEGAA--TFTEHVAKLRILAGDVDVDKFLATTM-S-DTNSQLAIQVRQ 108
381 IDV-N----DTISEGAA--TFTEHVAKLRILAGDVDVDKFLATTM-S-DTNSQLAIQVRQ 108
382 IGVGD----TITAKAAA--TFTQVTSNLTKIIGDAEVDGLIQATR-S-ADNDQTGVQIAS 110
383 IDV-N----DTIAEGAA--TFTEHVAKLRILAGDVDVDKFLATTM-S-DTNNQLAIQVRQ 108
384 IDV-N----DTISEGAA--TFTEHVAKLRILAGDVDVDKFLATTM-S-DTNSQLAIQVRQ 108
385 IDV-N----DTITEGAA--TFTEHVAKLRILAGDVDVDKFLATTM-S-DTNNQLAIQVRQ 108
386 IDV-N----DTITEGAA--TFEEKVAKLRILAGDVDVDKFLATTM-A-DTNNQLAIQVRQ 108
387 IDPYN----DTVPEEAS--TVTDVTVTLKTLIGDVDVDKFSEATK-S-DTNDQKALQIAM 109
388 IDV-N----DTIAEGAA--TFTEHVAKLRILAGDVDVDKFLATTM-S-DTNSQLAIQVRQ 108
389 IDV-N----DTITEGAA--TFEEKVAKLRILAGDVDVDKFLATTM-A-DTNNQLAIQVRQ 108
390 AGVGA----TITAKAAT--TFTSVTSVLTSILGDAEVNGLVQATQ-S-PDTDQTAQQIAS 110
391 IDV-N----DTISEGAA--TFTEHVAKLRILAGDVDVDKFLATTM-S-DTNSQLAIQVRQ 108
```

```
392  IDV-N----DTITEGAA--TFTEHVAKLRILAGDVDVDKFLATTM-S-DTNNQLAIQVRQ  108
393  IDV-N----DTITEGAA--TFTEHVAKLRILAGDVDVDKFTATTM-S-DTNNQLAIRVRQ  108
394  IDV-N----DTISEGAA--TFTEHVAKLRILAGDVDVDKFLATTM-S-DTNSQLAIQVRQ  108
395  IDV-N----DTIAEGAA--TFTEHVAKLRILAGDVDVDKFLATTM-S-DTNSQLAIQVRQ  108
396  IDV-N----ETIAEGAA--TFTEHVAKLRILAGDVDVDKFLATTM-A-DTNNQLAIQVRQ  108
397  AGVGS----TITAKNPA--TFTQVTSTLTTIIGDAEVNGLIQATR-SGDGNDQTAVQVAS  111
398  IDV-N----DTIPEGAA--TFEECVAKLRILAGDVDVDKFLATTM-A-DTNNQLAIQVRQ  108
399  IDV-N----DTIAEGAA--TFTEHVAKLRILAGDVDVDKFLATTM-S-DTNNQLAIQVRQ  108
400  IDV-N----DTISEGAA--TFTEHVAKLRILAGDVDVDKFLATTM-S-DTNSQLAIQVRQ  108
401  IDV-N----DTISEGAA--TFTEHVAKLRILAGDVDVDKFLATTM-S-DTNSQLAIQVRQ  108
402  IDV-N----DTIAEGAA--TFTEHVAKLRILAGDVDVDKFLATTM-S-DTNSQLAIQVRQ  108
403  IDV-N----DTITEGAA--TFEEKVAKLRILAGDVDVDKFLATTM-A-DTNNQLAIQVRQ  108
404  IDV-N----DTITEGAA--TFEEKVAKLRILAGDVDVDKFLATTM-A-DTNNQLAIQVRQ  108
405  IDV-N----DTIPEGAA--TFEECVAKLRILAGDVDVDKFLATTM-A-DTNNQLAIQVRQ  108
406  IDV-N----DTIAEGAA--TFTEHVAKLRILAGDVDVDKFLATTM-S-DTNSQLAIQVCQ  108
407  IDV-N----DTITEGAA--TFEEKVAKLRILAGDVDVDKFLATTM-A-DTNNQLAIQVRQ  108
408  IDV-N----DTITEGAA--TFTEHVAKLRILAGDVDVDKFLATTM-S-DTNNQLAIQVRQ  108
409  IDV-N----DTISEGAA--TFTEHVAKLRILAGDVDVDKFLATTM-S-DTNSQLAIQVRQ  108
410  IDV-N----DTISEGAA--TFTEHVAKLRILAGDVDVDKFLATTM-S-DTNSQLAIQVRQ  108
411  IDV-N----DTIAEGAA--TFTEHVAKLRILAGDVDVDKFLATTM-S-DTNSQLAIQVRQ  108
412  IDV-N----DTISEGAA--TFTEHVAKLRILAGDVDVDKFLATTM-S-DTNSQLAIQVRQ  108
413  IDV-N----DTIPEGAA--TFEECVAKLRILAGDVDVDKFLATTM-A-DTNNQLAIQVRQ  108
414  IDV-N----DTIAEGAA--TFTEHVAKLRILAGDVDVDKFLATTM-S-DTNSQLAIQVRQ  108
415  IDV-N----DTIAEGAA--TFTEHVAKLRILAGDVDVDKFLATTM-S-DTNSQLAIQVRQ  108
416  IDV-N----DTISEGAA--TFTEHVAKLRILAGDVDVDKFLATTM-S-DTNSQLAIQVRQ  108
417  IDV-N----ETIAEGAA--TFTEHVAKLRILAGDVDVDKFLATTM-A-DTNNQLAIQVRQ  108
418  IDV-N----DTITEGAA--TFTEHVAKLRILAGDVDVDKFLATTM-S-DTNNQLAIQVRQ  108
419  YDA-Y----EDIEESAA--KYVEVETKLKVLAGQVDIDNFTAETQ-S-ELNDQVALQLAA  108
420  IDV-N----DTIAEGAA--TFTEHVAKLRILAGDVDVDKFLATTM-S-DTNSQLAIQVRQ  108
421  IDV-N----DTIPEGAA--TFEECVAKLRILAGDVDVDKFLATTM-A-DTNNQLAIQVRQ  108
422  IDV-N----DTIAEGAA--TFSEHVAKLRILAGDVDVDKFLATTM-S-DTNNQLAVQVRQ  108
423  IDV-N----ETIAEGAA--TFTEHVAKLRILAGDVDVDKFLATTM-A-DTNNQLAIQVRQ  108
424  IDV-N----DTISEGAA--TFTEHVAKLRILAGDVDVDKLLATTM-S-DTNSQLAIQVRQ  108
425  IDV-N----DTIAEGAA--TFSEHVAKLRILAGDVDVDKFLATTM-S-DTNNQLAVQVRQ  108
426  IDV-N----DTIAEGAA--TFSEHVAKLRILAGDVDVDKFLATTM-S-DTNNQLAVQVRQ  108
427  IDV-N----DTIAEGAA--TFSEHVAKLRILAGDVDVDKFLATTM-S-DTNNQLAVQVRQ  108
428  IDV-N----DTISEGAA--TFTEHVAKLRILAGDVDVDKFLATTM-S-DTNSQLAIQVRQ  108
429  IDV-N----DTIPEGAA--TFEECVAKLRILAGDVDVDKFLATTM-A-DTNNQLAIQVRQ  108
430  IDV-N----DTIAEGAA--TFSEHVAKLRILAGDVDVDKFLATTM-S-DTNNQLAVQVRQ  108
431  IDV-N----DTISEGAA--TFTEHVAKLRILAGDVDVDKFLATTM-S-DTNSQLAIQVRQ  108
432  ATVGD----TITAKNAA--TFTKVNSNLTTILGDAEVNGLIQATR-S-ATTDQEATQIAS  110
433  IDV-N----DTITEGAA--TFEEKVAKLRILAGDVDVDKFLATTM-A-DTNNQLAIQVRQ  108
434  IDV-N----DTITEGAA--TFEEKVAKLRILAGDVDVDKFLATTM-A-DTNNQLAIQVRQ  108
435  IDV-N----DTITEGAA--TFEEKVAKLRILAGDVDVDKFLATTM-A-DTNNQLAIQVRQ  108
436  IDV-N----DTIAEGAA--TFTEHVAKLRILAGDVDVDKFLATTM-S-DTNSQLAIQVRQ  108
437  IDV-N----DVVPEGAA--TFEECVAKLRILAGDVDVDKFLATTM-A-DTNNQLAIQVRQ  108
438  IDV-N----DTIAEGAA--TFTEHVAKLRILAGDVDVDKFLATTM-S-DTNSQLAIQVRQ  108
439  LDP-N----DTVQESAS--TFTEVVAKLRILIGDVDVDKFLQATM-S-DHNNQLAIQIAK  108
440  IDV-N----DTIPEGAA--TFEECVAKLRILAGDVDVDKFLATTM-A-DTNNQLAIQVRQ  108
441  YEV-G----DTWTESTP--TFTQVTTGLKILGGDADIDHFLLQTY-R-DPNELEAIVIQS  108
442  IDV-N----DVIPEGAA--KFTEHVAKLRILAGDVDVDKFLASTM-G-DTNSQLAIQIRA  108
443  IDV-N----EVIPEGAA--TFTEHTAKLRIMAGDVDVDKFLATTM-D-DTNSQLAIQVRQ  108
444  LGVGG----TITAKAAT--TFTQVTSPLKRIVGDAEVDNFVEATH-S-DTTDQEAVQVAG  110
445  IGVGG----TITAKTAA--TFTQINSNLTKIIGDAEVDNMIQATR-S-SINDQTGQQIAS  110
446  IDV-N----DTIPEGAA--TFEECVAKLRILAGDVDVDKFLATTM-A-DTNNQLAIQVRQ  108
447  IDV-N----DTIPEGAA--TFEECVAKLRILAGDVDVDKFLATTM-A-DTNNQLAIQVRQ  108
448  YDV-G----DTWAEATP--TFTQVTVPLRILGGDADIDNFLQQTY-A-NPNDLEAVVLES  106
449  IDV-N----DTIPEGAA--TFEECVAKLRILAGDVDVDKFLSTTM-A-DTNNQLAIQVRQ  108
450  IDV-N----DTITEGAA--TFEEKVAKLRILAGDVDVDKFLATTM-A-DTNNQLAIQVRQ  108
451  YDV-G----DTWTESTP--TFEQVTTGLKILGGDADIDHFLLQTY-R-DPNQLEAVVLQQ  108
452  YDV-G----DTWEEATP--EFTQVTVALKILGGDADVDNFLQQTY-A-DPNDLEAVVIES  108
453  IDV-N----DTIAEGAA--TFTEHVAKLRILAGDVDVDKFLATTM-S-DTNSQLAIQVRQ  108
454  AGVGS----TITAKAPA--TFTQVTSTLTTIIGDAEVNGLIQATR-SGDGNDQTAVQVAS  111
455  LSP-N----DTVNEDAG--TFDEVVSKLRILAGDVDVDKFLQETD-S-DTNDQRATQIAM  108
456  IDV-N----DTITEGAA--TFEEKVAKLRILAGDVDVDKFLATTM-A-DTNNQLAIQVRQ  108
457  IDV-N----DTIPEGAA--TFEECVAKLRILAGDVDVDKFLATTM-A-DTNNQLAIQVRQ  108
458  IDV-N----DTISEGAA--TFTEHVAKLRILAGDVDVDKFLATTM-S-DTNSQLAIQVRQ  108
```

```
459  LDP-N----DVVPESTG--DFTEVVTKLRILAGDVDVDKFLAETM-S-DTENQLATQIGL  108
460  IDV-N----DTIPEGAA--TFEECVAKLRILAGDVDVDKFLATTM-A-DTNNQLAIQVRQ  108
461  AGVGS----TITAKNPA--TFTQVTSTLTTIIGDAEVNGLIQATR-SGDGNDQTAVQVAS  111
462  IDV-N----DTIPEGAA--TFEECVAKLRILAGDVDVDKFLATTM-A-DTNNQLAIQVRQ  108
463  --------------------------------------MASQDIF-R-HRNDLVDFSVTL  20
464  IDV-N----DTIAEGAA--TFSEHVAKLRILAGDVDVDKFLATTM-S-DTNNQLAVQVRQ  108
465  IDV-N----DTIPEGAA--TFEECVAKLRILAGDVDVDKFLATTM-A-DTNNQLAIQVRQ  108
466  IDV-N----DTIPEGAA--TFEECVAKLRILAGDVDVDKFLATTM-A-DTNNQLAIQVRQ  108
467  IDV-N----DTITEGAA--TFEEKVAKLRILAGDVDVDKFLATTM-A-DTNNQLAIQVRQ  108
468  IDV-N----DTIPEGAA--TFEECVAKLRILAGDVDVDKFLATTM-A-DTNNQLAIQVRQ  108
469  IDV-N----DVVPEGAA--TFTEHVAKLRIIAGDVDVDKFLASTM-G-DTNSQLAIQVRQ  108
470  IDV-N----DTIPEGAA--TFEECVAKLRILAGDVDVDKFLATTM-A-DTNNQLAIQVRQ  108
471  AGVDA----TITAKAPA--TFTKVTSELTTIIGDAEVNGLIQATR-SSDGNDQTAIQVAS  111
472  LDP-N----DPINESAA--TFEEVVAKLRILAGDVDVDKFLNQTM-S-DTNSQMATQIAK  108
473  AGVDA----TITAKAPA--TFTKVTSELTTIIGDAEVNGLIQATR-SSDGNDQTAIQVAS  111
474  IDV-N----DTIPEGAA--TFEECVAKLRILAGDVDVDKFLATTM-A-DTNNQLAIQVRQ  108
475  IDV-N----DTITEGAA--TFTEHVAKLRILAGDVDVDKFLATTM-S-DTNNQLAIQVRQ  108
476  AGVGS----TITAKNPA--TFTQVTSTLTTIIGDAEVNGLIQATR-SGDGNDQTAVQVAS  111
477  AGVGD----TITAKNPA--TFEQITSSLTTLIGDAEVNGLIQATR-S-NINDQKTVQIMS  110
478  LGVGG----TITAKAPT--TFTQVTSALKRIVGDAEVDNFVEATH-S-DTTDQLAVQVAG  110
479  AGVGT----TITAKNPA--TFTHVTSTLTTIIGDAEVNGLIQATR-SGDGNDQTAVQVAS  111
480  AGVDA----TITAKAPA--TFTKVTSELTTIIGDAEVNGLIQATR-SSDGNDQTAVQVAS  111
481  IDV-N----DTIPEGAA--TFEECVAKLRILAGDVDVDKFLATTM-A-DTNNQLAIQVRQ  108
482  IDV-N----DTIPEGAA--TFEECVAKLRILAGDVDVDKFLATTM-A-DTNNQLAIQVRQ  108
483  IDV-N----DVVPEGAA--KFTEHVAKLRILAGDVDVDKFLATTM-A-DTNSQLAIQIRS  108
484  IDV-N----DTIPEGAA--TFEECVAKLRILAGDVDVDKFLATTM-A-DTNNQLAIQVRQ  108
485  YDV-G----DTWQEATP--EFTQVTVALKILGGDADVDNFLQQTY-A-DPNDLEAIVIES  108
486  IDV-N----DIVPEGAA--KFTEHVAKLRILAGDVDVDKFLATTM-A-DTNSQLAIQIRA  108
487  IDV-N----DTISEGAA--TFTEHVAKLRILAGDVDVDKFLATTM-S-DTNSQLAIQVRQ  108
488  IDV-N----DEVPEGAA--KFSEHVAKLRILAGDVDVDKFLASTM-G-DTNSQMAIQIRS  108
489  IDV-N----DVVPEGAA--KFTEHVAKLRIMAGDVDVDKFLATTM-A-DTNSQLAIQIRA  108
490  AGVGS----TITAKNPA--TFTQVTSTLTTIIGDAEVNGLIQATR-SGDGNDQTAVQVAS  111
491  IDV-N----DTIPEGAA--TFEECVAKLRILAGDVDVDKFLATTM-A-DTNNQLAIQVRQ  108
492  LGVGS----SITAKAAT--TFTQVTSPLKRIVGDAEVDNFIEATH-S-DTTDQLAVQVAG  110
493  IDV-N----DTITEGAA--TFEEKVAKLRILAGDVDVDKFLATTM-A-DTNNQLAIQVRQ  108
494  YDV-G----DTWSEATP--TYTQVTASLKILGGDADVDTFLQQTY-A-DPNDLEALVLES  108
495  IDV-N----DTITEGAA--TFEEKVAKLRILAGDVDVDKFLATTM-A-DTNNQLAIQVRQ  108
496  IDV-N----DTITEGAA--TFEEKVAKLRILAGDVDVDKFLATTM-A-DTNNQLAIQVRQ  108
497  AGNDD----TITAKSPA--TFTRVSADLTTIIGDAEINGLIQATR-S-NITDQKAVQIAS  110
498  IDV-N----DTIPEGAA--TFEECVAKLRILAGDVDVDKFLATTM-A-DTNNQLAIQVRQ  108
499  IDV-N----DEVPEGAA--KFTEHVAKLRILAGDVDVDKFLSSTM-G-DTNSQMAIQIRS  108
500  AGVGS----TITAKNPA--TFTQVTSTLTTIIGDAEVNGLIQATR-SGDGNDQTAVQVAS  111
```

```
              190       200       210       220       230       240
1    ATRQLLYKFSEQLESGTNG-----FLGFTQL--------IQP----------SNVTDLN-  144
2    ATRQLLYKFSEQLESGTNG-----FLGFTQL--------IQP----------SNVTDLN-  144
3    ATRQLLYKFSEQLESGTNG-----FLGFTQL--------IQP----------SNVTDLN-  144
4    ATRQLLYKFSEQLESGTNG-----FLGFTQL--------IQP----------SNITDLN-  144
5    ATRQLLYKFSEQLESGTNG-----FLGFTQL--------IKP----------SNVTDLN-  144
6    ATRQLLYKFSEQLESGTNG-----FLGFTQL--------IQP----------SNVTDLN-  144
7    ATRQLLYKFSEQLESGTNG-----FLGFTQL--------IKP----------SNVTNLN-  144
8    ATRQLLYKFSEQLESGSNG-----FLGFTQL--------IKP----------SNVTDLN-  144
9    ATRQLLYKFSEQLESGTNG-----FLGFTQL--------IKP----------SNVTNLN-  144
10   ATRQLLYKFSEQLESGTKG-----FLGFTQL--------VQP----------SNITDLN-  144
11   ATRQLLYKFSEQLESGTNG-----FLGFTQL--------IKP----------SNVVDLN-  144
12   ATRQLLYKFSEQLESGTNG-----FLGFTQL--------IKS----------SNVTDLN-  144
13   ATRQLLYKFSEQLESGSNG-----FLGFKQL--------IKP----------SNVVDVN-  144
14   AIRQLLYRFSKELQNGSDG-----FLGFKQL--------ISL----------ENSVDLR-  144
15   AVRELLYKFWTLFESGDSSVNPDEFDGLLKL--------VDP----------SKVVDLG-  152
16   AVRELLYKFWILFERGDASANPEEFDGLLSL--------VNP----------GNTIDLQ-  152
17   AVRELLYRFWILFERGDASTNPEEFDGLLSL--------VNP----------GNTIDLQ-  152
18   AVRELLYKFWVLFESGDTLVNPDEFDGLLRL--------VDP----------SNTIDLQ-  152
19   AIRELLYKFWTLFESGDSA-NPGEFDGLQKL--------VDP----------SRVLDLG-  151
```

20	AVRELLYKFWMLFEGGDSATNPDEFDGLLKL--------VDP----------SKVVDLE-	153
21	AIRELLYKFWTLFESGDSV-NAGEFDGLHKL--------ADP----------SRVLDLG-	151
22	AIRELLYKFWILFESGDSA-NAGEFDGLHKI--------VDP----------SRVLDLG-	151
23	AIRELLYKFWTLFESGDSV-NAGEFDGLHKL--------ADP----------SRVLDLG-	152
24	AIRELLYKFWTLFESGDSA-NTGEFDGLYKL--------VDP----------SRVLDLG-	151
25	AIRELLYKFWMLFESGDSA-NPGEFDGLQKL--------VDP----------SRILDLG-	151
26	AIRELLYKFWTLFESGDSA-NTGEFDGLHRL--------VDS----------SRVLDLG-	151
27	AVRELLYRFWILFERGDTSTNPEEFDGLLRL--------VNP----------DNTIDLQ-	152
28	AIRELLYKFWTLFESGDSA-NTGEFDGLYKL--------VDP----------SRVLDLG-	151
29	AVQRLVYRYFRQLGLGATGS--FNFPSLQDV--------VGT----------GQTIDMG-	151
30	AVQRLVYRYFRQLGLGATGS--FNFPSLQDV--------VGT----------GQTIDMG-	157
31	AQMRLVYRYFRRLGQAAAGT--FDFPTLQDV--------VGT----------GQTINMG-	153
32	AQMRLIYRYFRRLGQAAAGT--FDFPTLQDV--------VGT----------GQTIDMG-	153
33	AQMRLIYRYFRRLGQAAAGT--FDFPTLQDV--------VGT----------GQTIDMG-	153
34	AIRRLMYMYFRKLDLGTTGGN-GDFPSLADM--------AVL----------GNVVTSA-	159
35	ACKRLMYMYARKLDLNNGGG-SGDFDSLYDM--------CAA----------GQKVAGG-	170
36	ATRQLLYKFSEQLESGSNG-----FLGFTQL--------IKP----------SNVTDLN-	67
37	AIRRLTYMFYRKLDLATTGNP-GDFPSLSDM--------CAA----------GQRFNSA-	159
38	AKIRLLYRYFQRLGETTGAV--TTFPGLTGM--------VGT----------GQTIAMG-	151
39	ANRRLLYAFCRLLEVGDTTANPNEFDGFLKL--------VNP----------ANVVDKA-	149
40	AIRRLMYMYFRKLDLDNAGTKVGDFDSLGEL--------AT-------------NITTA-	156
41	AIQRLLYKFFNLLEIGNPAINPNEFAGLKKL--------VDP----------FNVVNLG-	149
42	ADMRLMYRYFQRLDEVTGGI--SQFPGLQDM--------VGA----------GKTINMG-	151
43	AKIRLLYGYFRRLGEATGGI--SQFPGLQDM--------VGA----------GKTINMA-	150
44	ADMRLMYRYFQRLGQATGGV--SQFPGLQDI--------VGA----------GKTINMG-	151
45	AKIRLLYRYFQRLGETTGVV--TTFPSLTGM--------VGA----------GQTIAMG-	151
46	AKRRILYAYAQQLDSF--GS--AVGPGLPDL--------CAA----------SQLVDMG-	143
47	AKRRILYAYAARLGAAT-GN--PAQGGLRDL--------ADP----------ARVINLA-	144
48	AKRRILYAYAQQLDSF--GS--AVGPGLPDL--------CAA----------GQLVDMA-	143
49	AKRRILYAYAQQLDSF--GS--AVGPGLPDL--------CAA----------GQLVDMA-	143
50	AKRRILYAYAQQLDSF--GS--AVGPGLPDL--------CAA----------GQLVDMA-	143
51	AKRQVLYAYAQWIV--------NTATGLVTL--------CDA----------SRIVDMA-	139
52	AKIKLDYGYYAHLDVAT-GD--PVFGGLQDI--------VAP----------GNVINVG-	144
53	AVKRLYYKFWETFNTGDNSANPQEFDGLRRL--------VAS----------GQTVTARD	154
54	ACKRLMYMYFRKLDLDNGNTLSGDFDSLFDIANDVAPTMSVG----------ANVVDMG-	151
55	AIRRMMYQFWATFNTGNEGANPQEFNGLQQL--------VTG----------GQTITANA	154
56	AVQRLIYRYFRQLGLAATGS--FNFPSLQDV--------VGA----------GQTIDMG-	151
57	ALAELIYGYFAWLD----GT--PGTGGLRDY--------IAA----------ARIVDMG-	142
58	ACTRLLLMSFRKLDKVNGGT-EGDYDSLYDM--------CDA----------NQIVAGG-	154
59	AIKRLYYKFWGTFNTGNSSLNPEEFDGLRQL--------VSS----------GQTLTARD	154
60	AEVQNWYGFYARMDVLT-GN--PSLGGLPDL--------VAP----------SRTLNLG-	144
61	AIRRMLYQFWTTLNTGNEGTNPQEFNGLRQL--------VVA----------GQTITANA	154
62	AKRRILYSYAAQLDSA--GV--LAAPGLPDL--------CAA----------GQLVDMG-	143
63	AEIKCDYGYYARMDTLT-GD--PVHGGLLDL--------VAP----------GNIINPG-	144
64	AVKRLYYKFWETFNTGDNSVNVQEFDGLRRL--------VVS----------GQTLTARD	154
65	AVRRMTYQFWTAFNTGNEGTNPQEFNGLRQL--------VVA----------GQTVTANG	154
66	AKRQILYAYAEHIV--------SPTIGLPAA--------CDS----------DRNVDMG-	139
67	MIRQLLYGFSERLDAAP-SP--ANPLALRNY--------IEP----------DRIVNLA-	144
68	ACRQLLYGFFRLLFIGN-PVNPGEFIGFDNI--------ITGA------AFAGQIVDAL-	152
69	ATRQVLYGFFRLLFVGN-PANPGEFIGFDNI--------ITGA------PFAGQIVDAL-	168
70	ACRQCMYEGFRLLESGISGND-GEFAGLRSL--------VPA----------NMTVTVG-	154
71	ACRQCMYQGFRLLESGISGND-GEFAGLRSL--------VPA----------NMTVTVG-	205
72	ACRQCMYQGFRLLESGISGND-GEFAGLRSL--------VPA----------NMTVTVG-	200
73	ACRQCMYDGFRLLENGIAGND-GEFAGLRSL--------VPA----------NMTVTVG-	154
74	KAKALGRKFKRTLATGDRSVNSKAFDGIAKL--------VAA----------GQTISAGT	150
75	AKRRILYSYAQRLGTAT-GN--PAQAGLRDL--------ADP----------ARVIDLA-	144
76	AVQRLVYRYFRQLGLGATGS--FNFPSLQDV--------VGT----------GQTIDMG-	86
77	GEIENWYGYYGRMDTAT-GD--PVFGGLLDL--------VAP----------GRIVNMG-	144
78	KAKAMGRKFRRTLAIGDSGSNAKEFDGIASL--------TPA----------GQTLAAAT	150
79	AVQRLIYRYFRQLGLAATGS--FNFPSLQDV--------VGA----------GQTIDMG-	151
80	KAKGIARKFRRTLANGNASVNAKEFDGVHRL--------VTA----------NQTISAGT	150
81	KAKGLARKFRRTLAIGDNGTNAKEFDGVASL--------VTG----------GQTITAGA	151
82	KAKGMARQFHRTLAMGDSSKNRKEFDGLASL--------TPA----------AQTLVAGK	150
83	KAKGLGRKFKRTIVNGDSAVNAKSFDGIKKL--------TPA----------AQTLVAGA	150
84	KAKGLGRKFKRTIVNGDSSVNAKSFDGIKKL--------TPA----------AQTLIAGT	150
85	KAKGLGRKFKRTIVNGDSSVNAKSFDGIKKL--------TPA----------SQTLIAGT	150
86	KAKGLGRKFKRTIVNGDSSVNAKSFDGIKKL--------TPS----------AQTLIAGT	150

```
87   AERRLQQLFWELIDTGVAGS--GSFLGLSGL--------LPA----------GQKVVVG-  145
88   ACKRLMYMYFRKLDLVNGNTLSGDFDSLFDIAGDV----NSG----------SQIIDMS-  102
89   KAKALGRKFKRTIVNGDSSVNAKSFDGVKKL--------TPA----------SQTLIAGT  150
90   KSKALGRKFRRALVQGDSSVNAKEFDGIRAL--------TPA----------AQTLVAGA  150
91   AKRRLLYAYATFIAD----S--NQSGSLVGI--------CDA----------ARVVNMA-  100
92   AERRLQQLFWELIDTGVSGS--GSFLGLSGL--------LPA----------GQRVVVG-  145
93   AERRLQQLFWELIDTGVAGS--GRFLGLAGL--------LPA----------GQRVVVG-  145
94   KAKGITRKFRRSLAQGDSSVNAKEFDGIDRL--------VYN----------DQVIVGG-  149
95   KAKGLGRKFKRTIVNGDSAVNPKSFDGIKKL--------TPA----------AQTLVAGT  150
96   KAKGVAREFHRTLMLGDSATNAKEFDGLPKL--------VSG----------SQTISAGA  150
97   KAKGIGRQFRRTLVNGDSSVNRKEFDGVKKL--------VPA----------KQTLIAGK  150
98   KAKGMARTFRRAVVQGNSVTDPKSFDGIEKL--------VADT---------GNFFEAGL  151
99   KAKALGRQFRRTLVNGDSAVNRKEFDGIKKL--------VPA----------KQTLVAGL  150
100  KAKGMATTFRRALVVGDSTVNPKQFDGLEKL--------VTDT---------GNFIEAGV  151
101  KAKGMARKFRRTLAIGDATSNPKEFNGVAKL--------VTA----------GQTITAGT  151
102  AVRNCMNGFFYLILADAAGG-TGSPPTLVSL-------CTGT----------SQMNDFN-  153
103  KAKGMARTFRRAVVQGDSTVDNKSFDGIEKL--------VKDT---------GNFFEAGV  151
104  KAKGVGRKFHKTLATGDASVNPKEFDGIAKL--------VTE----------EQTISAGP  150
105  KAKGVAREFHRTLARGDSKTNAKEFDGFDKL--------VTS----------TQIVDAGA  150
106  KAKGVAREFHRTLARGDSKTNAKEFDGFDKL--------VTS----------TQIVDAGA  148
107  KAKSLAMTFHKTLAQGDASANPKAFDGLPKM--------VTP----------TQTVDVG-  149
108  KAKGVAREFHRTLARGDSKTNAKEFDGFDKL--------VTS----------TQIVDAGA  150
109  KAKGVAREFHRTLARGDSKTNAKEFDGFDKL--------VTS----------TQIVDAGA  150
110  KAKGVAREFHRTLARGDSKTNAKEFDGFDKL--------VTS----------TQIVDAGA  150
111  KSKALNRLFKQTLVSGDATANPKQFDGLSKL--------VTA----------GQTMDAAP  150
112  KAKGVAREFHRTLARGDSKTNAKEFDGFDKL--------VTS----------TQIVDAGA  152
113  KAKGVAREFHRTLARGDSKTNAKEFDGFDKL--------VTS----------TQIVDAGA  150
114  KAKGVAREFHRTLARGDSKTNAKEFDGFDKL--------VTS----------TQIVDAGA  150
115  KAKGLATTFRRALIQGDSAVNPKSFDGLNKL--------VNDT---------GNYFEAGP  151
116  KAKGMSRQYRAALVEGDSTTNAKQFDGLKKL--------VTT----------TQTLDAK-  149
117  KAKGVGRKFHKTLATGDASVNPKEFDGIAKL--------VTE----------EQTISAGP  150
118  KAKGVGRKFHKTLATGDASVNPKEFDGIAKL--------VTE----------EQTISAGP  150
119  KAKGMSRQYRAALVEGDSATNAKQFDGLKKL--------VTT----------AQTLDAK-  149
120  KAKSMARAFQKSVVNGDSSVNEKEFDGLKAL--------VTA----------EQTIEAGV  150
121  KAKGVAREFHRTLARGDSKTNAKEFDGFDKL--------VTS----------TQIVDAGA  150
122  KAKGVAREFHRTLARGDSKTNAKEFDGFDKL--------VTS----------TQIVDAGA  150
123  KAKGVGRKFHQTLATGDATANTKAFDGLPKL--------VDA----------SQTITAGD  150
124  KAKGVAREFHRTLARGDSKTNAKEFDGFDKL--------VTS----------TQIVDAGA  150
125  KAKGVAREFHRTLARGDSKTNAKEFDGFDKL--------VTS----------TQIVDAGA  150
126  KAKGVAREFHRTLARGDSKTNAKEFDGFDKL--------VTS----------TQIVDAGA  150
127  KAKALGMQFRRTLVNGDSTKNRKEFDGVKML--------VPA----------KQTLIAGA  150
128  KAKALGMQFRRTLVNGDTTKNRKEFDGVKAL--------VPA----------KQTLIAGA  150
129  KAKAVGRIFKKTIAQGNASKNPKEFDGLPQL--------VTS----------AMTLDADT  150
130  KAKAVGRIFKKTIAQGNAAKNPKEFDGLPQL--------VTS----------AMTLDADT  150
131  KAKAVGRIFKKTIAQGNAAKNPKEFDGLPQL--------VTS----------AMTLDADA  150
132  KAKGVAREFHRTLARGDSKTNAKEFDGFDKL--------VTS----------TQIVDAGA  150
133  KTKTASRKYQDMLINGTGAGN--EFLGLLGL--------VPS----------AQKVPTGA  150
134  KAKAVGRIFKKTIAQGNASKNPKEFDGLPQL--------VTS----------AMTLDADT  150
135  KAKGVAREFHRTLARGDSKTNAKEFDGFDKL--------VTS----------TQIVDAGA  150
136  KAKAVGREFRKVLAEGDHDTNAKQFDGISKL--------VTT----------TQKIDAG-  149
137  KAKGMARKFQRTLATGNSGTDPKSFDGISEL--------CAD----------NQVLPTSG  150
138  KAKAVAREFHRTLAKGNATANPKEFDGLPQL--------AAAA-------PNSQQVVAGA  153
139  KAKALTRKLKRTLVNGNNAVNAKEFDGLKVL--------TPA----------EQTLIAGT  150
140  KVKGLARSFKRALVLGDETSNSKSFDGLGRL--------TAS----------DQIIDAA-  149
141  KAKGLRTKFQKTLINGDRSVHSKEFDGIAAI--------SNG-------LGSSQVQVVGA  153
142  KAKGMSLQFRSELVNGDTAVNAKSFDGLRKL--------TPA----------GQTLGAGT  150
143  KVKGLARSFKRALVLGDETSNSKSFDGLGRL--------TAS----------EQIIDAA-  149
144  KAKAINRTFRNTLVNGDSAVNAKAFDGVKKL--------TPA----------GQTLVAGA  150
145  KAKALGRALRRTIINGDNSVNAKEFDGLKKL--------MPA----------NQTLVAGN  150
146  KAKGMSRKFQRTLATGNSGTDPKSFDGIAEL--------CAD----------NQVISADG  150
147  KVKGLARAFRRNLILGDSTTNSKAFDGIPKL--------MHK----------DQKVDIA-  149
148  KVKGLARAFRRNLILGDSTANSKAFDGIPKL--------MHN----------DQKIDIA-  149
149  KVKGLARAFRRNLILGDSTTNSKAFDGIPKL--------MHK----------DQKVDIA-  149
150  KVKGLARAFRRNLILGDSTTNSKAFDGIPKL--------MHD----------DQKIDIA-  149
151  KVKGLARAFRRNLILGNSETNTKAFDGIPRL--------MHK----------DQKIDIA-  149
152  KVKGLARAFRRNLILGDSTTNSKAFDGIPKL--------MHD----------DQKIDIA-  149
153  KAKAINRTFRNTLVNGDSAVNAKAFDGVKKL--------TPA----------GQTLVAGA  150
```

```
154 KVKGLARAFRRNLILGDSTTNTKAFDGIPKL---------MID----------DQKIDIA- 149
155 KVKGLARAFRRNLILGNSETNTKAFDGIPRL---------MHK----------DQNIDIA- 149
156 KVKGLARAFRRNLILGDSTTNSKAFDGIPKL---------MHD----------DQKIDIA- 149
157 KVKGLARAFRRNLILGDSTTNSKAFDGIPKL---------MHD----------DQKIDIA- 149
158 KVKGLARAFRRNLILGNSETNTKAFDGIPRL---------MHK----------DQKIDIA- 149
159 KVKGLARAFRRNLILGNSETNTKAFDGIPRL---------MHK----------DQKIDIA- 149
160 KVKGLARAFRRNLILGNSETNTKAFDGIPRL---------MHK----------DQNIDIA- 149
161 KAKGVGRKFHKTLATGDASVNPKEFDGIAKL---------VTE----------EQTISAGP 150
162 KAKGLARKFKRTLVVGDATSSPKEFDGLGKI---------AEA----ITAGDASRTVVAGT 156
163 KVKGLARAFRRNLILGNSETNTKAFDGIPRL---------MHK----------DQKIDIA- 149
164 KVKGLARAFRRNLILGDSTTNSKAFDGIPKL---------MHD----------DQKIDIA- 149
165 KVKGLARAFRRNLILGDSSANSKAFDGIPKL---------MHN----------DQKIDIA- 149
166 KVKGLARAFRRNLILGDSTTNSKAFDGIPKL---------MHD----------DQKIDIA- 149
167 KVKGLARAFRRNLILGDSTTNSKAFDGIPKL---------MHK----------DQNVDIA- 149
168 KVKGLARAFRRNLILGDSTTNSKAFDGIPKL---------MHN----------DQKIDIA- 149
169 KVKGLARAFRRNLILGDSTTNSKAFDGIPKL---------MHK----------DQNVDIA- 149
170 KVKGLARAFRRNLILGDSSSNNKAFDGIPRL---------IHA----------DQKIDIA- 149
171 KVKGLARAFRRNLILGDSTANSKAFDGIPKL---------MHN----------DQKIDIA- 149
172 KVKGLARAFRRNLILGNSETNTKAFDGIPRL---------MHK----------DQNIDIA- 149
173 KVKGLARAFRRNLILGDSSSNNKAFDGIPRL---------MHA----------DQKIDIA- 149
174 KVKGLARAFRRNLIVGDSSSNNKAFDGIPRL---------MHA----------DQKIDIA- 149
175 KVKGLARAFRRNLILGDSSSNNKAFDGIPRL---------MHA----------DQKIDIA- 149
176 KVKGLARAFRRNLILGDSTTNSKAFDGIPKL---------MHK----------DQKVDIA- 149
177 KVKGLARAFRRNLILGNSETNTKAFDGIPRL---------MHK----------DQKIDIA- 149
178 KVKGLARAFRRNLILGNSETNTKAFDGIPRL---------MHK----------DQKIDIA- 149
179 KVKGLARAFRRNLILGDSSSNNKAFDGIPRL---------MHA----------DQKIDIA- 149
180 KVKGLARAFRRNLILGDSTTNSKAFDGIPKL---------MHK----------DQNVDIA- 149
181 KVKGLARAFRRNLIVGDSSSNNKAFDGIPRL---------MHA----------DQKIDIA- 149
182 KVKGLARAFRRNLILGDSSSNNKAFDGIPRL---------MHA----------DQKIDIA- 149
183 KVKGLARAFRRNLILGDSSSNNKAFDGIPRL---------MHA----------DQKIDIA- 149
184 KVKGLARAFRRNLILGDSSSNNKAFDGIPRL---------MHA----------DQKIDIA- 149
185 KVKGLARAFRRNLILGNSETNTKAFDGIPRL---------MHK----------DQKIDIA- 149
186 KVKGLARAFRRNLIVGDSSSNNKAFDGIPRL---------MHA----------DQKIDIA- 149
187 KVKGLARAFRRNLILGDSSSNNKAFDGIPRL---------MHA----------DQKIDIA- 149
188 KVKGLARAFRRNLILGDSSSNKKAFDGIPRL---------MHA----------DQKIDIA- 149
189 KVKGLARAFRRNLILGDSSSNTKAFDGIPRL---------MHA----------DQKIDIA- 149
190 KAKAVAHKFMETFYYGDTAEDENAFDGLAKL---------VDA----------GQEVSMGT 150
191 KVKGLARAFRRNLILGNSETNTKAFDGIPRL---------MHK----------DQKIDIA- 149
192 KAKGLGIKFRRTLVNGDSSQNKKSFDGIKTL---------TPA----------GQTLVAGA 150
193 KVKGLARAFRRNLILGDSSSNNKAFDGIPRL---------MHA----------DQKIDIA- 149
194 KVKGLARAFRRNLILGDSSTNNKAFDGIPRL---------MHA----------DQKIDIA- 149
195 KVKGLARAFRRNLILGDSSSNNKAFDGIPRL---------MHA----------DQKIDIA- 149
196 KVKGLARAFRRNLILGDSISNNKAFDGIPRL---------MHA----------DQKIDIA- 149
197 KVKGLARAFRRNLILGDSSSNNKAFDGIPRL---------MHA----------DQKIDIA- 149
198 KVKGLARAFRRNLILGDSSSNSKAFDGIPRL---------MHA----------DQKIDIA- 149
199 KVKGLARAFRRNLILGDSSSNNKAFDGIPRL---------MHA----------DQKIDIA- 149
200 KVKGLARAFRRNLIVGDSSSNNKAFDGIPRL---------MHA----------DQKIDIA- 149
201 KVKGLARAFRRNLILGDSSSNNKAFDGIPRL---------MHA----------DQKIDIA- 149
202 KAKSINRVFRNAIVNGDSDVNAKSFDGIKKL---------TPA----------SQTLVAGA 150
203 KVKGLARAFRRNLIVGDSSSNNKAFDGIPRL---------MHA----------DQKIDIA- 149
204 KAKALAYAFQQTVATGDSGSNAKQFDGLAVL---------NDN---------TNQVVSAGT 151
205 KVKGLARAFRRNLILGDSSSNNKAFDGIPRL---------MHA----------DQKIDIA- 149
206 KVKGLARAFRRNLILGDSSSNNKAFDGIPRL---------MHA----------DQKIDIA- 149
207 KVKGLARAFRRNLILGDSSSNNKAFDGIPRL---------MHA----------DQKIDIA- 149
208 KVKGLARAFRRNLILGDSSSNNKAFDGIPRL---------MHA----------DQKIDIA- 149
209 KVKGLARAFRRNLILGDSSSNNKAFDGITRL---------MHA----------DQKIDIA- 149
210 KVKGLARAFRRNLILGDSSSNNKAFDGIPRL---------MHA----------DQKIDIA- 149
211 KVKGLARAFRRNLILGDSSTNNKAFDGIPRL---------MHA----------DQKIDIA- 149
212 KVKGLARAFRRNLILGDSSTNNKAFDGIPRL---------MHA----------DQKIDIA- 149
213 KVKGLARAFRRNLILGDSSSNNKAFDGIPRL---------MHA----------DQKIDIA- 149
214 KVKGLARAFRRNLILGNSETNTKAFDGIPRL---------MHK----------DQKIDIA- 149
215 KVKGLARAFRRNLILGDSSSNNKAFDGIPRL---------MHA----------DQKIDIA- 149
216 KVKGLARAFRRNLIVGDSTTNNKAFDGIPRL---------MHA----------DQKIDIA- 149
217 KVKGLARAFRRNLILGDSSSNNKAFDGIPRL---------MHA----------DQKIDIA- 149
218 KVKGLARAFRRNLILGDSSSNNKAFDGIPRL---------MHA----------DQKIDIA- 149
219 KVKGLARAFRRNLILGDSSSNTKAFDGIPRL---------MHA----------DQKIDIA- 149
220 KVKGLARAFRRNLILGDSSSNSKAFDGIPRL---------MHT----------DQKIDIA- 149
```

```
221 KVKGLARAFRRNLILGDSSSNNKAFDGIPRL--------MHA----------DQKIDIA- 149
222 KVKGLARAFRRNLILGDSSSNNKAFDGIPRL--------MHE----------DQKIDIA- 149
223 KVKGLARAFRRNLILGDSSSNNKAFDGIPRL--------MHA----------DQKIDIA- 149
224 KVKGLARAFRRNLIVGDSTTNNKAFDGIPRL--------MHA----------DQKIDIA- 149
225 KVKGLARAFRRNLILGDSSSNNKAFDGIPRL--------MHA----------DQKIDIA- 149
226 KVKGLARAFRRNLILGDSSSNNKAFDGIPRL--------MHA----------DQKIDIA- 149
227 KVKGLARAFRRNLILGDSNTNNKAFDGIPRL--------MHA----------DQKIDIA- 149
228 KAKGMGREFSKVLIQGDSAVNAKQFDGVAKL--------VTS----------DQTIDGK- 149
229 KVKGLARAFRRNLILGDSSTNNKAFDGIPRL--------MHA----------DQKIDIA- 149
230 KAKGMGREFSKVLIQGDSAVNAKQFDGVAKL--------VTS----------DQTIDGK- 149
231 KVKGLARAFRRNLILGDSSSNNKAFDGIPRL--------MHA----------DQKIDIA- 149
232 KVKGLARAFRRNLILGNSETNTKAFDGIPRL--------MHK----------DQKIDIA- 149
233 KVKGLARAFRRNLILGDSSSNTKAFDGIPRL--------MHT----------DQKIDIA- 149
234 KVKGLARAFRRNLILGDSSSNNKAFDGIPRL--------MHA----------DQKIDIA- 149
235 KVKGLARAFRRNLIVGDSTTNNKAFDGIPRL--------MHA----------DQKIDIA- 149
236 KVKGLARAFRRNLILGDSSSNTKAFDGIPRL--------MHA----------DQKIDIA- 149
237 KVKGLARAFRRNLILGDSSSNNKAFDGIPRL--------MHA----------DQKIDIA- 149
238 KVKGLARAFRRNLIVGDSSSNNKAFDGIPRL--------MHA----------DQKIDIA- 149
239 KAKAVAHAFLQAFYRGNSVVNPKQFDGLRKL--------VPA----------SQTIQPGP 150
240 KVKGLARAFRRNLILGNSETNTKAFDGIPRL--------MHK----------DQKIDIA- 149
241 KVKGLARAFRRNLILGDSSTNTKAFDGIPKL--------MHD----------DQKIDIE- 149
242 KVKGLARAFRRNLILGDSSTNNKAFDGIPRL--------MHA----------DQKIDIA- 149
243 KVKGLARAFRRNLIVGDSSSNNKAFDGIPRL--------MHA----------DQKIDIA- 149
244 KVKGLARAFRRNLILGDSSSNNKAFDGIPRL--------MHA----------DQKIDIA- 149
245 KVKGLARAFRRNLILGDSSTNNKAFDGIPRL--------MHA----------DQKIDIA- 149
246 KVKGLARAFRRNLILGDSSSNNKAFDGIPRL--------MHA----------DQKIDIA- 149
247 KVKGLARAFRRNLILGDSSSNTKAFDGIPRL--------MHA----------DQKIDIA- 149
248 KVKGLARAFRRNLILGDSSSNNKAFDGIPRL--------MHA----------DQKIDIA- 149
249 KVKGLARAFRRNLILGDSSSNNKAFDGIPRL--------MHA----------DQKIDIA- 149
250 KVKGLARAFRRNLILGDSSSNNKAFDGIPRL--------MHA----------DQKIDIA- 149
251 KVKGLARAFRRNLILGDSSSNSKAFDGIPRL--------MHE----------DQKIDIA- 149
252 KVKGLARAFRRNLILGDSSSNNKAFDGIPRL--------MHA----------DQKIDIA- 149
253 KVKGLARAFRRNLILGDSSSNNKAFDGIPRL--------MHA----------DQKIDIA- 149
254 KVKGLARAFRRNLILGDSSSNNKAFDGIPRL--------MHA----------DQKIDIA- 149
255 KVKGLARAFRRNLILGDSSSNNKAFDGIPRL--------MHA----------DQKIDIA- 149
256 KVKGLARAFRRNLILGNSSTNTKAFDGIPKL--------MHD----------DQKIDIE- 149
257 KVKGLARAFRRNLILGDSSTNTKAFDGIPKL--------MHD----------DQKIDIE- 149
258 KVKGLARAFRRNLILGDSSSNNKAFDGIPRL--------MHA----------DQKIDIA- 149
259 KVKGLARAFRRNLILGDSSTNNKAFDGIPRL--------MHA----------DQKIDIA- 149
260 KVKGLARAFRRNLIVGDSTTNNKAFDGIPKL--------MHD----------DQKIDIA- 149
261 KVKGLARAFRRNLIVGDSSTNNKAFDGIPKL--------MHD----------DQKIDIS- 149
262 KVKGLARAFRRNLILGDSSSNNKAFDGIPRL--------MHA----------DQKIDIA- 149
263 KVKGLARAFRRNLILGNSTENTKAFDGIPKL--------MHD----------DQKIDIA- 149
264 KVKGLARAFRRNLILGDSSTNNKAFDGIPKL--------MHD----------DQKIDIA- 149
265 KVKGLARAFRRNLIVGDSITNNKAFDGIPKL--------MHD----------DQKIDIS- 149
266 KVKGLARAFRRNLILGDSSTNNKAFDGIPRL--------MHA----------DQKIDIA- 149
267 KVKGLARAFRRNLIVGDSTTNNKAFDGIPKL--------MHD----------DQKIDIS- 149
268 KVKGLARAFRRNLIVGDSTTNNKAFDGIPKL--------MHD----------DQKIDIS- 149
269 KVKGLARAFRRNLILGDSSTNTKAFDGIPKL--------MHD----------DQKIDIE- 149
270 KAKGMGREFSKVLIQGNSTTNAKQFDGIAKL--------VTS----------DQTIDGK- 149
271 KVKGLARAFRRNLILGDSSTNNKAFDGIPRL--------MHA----------DQKIDIA- 149
272 KVKGLARAFRRNLIVGDSTTNNKAFDGIPKL--------MHD----------DQKIDIS- 149
273 KVKGLARAFRRNLIVGDSTTNSKAFDGIPKL--------MHD----------DQKIDIS- 149
274 KVKGLARAFRRNLIVGDSTTNNKAFDGIPKL--------MHD----------DQMIDIS- 149
275 KVKGLARAFRRNLILGDSSSNSKAFDGIPRL--------MHT----------DQKIDIA- 149
276 KVKGLARAFRRNLIVGDSTTNNKAFDGIPRL--------MHA----------DQKIDIA- 149
277 KVKGLARAFRRNLILGDSSSNTKAFDGIPRL--------MHA----------DQKIDIA- 149
278 KVKGLARAFRRNLIVGDSTTNNKAFDGIPKL--------MHD----------DQKIDIS- 149
279 KVKGLARAFRRNLILGDSSSNNKAFDGIPRL--------MHA----------DQKIDIA- 149
280 KVKGLARAFRRNLIVGDSTTDNKAFDGIPRL--------MHA----------DQKIDIA- 149
281 KAKGVAREFHRTLARGDSKTNAKEFDGFDKL--------VTS----------TQIVDAGA 150
282 KVKGLARAFRRNLIVGDSTTNNKAFDGIPKL--------MHD----------DQKIDIS- 149
283 KVKGLARAFRRNLILGDFSTNNKAFDGIPRL--------MHA----------DQKIDIA- 149
284 KVKGLARAFRRNLILGDSSSNNKAFDGIPRL--------MHA----------DQKIDIA- 149
285 KVKGLARAFRRNLILGDSSTNNKAFDGIPRL--------MHA----------DQKIDIA- 149
286 KVKGLARAFRRNLILGDSSTNNKSFDGIPKL--------MHD----------DQKIDIE- 149
287 KVKGLARAFRRNLILGDSSSNNKAFDGIPRL--------MHA----------DQKIDIA- 149
```

```
288  KVKGLARAFRRNLILGDSSTNNKAFDGIPKL--------MHD----------DQKIDIE-  149
289  KVKGLARAFRRNLIVGDSSTNNKAFDGIPKL--------MHD----------DQKIDIS-  149
290  KVKGLARAFRRNLILGDSSSNTKAFDGIPRL--------MHT----------DQKIDIA-  149
291  KVKGLARAFRRNLILGDSSTNNKAFDGIPRL--------MHA----------DQKIDIA-  149
292  KVKGLARAFRRNLILGDSSSNNKAFDGIPRL--------MHA----------DQKIDIA-  149
293  KVKGLARAFRRNLIVGDSTTNNKAFDGIPKL--------MHD----------DQKIDIS-  149
294  KVKGLARAFRRNLIVGDSSSNNKAFDGIPRL--------MHE----------DQKIDIA-  149
295  KVKGLARAFRRNLIVGDSTTNNKAFDGIPKL--------MHD----------DQKIDIS-  149
296  KVKGLARAFRRNLIVGDSTTNSKAFDGIPKL--------MHD----------DQKIDIS-  149
297  KVKGLARAFRRNLIVGDSTTNNKAFDGIPKL--------MHD----------DQKIDIS-  149
298  KVKGLARAFRRNLILGDSSSNNKAFDGIPRL--------MHE----------DQKIDIA-  149
299  KVKGLARAFRRNLIVGDSTTNNKAFDGIPKL--------MHD----------DQKIDIS-  149
300  KVKGLARAFRRNLILGDSSSNNKAFDGIPRL--------MHA----------DQKIDIA-  143
301  KVKGLARAFRRNLIVGDSTTNNKAFDGIPKL--------MHD----------DQKIDIS-  149
302  KVKGLARAFRRNLIVGDSITNNKAFDGIPKL--------MHD----------DQKIDIS-  149
303  KVKGLARAFRRNLILGDSSTNTKAFDGIPKL--------MHD----------DQKIDIE-  149
304  KVKGLARAFRRNLIVGDSTTNNKAFDGIPKL--------MHD----------DQKIDIS-  149
305  KVKGLARAFRRNLIVGDSTTNNKAFDGIPKL--------MHD----------DQKIDIS-  149
306  KVKGLARAFRRNLIVGDSTTNNKAFDGIPKL--------MHD----------DQKIDIS-  149
307  KVKGLARAFRRNLILGDSSTNNKAFDGIPKL--------MHD----------DQKIDIE-  149
308  KVKGLARAFRRNLIVGDSTTNNKAFDGIPKL--------MHD----------DQKIDIS-  149
309  KVKGLARAFRRNLILGDSSTNTKAFDGIPKL--------MHD----------DQKIDIE-  149
310  KVKGLARAFRRNLILGDSSTNNKAFDGIPKL--------MHD----------DQKIDIE-  149
311  KVKGLARAFRRNLILGDSSTNTKAFDGIPKL--------MHD----------DQKIDIE-  149
312  KVKGLARAFRRNLIVGDSTTNNKAFDGIPKL--------MHD----------DQKIDIS-  149
313  KVKGLARAFRRNLILGDSSSNNKAFDGIPRL--------MHA----------DQKIDIA-  149
314  KVKGLARAFRRNLILGDSSTNTKAFDGIPKL--------MHD----------DQKIDIE-  149
315  KVKGLARAFRRNLILGDSSSNNKAFDGIPRL--------MHT----------DQKIDIA-  149
316  KVKGLARAFRRNLIVGDSTANNKAFDGIPKL--------MHD----------DQKIDIS-  149
317  KVKGLARAFRRNLILGDSSTNNKAFDGIPKL--------MHD----------DQKIDIA-  149
318  KVKGLARAFRRNLIVGDASTNNKAFDGIPKL--------MHD----------DQKIDIS-  149
319  KVKGLARAFRRNLIVGDSTTNNKAFDGIPKL--------MHD----------DQKIDIS-  149
320  KVKGLARAFCRNLILGDSTTNTKAFDGIPKL--------MHD----------DQKIDIE-  149
321  KVKGLARAFRRNLIVGDSTTNNKAFDGIPKL--------MHD----------DQKIDIS-  149
322  KVKGLARAFRRNLILGDSSTNTKAFDGIPKL--------MHD----------DQKIDIE-  149
323  KVKGLARAFRRNLILGDSSTNNKAFDGIPKL--------MHD----------DQKIDIE-  149
324  KVKGLARAFRRNLILGDSSSNNKAFDGIPRL--------MHA----------DQKIDIA-  149
325  KVKGLARAFRRNLILDDSSTNNKAFDGIPRL--------MHA----------DQKIDIA-  149
326  KVKGLARAFRRNLIVGDSTTNNKAFDGIPKL--------MHD----------NQKIDIS-  149
327  KVKGLARAFRRNLIVGDSTTNNKAFDGIPKL--------MHD----------DQKIDIS-  149
328  KVKGLARAFRRNLIVGDSTTNNKAFDGIPKL--------MHD----------DQKIDIS-  149
329  KVKGLARAFRRNLILGDSSTNNKAFDGIPKL--------MHD----------DQKIDIE-  149
330  KVKGLARAFRRNLILGDSSTNTKAFDGIPKL--------MHD----------DQKIDIE-  149
331  KVKGLARAFRRNLIVGDSTTNNKAFDGIPKL--------MHD----------DQKIDIS-  149
332  KVKGLARAFRRNLILGDSSTNTKAFDGIPKL--------MHD----------DQKIDIE-  149
333  KVKGLARAFRRNLILGDSSTDTKAFDGIPKL--------MHD----------DQKIDIE-  149
334  KVKGLARAFRRNLILGNSTENTKAFDGIPKL--------MHN----------DQKIDIA-  149
335  KVKGLARAFRRNLILGDSSTNNKAFDGIPKL--------MHD----------DQKIDIE-  149
336  KVKGLARAFRRNLIVGDSTTNNKAFDGIPRL--------MHA----------DQKIDIA-  149
337  KVKGLARAFRRNLILGDSSTNNKAFDGIPKL--------MHD----------DQKIDIE-  149
338  KVKGLARAFRRNLIVGDSTTNNKAFDGIPRL--------MHD----------DQKIDIS-  149
339  KVKGLARAFRRNLILGDSSTNTKAFDGIPKL--------MHD----------DQKIDIE-  149
340  KVKGLARAFRRNLIVGDSTTNNKAFDGIPKL--------MHD----------DQKIDIS-  149
341  KVKGLARAFRRNLILGDSSTNTKAFDGIPKL--------MHD----------DQKIDIE-  149
342  KVKGLARAFRRNLILGNSETNTKAFDGIPRL--------MHK----------DQNIDIA-  149
343  KVKGLARAFRRNLILGDSTTNTKAFDGIPKL--------MHD----------DQKIDIE-  149
344  KVKGLARAFRRNLILGDSSTNNKAFDGIPKL--------MHD----------DQKIDIE-  149
345  KVKGLARAFRRNLIVGDSTTNNKAFDGIPKL--------MHD----------DQKIDIS-  149
346  KVKGLARAFRRNLIVGDSTTNNKAFDGIPKL--------MHD----------DQKIDIS-  149
347  KVKGLARAFRRNLILGDSSSNNKAFDGIPRL--------MHA----------DQKIDIA-  149
348  KVKGLARAFRRNLILGNSTENTKAFDGIPKL--------MHN----------DQKIDIA-  149
349  KVKGLARAFRRNLILGDSSTNTKAFDGIPKL--------MHD----------DQKIDIE-  149
350  KVKGLARAFRRNLILGDSSTNNKAFDGIPKL--------MHD----------DQKIDIE-  149
351  KAKGVAREFHRTLARGDSKTNAKEFDGFDKL--------VTS----------TQIVDAGA  131
352  KVKGLARAFRRNLILGNSTENNKAFDGIPKL--------MHD----------DQKIDIE-  149
353  KAKSVGRQYQDTMINGDGTSE--TFQGLLSL--------VAA----------GQTITGDT  150
354  KVKGLARAFRRNLIVGDSTTNNKAFDGIPKL--------MHD----------DQKIDIS-  146
```

355 KVKGLARAFRRNLILGNSTENTKAFDGIPKL--------MHN----------DQKIDIA- 149
356 KVKGLARAFRRNLILGDSSTNTKAFDGIPKL--------MHD----------DQKIDIE- 149
357 KVKGLARAFRRNLIVGDSTTNNKAFDGIPKL--------MHD----------DQKIDIS- 149
358 KVKGLARAFRRNLILGDSSTNTKAFDGIPKL--------MHD----------DQKIDIE- 149
359 KVKGLARAFRRNLIVGDSTTNNKAFDGIPKL--------MHD----------DQKIDIS- 149
360 KVKGLARAFRRNLILGDSSTNTKAFDGIPKL--------MHD----------DQKIDIE- 149
361 KVKGLARAFRRNLILGDSSSNNKAFDGIPRL--------MHA----------DQKIDIA- 149
362 KVKGLARAFRRNLIVGDSTTNNKAFDGIPKL--------MHD----------DQKIDIS- 149
363 KVKGLARAFRRNLILGDSSTNTKAFDGIPKL--------MHD----------DQKIDIE- 149
364 KVKGLARAFRRNLILGDSTTNTKAFDGIPKL--------MHD----------DQKIDIE- 149
365 KVKGLARAFRRNLILGDSSTNTKAFDGIPKL--------MHD----------DQKIDIE- 149
366 KVKGLARAFRRNLILGDSSTNTKAFDGIPKL--------MHD----------DQKIDIE- 149
367 KVKGLARAFRRNLILGNSTENTKAFDGIPKL--------MHD----------DQKIDIA- 149
368 KVKGLARAFRRNIILGNSTENTKAFDGIPKL--------MHN----------DQKIDIA- 149
369 KVKGLARAFRRNLILGDSSTNTKAFDGIPKL--------MHD----------DQKIDIE- 149
370 KVKGLARAFRRNLILGDSSTNTKAFDGIPKL--------MHD----------DQKIDIE- 149
371 KVKGLARAFRRNLILGDSSTNTKAFDGIPKL--------MHD----------DQKIDIE- 149
372 KVKGLARAFRRNLILGDSSTNNKAFDGIPKL--------MHD----------DQKIDIE- 149
373 KVKGLARAFRRNLILGDSTTNTKAFDGIPKL--------MHD----------DQKIDIE- 149
374 KVKGLARAFRRNLILGDSSSNSKAFDGIPRL--------MHE----------DQKIDIA- 149
375 KVKGLARAFRRNLILGDSSTNTKAFDGIPKL--------MHD----------DQKIDIE- 149
376 KVKGLARAFRRNLIVGDSTTNNKAFDGIPRL--------MHA----------DQKIDIA- 149
377 KAKAVAHAFLATFYTGDASADPKQFDGLRRL--------VPA----------SQTIQPGP 150
378 KVKGLARAFRRNLILGDSSTNNKAFDGIPKL--------MHD----------DQKIDIE- 149
379 KVKGLARAFRRNLILGDSSTNTKAFDGIPKL--------MHD----------DQKIDIE- 149
380 KVKGLARAFRRNLILGNSTENTKAFDGIPKL--------MNN----------DQKIDIA- 149
381 KVKGLARAFRRNLILGNSTENTKAFDGIPKL--------MHN----------DQKIDIA- 149
382 KAKSAGRNYQNQLINGSGASD--EFNGLINL--------CAS----------GQKATTGG 150
383 KVKGLARAFRRNIILGNSTENTKAFDGIPKL--------MHN----------DQKIDIA- 149
384 KVKGLARAFRRNLILGNSTENTKAFDGIPKL--------MHN----------DQKIDIA- 149
385 KVKGLARAFRRNLILGDSSTNTKAFDGIPKL--------MHD----------NQKIDIE- 149
386 KVKGLARAFRRNLIVGDSTTNNKAFDGIPRL--------MHA----------DQKIDIA- 149
387 KAKGMGRRFRRTLVQGDSAANSKEFDGLWKL--------VTA----------QQSIAAGA 151
388 KVKGLARAFRRNLILGNSTENTKAFDGIPKL--------MHN----------DQKIDIA- 149
389 KVKGLARAFRRNLIVGDSTTNNKAFDGIPKL--------MHD----------DQKIDIS- 149
390 KTKTASRKYQDMLINGTGAGD--EFLGLLGL--------VPV----------AQKVATGA 150
391 KVKGLARAFRRNLILGNSTENTKAFDGIPKL--------MHN----------DQKIDIA- 149
392 KVKGLARAFRRNLILGDSSTNTKAFDGIPKL--------MHD----------DQKIDIE- 149
393 KVKGLARAFRRNLILGDSSTNTKAFDGIPKL--------MHD----------DQKIDIE- 149
394 KVKGLARAFRRNLILGNSTENTKAFDGIPKL--------MHN----------DQKIDIA- 149
395 KVKGLARAFRRNLILGNSTENTKAFDGIPKL--------MHN----------DQKIDIA- 149
396 KVKGMARAFRRNLILGNSETNNKAFDGIPRL--------MHE----------DQKIDIA- 149
397 KAKSCGRKYQDQMVNGDGTGN--NMTGMFGL--------VAA----------GQTITANN 151
398 KVKGLARAFRRNLILGDSSSNNKAFDGIPRL--------MHA----------DQKIDIA- 149
399 KVKGLARAFRRNLILGDSSTNNKAFEGIPKL--------MHD----------DQKIDIA- 149
400 KVKGLARAFRRNLILGNSTANNKAFDGIPKL--------MHN----------DQKIDIA- 149
401 KVKGLARAFRRNLILGNSTANNKAFDGIPKL--------MHN----------DQKIDIA- 149
402 KVKGLARAFRRNLILGNSTENTKAFDGIPKL--------MRN----------DQKIDIA- 149
403 KVKGLARAFRRNLIVGDSTTNNKAFDGIPKL--------MHD----------DQKIDIS- 149
404 KVKGLARAFRRNLIVGDSTTNNKAFDGIPKL--------MHD----------DQKIDIS- 149
405 KVKGLARAFRRNLILGDSSSNNKAFDGIPRL--------MHA----------DQKIDIA- 149
406 KVKGLARAFRRNLILGNSTANTKAFDGIPKL--------MHN----------DQKIDIA- 149
407 KVKGLARAFRRNLIVGDSTTNNKAFDGIPKL--------MHD----------DQKIDIS- 149
408 KVKGLARAFRRNLILGDSSTNTKAFDGIPKL--------MHD----------DQKIDIE- 149
409 KVKGLARAFRRNLILGNSTANNKAFDGIPKL--------MHN----------DQKIDIA- 149
410 KVKGLARAFRRNLILGNSTANTKAFDGIPKL--------MHN----------DQKIDIA- 149
411 KVKGLARAFRRNLILGNSTANTKAFDGIPKL--------MHN----------DQKIDIA- 149
412 KVKGLARAFRRNLILGNSTANNKAFDGIPKL--------MHN----------DQKIDIA- 149
413 KVKGLARAFRRNLILGDSSSNNKAFDGIPRL--------MHA----------DQKIDIA- 149
414 KVKGLARAFRRNIILGNSTENTKAFDGIPKL--------MHN----------DQKIDIA- 149
415 KVKGLARAFRRNLILGNSTANTKAFDGIPKL--------MHN----------DQKIDIA- 149
416 KVKGLARAFRRNLILGNSTANTKAFDGIPKL--------MHN----------DQKIDIA- 149
417 KVKGMARAFRRNLILGNSETNNKAFDGIPRL--------MHE----------DQKIDIA- 149
418 KVKGLARAFRRNLILGDSSTNNKAFDGIPKL--------MHD----------DQKIDIE- 149
419 KAKGLGRKFRMNLVTGDTSVNSKQFDGVRKL--------VTA----------DQTLDAGV 150
420 KVKGLARAFRRNIILGNSTENTKAFDGIPKL--------MHN----------DQKIDIA- 149
421 KVKGLARAFRRNLILGDSSTNNKAFDGIPRL--------MHA----------DQKIDIA- 149

```
422  KVKGLARAFRRNLILGNSETNTKAFDGIPRL--------MHK----------DQNIDIA-  149
423  KVKGMARAFRRNLILGNSEANNKAFDGIPRL--------MHE----------DQKIDIA-  149
424  KVKGLARAFRRNLILGNSTENTKAFDGIPKL--------MHN----------DQKIDIA-  149
425  KVKGLARAFRRNLILGNSETNTKAFDGIPRL--------MHK----------DQKIDIA-  149
426  KVKGLARAFRRNLILGNSETNTKAFDGIPRL--------MHK----------DQKIDIA-  149
427  KVKGLARAFRRNLILGNSETNTKAFDGIPRL--------MHK----------DQKIDIA-  149
428  KVKGLARAFRRNLILGNSTENTKAFDGIPKL--------MHN----------DQKIDIA-  149
429  KVKGLARAFRRNLILGDSSTNNKAFDGIPRL--------MHA----------DQKIDIA-  149
430  KVKGLARAFRRNLILGNSETNTKAFDGIPRL--------MHK----------DQKIDIA-  149
431  KVKGLARAFRRNLILGNSTANNKAFDGIPKL--------MHN----------DQKIDIA-  149
432  KAKSAGRQYQTMLATGDGTGN--TFAGLPTL--------VDA----------SQKVVTGT  150
433  KVKGLARAFRRNLILGDSSSNNKAFDGIPRL--------MHA----------DQKIDIA-  149
434  KVKGLARAFRRNLIVGDSTTNNKAFDGIPKL--------MHD----------DQKIDIS-  149
435  KVKGLARAFRRNLIVGDSTTNNKAFDGIPKL--------MHD----------DQKIDIS-  149
436  KVKGLARAFRRNIILGNSTENTKAFDGIPKL--------MHN----------DQKIDIA-  149
437  KVKGLARAFRRNLILGDSGANSKSFDGIPLL--------MHA----------DQKIDIA-  149
438  KVKGLARAFRRNIILGNSTENTKAFDGIPKL--------MHN----------DQKIDIA-  149
439  KAKGMGREFSKVLIQGNSTTNAKQFDGIAKL--------VTS----------DQTIDGK-  149
440  KVKGLARAFRRNLILGDSSSNNKAFDGIPRL--------MHA----------DQKIDIA-  149
441  KAKAVAHAFLATFYTGDASADPKQFDGLRRL--------VPA----------SQTFQPGP  150
442  KVKGLARSFRRSLILGDSTTNAKSFDGIPKL--------IHA----------DQKI-IA-  148
443  KVKGLARAFRRNLILGDSSTNTKAFDGIPKL--------MHD----------DQKIDIE-  149
444  KAKAVGRKYSSLLINGTGANE--EFVGLLGL--------VSS----------GQTLTAGT  150
445  KAKSAGRNYRNQLINGSGAAD--EFNGMINL--------CAT----------SQKVDTGN  150
446  KVKGLARAFRRNLILGDSSSNNKAFDGIPRL--------MHA----------DQKIDIA-  149
447  KVKGLARAFRRNLILGDSSSNNKAFDGIPRL--------MHA----------DQKIDIA-  149
448  RAKAVAYAFTESFFIGDSTANPKSFDGLRKL--------VPA----------AQTISPGA  148
449  KVKGLARAFRRNLILGDSSSNNKAFDGIPRL--------MHA----------DQKIDIA-  149
450  KVKGLARAFRRNLIVGDSTTNNKAFDGIPRL--------MHA----------DQKIDIA-  149
451  KAKAVAHAFLNAFYRGDSAVNPKQFDGLRKL--------VPA----------SQTIQPGP  150
452  RAKAVAHLFSDTFFNGDSTANPKAFDGLAKL--------VPP----------SQTLEPGP  150
453  KVKGLARAFRRNIILGNSTENTKAFDGIPKL--------MHN----------DQKIDIA-  149
454  KAKSCGRKYQDQMVNGDGTAN--NMTGLFGL--------VAA----------GQTITANN  151
455  KAKAVGRIFKKTVAQGNATANPKEFDGLPQL--------VTS----------PMTLAAGA  150
456  KVKGLARAFRRNLIVGDSTTNNKAFDGIPKL--------MHD----------DQKIDIS-  149
457  KVKGLARAFRRNLILGDSSSNNKAFDGIPRL--------MHA----------DQKIDIA-  149
458  KVKGLARAFRRNLILGNSTENTKAFDGIPKL--------MHN----------DQKIDIA-  149
459  KAKALARTFQDSVVNGDNVARPKEFDGLKAL--------TTA----------AQTITAGA  150
460  KVKGLARAFRRNLILGDSSSNNKAFDGIPRL--------MHA----------DQKIDIA-  149
461  KAKSAGRQYQSQMVNGDGTGN--NMAGLFGL--------VAG----------SQTITANN  151
462  KVKGLARAFRRNLILGDSSSNNKAFDGIPRL--------MHA----------DQKIDIA-  149
463  GEIQTWYGYYGRMDVLT-GN--PVFGGLPDL--------VAP----------ARTINMG-  58
464  KVKGLARAFRRNLILGNSETNTKAFDGIPRL--------MHK----------DQKIDIA-  149
465  KVKGLARAFRRNLILGDSSSNNKAFDGIPRL--------MHA----------DQKIDIA-  149
466  KVKGLARAFRRNLILGDSSSNNKAFDGIPRL--------MHA----------DQKIDIA-  149
467  KVKGLARAFRRNLILGDSSSNNKAFDGIPRL--------MHA----------DQKIDIA-  149
468  KVKGLARAFRRNLILGDSSTNNKAFDGIPRL--------MHA----------DQKIDIA-  149
469  KVKGLARAFRRNLILGDESVDAKAFNGIPKL--------MVP----------DQSI-VA-  148
470  KVKGLARAFRRNLILGDSSSNNKAFDGIPRL--------MHE----------DQKIDIA-  149
471  KAKSCGRKFQDMLINGTGASD--QFEGLINL--------VAA----------GQTLDNGT  151
472  KAKTVAREFHRALATGDSSVNSKVFDGLPRL--------LKQAQDFAATAGGSQIVSAGT  160
473  KAKSCGRKFQDMLINGTGAND--QFEGLINL--------VAA----------GQTLDNGT  151
474  KVKGLARAFRRNLILGDSSSNNKAFDGIPRL--------MHA----------DQKIDIA-  149
475  KVKGLARAFRRNLILGDSSSNSKAFDGIPRL--------MHE----------DQKIDIA-  149
476  KAKSAGRQYQSQMVNGDGTGN--NMAGLFGL--------VAG----------SQTITANN  151
477  KAKSLGRKFQDSLINGDGTGN--SFQGLASL--------ASA----------SQIIDVGT  150
478  KAKAVGRKYANLLLNGTGANE--EFEGLLTL--------ASA----------GQTLIAGA  150
479  KAKSAGRKYQDQLINGDGTGN--TFTGLLGL--------AAA----------GQTIGANN  151
480  KAKSCGRKYQDMLINGTGASD--EFEGIINL--------CAE----------GQKVATGD  151
481  KVKGLARAFRRNLILGDSSSNNKAFDGIPRL--------MHA----------DQKIDIA-  149
482  KVKGLARAFRRNLILGDSSSNNKAFDGIPRL--------MHA----------DQKIDIA-  149
483  KVKGLARAFRRNLILGDSATNTKSFDGIPKL--------IHK----------DQKI-IA-  148
484  KVKGLARAFRRNLILGDSSSNNKAFDGIPRL--------MHA----------DQKIDIA-  149
485  RAKAVAHLFSDTFFNGDSTANPKAFDGLATL--------VPA----------SQTLEPGP  150
486  KVKGLARAFRRSLILGNADENAKSFDGIPRL--------IHK----------DQKI-VA-  148
487  KVKGLARAFRRNLILGNSTENTKAFDGIPKL--------MHN----------DQKIDIA-  149
488  KVKGLARAFRRNLILGDSSANAKTFDGIPKL--------IHN----------DQKI-IA-  148
```

489 KVKGLARAFRRNLILGDSVTNAKSFDGIPKL--------IHN----------DQKI-VA- 148
490 KAKSAGRQYQNQMVNGDGTGN--NVAGLFSL--------VAA----------SQTITANN 151
491 KVKGLARAFRRNLILGDSSSNNKAFDGIPRL--------MHA----------DQKIDIA- 149
492 KAKAVGRKYSNLLINGTGAND--EFVGLLGL--------ASS----------GQTLTAGA 150
493 KVKGLARAFRRNLIVGDSTTNNKAFDGIPKL--------MHD----------DQKIDIS- 149
494 RAKAVAHAFSDAFYNGDSAANPKQFDGLKKL--------VSA----------AQTIAPGA 150
495 KVKGLARAFRRNLIVGDSTTNNKAFDGIPKL--------MHD----------DQKIDIS- 149
496 KVKGLARAFRRNLIVGDSTTNNKAFDGIPKL--------MHD----------DQKIDIS- 149
497 KAKSIGRQFQTTMVTGDGSGN--TFEGLMSL--------VAS----------TQSFEVGT 150
498 KVKGLARAFRRNLILGDSSSNNKAFDGIPRL--------MHA----------DQKIDIA- 149
499 KVKGLARAFRRNLILGDSSSNAKTFDGIPKL--------IHN----------DQKI-IA- 148
500 KAKSAGRQYQNQMVNGDGTGN--NVAGLFSL--------VAG----------SQTITANN 151

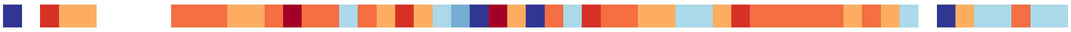

250 260 270 280 290 300

1 -----GNI----LKLEDLDRAKGQIRVKEGESGVIFTNQSGYEQIRKAYYNR--NQIP-- 191
2 -----GNT----LKLEDLDHAKGQIRVKEGESGVIFTNQSGYEQIRKAYYNR--NQIP-- 191
3 -----GNT----LKLEDLDRAKGQIRVKEGESGVIFTNQSGYEQIRKAYYNR--NQIP-- 191
4 -----GNT----LKLEDLDRAKGQIRVKEGESGVIFTNQSGYEQIRKAYYNR--NQIP-- 191
5 -----GNT----LKLEDLDRAKGQIRVKEGESGVIFTNQSGYEQIRKAYYNR--NQIP-- 191
6 -----GNT----LKLEDLDRAKGQIRVKEGESGVIFTNQSGYEQIRRAYYNR--NQIP-- 191
7 -----GNT----LKLEDLDRAKGQIRVKEGESGVIFTNQSGYEQIRKAYYNR--NQIP-- 191
8 -----GNT----LKLEDLDRAKGQIRVKEGESGVIFTNQSGYEQIRKAYYNR--DQIP-- 191
9 -----GNT----LKLEDLDRAKGQIRVKEGESGVIFTNQSGYEQIRKAYYNR--NQIP-- 191
10 -----GNT----LKLEDLDRAKGQIRVKEGESGVIFTNQSGYEQIRKAYYNR--NQIP-- 191
11 -----GNI----LNLEDLDRAKGQIRVKEGESGVIFTNQSGYEQIRKAYYNR--DQIP-- 191
12 -----GNT----LKLEDLDRAKGQIRVKEGESGVIFTNQPGYEQIRKAYYNR--NQIP-- 191
13 -----GNI----LRLEDLDRAKGQIRVKEGEYGVIFTNQSGYEQIRKAYYNR--NQIP-- 191
14 -----GAT----LTLEDLDRAKGKIRIRNGESGIIITNEPGYRLIRKAYYNR--NILP-- 191
15 -----GRE----LTLEDMDSAKEMVRTNDGRHTVIFTSSIGKRAIHAAYWNR--AVAPEY 201
16 -----GRP----LTLEDLDRAKELVRANDGRCVVIFTSSIGKRAINASHYVR--GLRPDS 201
17 -----GRP----LTLEDLDRTKELVRANDGRCVVIFTSSIGKRAINASHYVR--GLRPDS 201
18 -----GRA----LTLEDLDRARERVRANDGRCVVIFTSSIGKRAINASHYVR--GFKPDS 201
19 -----FKP----LTLEVLEQGKELVRTNDGRGTVVFTNSIGKRAIHAAHWTR--GLTPQY 200
20 -----GRE----LTLEDMDRAKEMVRTNDGRCIVIFTSTIGKRAIHAAYWNR--GVAPEY 202
21 -----FQP----LTLEVLEQGKELVRTNDGRGTVLFTNSTGKRAIHAAHWTR--GLTPQY 200
22 -----FQP----LTLETLEQGKELVRTNDGRGTVLFTNSIGKRAIHAAHWTR--GLTPQY 200
23 -----FQP----LTLEVLEQGKELVRTNDGRGTVLFTNSTGKRAIHAAHWTR--GLTPQY 201
24 -----FQP----LTLEVLEQGKELVRTNDGRGTVLFTNSTGKRAIHAAHWTR--GLTPQY 200
25 -----FRP----LTLEVLEQGKELVRTNDGRGTVVFTNSIGKRAIHAAHWTR--GLTPQY 200
26 -----FNP----LTLEALEQGKELVRTNDGRGTVLFTNSIGKRAIHAAHWTR--GLTPQY 200
27 -----GRA----LTLEDLDSAKELVRANDGRCIVIFTSSIGKRAINASHYVR--GLRPAS 201
28 -----FQP----LTLEVLEQGKELVRTNDGRGAVLFTNSTGKRAIHAAHWTR--GLTPQY 200
29 -----GAA----LTFAALDQAYHLISENNGRPNAIMSNSRALRTYRNLFVAT-HGIEPPT 201
30 -----GAA----LTFAALDQAYHLISENNGRPNAIMSNSRALRTYRNLFVAT-HGIEPPT 207
31 -----AVN----LTFAALDQAYNLVTENEGRPNAIMSNSRALRTYQNLFVAT-HGILPPT 203
32 -----GVA----LTFAALDQTYNLVTENEGRPNAIMSNSRALRTYQNLFVAT-HGILPPT 203
33 -----GVA----LTFAALDQAYNLVTENEGRPNAIMSNSRALRTYQNLFATT-YGILPPT 203
34 -----GASLDTAGQMEELQQTYNLVTANNGRPTAIMCNSRALRYIVSTFYGL--GANLEY 212
35 -----SAA----PTLAKLQETYHLVAANAGMANCIMCNTRGSRAIIKAYNDV--GMHPLY 219
36 -----GNT----LKLEDLDRAKGQIRVKEGESGVIFTNQSGYEQIRKAYYNR--DQIP-- 114
37 -----AAP-NAAARIDELQKAYSLITSNNGRPNAIMCNSRAYRWIMAAYFGV--GANPEY 211
38 -----GAA----LTFAAMDQAYNLIKSNQGRPTAIMSNSRAQRTFANLYFGA-GAGEPPM 201
39 -----GAT----LTLKDLDIAKGNVRANNGAPIIITTNQHGYEAIRTAHYDA--GITPEI 198
40 -----TNT-DKYTKLRSLQEAYFKITANNGRPNAIMCNSAAMRWIINAYYLA--GMNPEV 208
41 -----GTP----LTLEAVDRAKGYIRTSEMKNKII-TNTTGYILYREAVHNA--GISFS- 196
42 -----GAA----LTFAALDQAYHLIDSNQGRANAIMSNSRALRTFRNLYFGD-GGGEPKD 201
43 -----GAA----LTFAAVDQAYHLIESNQGRPNAIMSNSRALRTFRSLYFGA-GGGEPKE 200
44 -----GAA----LTFAALDQAYHLIESNQGRANAIMSNSRALRTFRNLYFGD-GGGEPKD 201
45 -----GAA----LTFAALDQAYNLIRSNQGRPSAIMSNSRAQRTFASLYFGA-GGGEPPM 201
46 -----GGA----LTLDCLDDAYERITAGTGRPTLIMSHSRSLRTYRKLCRDA--GFKAER 192
47 -----GGA----LTLPCLDEAYERVTAGTGRPTLIMSHSRSLRTYRNLCRVA--GFKPER 193
48 -----GGA----LTLDCLDDAYERITAGTGRPTLIMSHSRSLRTYRKLCRDS--GLVPER 192
49 -----GGA----LTLDCLDDAYERITAGTGRPTLIMSHSRSLRTYRKLCRDA--GFKAER 192

50	-----GGA----LTLACLDDAYERITAGTGRPTLIMSHSRSLRTYRKLCRDA--GFKPER	192
51	-----AGA----LTLDCLDDAYERVVAGTGRPTLIMSSKRGLRTYRSLCRAA--GFKPER	188
52	-----GAP----LSFPCLEATFGKVTANNNRPSVIMSNLRSRESYRNLCWAA--GIEPPM	193
53	---GAGGV----PALRELDQLVGKITTNGGRPHVLYTSRKGAEAIRRAHYRA--SVTP-E	204
54	-----SAS----PSLAKLQETYHLVVAGGAEANCVMCNSRASRAIIAAYNAA--GMHPQR	200
55	---GAGGV----PTLAEFDNLLDLVKSADGRATVLYSCRTGFDAWKKAHYNL--NSVPTE	205
56	-----GAA----LTFAALDQAFHLISENNGRPNAIMSNSRALRTYRNLFVAT-HGIEPPT	201
57	-----GGA----LTFPCLNLAYNFVVSNNGRPTVIVSSSRELRTYEQLCRDN--GFAPPQ	191
58	-----SVA----PTLDLLQQTWHLVTAGNARANCIMCNNRASRAIIKAYNDQ--GMHPTE	203
59	---GAGGI----PSLRELDQLVGKITANGGRPHVLYTSRKGAESIRRAYYRA--GITP-E	204
60	-----GGL----LTFACLEEAHDLVTANQGKPTVIMSNARSLRSYRNLCWNA--GIKPPE	193
61	---GAGGQ----PTLAEFDQLMALVKSADGRPTVLYSSRKGFDSWKKAHYAL--STVPAE	205
62	-----GLG----LTLDCLDDAYERITAGTGRPTLIMSHSRSLRTYRKLCRDA--GFKAER	192
63	-----GAP----LSFPCLEAAFDKITGNNNRPSFIMSNLASRASYRNLCWAT--GIKPPE	193
64	---GAGGI----PALRELDQLIGKITTNGGRPHVFYTSRKGAEAIRRAYYRE--QVTP-E	204
65	---GAGGV----PTLAEFDQLIGKVKASDGHPTVLYTSRKGLEALKKAHYNL--NSVPSE	205
66	-----LSS----LTLDCLDDAYSRVTAGTGRPTLIMSSTKGLRTYRSLCRAA--GIPQER	188
67	-----GGA----LNFHCLGDAWHRVDANDGRPTMIMSATPALATYELLCRMS--GFEPPK	193
68	-----GLA----LTTRLLDRAQRRPKSGDNFDGYICTSAQGYIEIRQAFLAS--GTLPQE	201
69	-----GLA----LTTRLLDRAQRRIKAGDNFEGMIWTSAQGYIEIRQAFLAS--GTLPQE	217
70	-----GVP-----TLQQFDDAFFSVVDNEGYPNAIMGRTGSLKRYLNLMRAA--NQDIVY	202
71	-----GVP-----TLQQFDDAFFKVVDNEGYPNAVMGRTGSLKRFLNLMRAA--NQDIVY	253
72	-----GVP-----TLQQFDDAFFKVVDNEGYPNAVMGRTGSLKRFLNLMRAA--NQDIVF	248
73	-----GVP-----TLQQFDDAFFRVVDNEGYPSVIMGRTGSLKRFLNLMRAA--NQDIKY	202
74	---N-GNA----LSFSMLDELLDAVPY--GA-DAIIMRSGTVRAYRALLRAT-GATGPTE	198
75	-----GGA----LTLPCLDEAYERVAAGTGRPTLIMSHSRSLRTYRNLCRAA--GLKPER	193
76	-----GAA----LTFAALDQAYHLISENNGRPNAIMSNSRALRTYRNLFVAT-HGIEPPT	136
77	-----AAP----LSFPCLSRAYHLVAANNGKPTVIMSNARSLESYENLCRLN--GQNLEY	193
78	---N-GAA----VTCEMLDELKDTVLN--GA-DVLMMRQGTWRAIRSILRNM-GGNDASH	198
79	-----GAA----LTFAALDQAFHLISENNGRPNAIMSNSRALRTYRNLFVAT-HGIEPPT	201
80	---N-GNA----LTLSMLDELLDAVPN--GA-DVLMMRSGTIRAYRALLRAS-GGLEPAH	198
81	---N-GAP----ITLTMLDELLDAVPN--GA-DAIMMRSGTLRAYRALLRAM-GGTLPES	199
82	---D-GAA----LTLSMLDELCDAIPN--GA-DVLVMSRGAIRAYRTLLRAT-YGTDAVM	198
83	---N-GAA----VSAEMLDELLDAVKL--GA-DVLMMRRGTWRAIRAIMRSF-GGNTGDM	198
84	---N-GAA----VSAEMLDELLDAVKL--GA-DVLMMRKGTWRAIRAIMRSF-GGNTGDM	198
85	---N-GAA----VSAEMLDELLDAVKL--GA-DVLMMRKGTWRAIRAIMRSF-GGNTGDM	198
86	---N-GAA----VSAEMLDELLDAVKL--GA-DVLMMRRGTWRAIRAIMRSF-GGNTGDM	198
87	-----GVP-----TLQQFDEALSLVVDNEAHPTAIMSNWGALKRYLALLRAA--GLKPDL	193
88	-----SAA----PTLAKLQETYHLVVAGGAEANCIMCNSRASRAIIAAYNAA--GLHPNR	151
89	---N-GAA----ISAEMLDELLDAVKL--GA-DVLMMRRGTWRAIRAIMRSF-GGNTGDM	198
90	---N-GSA----VNFGMLDELKDAVKN--GA-DVLMMRQGTWRAVRALLRAA-GGNEAAT	198
91	-----GAA----LTLDCLDDAYERVTAGTGRPTLIMSHSRSLRTYRSLCRAA--GI-YQR	148
92	-----GVP-----TLAQFDQAYSLVVDNEAHPTAIMSNWGALRRYLDLLRAA--GLKPDL	193
93	-----GVP-----TLAQFDRALSLVVDNEAHPTAIMSNWGALRRYLDLLRTA--GLKPDL	193
94	-----GNA----MSFTMLDELLDAVPN--GA-DAIFMTSAHIRALRAMLRAT-GGLEPAH	196
95	---N-GAA----VSAEMLDELLDAVKL--GA-DVLMMRRGTWRAIRAIMRSF-GGNTGDM	198
96	---N-GGS----LTLAMLDELCDAVPK--GA-DVLMMRRGTIRAYRNLLRAT-SGTDAVM	198
97	---D-GAA----VSFTMLDELRDVVKN--GC-DVLMMRRGTWRSIKALLRAM-GGNDAGT	198
98	---N-GAA----ISLSMLDELIDKLEG--RRPDALMMRTGTLRALKALWRLA-GGNTGGM	200
99	---N-GSA----VTFTMLDELRDAVKN--GC-DVLMMRKGTWRAIKQLLRAM-GGNDATT	198
100	---N-GGA----VSLAILDELVDKLTG--KKPDAFVMRSGTLRALKALWRAA-GGNTGNM	200
101	---N-GAA----LTLSMLDELLDAVPY--GA-DVIMMRAGTLRAHRALLRSL-GGTSVDN	199
102	-----GAA----LALADYDTYYFKVSA--GRCNAIVSNSAQVIRYLTLMRAA--GLTPEF	200
103	---N-GGA----ISLSMLDELIDKLDG--RRPDALMMRTGTLRALKALWRLA-GGNTGGM	200
104	---D-GGA----ITLALLDELLDKVPH--GA-DCLVMRRGTIRAYRNLLRVT-SGTDAVM	198
105	---D-GNP----LTLTMLDELCDAVPN--GA-DVIVMRRGTIRAYRALLRAT-YGTDAVM	198
106	---D-GNP----LTLTMLDELCDAVPN--GA-DVIVMRRGTIRAYRALLRAT-YGTDAVM	196
107	-----GQA----LTLSMLDELCDTVPH--GA-DALVMRRGTIRAVRGLLRAT-YGTDAVM	196
108	---D-GNP----LTLTMLDELCDAVPN--GA-DVIVMRRGTIRAYRALLRAT-YGTDAVM	198
109	---D-GNP----LTLTMLDELCDAVPN--GA-DVIVMRRGTIRAYRALLRAT-YGTDAVM	198
110	---D-GNP----LTLTMLDELCDAVPN--GA-DVIVMRRGTIRAYRALLRAT-YGTDAVM	198
111	---N-GGA----LTLSMLDQLYDMVLT--GA-DAFFMRAGTIRAYRALVRAT-GGTDATQ	198
112	---D-GNP----LTLTMLDELCDAVPN--GA-DVIVMRRGTIRAYRALLRAT-YGTDAVM	200
113	---D-GNP----LTLTMLDELCDAVPN--GA-DVIVMRRGTIRAYRALIRAT-YGTDAVM	198
114	---D-GNP----LTLTMLDELCDAVPN--GA-DVIVMRRGTIRAYRALLRAT-YGTDAVM	198
115	---N-GSA----LSLAALDELIDMVNG---RPDALMMRPGTFRALKTLWRAA-GGNTGGM	199
116	-----GSA----LTFSMLDELVDAVPL--KP-DCLIMRSGTYRAYLQLLRAT-SGTNAPL	196

```
117  ---D-GGA----ITLALLDELLDKVPH--GA-DCLVMRRGTIRAYRNLLRVT-SGTDAVM  198
118  ---D-GGG----ITLALLDELLDKVPH--GA-DCLVMRRGTIRAYRNLLRVT-SGTDAVM  198
119  -----NAA----LTFSMLDELVDAVPL--KP-DCLIMRSGTYRAYLQLLRAT-SGTNAPL  196
120  ---D-GGA----LTLSALDHLVDSVPN--GGPDFLMMRSGTRRAYVAQLRAA-GGNTGAM  199
121  ---D-GNP----LTLTMLDELCDAVPN--GA-DVIVMRRGTIRAYRALLRAT-YGTDAVM  198
122  ---D-GNP----LTLTMLDELCDAVPN--GA-DVIVMRRGTIRAYRALLRAT-YGTDAVM  198
123  ---N-GGA----LTLAKLDELCDLVPN--GA-DVIVMRRGTIRAFRSLLRAT-YGTDAVM  198
124  ---D-GNP----LTLTMLDELCDAVPN--GA-DVIVMRRGTIRAYRALLRAT-YGTDAVM  198
125  ---D-GNP----LTLTMLDELCDAVPN--GA-DVIVMRRGTIRAYRALLRAT-YGTDAVM  198
126  ---D-GNP----LTLTMLDELCDAVPN--GA-DVIVMRRGTIRAYRALLRAT-YGTDAVM  198
127  ---D-GAA----VSFTMLDELRDAVIG--GA-DVLMMRKGTWRAIKALLRAM-GGNDAGT  198
128  ---D-GAA----VSFTMLDELRDAVIG--GA-DVLMMRKGTWRSIKALLRAM-GGNDAGT  198
129  ---N-GAP----LTLSMMDELADAVIN--GA-DAFVMRPGTIRAYRALLYAT-GGVQPAM  198
130  ---N-GAP----LTLSMMDELADAVIN--GA-DAFVMRPGTIRAYRALLYAT-GGIQPAM  198
131  ---N-GAS----LTLSMMDELADAVIN--GA-DAFVMRPGTIRAYRALLYAT-GGIQPAM  198
132  ---D-GNP----LTLTMLDELCDAVPN--GA-DVIVMRRGTIRAYRALLRAT-YGTDAVM  198
133  ---T-GMN----LSFGVLDTLMHLVVSKDGQVDYMLMPARTIRAYKALLRAL-GGAGVNE  201
134  ---N-GAP----LTLSMMDELADAVIN--GA-DAFVMRPGTIRAYRALLYAT-GGIQPAM  198
135  ---D-GNP----LTLTMLDELCDAVPN--GA-DVIVMRRGTIRAYRALLRAT-YGTDAVM  198
136  -----SNA----LTFSMLDELLDAIPL--GA-DCLMMRSGTYRAYLNLLRAT-SGTNAPL  196
137  ---D-GEA----LTFERLDQLLRMVPL--GA-DALVMRGGTHDAILAMLRNL-GGTTPND  198
138  ---N-GGA----LTLSMLDELCDAVPN--GA-DVIVMRRGTIRAFRGLLRAT-YGTDAVM  201
139  ---N-GAA----VTFSMLDELRDAVLL--GA-DCFMMRQGTWRAIKALLRGF-NGNTAET  198
140  -----GAA----MNWSMLDEVVDAVNAL-GC-DAIMMRSEHKRAYLQLLRTV-GGLQPSE  197
141  ---N-GSA----LTLSMLDELVDMVPN--GA-DCLVMRSGTVRAYRALLRAA-GGTSPSE  201
142  ---N-GGA----LSFAQLDELLDAVPK--GA-DCLMMRRGTWRAIRALNRAF-GGNVAED  198
143  -----GAA----MNWSMLDEVVDAVNAL-GC-DAIMMRSEHKRAYLQLLRTV-GGLQPSE  197
144  ---N-GAA----ISPSMLDELKDAVKY--GA-DVLMMRQGTWRALREIMRQF-GGNTGDM  198
145  ---D-GGA----VSLMALDELIDAVSL--GA-DCLMMREGTWRAIRALLRAT-GGITPEY  198
146  ---D-GEA----LTFERLDQLLRAVPL--GA-DALVMRGGTHDAILAMLRNL-GGTTPDH  198
147  -----KAA----MTFSMFDELVDAVKDL-GA-DCIMMRSEHLRAYRALLRTV--NIGPSE  196
148  -----KAA----MTFSMFDELVDAVKDL-GA-DCIMMRSEHLRAYRALLRTV--NIGPSE  196
149  -----KAA----MTFSMFDELVDAVKDL-GA-DCIMMRSEHLRAYRALLRTV--NIGPSE  196
150  -----KAA----MTFSMFDELVDAVKDL-GA-DCIMMRSEHLRAYRALLRTV--NIGPSE  196
151  -----GAS----MTFSMFDELVDAVKDL-GA-DCIMMRSEHLRAYRALLRTV--NVGPSE  196
152  -----KAA----MTFSMFDELVDAVKDL-GA-DCIMMRSEHLRAYRALLRTV--NIGPSE  196
153  ---N-GAA----INPSMLDELKDAVKY--GA-DVLMMRQGTWRALREIMRQF-GGNTGDM  198
154  -----KAA----MTFSMFDELVDAVKDL-GA-DCIMMRSEHLRAYRALLRTV--NVGPSE  196
155  -----GAS----MTFSMFDELVDAVKDL-GA-DCIMMRSEHLRAYRALLRTV--NVGPSE  196
156  -----KAA----MTFSMFDELVDAVKDL-GA-DCIMMRSEHLRAYRALLRTV--NIGPSE  196
157  -----KAA----MTFSMFDELVDAVKDL-GA-DCIMMRSEHLRAYRALLRTV--NIGPSE  196
158  -----GAS----MTFSMFDELVDAVKDL-GA-DCIMMRSEHLRAYRALLRTV--NVGPSE  196
159  -----GAS----MTFSMFDELVDAVKDL-GA-DCIMMRSEHLRAYRALLRTV--NVGPSE  196
160  -----GAS----MTFSMFDELVDAVKDL-GA-DCIMMRSEHLRAYRALLRTV--NVGPSE  196
161  ---D-GGG----ITLALLDELLDKVPH--GA-DCLVMRRGTIRAYRNLLRVT-SGTDAVM  198
162  ---N-GAA----LNLSLLDKLLDAVPN--GA-DALFMPSSVIRAFRALMRTTGGGTSAAE  205
163  -----GAS----MTFSMFDELVDAVKDL-GA-DCIMMRSEHLRAYRALLRTV--NVGPSE  196
164  -----KAA----MTFSMFDELVDAVKDL-GA-DCIMMRSEHLRAYRALLRTV--NIGPSE  196
165  -----KAA----MTFSMFDELVDAVKDL-GA-DCIMMRSEHLRAYRALLRTV--NIGPSE  196
166  -----KAA----MTFSMFDELVDAVKDL-GA-DCIMMRSEHLRAYRALLRTV--NIGPSE  196
167  -----KAA----MTFSMFDELVDAVKDL-GA-DCIMMRSEHLRAYRALLRTV--NIGPSE  196
168  -----KAA----MTFSMFDELVDAVKDL-GA-DCIMMRSEHLRAYRALLRTV--NIGPSE  196
169  -----KAA----MTFSMFDELVDAVKDL-GA-DCIMMRSEHLRAYRALLRTV--NIGPSE  196
170  -----GAS----MTFSMFDELVDAVKDL-GA-DCIMMRSEHLRAYRALLRTV--NVGPSE  196
171  -----KAA----MTFSMFDELVDAVKDL-GA-DCIMMRSEHLRAYRALLRTV--NIGPSE  196
172  -----GAS----MTFSMFDELVDAVKDL-GA-DCIMMRSEHLRAYRALLRTV--NVGPSE  196
173  -----GAS----MTFSMFDELVDAVKDL-GA-DCIMMRSEHLRAYRALLRTV--NVGPSE  196
174  -----GAS----MTFSMFDELVDAVKDL-GA-DCIMMRSEHLRAYRALLRTV--NVGPSE  196
175  -----GAS----MTFSMFDELVDAVKDL-GA-DCIMMRSEHLRAYRALLRTV--NVGPSE  196
176  -----KAA----MTFSMFDELVDAVKDL-GA-DCIMMRSEHLRAYRALLRTV--NIGPSE  196
177  -----GAS----MTFSMFDELVDAVKDL-GA-DCIMMRSEHLRAYRALLRTV--NVGPSE  196
178  -----GAS----MTFSMFDELVDAVKDL-GA-DCIMMRSEHLRAYRALLRTV--NVGPSE  196
179  -----GAS----MTFSMFDELVDAVKDL-GA-DCIMMRSEHLRAYRALLRTV--NVGPSE  196
180  -----KAA----MTFSMFDELVDAVKDL-GA-DCIMMRSEHLRAYRALLRTV--NIGPSE  196
181  -----GAS----MTFSMFDELVDAVKDL-GA-DCIMMRSEHLRAYRALLRTV--NVGPSE  196
182  -----GAS----MTFSMFDELVDAVKDL-GA-DCIMMRSEHLRAYRALLRTV--NVGPSE  196
183  -----GAS----MTFSMFDELVDAVKDL-GA-DCIMMRSEHLRAYRALLRTV--NVGPSE  196
```

184	-----GAS----MTFSMFDELVDAVKDL-GA-DCIMMRSEHLRAYRALLRTV--NVGPSE	196
185	-----GAS----MTFSMFDELVDAVKDL-GA-DCIMMRSEHLRAYRALLRTV--NVGPSE	196
186	-----GAS----MTFSMFDELVDAVKDL-GA-DCIMMRSEHLRAYRALLRTV--NVGPSE	196
187	-----GAS----MTFSMFDELVDAVKDL-GA-DCIMMRSEHLRAYRALLRTV--NVGPSE	196
188	-----GAS----MTFSMFDELVDAVKDL-GA-DCIMMRSEHLRAYRALLRTV--NVGPSE	196
189	-----GAS----MTFSMFDELVDAVKDL-GA-DCIMMRSEHLRAYRALLRTV--NVGPSE	196
190	---N-GAK----LTLAKFDEALDLVAP--GRPDLICMSKRTLRELKTLRRAA-GVSEEAH	199
191	-----GAS----MTFSMFDELVDAVKDL-GA-DCIMMRSEHLRAYRALLRTV--NVGPSE	196
192	---N-GGA----VSLEALDELKDAVLL--GA-DAFMMREGTWRGLKSILRTF-GGNTAPM	198
193	-----GAS----MTFSMFDELVDAVKDL-GA-DCIMMRSEHLRAYRALLRTV--NVGPSE	196
194	-----GAS----MTFSMFDELVDAVKDL-GA-DCIMMRSEHLRAYRALLRTV--NVGPSE	196
195	-----GAS----MTFSMFDELVDAVKDL-GA-DCIMMRSEHLRAYRALLRTV--NVGPSE	196
196	-----GAS----MTFSMFDELVDAVKDL-GA-DCIMMRSEHLRAYRALLRTV--NVGPSE	196
197	-----GAS----MTFSMFDELVDAVKDL-GA-DCIMMRSEHLRSYRALLRTV--NVGPSE	196
198	-----GAS----MTFSMFDELVDAVKDL-GA-DCIMMRSEHLRAYRALLRTV--NVGPSE	196
199	-----GAS----MTFSMFDELVDAVKDL-GA-DCIMMRSEHLRAYRALLRTV--NVGPSE	196
200	-----GAS----MTFSMFDELVDAVKDL-GA-DCIMMRSEHLRAYRALLRTV--NVGPSE	196
201	-----GAS----MTFSMFDELVDAVKDL-GA-DCIMMRSEHLRAYRALLRTV--NVGPSE	196
202	---N-GAA----VSPEMLDELKDAVKY--GA-DVLMMKQATWRAIRAILRGM-PGNAADS	198
203	-----GAS----MTFSMFDELVDAVKDL-GA-DCIMMRSEHLRAYRALLRTV--NVGPSE	196
204	---N-GGA----LTLTMLDQLCDAVPN--GP-DVIVMRRGTIRAFRALLRAT-YGTDAVM	199
205	-----GAS----MTFSMFDELVDAVKDL-GA-DCIMMRSEHLRAYRALLRTV--NVGPSE	196
206	-----GAS----MTFSMFDELVDAVKDL-GA-DCIMMRSEHLRAYRALLRTV--NVGPSE	196
207	-----GAS----MTFSMFDELVDAVKDL-GA-DCIMMRSEHLRAYRALLRTV--NVGPSE	196
208	-----GAS----MTFSMFDELVDAVKDL-GA-DCIMMRSEHLRAYRALLRTV--NVGPSE	196
209	-----GAS----MTFSMFDELVDAVKDL-GA-DCIMMRSEHLRAYRALLRTV--NVGPSE	196
210	-----GAS----MTFSMFDELVDAVKDL-GA-DCIMMRSEHLRAYRALLRTV--NVGPSE	196
211	-----GAS----MTFSMFDELVDAVKDL-GA-DCIMMRSEHLRAYRALLRTV--NVGPSE	196
212	-----GAS----MTFSMFDELVDAVKDL-GA-DCIMMRSEHLRAYRALLRTV--NVGPSE	196
213	-----GAS----MTFSMFDELVDAVKDL-GA-DCIMMRSEHLRAYRALLRTV--NVGPSE	196
214	-----GAS----MTFSMFDELVDAVKDL-GA-DCIMMRSEHLRAYRALLRTV--NVGPSE	196
215	-----GAS----MTFSMFDELVDAVKDL-GA-DCIMMRSEHLRAYRALLRTV--NVGPSE	196
216	-----GAS----MTFSMFDELVDAVKDL-GA-DCIMMRSEHLRAYRALLRTV--NVGPSE	196
217	-----GAS----MTFSMFDELVDAVKDL-GA-DCIMMRSEHLRAYRALLRTV--NVGPSE	196
218	-----GAS----MTFSMFDELVDAVKDL-GA-DCIMMRSEHLRAYRALLRIV--NVGPSE	196
219	-----GAS----MTFSMFDELVDAVKDL-GA-DCIMMRSEHLRAYRALLRTV--NVGPSE	196
220	-----GAS----MTFSMFDELVDAVKDL-GA-DCIMMRSEHLRAYRALLRTV--NVGPSE	196
221	-----GAS----MTFSMFDELVDAVKDL-GA-DCIMMRSEHLRAYRALLRTV--NVGPSE	196
222	-----GAS----MTFSMFDELVDAVKDL-GA-DCIMMRSEHLRAYRALLRTV--NVGPSE	196
223	-----GAS----MTFSMFDELVDAVKDL-GA-DCIMMRSEHLRAYRALLRTV--NVGPSE	196
224	-----GAS----MTFSMFDELVDAVKDL-GA-DCIMMRSEHLRAYRALLRTV--NVGPSE	196
225	-----GAS----MTFSMFDELVDAVKDL-GA-DCIMMRSEHLRAYRALLRTV--NVGPSE	196
226	-----GAS----MTFSMFDELVDAVKDL-GA-DCIMMRSEHLRAYRALLRTV--NVGPSE	196
227	-----GAS----MTFSMFDELVDAVKDL-GA-DCIMMRSEHLRAYRALLRTV--NVGPSE	196
228	-----ASA----LNFAMLDELLDKVPN--GA-DILVMNRPTIRAYRQILRAT-SGTDAVM	196
229	-----GAS----MTFSMFDELVDAVKDL-GA-DCIMMRSEHLRAYRALLRTV--NVGPSE	196
230	-----ASA----LNFAMLDELLDKVPN--GA-DILVMNRPTIRAYRQILRAT-SGTDAVM	196
231	-----GAS----MTFSMFDELVDAVKDL-GA-DCIMMRSEHLRAYRALLRTV--NVGPSE	196
232	-----GAS----MTFSMFDELVDAVKDL-GA-DCIMMRSEHLRAYRALLRTV--NVGPSE	196
233	-----GAS----MTFSMFDELVDAVKDL-GA-DCIMMRSEHLRAYRALLRTV--NVGPSE	196
234	-----GAS----MTFSMFDELVDAVKDL-GA-DCIMMRSEHLRAYRALLRTV--NVGPSE	196
235	-----GAS----MTFSMFDELVDAVKDL-GA-DCIMMRSEHLRAYRALLRTV--NVGPSE	196
236	-----GAS----MTFSMFDELVDAVKDL-GA-DCIMMRSEHLRSYRALLRTV--NVGPSE	196
237	-----GAS----MTFSMFDELVDAVKDL-GA-DCIMMRSEHLRAYRALLRTV--NVGPSE	196
238	-----GAS----MTFSMFDELVDAVKDL-GA-DCIMMRSEHLRAYRALLRTV--NVGPSE	196
239	---N-GGS----LTLELLDQLIDLVKP--GKPDALLMSKRTRRKLAQLRRSS-GTVLESE	199
240	-----GAS----MTFSMFDELVDAVKDL-GA-DCIMMRSEHLRAYRALLRTV--NVGPSE	196
241	-----GAS----MTFSMFDELVDAVKDL-GA-DCIMMRSEHLRAYRALLRTV--NIGPSE	196
242	-----GAS----MTFSMFDELVDAVKDL-GA-DCIMMRSEHLRAYRALLRTV--NVGPSE	196
243	-----GAS----MTFSMFDELVDAVKDL-GA-DCIMMRSEHLRAYRALLRTV--NVGPSE	196
244	-----GAS----MTFSMFDELVDAVKDL-GA-DCIMMRSEHLRAYRALLRTV--NVGPSE	196
245	-----GAS----MTFSMFDELVDAVKDL-GA-DCIMMRSEHLRAYRALLRTV--NVGPSE	196
246	-----GAS----MTFSMFDELVDAVKDL-GA-DCIMMRSEHLRAYRALLRTV--NVGPSE	196
247	-----GAS----MTFSMFDELVDAVKDL-GA-DCIMMRSEHLRAYRALLRTV--NVGPSE	196
248	-----GAS----MTFSMFDELVDAVKDL-GA-DCIMMRSEHLRAYRALLRTV--NVGPSE	196
249	-----GAS----MSFSMFDELVDAVKDL-GA-DCIMMRSEHLRAYRALLRTV--NVGPSE	196
250	-----GAS----MTFSMFDELVDAVKDL-GA-DCIMMRSEHLRAYRALLRTV--NVGPSE	196

```
251  -----GAS----MTFSMFDELVDAVKDL-GA-DCIMMRSEHLRAYRALLRTV--NVGPSE  196
252  -----GAS----MTFSMFDELVDAVKDL-GA-DCIMMRSEHLRAYRALLRTV--NVGPSE  196
253  -----GTS----MTFSMFDELVDAVKDL-GA-DCIMMRSEHLRAYRALLRTV--NVGPSE  196
254  -----GAS----MTFSMFDELVDAVKDL-GA-DCIMMRSEHLRAYRALLRTV--NVGPSE  196
255  -----GAS----MTFSMFDELVDAVKDL-GA-DCIMMRSEHLRAYRALLRTV--NVGPSE  196
256  -----GAS----MTFSMFDELVDAVKDL-GA-DCIMMRSEHLRAYRALLRTV--NIGPSE  196
257  -----GAS----MTFSMFDELVDAVKDL-GA-DCIMMRSEHLRAYRALLRTV--NIGPSE  196
258  -----GVS----MTFSMFDELVDAVKDL-GA-DCIMMRSEHLRAYRALLRTV--NVGPSE  196
259  -----GAS----MTFSMFDELVDAVKDL-GA-DCIMMRSEHLRAYRALLRTV--NVGPSE  196
260  -----GAS----MTFSMFDELVDAVKDL-GA-DCIMMRSEHLRAYRALLRTV--NVGPSE  196
261  -----GAS----MTFSMFDELVDAVKDL-GA-DCIMMRSEHLRAYRALLRTV--NVGPSE  196
262  -----GAS----MTFSMFDELVDAVKDL-GA-DCIMMRSEHLRAYRALLRTV--NVGPSE  196
263  -----KAS----MTFSMFDELVDAVKDL-GA-DCIMMRSEHLRAYRALLRTV--NVGPSE  196
264  -----KAS----MTFSMFDELVDAVKDL-GA-DCIMMRSEHLRAYRALLRTV--NVGPSE  196
265  -----GAS----MTFSMFDELVDAVKDL-GA-DCIMMRSEHLRAYRALLRTV--NVGPSE  196
266  -----GAS----MTFSMFDELVDAVKDL-GA-DCIMMRSEHLRAYRALLRTV--NVGPSE  196
267  -----GAS----MTFSMFDELVDAVKDL-GA-DCIMMRSEHLRAYRALLRTV--NVGPSE  196
268  -----GAS----MTFSMFDELVDAVKDL-GA-DCIMMRSEHLRAYRALLRTV--NVGPSE  196
269  -----GAS----MTFSMFDELVDAVKDL-GA-DCIMMRSEHLRSYRALLRTV--NIGPSE  196
270  -----ASA----LNFAMLDELLDKVPN--GA-DVLVMNRPTIRAYRQILRAT-SGTDAVM  196
271  -----GAS----MTFSMFDELVDAVKDL-GA-DCIMMRSEHLRAYRALLRTV--NVGPSE  196
272  -----GAS----MTFSMFDELVDAVKDL-GA-DCIMMRSEHLRAYRALLRTV--NVGPSE  196
273  -----GAS----MTFSMFDELVDAVKDL-GA-DCIMMRSEHLRAYRALLRTV--NVGPSE  196
274  -----GAS----MTFSMFDELVDAVKDL-GA-DCIMMRSEHLRAYRALLRTV--NVGPSE  196
275  -----GAS----MTFSMFDELVDAVKDL-GA-DCIMMRSEHLRAYRALLRTV--NVGPSE  196
276  -----GAS----MTFSMFDELVDAVKDL-GA-DCIMMRSEHLRAYRALLRTV--NVGPSE  196
277  -----GAS----MTFSMFDELVDAVKDL-GA-DCIMMRSEHLRAYRALLRTV--NVGPSE  196
278  -----GAS----MTFSMFDELVDAVKDL-GA-DCIMMRSEHLRAYRALLRTV--NVGPSE  196
279  -----GAS----MTFSMFDELVDAVKDL-GA-DCIMMRSEHLRAYRALLRTV--NVGPSE  196
280  -----GAS----MTFSMFDELVDAVKDL-GA-DCIMMRSEHLRAYRALLRTV--NVGPSE  196
281  ---D-GNP----LTLTMLDELCDAVPN--GA-DVIVMRRGTIRAYRALLRAT-YGTDAVM  198
282  -----GAS----MTFSMFDELVDAVKDL-GA-DCIMMRSEHLRAYRALLRTV--NVGPSE  196
283  -----GAS----MTFSMFDELVDAVKDL-GA-DCIMMRSEHLRAYRALLRTV--NVGPSE  196
284  -----GAS----MTFSMFDEPVDAVKDL-GA-DCIMMRSEHLRAYRALLRTV--NVGPSE  196
285  -----GAS----MTFSMFDELVDAVKDL-GA-DCIMMRSEHLRAYRALLRTV--NVGPSE  196
286  -----GAS----MTFSMFDELVDAVKDL-GA-DCIMMRSEHLRAYRALLRTV--SLGPSE  196
287  -----GAS----MTFSMFDELVDAVKDL-GA-DCIMMRSEHLRAYRALLRTV--NVGPSE  196
288  -----GAS----MTFSMFDELVDAVKDL-GA-DCIMMRSEHLRAYRALLRTV--SLGPSE  196
289  -----GAS----MTFSMFDELVDAVKDL-GA-DCIMMRSEHLRAYRALLRTV--NVGPSE  196
290  -----GAS----MTFSMFDELVDAVKDL-GA-DCIMMRSEHLRAYRALLRTV--NVGPSE  196
291  -----GAS----MTFSMFDELVDAVKDL-GA-DCIMMRSEHLRAYRALLRTV--NVGPSE  196
292  -----GAS----MTFSMFDELVDAVKDL-GA-DCIMMRSEHLRAYRALLRTV--NVGPSE  196
293  -----GAS----MTFSMFDELVDAVKDL-GA-DCIMMRSEHLRAYRALLRTV--NVGPSE  196
294  -----GAS----MTFSMFDELVDAVKDL-GA-DCIMMRSEHLRAYRALLRTV--NVGPSE  196
295  -----GAS----MTFSMFDELVDAVKDL-GA-HCIMMRSEHLRAYRALLRTV--NVGPSE  196
296  -----GAS----MTFSMFDELVDAVKDL-GA-DCIMMRSEHLRAYRALLRTV--NVGPSE  196
297  -----GAS----MTFSMFDELVDAVKDL-GA-DCIMMRSGHLRAYRALLRTV--NVGPSE  196
298  -----GAS----MTFSMFDELVDAVKDL-GA-DCIMMRSEHLRAYRALLRTV--NVGPSE  196
299  -----GAS----MTFSMFDELVDAVKDL-GA-DCIMMRSEHLRAYRALLRTV--NVGPSE  196
300  -----GAS----MTFSMFDELVDAVKDL-GA-DCIMMRSEHLRAYRALLRTV--NVGPSE  190
301  -----GAS----MTFSMFDELVDAVKDL-GA-DCIMMRSEHLRAYRALLRTV--NVGPSE  196
302  -----GAS----MTFSMFDELVDAVKDL-GA-DCIMMRSEHLRAYRALLRTV--NVGPSE  196
303  -----GAS----MTFSMFDELVDAVKDL-GA-DCIMMRSEHLRAYRALLRTV--NIGPSE  196
304  -----GAS----MTFSMFDELIDAVKDL-GA-DCIMMRSEHLRAYRALLRTV--NVGPSE  196
305  -----GAS----MTFSMFDELVDAVKDL-GA-DCIMMRSEHLRAYRALLRTV--NVGPSE  196
306  -----GAS----MTFSMFDELVDAVKDL-GA-DCIMMRSEHLRAYRALLRTV--NVGPSE  196
307  -----GAS----MTFSMFDELVDAVKDL-GA-DCIMMRSEHLRAYRALLRTV--NIGPSE  196
308  -----GAS----MTFSMFDELVDAVKDL-GA-DCIMMRSEHLRAYRALLRTV--NVGPSE  196
309  -----GAS----MTFSMFDELVDAVKDL-GA-DCIMMRSEHLRAYRALLRTV--SLGPSE  196
310  -----GAS----MTFSMFDELVDAVKDL-GA-DCIMMRSEHLRAYRALLRTV--SLGPSE  196
311  -----GAS----MTFSMFDELVDAVKDL-GA-DCIMMRSEHLRAYRALLRTV--NIGPSE  196
312  -----GAS----MTFSMFDELVDAVKDL-GA-DCIMMRSEHLRAYRALLRTV--NVGPSE  196
313  -----GAS----MTFSMFDELVDAVKDL-GA-DCIMMRSEHLRAYRALLRTV--NVGPSE  196
314  -----GAS----MTFSMFDELVDAVKDL-GA-DCIMMRSEHLRAYRALLRTV--SLGPSE  196
315  -----GAS----MTFSMFDELVDAVKDL-GA-DCIMMRSEHLRAYRALLRTV--NVGPSE  196
316  -----GAS----MTFSMFDELVDAVKDL-GA-DCIMMRSEHLRAYRALLRTV--NVGPSE  196
317  -----KAS----MTFSMFDELVDAVKDL-GA-DCIMMRSEHLRAYRALLRTV--NVGPSE  196
```

```
318  -----GAS----MTFSMFDELVDAVKDL-GA-DCIMMRSEHLRAYRALLRTV--NVGPSE  196
319  -----GAS----MTFSMFDELVDAVKDL-GA-DCIMMRSEHLRAYRALLRTV--NVGPSE  196
320  -----GAS----MTFSMFDELVDAVKDL-GA-DCIMMRSEHLRAYRALLRTV--SLGPSE  196
321  -----GAS----MTFSMFDELVDAVKDL-GA-DCIMMRSEHLRAYRALLRTV--NVGPSE  196
322  -----GAS----MTFSMFDELIDAVKDL-GA-DCIMMRSEHLRAYRALLRTV--SLGPSE  196
323  -----GAS----MTFSMFDELVDAVKDL-GA-DCIMMCSEHLRAYRALLRTV--SLGPSE  196
324  -----GAS----MTFSMFDELVDAVKDL-GA-DCIMMRSEHLRAYRALLRTV--NVGPSE  196
325  -----GAS----MTFSMFDELVDAVKDL-GA-DCIMMRSEHLRAYRALLRTV--NVGPSE  196
326  -----GAS----MTFSMFDELVDAVKDL-GA-DCIMMRSEHLRAYRALLRTV--NVGPSE  196
327  -----GAS----MTFSMFDELVDAVKDL-GA-DCIMMRSEHLRAYRALLRTV--NVGPSE  196
328  -----GSS----MTFSMFDELVDAVKDL-GA-DCIMMRSEHLRAYRALLRTV--NVGPSE  196
329  -----GAS----MTFSMFDELVDAVKDL-GA-DCIMMRSEHLRAYRALLRTV--SLGPSE  196
330  -----GAS----MTFSMFDELVDAVKDL-GA-DCIMMRSEHLRAYRALLRTV--SLGPSE  196
331  -----GAS----MTFSMFDELVDAVKDL-GA-DCIMMRSEHLRAYRALLRTV--NVGPSE  196
332  -----GAS----MTFSMFDELVDAVKDL-GA-DCIMMRSEHLRAYRALLRTV--SLGPSE  196
333  -----GAS----MTFSMFDELVDAVKDL-GA-DCIMMRSEHLRAYRALLRTV--SLGPSE  196
334  -----KAS----MTFSMFDELVDAVKDL-GA-DCIMMRSEHLRAYRALLRTV--NVGPSE  196
335  -----GAS----MTFSMFDELVDAVKDL-GA-DCIMMRSEHLRAYRALLRTV--SLGPSE  196
336  -----GAS----MTFSMFDELVDAVKDL-GA-DCIMMRSEHLRAYRALLRTV--NVGPSE  196
337  -----GAS----MTFSMFDELVDAVKDL-GA-DCIMMRSEHLRAYRALLRTV--SLGPSE  196
338  -----GAS----MTFSMFDELVDAVKDL-GA-DCIMMRSEHLRAYRALLRTV--NVGPSE  196
339  -----GAS----MTFSMFDELVDAVKDL-GA-DCIMMRSEHLRAYRALLRTV--NIGPSE  196
340  -----GAS----MTFSMFDELVDAVKDL-GA-DCIMMRSEHLRAYRALLRTV--NVGPSE  196
341  -----GAS----MTFSMFDELVDAVKDL-GA-DCIMMRSEHLRAYRALLRTV--SLGPSE  196
342  -----GAS----MTFSMFDELVDAVKDL-GA-DCIMMRSEHLRAYRALLRTV--NVGPSE  196
343  -----GAS----MTFSMFDELVDAVKDL-GA-DCIMMRSEHLRAYRALLRTV--SLGPSE  196
344  -----GAS----MTFSMFDELVDAVKDL-GA-DCIMMRSEHLRAYRALLRTV--SLGPSE  196
345  -----GAS----MTFSMFDELVDAVKDL-GA-DCIMMRSEHLRAYRALLRTV--NVGPSE  196
346  -----GAS----MTFSMFDELVDAVKDL-GA-DCIMMRSEHLRAYRALLRTV--NVGPSE  196
347  -----GAS----MTFSMFDELVDAVKDL-GA-DCIMMRSEHLRAYRALLRTV--NVGPSE  196
348  -----KAS----MTFSMFDELVDAIKDL-GA-DCIMMRSEHLRAYRALLRTV--NVGPSE  196
349  -----GAS----MTFSMFDELVDAVKDL-GA-DCIMMRSEHLRAYRALLRTV--NIGPSE  196
350  -----GAS----MTFSMFDELVDAVKDL-GA-DCIMMRSEHLRAYRALLRTV--SLGPSE  196
351  ---D-GNP----LTLTMLDELCDAVPN--GA-DVIVMRRGTIRAYRALLRAT-YGTDAVM  179
352  -----GAS----MTFSMFDELVDAVKDL-GA-DCIMMRSEHLRAYRALLRTV--SLGPSE  196
353  --AD-GDA----LSFGLLDELLDKVDDKDGEVDYILMNSRTLRSYLGLLRGL-GGASIGD  202
354  -----GAS----MTFSMFDELVDAVKDL-GA-DCIMMRSEHLRAYRALLRTV--NVGPSE  193
355  -----KAS----MTFSMFDELVDAVKDL-GA-DCIMMRSEHLRAYRALLRTV--NVGPSE  196
356  -----GAS----MTFSMFDELVDAVKDL-GA-DCIMMRSEHLRAYRALLRTV--SLGPSE  196
357  -----GAS----MTFSMFDELVDAVKDL-GA-DCIMMRSEHLRAYRALLRTV--NVGPSE  196
358  -----GAS----MTFSMFDELVDAVKDL-GA-DCIMMRSEHLRAYRALLRTV--NIGPSE  196
359  -----GAS----MTFSMFDELVDAVKDL-GA-DCIMMRSEHLRAYRALLRTV--NVGPSE  196
360  -----GAS----MTFSMFDELVDAVKDL-GA-DCIMMRSEHLRAYRALLRTV--SLGPSE  196
361  -----KAA----MTFSMFDELVDAVKDL-GA-DCIMMRSEHLRAYRALLRTV--NVGPSE  196
362  -----SAS----MTFSMFDELVDAVKDL-GA-DCIMMRSEHLRAYRALLRTV--NVGPSE  196
363  -----GAS----MTFSMFDELVDAVKDL-GA-DCIMMRSEHLRAYRALLRTV--SLGPSE  196
364  -----GAS----MTFSMFDELVDAVKDL-GA-DCIMMRSEHLRAYRALLRTV--SLGPSE  196
365  -----GAS----MTFSMFDELVDAVKDL-GA-DCIMMRSEHLRAYRALLRTV--SLGPSE  196
366  -----GAS----MTFSMFDELVDAVKDL-GA-DCIMMRSEHLRAYRALLRTV--SLGPSE  196
367  -----KAS----MTFSMFDELVDAVKDL-GA-DCIMMRSEHLRAYRALLRTV--NVGPSE  196
368  -----KAS----MTFSMFDELVDAIKDL-GA-DCIMMRSEHLRAYRALLRTV--NVGPSE  196
369  -----GAS----MTFSMFDELVDAVKDL-GT-DCIMMRSEHLRAYRALLRTV--SLGPSE  196
370  -----GAS----MTFSMFDELVDAVKDL-GA-DCIMMRSEHLRAYRALLRTV--SLGPSE  196
371  -----GAS----MTFSMFDELVDAVKDL-GA-DCIMMRSEHLRAYRALLRTV--SLGPSE  196
372  -----GAS----MTFSMFDELVDAVKDL-GA-DCIMMRSEHLRAYRALLRTV--SLGPSE  196
373  -----GAS----MTFSMFDELVDAVKDL-GA-DCIMMRSEHLRAYRALLRTV--SLGPSE  196
374  -----CAS----MTFSMFDELVDAVKDL-GA-DCIMMRSEHLRAYRALLRTV--NVGPSE  196
375  -----GAS----MTFSMFDELVDAVKDL-GA-DCIMMRSEHLRAYRALLRTV--SLGPSE  196
376  -----GAS----MTFSMFDELVDAVKDL-GA-DCIMMRSEHLRAYRALLRTV--NVGPSE  196
377  ---N-GGS----LTLELLDQLVDLVKP--GKPDALLMSKRTRRKLSQLRRQS-GTVLESD  199
378  -----GAS----MTFFMFDELVDAVKDL-GA-DCIMMRSEHLRAYRALLRTV--SLGPSE  196
379  -----GAS----MTFSMFDELVDAVKDL-GA-DCIMMRSEHLRAYRALLRTV--SLGPSE  196
380  -----KAS----MTFSMFDELVDAIKDL-GA-DCIMMRSEHLRAYRALLRTV--NVGPSE  196
381  -----KAS----MTFSMFDELVDAIKDL-GA-DCIMMRSEHLRAYRALLRTV--NVGPSE  196
382  ---N-GSN----LSFEILDEMLDLVTAKDGEVDYLVMHARTIRSYKTLLRAL-GGVTMQE  201
383  -----KAS----MTFSMFDELVDAIKDL-GA-DCIMMRSEHLRAYRALLRTV--NVGPSE  196
384  -----KAS----MTFSMFDELVDAIKDL-GA-DCIMMRSEHLRAYRALLRTV--NVGPSE  196
```

```
385  -----GAS----MTFSMFDELVDAVKDL-GA-DCIMMRSEHLRAYRALLRTV--SLGPSE  196
386  -----GAS----MTFSMFDELVDAVKDL-GA-DCIMMRSEHLRAYRALLRTV--NVGPSE  196
387  ---GPTNN----LTFSMLDDLVDRVPN--GA-DAIMMHSSTLRYYRALCRAQ-NGTDAAQ  200
388  -----KAS----MTFSMFDELVDAIKDL-GA-DCIMMRSEHLRAYRALLRTV--NVGPSE  196
389  -----DAS----MTFSMFDELVDAVKDL-GA-DCIMMRSEHLRAYRALLRTV--NVGPSE  196
390  ---T-GKN----LGFEDLDTLLHLVVSKDGQVDYMLFPARTIRSFKALLRGL-GGASVNE  201
391  -----KAS----MTFSMFDELVDAIKDL-GA-DCIMMRSEHLRAYRALLRTV--NVGPSE  196
392  -----GAS----MTFSMFDELVDAVKDL-GA-DCIMMRSEHLRAYRALLRTV--SLGPSE  196
393  -----GAS----MTFSMFDELVDAVKDL-GA-DCIMMRSEHLRAYRALLRTV--SLGPSE  196
394  -----KAS----MTFSMFDELVDAIKDL-GA-DCIMMRSEHLRAYRALLRTV--NVGPSE  196
395  -----KAS----MTFSMFDELVDAIKDL-GA-DCIMMRSEHLRAYRALLRTV--NVGPSE  196
396  -----AAS----MTFSMFDELVDAVKDL-GA-DCIMMRSEHLRAYRALLRTV--NVGPSE  196
397  GAAN-GAV----LSFEDLDALIDLVTDKDGQVDYLMMHGRTRRKYLALLRSL-GGTSPAD  205
398  -----GAS----MTFSMFDELVDAVKDL-GA-DCIMMRSEHLRAYRALLRTV--NVGPSE  196
399  -----NAS----ITFSMFDELVDAVKDL-GA-DCIMMRSEHLRAYRALLRTV--NVGPSE  196
400  -----KAS----MTFSMFDELVDAIKDL-GA-DCIMMRSEHLRAYRALLRTV--NVGPSE  196
401  -----KAS----MTFSMFDELVDAIKDL-GA-DCIMMRSEHLRAYRALLRTV--NVGPSE  196
402  -----KAS----MTFSMFDELVDAIKDL-GA-DCIMMRSEHLRAYRALLRTV--NVGPSE  196
403  -----GAS----MTFSMFDELVDAVKDL-GA-DCIMMRSEHLRAYRALLRTV--NVGPSE  196
404  -----GAS----MTFSMFDELVDAVKDL-GA-DCIMMRSEHLRAYRALLRTV--NVGPSE  196
405  -----GAS----MTFSMFDELVDAVKDL-GA-DCIMMRSEHLRAYRALLRTV--NVGPSE  196
406  -----KAS----MTFSMFDELVDAIKDL-GA-DCIMMRSEHLRAYRALLRTV--NVGPSE  196
407  -----SAS----MTFSMFDELVDAVKDL-GA-DCIMMRSEHLRAYRALLRTV--NVGPSE  196
408  -----GAS----MTFSMFDELVDAVKDL-GA-DCIMMRSEHLRAYRALLRTV--SLGPSE  196
409  -----KAS----MTFSMFDELVDAIKDL-GA-DCIMMRSEHLRAYRALLRTV--NVGPSE  196
410  -----KAS----MTFSMFDELVDAIKDL-GA-DCIMMRSEHLRAYRALLRTV--NVGPSE  196
411  -----KAS----MTFSMFDELVDAIKDL-GA-DCIMMRSEHLRAYRALLRTV--NVGPSE  196
412  -----KAS----MTFSMFDELVDAIKDL-GA-DCIMMRSEHLRAYRALLRTV--NVGPSE  196
413  -----GAS----MTFSMFDELVDAVKDL-GA-DCIMMRSEHLRAYRALLRTV--NVGPSE  196
414  -----KAS----MTFSMFDELVDAIKDL-GA-DCIMMRSEHLRAYRALLRTV--NVGPSE  196
415  -----KAS----MTFSMFDELVDAIKDL-GA-DCIMMRSEHLRAYRALLRTV--NVGPSE  196
416  -----KAS----MTFSMFDELVDAIKDL-GA-DCIMMRSEHLRAYRALLRTV--NVGPSE  196
417  -----AAS----MTFSMFDELVDAVKDL-GA-DCIMMRSEHLRAYRALLRTV--NVGPSE  196
418  -----GAS----MTFSMFDELVDAVKDL-GA-DCIMMRSEHLRAYRALLRTV--SLGPSE  196
419  ---D-GNP----ISYAALDELKDMVKL--GA-DVFMMRSGTWRAIRELNRIH-GGNTAET  198
420  -----KAS----MTFSMFDELVDAIKDL-GA-DCIMMRSEHLRAYRALLRTV--NVGPSE  196
421  -----GAS----MTFSMFDELVDAVKDL-GA-DCIMMRSEHLRAYRALLRTV--NVGPSE  196
422  -----GAS----MTFSMFDELVDAVKDL-GA-DCIMMRSEHLRAYRALLRTV--NVGPSE  196
423  -----AAS----MTFSMFDELVDAVKDL-GA-DCIMMRSEHLRAYRALLRTV--NVGPSE  196
424  -----KAS----MTFSMFDELVDAIKDL-GA-DCIMMRSEHLRAYRALLRTV--NVGPSE  196
425  -----GAS----MTFSMFDELVDAVKDL-GA-DCIMMRSEHLRAYRALLRTV--NVGPSE  196
426  -----GAS----MTFSMFDELVDAVKDL-GA-DCIMMRSEHLRAYRALLRTV--NVGPSE  196
427  -----GAS----MTFSMFDELVDAVKDL-GA-DCIMMRSEHLRAYRALLRTV--NVGPSE  196
428  -----KAS----MTFSMFDELVDAIKDL-GA-DCIMMRSEHLRAYRALLRTV--NVGPSE  196
429  -----GAS----MTFSMFDELVDAVKDL-GA-DCIMMRSEHLRAYRALLRTV--NVGPSE  196
430  -----GAS----MTFSMFDELVDAVKDL-GA-DCIMMRSEHLRAYRALLRTV--NVGPSE  196
431  -----KAS----MTFSMFDELVDAIKDL-GA-DCIMMRSEHLRAYRALLRTV--NVGPSE  196
432  ---N-GGA----LSFAFLDELLDLVVDKDGNVDYITMHSRTIRSYKALIRAL-GGVTADD  201
433  -----GAS----MTFSMFDELVDAVKDL-GA-DCIMMRSEHLRAYRALLRTV--NVGPSE  196
434  -----GAS----MTFSMFDELVDAVKDL-GA-DCIMMRSEHLRAYRALLRTV--NVGPSE  196
435  -----GAS----MTFSMFDELVDAVKDL-GA-DCIMMRSEHLRAYRALLRTV--NVGPSE  196
436  -----KAS----MTFSMFDELVDAIKDL-GA-DCIMMRSEHLRAYRALLRTV--NVGPSE  196
437  -----GAS----MTFSMFDELVDAVKDL-GA-DCIMMRSEHLRAYRALLRTV--SVGPSE  196
438  -----KAS----MTFSMFDELVDAIKDL-GA-DCIMMRSEHLRAYRALLRTV--NVGPSE  196
439  -----ASA----LNFAMLDELLDKVPN--GA-DVLVMNRPTIRAYRQILRAT-SGTDAVM  196
440  -----GVS----MTFSMFDELVDAVKDL-GA-DCIMMRSEHLRAYRALLRTV--NVGPSE  196
441  ---N-GGS----LTLELLDQLVDLVKP--GKPDALLMSKRTRRKLSQLRRQS-GTVLESD  199
442  -----NSA----MTFSMLDELVDAVKDL-GA-DAIMMRSEHIRAYRALLRTV--NVGPQE  195
443  -----GAS----MTFSMFDELVDAVKDL-GA-DCIMMRSEHLRAYRALLRTV--SLGPSE  196
444  ---N-GAD----VSFSLLDELLDAVKAKDGKVDFLMMHARTIRSVKTLLRGQ-SGAGIIE  201
445  ---N-GGA----LSFEWLDNLLDLVVSKDGDVDYLIMHARTRRSYKTLLRAL-GGVPMEE  201
446  -----GAS----MTFSMFDELVDAVKDL-GA-DCIMMRSEHLRAYRALLRTV--NVGPSE  196
447  -----GAS----MTFSMFDELVDAVKDL-GA-DCIMMRSEHLRAYRALLRTV--NVGPSE  196
448  ---N-GGS----LTLDLMDQLIDLVKP--GKPDALLMSKRTRRKLSALRRAS-GNLLETD  197
449  -----GAS----MTFSMFDELVDAVKDL-GA-DCIMMRSEHLRAYRALLRTV--NVGPSE  196
450  -----GAS----MTFSMFDELVDAVKDL-GA-DCIMMRSEHLRAYRALLRTV--NVGPSE  196
451  ---N-GGS----LTLELLEQLIDLVKP--GKPDALLMAKRTRRKLAQLRRSS-GSVLETE  199
```

```
452  ---D-GGA----LTLDLMDELIDLVRP--GRPDALLMHKRTRRKLSQLRRTS-GNLLETD  199
453  -----KAS----MTFSMFDELVDAIKDL-GA-DCIMMRSEHLRAYRALLRTV--NVGPSE  196
454  GAAN-GAA----LSFEDLDALIDLVTDKDGQVDYLMMHGRTRRKYLALLRSL-GGTSPAD  205
455  ---N-GAA----LTLSMMDELADQVIN--GA-DAFIMRPGTIRAYRALLYAS-GGIQPAM  198
456  -----GAS----MTFSMFDELVDAVKDL-GA-DCIMMRSEHLRAYRALLRTV--NVGPSE  196
457  -----GAS----MTFSMFDELVDAVKDL-GA-DCIMMRSEHLRAYRALLRTV--NVGPSE  196
458  -----KAS----MTFSMFDELVDAIKDL-GA-DCIMMRSEHLRAYRALLRTV--NVGPSE  196
459  ---N-GAA----MTLGMLDQLIDAVPN--GA-DFLMMRSGTRRAYTALVRAA-GGNTASM  198
460  -----GAS----MTFSMFDELVDAVKDL-GA-DCIMMRSEHLRAYRALLRTV--NVGPSE  196
461  GAAN-GAG----LSFEDLDALIDLVTDKDGQVDYLMMHSRTRRKYLALLRAL-GGTSPGD  205
462  -----GAS----MTFSMFDELVDAVKDL-GA-DCIMMRSEHLRAYRALLRTV--NVGPSE  196
463  -----GAP----LSFPCLSKAWHLVKANNGKPTVIMSNARSLESYENLCRLS--GQNVEY  107
464  -----GAS----MTFSMFDELVDAVKDL-GA-DCIMMRSEHLRAYRALLRTV--NVGPSE  196
465  -----GVS----MTFSMFDELVDAVKDL-GA-DCIMMRSEHLRAYRALLRTV--NVGPSE  196
466  -----GAS----MTFSMFDELVDAVKDL-GA-DCIMMRSEHLRAYRALLRTV--NVGPSE  196
467  -----GAS----MTFSMFDELVDAVKDL-GA-DCIMMRSEHLRAYRALLRTV--NVGPSE  196
468  -----GAS----MTFSMFDELVDAVKDL-GA-DCIMMRSEHLRAYRALLRTV--NVGPSE  196
469  -----GAA----ITFSMFDELVDAVKDL-GA-DCIMMRSEHLRAYRALLRTM--NAGPAE  195
470  -----GAS----MTFSMFDELVDAVKDL-GA-DCIMMRSEHLRAYRALLRTV--NVGPSE  196
471  ---N-GAA----LSFEKLDELMDTVTDKDGEVDYLTMHARSLRSFNALLRGL-GGASIGD  202
472  ---N-GNP----LTLTMMDELCDAVPN--GA-DVLVMRRGTIRAYRALLRAT-YGTDAVM  208
473  ---N-GAA----LSFEKLDELMDTVTDKDGEVDYLTMHARSLRSFNALLRGL-GGASIGD  202
474  -----GAS----MTFSMFDELVDAVKDL-GA-DCIMMRSEHLRAYRALLRTV--NVGPSE  196
475  -----GAS----MTFSMFDELVDAVKDL-GA-DCIMMRSEHLRAYRALLRTV--NVGPSE  196
476  GAAN-GAA----LSFEDLDALIDLVTDKDGQVDYLMMHSRTRRKYLALLRAL-GGTSPGD  205
477  ---N-GSA----LQFELLDELIDSVKDKDGQVDYILASARTIRSYYALLRGL-GGATIGD  201
478  ---N-GAT----LSFELLDELLDRVTAKDGKVDFLMMHGRTIRSLKALLRAQ-GGAGIVE  201
479  GAAN-GAA----LSFEDLDALIDLVTDKDGQVDYIMMHGRTRRKYLALLRAL-GGTSPGD  205
480  ---N-GSA----LSFEILDELMDLVVDKDGQVDYLTMHARTLRSYNALLRSL-GGASIGD  202
481  -----GVS----MTFSMFDELVDAVKDL-GA-DCIMMRSEHLRAYRALLRTV--NVGPSE  196
482  -----GVS----MTFSMFDELVDAVKDL-GA-DCIMMRSEHLRAYRALLRTV--NVGPSE  196
483  -----DSA----MTFSMLDELVDAVKDL-GA-DAIMMRSEHLRAYRALLRTV--NVGPQE  195
484  -----GVS----MTFSMFDELVDAVKDL-GA-DCIMMRSEHLRAYRALLRTV--NVGPSE  196
485  ---D-GGA----LTLDLMDELIDLVRP--GRPDALLMHKRTRRKLSQLRRTS-GNLLETD  199
486  -----DSA----MTFSMLDELVDAVKDL-GA-DAIMMRSEHIRAYRALLRTV--NVGPQE  195
487  -----KAS----MTFSMFDELVDAIKDL-GA-DCIMMRSEHLRAYRALLRTV--NVGPSE  196
488  -----DSA----MTFSMLDELVDAVKDL-GA-DAIMMRSEHIRAYRALLRTV--NVGPTE  195
489  -----DSA----MTFSMLDELVDAVKDL-GA-DAIMMRSEHIRAYRALLRTV--NVGPQE  195
490  GAAN-GAG----LSFEDLDALIDLVTDKDGQVDYLMMHSRTRRKYLALLRAL-GGTSPGD  205
491  -----GAS----MTFSMFDELVDAVKDL-GA-DCIMMRSEHLRAYRALLRTV--NVGPSE  196
492  ---N-GGA----LSFEMLDELLDAVKAKDGKVDFLMMHGRTIRSLKSLLRGQ-GGAGIVE  201
493  -----GAS----MTFSMFDELVDAVKDL-GA-DCIMMRSEHLRAYRALLRTV--NVGPSE  196
494  ---N-GGS----LTLDLMDQLIDLVKP--GRPDALLMSKRSRRKLSALRRAS-GNLLETD  199
495  -----GAS----MTFSMFDELVDAVKDL-GA-DCIMMRSEHLRAYRALLRTV--NVGPSE  196
496  -----GAS----MTFSMFDELVDAVKDL-GA-DCIMMRSEHLRAYRALLRTV--NVGPSE  196
497  --DN-GAN----LSFDQLDQLIDYVKDKDGQVDYMMMPARTIRSFYALLRQL-GGATIND  202
498  -----GAS----MTFSMFDELVDAVKDL-GA-DCIMMRSEHLRAYRALLRTV--NVGPSE  196
499  -----DSA----MTFSMLDELVDAVKDL-GA-DAIMMRSEHIRAYRALLRTV--NVGPTE  195
500  GAAN-GAG----LSFEDLDALIDLVTDKDGQVDYLMMHSRTRRKYLALLRAL-GGTSPGD  205

           310       320       330       340       350       360

1    -----PVL-NGKS------SFDGWKIYINDF-QPTHKIK-----------------DKDK  221
2    -----PVL-NGKS------SFDGWKIYINDF-QPTHKIK-----------------DKDK  221
3    -----PVV-NGKS------SFDGWKIYINDF-QPTHKIK-----------------DKDK  221
4    -----PVL-NGKS------SFDGWKIYINDF-QPTHKIK-----------------DKDK  221
5    -----PVL-NGKS------SFDGWKIYINDF-QPTHKIK-----------------DKDK  221
6    -----TVV-NGKS------SFDGWKIYINDF-QPTHKIK-----------------DKDK  221
7    -----PVL-NGKS------SFDGWKIYINDF-QPIHKI-------------------KDK  219
8    -----PVI-DGKS------SFDGWKIYINDF-QPTHCI-------------------KDK  219
9    -----PVL-TGKS------SFDGWKIYINDF-QPIHKI-------------------KDK  219
10   -----PVV-DGKA------SFDGWKIYINDF-QPTHCI-------------------KDK  219
11   -----PVV-DGKS------SFDGWKIYINDF-QPTHCI-------------------KDK  219
12   -----PVV-NGKS------SFDGWKIYINDF-QPTHKI-------------------KDK  219
```

```
13  -----PVI-DGKS------SFDGWKIYINDF-QPTHCI-------------------KDK  219
14  -----PEV-NGHT------VFDGWLIYINDM-QPLNCN-------------------DSE  219
15  EEMTFPGP-DGSSRMQQVLKFDGAPVYINDLNQIYACDGSKPDKLIPPEKALNLDGFDPS  260
16  EEMEFTCP-DGTIKFEHVLKFDGAPVYINDLNQVYECGHNHVGAAISPENVLELNGMEPP  260
17  EEMEFACP-DGTTKFEHVLKFDGAPVYINDLNQVYECGHNHVGAAISPENVLELNGMEPP  260
18  EEMEFTSP-DGTAKFEQVLKFDGAPVYINDLNQIYECNDNNTGTPIPSEKISRLNDNEPP  260
19  TDMIFPCP-RNENQVERVLTVDGAPVYVNDLNQNLSGSPDNPAGESVHENPGELG--NSD  257
20  EEMAFPSP-DGSSKMQQVLKFDGAPVYINDLNQIYACDGSKPGKLIPPPKALNLDGFDLP  261
21  TDMIFPCP-RNENQAEQVLTFDGAPVYVNDLNQNLGGSPDAPDGEPVPENPLAPD--SAG  257
22  TDMIFPCP-RNENQMERVLTFDGAPVYINDLNQNFSGSPDNPSGELVHENPQILG--SPG  257
23  TDMIFPCP-RNENQAEQVLTFDGAPVYVNDLNQNLGGSPDAPDGEPVPENPLAPD--SAG  258
24  TDMIFSCP-YNENQAEQVLTFDGAPVYVNDLNQNLGRSPDTPDGESIVENPLAFG--SAG  257
25  TDMIFPCP-RNENHTERVLTVDGAPVYVNDLNQNLDGSPDAPDGEPVPENPLAPD--SAG  257
26  TDMIFPCP-RNENQGERVLTVDGAPVYVNDLNQNLSGSPDTPDGEPVPENPLASD--STG  257
27  EEMEFTCP-DGTTKFEHVLKFDGAPVYINDLNQIFECSHNQVGAAIPPERVLALNGTEPP  260
28  TDMIFSCP-YNENQAEQVLTFDGAPVYVNDLNQNLGRSPDTPDGESIVENPLAFG--SAG  257
29  VTETWTDPVKGRVSAP-ITCFNGTPWYINDLIDDQAG-----------------------  237
30  VTETWTDPVKGRVSAP-ITCFNGTPWYINDLIDDQAG-----------------------  243
31  VKVARPDPIKGMVLEE-IAAFNGTPWYINDLIDDQAS-----------------------  239
32  VKVARPDPIKGMVLEE-IAAFNGTPWYINDLIDDQAS-----------------------  239
33  VKVPRTDPIKGIVLEE-IASFNGTPWFINDLIDDQAN-----------------------  239
34  VDAEWTDPTKGTVRVPQI-AINGTPVYVNDMIATVEDPGP-----------------PAT  254
35  TEMDWFDPAQGRTVKRLVPSINGAPILINDMVAT-DG----------------------S  256
36  -----PVI-DGKS------SFDGWKIYINDF-QPTHCI-------------------KDK  142
37  VPYEWTDPIKGRLTVQVA-SINGTPIFINDMIAT-EDSDP-------------------E  250
38  VMDTWFDPIRGEITAG-ITSFKGTPWYINDLLDDLPG-----------------------  237
39  VEFI-PLQ-DGPA--QKVMIYDGCPILINDA-QPEHVLGGNP------------------  235
40  VETEWGDPLKGTMRAPQI-AINGTPVYINDWIAT------------------------DT  243
41  -------------------NFEDWPICLND--QQPTCSACGVD-----------------  218
42  VLDTWMDPVLGEVTAP-ITAFNGTPWYINDLIDDEAG-----------------------  237
43  VMDTWSDPIHGQITAP-ITAFNGTPWYINDLIDDQAG-----------------------  236
44  VPASWMDPVLGEVTAP-ITAFNGTPWYINDLIDDQAG-----------------------  237
45  VMDTWFDPIRGQVSAG-ITSFKGTPWYINDLIDDLPG-----------------------  237
46  APFRWYNPASRRMEDGSVDAFNGTPWLANDLCNAAGAP---------------------G  231
47  VPFRWYNPAIGGMQEGSVDALNGTPWLANDMLGS--------------------------  227
48  VPFMLYDPASRRMEDDAVDAFNGTPWLANDLCNADGL-----------------------  229
49  APFRWYNPASRRMEDGSVDAFNGTPWLANDLCNAAGTP---------------------G  231
50  VPFMLYNPASRRMEDDTVDAFNGTPWLANDLCNAAGI-----------------------  229
51  EPWTWYHPDKRRMGPGSVDSFNGTPWLVNDFINKSGLD----------------------  226
52  VPWTWYDPFKG-WQQGKVTSFHGVPWLVNTKMNAGLLP----------------------  230
53  TIEMRIEDADGSTRVMPVLAWDGIPVLVDDQVPSDET-------------------SNNY  245
54  VEMEWIDPLTGGKKRQWMNAINGAPILINDMIET-DE----------------------G  237
55  FEVMELPAPGGGTRMVKVPCWDGIPWVIDDNVPANEQP------------------GGTS  247
56  VMETWTDPVQGRMSAP-ITCFNGTPWYINDLIDDQAG-----------------------  237
57  MPWQWYNPSLGRMEESLVTQFNGTPWLINGCMAGEASPK--------------------P  231
58  VEMPFPDPISGGVKMVRVPAINGTPILVNDLIET-TSP---------------------G  241
59  MKATTSEDVDGSRREIPGLAFDGIPVVVDDLVPNDEG-------------------SGSQ  245
60  VQWRWYCPATRMWHDDWISAFRGVPWVINQEMNPGLLP----------------------  231
61  YEMMEIPVAGGGSRMIKVPCWDGVPWVIDDNVPNDEQP------------------SGTN  247
62  AAFKWYNPASRRMEDGSVDAFNGTPWLANDLCNADGT-----------------------  229
63  MPWRWYDPLRG-WQVGKVTTFHGVPWLVNTKMNAGLLA----------------------  230
64  TVLMQVEDADGASRTVSALAFDGIPVVVDDQVPNNET-------------------SNSY  245
65  YMDWEVPRPGGGTRTVRTLCWEGVPVIVDDSVPNDEQP------------------GGTS  247
66  SPWTWYYPAKGSSGPGSVDSFNGTPWLVNDFINKDGHD----------------------  226
67  VDFMWYDPVRRRMTPVKVTAFNGTPWLVNDRMEGKTNPA--------------------A  233
68  VAVVVPDG-NGGLKTVTMMHVNGWLVMWTDFVPIEQFQ------------------GSTV  242
69  VSVAVPDG-NGGLKTISMMHVNGWLVSWTDFVPIEQFQ------------------GSTV  258
70  AEIEVPDPRTGTR-LARVPSIHGVPWYINDAMEVPPEGE---------------------  240
71  ADIEVPDPRTGTR-IARVPSIHGVPWYINDAMEVPPEGE---------------------  291
72  ADIEVPDPRTGTR-IARVPSIHGVPWYINDAMEVPPEGE---------------------  286
73  VEIDAPDPLTGTK-KQWVPSIHGVPWYINDAMEVPPEGE---------------------  240
74  VMIENF--------GRPMLCHNGVPIIVNDFLPLNEVQ------------------GSSS  232
75  VPYRWYNPTVGGMQEGSVDAFNGTPWLANDMLGS--------------------------  227
76  VTETWTDPVKGRVSAP-ITCFNGTPWYINDLIDDQAG-----------------------  172
77  VPWRWYCPATGRWKDGVVPSFNGTPWVINDEMNPGVQP----------------------  231
78  ITVENF--------G-TVRAYDGTPVIINDFLPGDEVQ------------------GTEN  231
79  VTETWTDPVKGRVSAP-ITCFNG-------------------------------------  223
```

```
80   VMMENF--------GRPMLTHNGVPIIVNDFLPSNEVQ------------------GSSS  232
81   IMIENF--------GRAVPGHNGIPIIVNDFLPIDEVQ------------------GTET  233
82   QQLENF--------GRPMLTHNGIPILMNEFLPKNEVQ------------------GASS  232
83   IQIPNF--------GKPVPAYDGVPVIINDFMTADEVQ------------------GSAN  232
84   IQIPNF--------GKPVPAYDGIPVIINDFLTADEVM------------------GSAN  232
85   IQIPNF--------GKPVPAYDGIPVIINDFLTADEVM------------------GSAN  232
86   IMIPNF--------GKPVPAYDGVPVIINDFLTADEVM------------------GSAN  232
87   VETHWADPVKGVV-RGKVISMYGTPWYINAAMTTRPEGT---------------------  231
88   VEMEWVDPLTGGKKRQWMNAINGAPILINDMIET-DG----------------------S  188
89   IQIPNF--------GKPVPAYDGIPVIINDFLTADEVM------------------GSAN  232
90   MMIKNF--------GQAVPCYDGKPVIINDFLPADEVQ------------------GTAD  232
91   VPWRWYDPARGRMVEGSVDAFNGTPWLANDFVN-AGAD---------------------P  186
92   VDAPWADPIRGVA-LGKVVSMYGTPWYINGAMNTRPEGT---------------------  231
93   VETQWADPIRGVV-PGKVVSVYGTPWYINGAMTTRPEGT---------------------  231
94   IMMENF--------GRPMLTHNGVPILVNDFLNVAQ-D------------------GEGK  229
95   IQIANF--------GKPVPAYDGIPVIINDFITADEVQ------------------GSAN  232
96   QMLDNF--------GRPMLTHNGVPIIMNEFSPGTEDQ------------------GTAT  232
97   IIVKDF--------GKALPAFDGTPIIINDFMTADEVQ------------------GTNE  232
98   LQIENF--------GM-MPAHDGIPIIINDFIPVK-AQ------------------GTTA  232
99   IMIPNF--------GRPIPAFDGTPVIINDYMNADETQ------------------GTAE  232
100  LQLDNF--------GV-IPAHDGVPIIINDFIPVK-AQ------------------GTNA  232
101  VVQGEY--------G-PIPAHNGVPIIVNDFLPADEVQ------------------GNSS  232
102  IECVKEDPILGPVRVKQL-CLYGTPWYICDLIQARTVNS--------------------Q  239
103  LQLDNY--------GQ-IPAHDGIPIIINDFIPVK-AQ------------------GTTA  232
104  QMVENF--------GRPMLTHNGVPIVMNEFLPADEVM------------------GTNT  232
105  QQLSNF--------GRPMLTHNGIPIIMNDWIAGDEVK------------------GTNS  232
106  QQLSNF--------GRPMLTHNGIPIIMNDWIAGDEVK------------------GTNS  230
107  QQGLNFD-------GRPVLTHNGIPIIMNEYLDGNENG------------------N---  228
108  QQLSNF--------GRPMLTHNGIPIIMNDWIAGDEVK------------------GTNS  232
109  QQLSNF--------GRPMLTHNGIPIIMNDWIAGDEVK------------------GTNS  232
110  QQLSNF--------GRPMLTHNGIPIIMNDWIAGDEVK------------------GTNS  232
111  VMIKDF--------GM-VMAHNGTPLLRNDFLPGNETL------------------GTGN  231
112  QQLSNF--------GRPMLTHNGIPIIMNDWIAGDEVK------------------GTNS  234
113  QQLSNF--------GRPMLTHNGIPIIMNDWIAGDEVK------------------GTNS  232
114  QQLSNF--------GRPMLTHNGIPIIMNDWIAGDEVK------------------GTNS  232
115  LQIDNF--------GLSVPAHDGIPIIINEFIPGNVAQ------------------GSTA  233
116  VMLKNF--------GHPMLTHNGVPIIVNDYIKVDE--------------------GDES  228
117  QMVENF--------GRPMLAHNGVPIVMNEFLPADEVM------------------GTNT  232
118  QMVENF--------GRPMLTHNGVPIVMNEFLPADEVM------------------GTNT  232
119  VMLKNF--------GHPMLTHNGVPIIVNDYIKVDE--------------------GDES  228
120  IQHPNF--------DVPVLAHNGCPIIINDFMPGDELM------------------GSNS  233
121  QQLSNF--------GRPMLTHNGIPIIMNDWIAGDEVK------------------GTNS  232
122  QQLSNF--------GRPMLTHNGIPIIMNDWIAGDEVK------------------GTNS  232
123  QQLENF--------GRPMLTHNGIPIIMNEFLAADEAQ------------------GTAT  232
124  QQLSNF--------GRPMLTHNGIPIIMNEWIAGDEVK------------------GTNQ  232
125  QQLSNF--------GRPMLTHNGIPIIMNEWIAGDEVK------------------GTNQ  232
126  QQLSNF--------GRPMLTHNGIPIIMNDWIAGDEVK------------------GTNS  232
127  IIVKDF--------GKPLPAFDGTPVIINDYIGADEVQ------------------GAST  232
128  IIVKDF--------GKALPAFDGTPVIINDYITADEVQ------------------GAST  232
129  VEIPNF--------GQAVLAHNGIPILRNDWLANDETM------------------GTNA  232
130  VEIPNF--------GQAVLAHNGIPILRNDWLANDETM------------------GTNA  232
131  VEIPNF--------GQAVLAHNGIPILRNDWLANDETM------------------GTNA  232
132  QQLSNF--------GRPMLTHNGIPIIMNDWIAGDEVK------------------GTNS  232
133  VMEMPN--------GARVIAYEGVPIFRNDYIPTNQTK------------------GGYA  235
134  VEIPNF--------GQAVLAHNGIPILRNDWLANDETM------------------GSNA  232
135  QQLSNF--------GRPMLTHNGIPIIMNDWIAGDEVK------------------GTNS  232
136  VMMENF--------GRPMLTHNGTPIIVNDFI-AKT--------------------GDAG  227
137  VTLPGT--------GISVPAYRRTPIIINDFLPGDELH------------------GTKS  232
138  QQLENF--------GRPMLTHNGIPVIMNEFLAADEAQ------------------GSAN  235
139  IMLPNF--------GHPVPAIDGMPVIINDFIPANEVT------------------GSSS  232
140  VMLPQF--------GKVMLTHNGVPIIVNDFLPVTD--------------------GEGT  229
141  LILPNF--------GRPMLCHNGMPIIVNDYI-GTEAQ------------------GSAD  234
142  TMIENF--------GHPVKAYDGTPVIINDFLPANEVQ------------------GTAN  232
143  VMLPQF--------GKVMLTHNGVPIIVNDFLPVTD--------------------GEGT  229
144  IMIPNF--------GHPVKAYDGTPVIINDFLTANEVQ------------------GSAT  232
145  IMLENF--------GQSIPAYNGKPVIINNYITADETQ------------------GAGD  232
146  VTLPGT--------GINVPAYRRTPIIINDFLPGDEVH------------------AAGT  232
```

```
147  IMMENF--------SRPMLCHNGVPFIVNDFIPK----------------------NEGD  226
148  IMMENF--------GRPMLCHNGVPFIVNDFIPK----------------------NEGD  226
149  IMMENF--------GRPMLCHNGVPFIVNDFIPK----------------------NEGD  226
150  IMIENF--------GRPMLCHNGVPFIVNDFIPK----------------------NDDN  226
151  IMMENF--------GRPMLCHNGIPFIVNDFIPKNGVKVTEDSEQ---------KTEGQE  239
152  IMMENF--------GRPMLCHNGVPFIVNDFIPK----------------------NDDN  226
153  IMIPNF--------GHPVKAYDGTPVIINDFLTADEVQ------------------GSAT  232
154  IMMENF--------GRPMLCHNGVPFIINDFIPK----------------------TEEN  226
155  IMMENF--------GRPMLCHNGIPFIVNDFIPKNGVKVTEYSEQ---------KTEGQE  239
156  IMIENF--------GRPMLCHNGVPFIVNDFIPK----------------------NDDN  226
157  IMIENF--------GRPMLCHNGVPFIVNDFIPK----------------------NDDN  226
158  IMMENF--------GRPMLCHNGIPFIVNDFIPKNGVKVTEDSEQ---------KTEGQE  239
159  IMMENF--------GRPMLCHNGIPFIVNDFIPKNGVKVTEGSEQ---------KTEGQE  239
160  IMMENF--------GRPMLCHNGIPFIVNDFIPKNGVKVTEDSEQ---------KTEGQE  239
161  QMVENF--------GRPMLTHNGVPIVMNEFLPADEVM------------------GTNT  232
162  VMMPAF--------GRPMLTHNGVPILINDFIPLNETT------------------GTSN  239
163  IMMENF--------GRPMLCHNGIPFIVNDFIPKNGVKVTEGSEQ---------KTEGQE  239
164  IMIENF--------GRPMLCHNGVPFIVNDFIPK----------------------NDDN  226
165  IMMENF--------GRPMLCHNGVPFIVNDFIPK----------------------NDDS  226
166  IMMENF--------GRPMLCHNGVPFIVNDFIPK----------------------NDDN  226
167  IMMENF--------GRPMLCHNGVPFIVNDFIPK----------------------NDGD  226
168  IMMENF--------GRPMLCHNGVPFIVNDFIPK----------------------NDDS  226
169  IMMEDF--------GRPMLCHNGVPFIVNDFIPK----------------------NDGD  226
170  IMMENF--------GRPMLCHNGVPFIINDFIP------------------------TDS  224
171  IMMENF--------GRPMLCHNGVPFIVNDFIPK----------------------NDDS  226
172  IMMENF--------GRPMLCHNGIPFIVNDFIPKNGVKVTGDSEQ---------KTEGQE  239
173  IMMENF--------GRPMLCHNGVPFIINDFIP------------------------TDS  224
174  IMMENF--------GRPMLCHNGVPFIINDFIP------------------------TDS  224
175  IMMENF--------GRPMLCHNGVPFIINDFIP------------------------TDS  224
176  IMMENF--------GRPMLCHNGVPFIVNDFIPK----------------------NDGD  226
177  IMMENF--------GRPMLCHNGIPFIVNDFIPKNGVKVTEDSEQ---------KTEGQE  239
178  IMMENF--------GRPMLCHNGIPFIVNDFIPKNGVKVTEDSEQ---------KTEGQE  239
179  IMMENF--------GRPMLCHNGVPFIINDFIP------------------------TDS  224
180  IMMENF--------GRPMLCHNGVPFIVNDFIPK----------------------NDDS  226
181  IMMENF--------GRPMLCHNGVPFIINDFIP------------------------TDA  224
182  IMMENF--------GRPMLCHNGVPFIINDFIP------------------------TDS  224
183  IMMENF--------GRPMLCHNGVPFIINDFIP------------------------TDA  224
184  IMMENF--------GRPMLCHNGVPFIINDFIP------------------------TDS  224
185  IMMENF--------GRPMLCHNGIPFIVNDFIPKNGVKVTEDSEQ---------KTEGQE  239
186  IMMENF--------GRPMLCHNGVPFIVNDFIP------------------------TDS  224
187  IMMENF--------GRPMLCHNGVPFIINDFIP------------------------TDS  224
188  IMMENF--------GRPMLCHNGVPFIINDFIP------------------------TDA  224
189  IMMENF--------GRPMLCHNGVPFIINDFIP------------------------TDS  224
190  V--NAF--------GRRIETYDGIPIAADDFILNTETQ------------------GTSS  231
191  IMMENF--------GRPMLCHNGIPFIVNDFIPKNGVKISEEPEK---------KTEGQE  239
192  MMIENF--------G-MVPAIDGMPVIINDFIRADEVQ------------------GTND  231
193  IMMENF--------GRPMLCHNGVPFIINDFIP------------------------TDS  224
194  IMMENF--------GRPMLCHNGVPFIINDFIP------------------------TDS  224
195  IMMENF--------GRPMLCHNGVPFIINDFIP------------------------TDS  224
196  IMMENF--------GRPMLCHNGVPFIINDFIP------------------------TDS  224
197  IMMENF--------GRPMLCHNGVPFIINDFIP------------------------TDS  224
198  IMMENF--------GRPMLCHNGVPFIINDFIP------------------------TDA  224
199  IMMENF--------GRPMLCHNGVPFIINDFIP------------------------TDS  224
200  IMMENF--------GRPMLCHNGVPFIVNDFIP------------------------TDS  224
201  IMMENF--------DRPMLCHNGVPFIINDFIP------------------------TDA  224
202  ITIKDF--------G-TVKAYDGTPVIINDFLTDNEVQ------------------GTAN  231
203  IMMENF--------GRPMLCHNGVPFIVNDFIP------------------------TDA  224
204  QQLENF--------GRPMLTHNGIPVIMNEWLPANETQ------------------GTSV  233
205  IMMENF--------GRPMLCHNGVPFIINDFIP------------------------TDS  224
206  IMMENF--------GRPMLCHNGVPFIINDFIP------------------------TDS  224
207  IMMENF--------GRPMLCHNGVPFIINDFIP------------------------TDA  224
208  IMMENF--------GRPMLCHNGVPFIVNDFIP------------------------TDA  224
209  IMMENF--------GRPMLCHNGVPFIINDFIP------------------------TDS  224
210  IMMENF--------GRPMLCHNGVPFIINDFIP------------------------TDA  224
211  IMMENF--------GRPMLCHNGVPFIINDFIP------------------------TDS  224
212  IMMENF--------GRPMLCHNGVPFIINDFIP------------------------TDA  224
213  IMMENF--------GRPMLCHNGVPFIINDFIP------------------------TDA  224
```

```
214  IMMENF--------GRPMLCHNGIPFIVNDFIPKNGVKVTEGSEQ---------KTEGQE  239
215  IMMENF--------GRPMLCHNGVPFIINDFIP------------------------TDS  224
216  IMMENF--------GRPMLCHNGVPFIINDFIP------------------------TDS  224
217  IMMENF--------GRPMLCHNGVPFIINDFIP------------------------TDS  224
218  IMMENF--------GRPMLCHNGVPFIINDFIP------------------------TDS  224
219  IMMENF--------GRPMLCHNGVPFIINDFIP------------------------TDS  224
220  IMMENF--------GRPMLCHNGVPFIINDFIP------------------------TDS  224
221  IMMENF--------GRPMLCHNGVPFIINDFIP------------------------TDS  224
222  IMMENF--------GRPMLCHNGVPFIINDFIP------------------------TDS  224
223  IMMENF--------GRPMLCHNGVPFIINDFIP------------------------TDA  224
224  IMMENF--------GRSMLCHNGVPFIVNDFIP------------------------TDA  224
225  IMMENF--------GRPMLCHNGVPFIINDFIP------------------------TDA  224
226  IMMENF--------GRPMLCHNGVPFIINDFIP------------------------TDS  224
227  IMMENF--------GRPMLCHNGVPFIINDFIP------------------------TDA  224
228  QMLPAF--------GHPMLVHQGMPILMNEFIPMGE-D------------------G---  226
229  IMMENF--------GRPMLCHNGVPFIINDFIP------------------------TDS  224
230  QMLPAF--------GHPMLVHQGMPILMNEFIPMGE-D------------------G---  226
231  IMMENF--------GRPMLCHNGVPFIINDFIP------------------------TGA  224
232  IMMENF--------GRPMLCHNGIPFIVNDFIPKNGVKVTEDSEQ---------KTEGQE  239
233  IMMENF--------GRPMLCHNGVPFIINDFIP------------------------TDS  224
234  IMMENF--------GRPMLCHNGVPFIVNDFIP------------------------TDA  224
235  IMMENF--------GRPMLCHNGVPFIVNDFIP------------------------TDA  224
236  IMMENF--------GRPMLCHNGVPFIINDFIP------------------------TDS  224
237  IMMENF--------GRPMLCHNGVPFIINDFIP------------------------TDA  224
238  IMMENF--------GRPMLCHNGVPFIVNDFIP------------------------TDS  224
239  I--DQF--------GQRIVTYDGIPVLVDDFVPDDETL------------------GSGT  231
240  IMMENF--------GRPMLCHNGIPFIVNDFIPKNGVKVTEDSEQ---------KTEGQE  239
241  IMMENF--------GRPMLCHNGVPFIVNDFIP------------------------TDA  224
242  IMMENF--------GRPMLCHNGVPFIINDFIP------------------------TDA  224
243  IMMENF--------GRPMLCHNGVPFIVNDFIP------------------------TDA  224
244  IMMENF--------GRPMLCHNGVPFIINDFIP------------------------TDA  224
245  IMMENF--------GRPMLCHNGVPFIINDFIP------------------------TDA  224
246  IMMENF--------GRPMLCHNGVPFIVNDFIP------------------------TDA  224
247  IMMENF--------GRPMLCHNGVPFIINDFIP------------------------TDA  224
248  IMMENF--------GRPMLCHNGVPFIINDFIP------------------------TDS  224
249  IMMENF--------GRPMLCHNGVPFIINDFIP------------------------TDA  224
250  IMMENF--------GRPMLCHNGVPFIINDFIP------------------------TDA  224
251  IMMENF--------GRPMLCHNGVPFIINDFIP------------------------TDS  224
252  IMMENF--------GRPMLCHNGVPFIINDFIP------------------------TDS  224
253  IMMENF--------GRPMLCHNGVPFIINDFIP------------------------TDS  224
254  IMMENF--------GRPMLCHNGVPFIINDFIP------------------------TDA  224
255  IMMENF--------GRPMLCHNGVPFIINDFIP------------------------TDS  224
256  IMMENF--------GRPMLCHNGVPFIVNDFIP------------------------TDA  224
257  IMMENF--------GRPMLCHNGVPFIVNDFIP------------------------TDA  224
258  IMMENF--------GRPMLCHNGVPFIINDFIP------------------------TDS  224
259  IMMENF--------GRPMLCHNGVPFIINDFIP------------------------TDA  224
260  IMMENF--------GRPMLCHNGVPFIVNDFIP------------------------TDA  224
261  IMMENF--------GRPMLCHNGVPFIINDFIP-----------------------VADS  225
262  IMMENF--------GRPMLCHNGVPFIINDFIP------------------------TDS  224
263  IMLENF--------GRPMLCHNGIPFIINDFIPK----------------------TEEN  226
264  IMMENF--------GRPMLCHNGVPFIINDFIPK----------------------NEDN  226
265  IMMENF--------GRPMLCHNGVPFIVNDFIP-----------------------VSDS  225
266  IMMENF--------GRPMLCHNGVPFIINDFIP------------------------TDS  224
267  IMMENF--------GRPMLCHNGVPFIVNDFIP-----------------------VADS  225
268  IMMENF--------GRPMLCHNGVPFIVNDFIP-----------------------VSDS  225
269  IMMENF--------GRPMLCHNGVPFIVNDFIP------------------------TDA  224
270  QMLPAF--------GHHMLVHQGMPILMNEFIPMET-D------------------G---  226
271  IMMENF--------GRPMLCHNGVPFIVNDFIP------------------------TDA  224
272  IMMENF--------GRPMLCHNGVPFIVNDFIP------------------------TDA  224
273  IMMENF--------GRPMLCHNGVPFIVNDFIP-----------------------VADS  225
274  IMMENF--------GRPMLCHNGVPFIVNDFIP-----------------------VADS  225
275  IMMENF--------GRPMLCHNGVPFIINDFIP------------------------TDS  224
276  IMMENF--------GRPMLCHNGVPFIVNDFIP------------------------TDA  224
277  IMMENF--------GRPMLCHNGVPFIINDFIP------------------------TDS  224
278  IMMENF--------GRPMLCHNGVPFIINDFIP-----------------------VADS  225
279  IMMENF--------GRPMLCHNGVPFIINDFTP------------------------TDA  224
280  IMMENF--------GRPMLCHNGVPFIVNDFIP------------------------TDA  224
```

```
281 QQLSNF--------GRPMLTHNGIPIIMNDWIAGDEVK------------------GTNS 232
282 IMMENF--------GRPMLCHNGVPFIVNDFIP-----------------------VADS 225
283 IMMENF--------GRPMLCHNGVPFIINDFIP------------------------TDA 224
284 IMMENF--------GRPMLCHNGVPFIINDFIP------------------------TDS 224
285 IMMENF--------GRPMLCHNGVPFIINDFIP------------------------TDS 224
286 IMMENF--------GRPMLCHNGVPFIVNDFIP------------------------TDS 224
287 IMMENF--------GRPMLCHNGVPFIVNDFIP-----------------------VVDS 225
288 IMMENF--------GRPMLCHNGVPFIVNDFIP------------------------TDA 224
289 IMMENF--------GRPMLCHNGVPFIVNDFIP-----------------------VADS 225
290 IMMENF--------GRPMLCHNGVPFIINDFIP------------------------TDS 224
291 IMMENF--------GRPMLCHNGVPFIINDFIP------------------------TDS 224
292 IMMENF--------GRPMLCHNGVPFIINDFIP------------------------TDS 224
293 IMMENF--------GRPMLCHNGVPFIVNDFIP------------------------TDA 224
294 IMMENF--------GRPMLCHNGVPFIVNDFIP------------------------TDA 224
295 IMMENF--------GRPMLCHNGVPFIVNDFIP-----------------------VADS 225
296 IMMENF--------GRPMLCHNGVPFIVNDFIP-----------------------VADS 225
297 IMMENF--------GRPMLCHNGVPFIVNDFIP-----------------------VADS 225
298 IMMENF--------GRPMLCHNGVPFIINDFIP------------------------TDS 224
299 IMMENF--------GRPMLCHNGVPFIVNDFIP-----------------------VADS 225
300 IMMENF--------GRPMLCHNGVPFIINDFIP------------------------TDS 218
301 IMIENF--------GRPMLCHNGVPFIVNDFIP-----------------------VADS 225
302 IMMENF--------GRPMLCHNGVPFIVNDFIP-----------------------VADS 225
303 IMMENF--------GRPMLCHNGVPFIVNDFIP------------------------TDA 224
304 IMMENF--------GRPMLCHNGVPFIVNDFIP-----------------------VVDS 225
305 IMMENF--------GRPMLCHNGVPFIVNDFIP-----------------------VADS 225
306 IMMENF--------GRPMLCHNGVPFIVNDFIP-----------------------VADS 225
307 IMMENF--------GRPMLCHNGVPFIVNDFIP------------------------ADA 224
308 IMMENF--------GRPMLCHNGVPFIVNDFIP-----------------------VADS 225
309 IMMENF--------GRPMLCHNGVPFIVNDFIP------------------------TDA 224
310 IMMENF--------GRPMLCHNGVPFIVNDFIP------------------------TDS 224
311 IMMENF--------GRPMLCHNGVPFIVNDFIP------------------------ADA 224
312 IMMENF--------GRPMLCHNGVPFIVNDFIP-----------------------VVDS 225
313 IMMENF--------GRPMLCHNGVPFIINDFIP------------------------TDA 224
314 IMMENF--------GRPMLCHNGVPFIVNDFIP------------------------TDS 224
315 IMMENF--------GRPMLCHNGVPFIINDFIP------------------------TDS 224
316 IMMENF--------GRPMLCHNGVPFIVNDFIP-----------------------VADS 225
317 IMMENF--------GRPMLCHNGVPFIINDFIPK----------------------NEDN 226
318 IMMENF--------GRPMLCHNGVPFIVNDFIP-----------------------VADS 225
319 IMMENF--------GRPMLCHNGVPFIVNDFIP-----------------------VADS 225
320 IMMENF--------GRPMLCHNGVPFIVNDFIP------------------------TDA 224
321 IMMENF--------GRPMLCHNGVPFIVNDFIP-----------------------VADS 225
322 IMMENF--------GRPMLCHNGVPFIVNDFIP------------------------TDA 224
323 IMMENF--------GRPMLCHNGVPFIVNDFIP------------------------TDS 224
324 IMMENF--------GRPMLCHNGVPFIINDFIP------------------------TDS 224
325 IMMENF--------GRPMLCHNGVPFIINDFIP------------------------TDA 224
326 IMMENF--------GRPMLCHNGVPFIVNDFIP-----------------------VADS 225
327 IMMENF--------GRPMLCHNGVPFIVNDFIP-----------------------VADS 225
328 IMMENF--------GRPMLCHNGVPFIVNDFIP-----------------------VADS 225
329 IMMENF--------GRPMLCHNGVPFIVNDFIP------------------------TDA 224
330 IMMENF--------GRPMLCYNGVPFIVNDFIP------------------------TDA 224
331 IMMENF--------GRPMLCHNGVPFIVNDFIP-----------------------IADS 225
332 IMMENF--------GRPMLCHNGVPFIVNDFIP------------------------TDA 224
333 IMMENF--------GRPMLCHNGVPFIVNDFIP------------------------TDA 224
334 IMMENF--------GRPMLCHNGVPFIINDFIPK----------------------SEDN 226
335 IMMENF--------GRPMLCHNGVPFIVNDFIP------------------------TDS 224
336 IMMENF--------GRPMLCHNGVPFIVNDFIP------------------------TDA 224
337 IMMENF--------GRPMLCHNGVPFIVNDFIP------------------------TDS 224
338 IMMENF--------GRPMLCHNGVPFIVNDFIP-----------------------VADS 225
339 IMMENF--------GRPMLCHNGVPFIVNDFIP------------------------ADA 224
340 IMMENF--------GRPMLCHNGVPFIVNDFIP-----------------------VADS 225
341 IMMENF--------GRPMLCHNGIPFIVNDFIP------------------------TDA 224
342 IMMENF--------GRPMLCHNGIPFIVNDFIPKNGVKVTEDSEQ---------KTEGQE 239
343 IMMENF--------GRPMLCHNGVPFIVNDFIP------------------------TDA 224
344 IMMENF--------GRPMLCHNGVPFIVNDFIP------------------------TDA 224
345 IMMENF--------GRPMLCHNGVPFIVNDFIP-----------------------VADS 225
346 IMMENF--------GRPMLCHNGVPFIVNDFIP------------------------TDA 224
347 IMMENF--------GRPMLCHNGVPFIINDFIP------------------------TDA 224
```

```
348  IMMENF--------GRPMLCHNGIPFIINDFIPK----------------------NEDG  226
349  IMMENF--------GRPMLCHNGVPFIVNDFIP------------------------TDA  224
350  IMMENF--------GRPMLCHNGVPFIVNDFIP------------------------TDS  224
351  QQLSNF--------GRPMLTHNGIPIIMNDWIAGDEVK------------------GTNS  213
352  IMMENF--------GRPMLCHNGVPFIVNDFIP------------------------TDA  224
353  VITLPD--------GSQIPAYRNIPIFRNDYIPTDQTK------------------GATS  236
354  IMMENF--------GRPMLCHNGVPFIVNDFIP-----------------------VADS  222
355  IMMENF--------GRPMLCHNGVPFIINDFIPK----------------------SEDN  226
356  IMMENF--------GRPMLCHNGVPFIVNDFIP------------------------TDA  224
357  IMMENF--------GRPMLCHNGVPFIVNDFIP-----------------------VADS  225
358  IMMENF--------GRPMLCHNGVPFIVNDFIS------------------------TDA  224
359  IMMENF--------GRPMLCHNGVPFIVNDFIP-----------------------VADS  225
360  IMMENF--------GRPMLCHNGVPFIVNDFIP------------------------ADA  224
361  IMMENF--------GRPMLCHNGVPFIVNDFIPL----------------------TTEK  226
362  IMMENF--------GRPMLCHNGVPFIINDFIP-----------------------VADS  225
363  IMMENF--------GRPMLCHNGVPFIVNDFIP------------------------TDA  224
364  IMMENF--------GRPMLCHNGVPFIVNDFIP------------------------ADA  224
365  IMMENF--------GRPMLCHNGVPFIVNDFIP------------------------TDA  224
366  IMMENF--------GRPMLCHNGVPFIVNDFIP------------------------TDA  224
367  IMLENF--------GRPMLCHNGIPFIINDFIPK----------------------TEDG  226
368  IMMENF--------GRPMLCHNGIPFIINDFIPK----------------------GEDG  226
369  IMMENF--------GRPMLCHNGVPFIVNDFIP------------------------TDA  224
370  IMMENF--------GRPMLCHNGVPFIVNDFIP------------------------TDS  224
371  IMMENF--------GRPMLCHNGVPFIVNDFIP------------------------ADA  224
372  IMMENF--------GRPMLCHNGVPFIVNDFIP------------------------ADA  224
373  IMMENF--------GRPMLCHNGVPFIVNDFIP------------------------TDA  224
374  IMMENF--------GRPMLCHNGVPFIINDFIP------------------------TDS  224
375  IMMENF--------GRPMLCHNGVPFIVNDFIP------------------------ADA  224
376  IMMENF--------GRPMLCHNGVPFIVNDFIP------------------------TDA  224
377  V--DQF--------GQRVLFYDGIPVLVDDFVPDNETL------------------GTGT  231
378  IMMENF--------GRPMLCHNGVPFIVNDFIP------------------------TDA  224
379  IMMENF--------GRPMLCHNGVPFIVNDFIP------------------------TDS  224
380  IMMENF--------GRPMLCHNGIPFIINDFIPK----------------------TEDG  226
381  IMMENF--------GRPMLCHNGIPFIINDFIPK----------------------TEDG  226
382  VFELPS--------GKNVPAYSGIPILRNDWIPINQTK------------------GSGT  235
383  IMMENF--------GRPMLCHNGIPFIINDFIPK----------------------SEDG  226
384  IMMENF--------GRPMLCHNGIPFIINDFIPK----------------------SEDG  226
385  IMMENF--------GRPMLCHNGVPFIVNDFIP------------------------TDA  224
386  IMMENF--------GRPMLCHNDVPFIVNDFIP------------------------TDA  224
387  IMIPNF--------GLALPAHNGVPIIINDFMPTTENV------------------GGTA  234
388  IMMENF--------GRPMLCHNGIPFIINDFIPK----------------------TEDG  226
389  IMMENF--------GRPMLCHNGVPFIVNDFIP-----------------------VADS  225
390  VVEMPN--------GTRIMAYEGVPIFRNDYIPVNQAK------------------GGQA  235
391  IMMENF--------GRPMLCHNGIPFIINDFIPK----------------------TEDG  226
392  IMMENF--------GRPMLCHNGVPFIVNDFIP------------------------TDA  224
393  IMMENF--------GRPMLCHNGVPFIVNDFIP------------------------TDA  224
394  IMMENF--------GRPMLCHNGIPFIINDFIPK----------------------TEDG  226
395  IMMENF--------GRPMLCHNGIPFIINDFIPK----------------------TEDG  226
396  IMIENF--------GRPMLCHNGIPFIVNDFIPKNGVTI-EGSEE---------KAEGTE  238
397  VYTMAS--------GRQVPAYRGIPLFRNDWIPINQTQ------------------GSAT  239
398  IMMENF--------GRPMLCHNGVPFIINDFIP------------------------TDS  224
399  IMMENF--------GRPMLCHNGVPFIINDFIPK----------------------DTAN  226
400  IMMENF--------GRPMLCHNGIPFIINDFIPK----------------------TEDG  226
401  IMMENF--------GRPMLCHNGIPFIINDFIPK----------------------TEDG  226
402  IMMENF--------GRPMLCHNGIPFIINDFIPK----------------------TEDG  226
403  IMMENF--------GRPMLCHNGVPFIVNDFIP-----------------------VADS  225
404  IMMENF--------GRPMLCHNDVPFIVNDFIP-----------------------VADS  225
405  IMMENF--------GRPMLCHNGVPFIINDFIP------------------------TDS  224
406  IMMENF--------GRPMLCHNGIPFIINDFIPK----------------------TEDS  226
407  IMMENF--------GRPMLCHNGVPFIINDFIP-----------------------VADS  225
408  IMMENF--------GRPMLCHNGVPFIVNDFIS------------------------TDA  224
409  IMMENF--------GRPMLCHNGIPFIINDFIPK----------------------TEDG  226
410  IMMENF--------GRPMLCHNGIPFIINDFIPK----------------------TEDG  226
411  IMMENF--------GRPMLCHNGIPFIINDFIPK----------------------TEDS  226
412  IMMENF--------GRPMLCHNGIPFIINDFIPK----------------------TEDG  226
413  IMMENF--------GRPMLCHNGVPFIINDFIP------------------------TDA  224
414  IMMENF--------GRPMLCHNGIPFIINDFIPK----------------------SEDG  226
```

```
415  IMMENF--------GRPMLCHNGIPFIINDFIPK----------------------TEDG  226
416  IMMENF--------GRPMLCHNGIPFIINDFIPK----------------------TEDG  226
417  IMIENF--------GRPMLCHNGIPFIVNDFIPKNGVTI-EGSEE---------KAEGTE  238
418  IMMENF--------GRPMLCHNGVPFIVNDFIL------------------------TDA  224
419  MMVKNF--------GMPIKCYDGTPVIINDFIPADEKQ------------------GSAT  232
420  IMMENF--------GRPMLCHNGIPFIINDFIPK----------------------SEDG  226
421  IMMENF--------GRPMLCHNGVPFIINDFIP------------------------TDS  224
422  IMMENF--------GRPMLCHNGIPFIVNDFIPKNGVKVTEDSEQ---------KTEGQE  239
423  IMIENF--------GRPMLCHNGIPFIVNDFIPKNGVTI-EGSEE---------KAEGTE  238
424  IMMENF--------GRPMLCHNGIPFIINDFIPK----------------------TEDG  226
425  IMMENF--------GRPMLCHNGIPFIVNDFIPKNGVKVTEDSEQ---------KTEGQE  239
426  IMMENF--------GRPMLCHNGIPFIVNDFIPKNGVKVTEDSEQ---------KTEGQE  239
427  IMMENF--------GRPMLCHNGIPFIVNDFIPKNGVKVTEDSEQ---------KTEGQE  239
428  IMMENF--------GRPMLCHNGIPFIINDFIPK----------------------TEDG  226
429  IMMENF--------GRPMLCHNGVPFIINDFIP------------------------TDS  224
430  IMMENF--------GRPMLCHNGIPFIVNDFIPKNGVKVTEDSEQ---------KTEGQE  239
431  IMMENF--------GRPMLCHNGIPFIINDFIPK----------------------TEDD  226
432  VYQMPS--------GETVIAYSGVPIFRNDYLATNQVK------------------GSGS  235
433  IMMENF--------GRPMLCHNGVPFIINDFIP------------------------TDA  224
434  IMIENF--------GRPMLCHNGVPFIVNDFIP-----------------------VADS  225
435  IMMENF--------GRPMLCHNGVPFIVNDFIP-----------------------VADS  225
436  IMMENF--------GRPMLCHNGIPFIINDFIPK----------------------SEGG  226
437  IMMENF--------GRPMLCHNGVPFIINDFIP------------------------NEG  224
438  IMMENF--------GRPMLCHNGIPFIINDFIPK----------------------SEDG  226
439  QMLPAF--------GHHMLVHQGMPILMNEFIPMAD-D------------------G---  226
440  IMMENF--------GRPMLCHNGVPFIINDFIP------------------------TDS  224
441  V--DQF--------GQRVLFYDGIPVLVDDFVPDNETL------------------GTGT  231
442  IMIENF--------GRPMLTHNGVPFIVNDFIPKTG--------------------EAKS  227
443  IMMENF--------GRPMLCHNGVPFIVNDFIP------------------------TDA  224
444  TLTLPS--------GEETLVYEGVPIFRNDWVPTNQTQ------------------GSST  235
445  VFTLPS--------GKEMLAYSGVPIFRNDYIPVNQTK------------------GTGS  235
446  IMMENF--------GRPMLCHNGVPFIINDFIP------------------------TDS  224
447  IMMENF--------GRPMLCHNGVPFIINDFIP------------------------TDS  224
448  V--DQF--------GRRALFYDGIPILVDDFIPDNETL------------------GTGT  229
449  IMMENF--------GRPMLCHNGVPFIINDFIP------------------------TDS  224
450  IMMENF--------GRPMLCHNGVPFIVNDFIP------------------------TDA  224
451  V--DQF--------GQRIVTYDGIPVLVDDFVPEDETL------------------GTGN  231
452  V--DAF--------GRRALFYDGIPIIVDDFIPIDETL------------------GAGS  231
453  IMMENF--------GRPMLCHNGIPFIINDFIPK----------------------SEDG  226
454  VYTMAS--------GRQVPAYRGIPLFRNDWIPINQTQ------------------GSAS  239
455  VEIPNF--------GQPVLAHNGIPILRNDWLSNSETM------------------GSNS  232
456  IMMENF--------GRPMLCHNGVPFIVNDFIP-----------------------VADS  225
457  IMMENF--------GRPMLCHNGVPFIINDFIP------------------------TDS  224
458  IMMENF--------GRPMLCHNGIPFIINDFIPK----------------------TEDG  226
459  IQHKNF--------DNPVLAHNGTPIIVNDFLPATEAK------------------GTGT  232
460  IMMENF--------GRPMLCHNGVPFIINDFIP------------------------TDS  224
461  VYVMAS--------GRSVPAYRGIPIFRNDWIPINQTQ------------------GTSA  239
462  IMMENF--------GRPMLCHNGVPFIINDFIP------------------------TDS  224
463  MPWRWYCPAKRHWVDGKVPSFNGTPWVINDEMNPGVLP----------------------  145
464  IMMENF--------GRPMLCHNGIPFIVNDFIPKNGVKVTEDSEQ---------KTEGQE  239
465  IMMENF--------GRPMLCHNGVPFIINDFIP------------------------TDS  224
466  IMMENF--------GRPMLCHNGVPFIINDFIP------------------------TDS  224
467  IMMENF--------GRPMLCHNGVPFIINDFIP------------------------TDA  224
468  IMMENF--------GRPMLCHNGVPFIINDFIP------------------------TDA  224
469  VMVENF--------GRPMLTHNGVPFIINDFIPVAD--------------------NGGT  227
470  IMMENF--------GRPMLCHNGVPFIINDFIP------------------------TDS  224
471  VVTLPS--------GAEVPAYRGVPIFRNDFIPVNQTV------------------GTST  236
472  QMMENF--------GRPMLTHNGIPIIMNEFLSGAEVK------------------GTSI  242
473  VVTLPS--------GAEVPAYRGVPIFRNDFIPVNQTV------------------GTST  236
474  IMMENF--------GRPMLCHNGVPFIINDFIP------------------------TDA  224
475  IMMENF--------GRPMLCHNGVPFIINDFIP------------------------TDS  224
476  VYTMAS--------GRTVPAYRGIPIFRNDWIPINQTQ------------------GTSA  239
477  VVTLPS--------GRQVPAYRGVPIFRNDYIATNQTK------------------GTAT  235
478  TLELPS--------GEEVLEYEGVPVFRNDWMPTNQTQ------------------GTST  235
479  IYTMPS--------GRQVPAYRGIPIFRNDWIPINQTQ------------------GTSV  239
480  VVTLPS--------GAEVPAYRGVPIFRNDYIPTNQTK------------------GTGT  236
481  IMMENF--------GRPMLCHNGVPFIINDFIP------------------------TDS  224
```

```
482  IMMENF--------GRPMLCHNGVPFIINDFIP------------------------TDS   224
483  IMIENF--------GRPMLTHNGIPFLVNDFIPVAG--------------------DGNA  227
484  IMMENF--------GRPMLCHNGVPFIINDFIP------------------------TDS   224
485  V--DAF--------GRRALFYDGIPIIVDDFIPIDETL------------------GAGS  231
486  IMIENF--------GRPMLTHNGVPFIVNDFIPVVG--------------------DAKA  227
487  IMMENF--------GRPMLCHNGIPFIINDFIPK----------------------TEDG  226
488  IMIENF--------GRPMLTHNGVPFLVNDFIPKTG--------------------DTKA  227
489  IMIENF--------GRPMLTHNGVPFIVNDFIPVVG--------------------DTKA  227
490  VYTMAS--------GRTVPAYRGIPIFRNDWIPITQTQ------------------GTAT  239
491  IMMENF--------GRPMLCHNGVPFIINDFIP------------------------TDS   224
492  TLKLPS--------GEEVLEYEGVPVFRNDWMPTNQTQ------------------GSST  235
493  IMMENF--------GRPMLCHNGVPFIVNDFIP-----------------------VVDS  225
494  V--DAF--------GRRALFYDGIPILVDDFISDSETL------------------GSGT  231
495  IMMENF--------GRPMLCHNGVPFIVNDFIP-----------------------VADS  225
496  IMMENF--------GRPMLCHNGVPFIVNDFIP-----------------------VADS  225
497  VLELPS--------GRRVPVYRDIPIFRNDFIPTDQTV------------------GTSD  236
498  IMMENF--------GRPMLCHNGVPFIINDFIP------------------------TDA   224
499  IMIENF--------GRPMLTHNGVPFLVNDFIPKTG--------------------DTKA  227
500  VYTMAS--------GRTVPAYRGIPIFRNDWIPITQTQ------------------GTAT  239
```

```
               370       380       390       400       410       420
1    L-TT----NIWFASLG--------MDALHGIIPEN--VGDNMFVTRNT------------  254
2    L-TT----NIWFASLG--------MDALHGIIPEN--VGDNMFVTRNT------------  254
3    P-TT----NIWFASLG--------MDALHGIIPEN--VGDNMFVTRKT------------  254
4    P-TT----NIWFASLG--------MDALHGIIPEN--VGDNMFVTRKT------------  254
5    P-TT----NIWFASLG--------MDALHGIIPEN--VGDNMFVTRKT------------  254
6    P-TT----NIWFASLG--------MDALHGIIPEN--VGDNMFVTRKT------------  254
7    P-TT----NIWFASLG--------MDALHGIIPEN--VGDNMFVTRKT------------  252
8    S-TT----NIWFASLG--------TDALHGIIPEN--VGDNMFVTRKT------------  252
9    P-TT----NIWFASLG--------IDALHGIIPEN--VGDNMFVTRKT------------  252
10   S-TT----NIWLASLG--------TDALHGIIPEN--VGDNMFVTRKT------------  252
11   L-TT----NIWFASLG--------MDALHGIIPEN--VGNNMFVTRKT------------  252
12   P-AT----NIWFASLG--------MDALHGIIPEN--VGDNMFVTRKT------------  252
13   S-TT----NIWFASLG--------TDALHGIIPEN--VGDNMFVTRKT------------  252
14   P-TT----NIWFVTLG--------PGGMHGIIPES--VGGNMFVTRQT------------  252
15   V-AT----NIWFFVLG--------QGNLHGIMPEK--ARS--IITRST------------  291
16   L-AT----NIWFLSLG--------PGNLHGIKPEK--AQN--IITRST------------  291
17   L-AT----NIWFLSLG--------PGNLHGIKPEK--AQN--IITRST------------  291
18   L-AT----NIWFFSLG--------PGSLHGIKPEK--AQN--IITRST------------  291
19   V-GT----NIWFFTLG--------ENNLHGMTPAA--LDRDLFVTRST------------  290
20   V-AT----NIWFFVLG--------QGNLHGIMPEK--AQS--IITRST------------  292
21   T-GT----NIWFFSMG--------ENNLHGMTPAA--LDTGLFVTRST------------  290
22   V-GV----NIWFFSLG--------ENNLHGMTPAA--LDTGLFVTRST------------  290
23   T-GT----NIWFFSMG--------ENNLHGMTPAA--LDTGLFVTRST------------  291
24   I-GT----NIWFFSMG--------ENNLHGMTPAA--LDTGLFVTRST------------  290
25   I-GT----NIWFFSMG--------ENNLHGMTPAA--LDTGLFVTRST------------  290
26   I-GT----NIWFFSMG--------ENNLHGMTPAA--LDTGLFVTRST------------  290
27   L-AT----NIWFFSLG--------PGSLHGIKPEK--AQS--IITRST------------  291
28   I-GT----NIWFFSMG--------ENNLHGMTPAA--LDTGLFVTRST------------  290
29   A-RG----IVFFMVLG-DDRGPGHFRGISGIVPRG--RERNMFIKRQVDGVVGD--IVVP  287
30   A-RG----IVFFMVLG-DDRGPGHFRGISGIVPKG--RERNMFIKRQVDGVVGD--IVVP  293
31   S-TG----IVFFMVLG-DDKGPGHFRGITGIVPKG--RERNMFIKRQTDGHTD-------  284
32   S-RG----IVFFMVLG-DDKGPGHFRGITGIVPKG--RERDMFIKRQTAGHTD-------  284
33   S-TG----IVFFMVLG-DDKGPGHFRGITGIVPKG--RERDMFIKRQTDGVVSM--TLDP  289
34   S-LT----KIYFMVLG-DSGEAGPTRGITGIVPGP--LKDTMFIRRESSEPV--------  298
35   N-LT----RIYFMVVG-ENCDRG-IHGVTGIVPLD--LKDKLFVRREAAEP---------  298
36   S-TT----NIWFASLG--------TDALHGIIPEN--VGDNMFVTRKT------------  175
37   F-YT----RIYFMVLG-DACESGPVRGITGIVPGP--LKDTLFIRRESTEPA--------  294
38   N-QG----IIYFMVLG-DDEGPGHERGIVGLVPRD--LADTWFIKREANGVVGT--QVIT  287
39   --VT----NIWFTYTG--------PNGLFGIVPHL--KDGSLFRKRQV------------  267
40   N-LT----KIYFMVLG-DACEAGPVRGITGIVPGP--IKPTMFVRHETAEP---------  286
41   --TT----NMWFVSLG--------LNGLCGIIPPK--ENGSMFRIRQV------------  250
42   E-TG----IIYFMVLG-DDGGPGHSRGITGLVPQG--MRDRWFVRRDANGLVGTGEATVG  289
```

```
43   E-TG----IIYFMVLG-DDGGPGHYRGITGLVPKK--LEHGWFVKRESNGVSGTGEALVT  288
44   E-TG----IIYFMVLG-DDGGPGHYRGITGLVPQG--FENRWFVRRDANGLVGTGEATVG  289
45   D-QG----IIYFMVLG-DDEGPGHERGIVGLVPHD--LSDTWFIKREANGVIGT--QTIT  287
46   G-VE----RTYFMVVG-DDGKSGPTRGVTGIVPAH--LGSEMFIKRTVPALV--------  275
47   T-PE----RTYFMVVG-DDGNAGPTRGVTGIVPAH--LGRNMFIKRTVPAIISVE-----  274
48   G-TE----RTYFMVVG-DDGKAGPTRGVTGIVPAN--LGREMFIKRTVPALV--------  273
49   G-VE----RTYFMVVG-DDGKAGPTRGVTGIVPAH--LGREMFIKRTVPALV--------  275
50   G-TE----RTYFMVVG-DDGKAGPTRGVTGIVPAH--MGREMFIKRTVPALL--------  273
51   --GE----YIYFMVTG-DDGGKGPTRGVTGILPRQ--QMSNPFNKRVVQGIYNPD-----  272
52   V-DR----RIYFMVMG-DDGGPGHTRGITCIRPAD--LMNRRYIKRAVNGVPDF------  276
53   T-------SIYALVLG-RE-------GIYGVIPEG--FQGRMIRANRA------------  276
54   N-LT----RIYMMVVG-EVCDRQ-IHGVTGIVPKD--LENKLFIRREAAEP---------  279
55   T-------SVYALVLG-QE-------GLHGIVPAG--TKNSMFRVARS------------  278
56   T-RG----IVFFMVLG-DDKGPGHFRG---------------------------------  258
57   S-NQ----RIYFMVLG-DDGGEGTTRGVTGIVPQH--LISWPFVKRVTNGTHTFD-----  278
58   N-LT----RIYMMVMG-EDCERQ-IYGVTGIIPKD--LEGKLFIRRESAEP---------  283
59   T-------SIYALVVG-RE-------GLYGVIPRG--FGGRMFRIQAG------------  276
60   A-DR----RIYFMVLG-DDDGKGPTRGLTRMVPAG--MEYNPFILRMTNGVPDF------  277
61   T-------SVYALVLG-QD-------GLHGIVPQG--TKDSMFRVART------------  278
62   G-TE----RTYFMVVG-DDGKASPTRGVTGIVPAH--LGSEMFIKRTVPALLPGP-----  276
63   P-DR----RIYFIVMG-DDGGKGPTRGLTGIRPAD--MMNRPYVKRAVNGVPDF------  276
64   T-------SIYALVLG-RE-------GIYGVIPRG--SEGRMIQAARV------------  276
65   T-------SVYALALG-RE-------GLHGIVPRG--TRESMFRVSKS------------  278
66   --GE----YIFFMVTG-DDGGQGPTRGVTGITPRQ--QVGRMFNKRTVQGIFAPD-----  272
67   G-AQ----RIYFMVLG-DDGSSGPTRGVTGIVPQE--LLHSMFVKRVVPAAYNIE-----  280
68   T-------KIWFFKFG--------RRHIHGIVPASG-GRRSMFKIRS-------------  273
69   T-------KIWFVKLG--------RRHIHGIVPASS-GTRSMFKVRS-------------  289
70   --------NVYFMVMG-DNGHHTPGHGLQLIMPQV--RRGDMFVKRHIPITPSND-----  284
71   --------NVYFMVMG-DNGHHTPGHGLQLILPQV--RRGDMFVKRHIPITPSND-----  335
72   --------NVYFMVMG-DNGHHTPGHGLQLILPQV--RRGDMFVKRHIPITPSND-----  330
73   --------NVYFMVLG-DNGHHTPGHGLQLIMPAA--RRGDMFVKRHIPITPTAD-----  284
74   T-TC----SIYAARFN-EAD------GLHGIYGGS---SAGIRIEDIG------------  265
75   T-PE----RTYFMVVG-DDGGAGPTRGVSS--PRH-------------------------  254
76   A-RG----IVFFMVLG-DDRGPGHFRGISGIVPRG--RERNMFIKRQVDGVVGD--IVVP  222
77   A-DR----RIWFMVLG-DDGGKGHTRGLTRIVPAG--MERSPFILRMTNGVPDF------  277
78   N-SC----SIYAMRLN-TAD------GFHGIYGGD---SAGLVFEDLG------------  264
79   ------------------------------------------------------------  223
80   A-TA----SIYAMRLN-EVD------GLHGIFGGG---NAGFVVEDIG------------  265
81   A-AT----SIYAMRLN-EVD------GLHAIYGGS---SAGIVVEDIG------------  266
82   A-TG----SIYAVRLN-EVD------GLHGIYGGS---NAGFSVEDIG------------  265
83   A-TC----SIYALRLN-EAD------GFHGIFGGS---SAGIQFEEIG------------  265
84   E-TC----SIYALRLN-EAD------GFHGIYGGS---SAGIQFEEIG------------  265
85   A-TC----SIYALRLN-EAD------GFHGIYGGA---SAGIQFEEIG------------  265
86   A-TC----SIYALRLN-EAD------GFHGIYGGS---SAGIQFEEIG------------  265
87   --------NIYFMVLG-DNGEARPGHGLTMIMPAE--RMGNPYVEREWPMLKTDG-----  275
88   N-LT----RIYMMVVG-EVCDRQ-IHGVTGIVPKD--LENKYFIRREAHEP---------  230
89   A-TC----SVYALRLN-EAD------GFHGIYGGP---SAGIQFEEIG------------  265
90   A-TC----SIYALRLN-ETD------GFHGIYGGP---NAGIQVESIG------------  265
91   T-EE----RTFFMVMG-DDGGQGPTRGVTGIVPAA--QGRNLFNRRRIEGVFAAD-----  233
92   --------NIYFMVLG-DNGEARPGHGLTMIMPAE--RRGNPYVEREWPMLKTDG-----  275
93   --------NIYFMVLG-DNGEARPGHGLTMIMPAE--RRGNPYIEREWPMLKTDG-----  275
94   R-TA----PIFAARLN-ETD------GLHGIFGGA---SAGVRMEEIG------------  262
95   E-TT----SIYALRLN-EAD------GFHGIFGGP---SAGIQFEDIG------------  265
96   D-TC----SVYALRLN-ELD------GLHGIYGGG---NAGIVVENLG------------  265
97   T-TC----SIYALRLN-EVD------GIHGLYGGA---SAGLRVEEIG------------  265
98   N-TC----SVYALRLN-EVD------GLHGLYGGA---SAGIRVEDIG------------  265
99   N-TC----SIYALRLN-EAD------GFHGIYGGA---SAGLRVEEIG------------  265
100  N-TT----SIYAVRLN-EVD------GLHGLYGGA---SAGVRVEDIG------------  265
101  A-CC----SIYAMRLN-VAD------GLHGLYGGP---ELGIRIEDVG------------  265
102  T-VS----NIYMMVLGANNKDPQGPFGVTGIIPKQNVQPDTMFMRRESNIVY--------  286
103  N-TC----SVYALRLN-EVD------GLHGLYGGA---SAGIRVEDIG------------  265
104  K-TC----SIYAIRAN-EVD------GLHGIYGGG---PAGIVVENIG------------  265
105  K-TT----SIYAVRLN-EVD------GLHGLYGGG---NAGIVVEDIG------------  265
106  K-TT----SIYAVRLN-EVD------GLHGLYGGG---NAGIVVEDIG------------  263
107  --TC----SVYAVRMN-ELD------GLHGIYGGD---NAGIVVENIG------------  260
108  K-TT----SIYAVRLN-EVD------GLHGLYGGG---NAGIVVEDIG------------  265
109  K-TT----SIYAVRLN-EVD------GLHGLYGGG---NAGIVVEDIG------------  265
```

```
110  K-TT----SIYAVRLN-EVD------GLHGLYGGG---NAGIVVEDIG------------  265
111  N-LC----SVYAARFN-ESD------GLHAIYGGD---NAGIVVEDIG------------  264
112  K-TT----SIYAVRLN-EVD------GLHGLYGGG---NAGIVVEDIG------------  267
113  K-TT----SIYAVRLN-EVD------GLHGLYGGG---NAGIVVEDIG------------  265
114  K-TT----SIYAVRLN-EVD------GLHGLYGGG---NAGIVVEDIG------------  265
115  N-TC----SVYALRLN-EVD------GVHGLFGGE---SAGIRIEDIG------------  266
116  SKSC----YIYAARFN-EVD------GVHGLFGGP---NAGMVVEDIG------------  262
117  K-TC----SIYAIRAN-EVD------GLHGIYGGG---PAGIVVENIG------------  265
118  K-TC----SIYAIRAN-EVD------GLHGIYGGG---PAGIVVENIG------------  265
119  SKSC----YIYAARFN-EVD------GVHGLFGGP---NAGMVVEDIG------------  262
120  S-TC----SVYAVRAN-ELD------GLHGLYGGA---SAGIRVETIG------------  266
121  K-TT----SIYAVRLN-EVD------GLHGLYGGG---NAGIVVEDIG------------  265
122  K-TT----SIYAGRLN-EVD------GLHGLYGGG---NAGIVVEDIG------------  265
123  A-TC----SVYALRLN-EAD------GLHGLYGGE---SAGIVVENLG------------  265
124  N-TT----SIYAVRLN-EVD------GLHGLYGGG---NAGIVVEDIG------------  265
125  N-TT----SIYAVRLN-EVD------GLHGLYGGG---NAGIVVEDIG------------  265
126  K-TT----SIYAVRLN-EVD------GLHGLYGGG---NAGIVVEDIG------------  265
127  A-ST----SIYALRLN-EVD------GVHGLYGGA---TAGLRVEEIG------------  265
128  A-ST----SIYALRLN-EVD------GIHGLYGGA---TAGLRVEEIG------------  265
129  K-TC----SIYAVRLN-ELD------GMHGLWGGK---DAGIRVEDVG------------  265
130  K-TC----SIYAVRLN-ELD------GMHGLWGGK---DAGIRVEDVG------------  265
131  K-TC----SIYAVRLN-ELD------GMHGLWGGK---DAGIRVEDVG------------  265
132  K-TT----SIYAVRLN-EVD------GLHGLYGGG---NAGIVVEDIG------------  265
133  N-CT----TIFAGCID-DGSR---KVGLTGLTARN---AYGLEVEQVG------------  271
134  K-TC----SVYAIRLN-ELD------GMHGLWGGK---DAGIRVEDVG------------  265
135  K-TT----SIYAVRLN-EVD------GLHGLYGGG---NAGIVVEDIG------------  265
136  SEVC----PIYAMRLN-EVD------GLHGLYGGN---NAGIVVENIG------------  261
137  K-SC----SIYAVRFN-EAD------GMHGIFGGP---TAGFRVQSIG------------  265
138  A-TT----SVYALRLN-ELD------GLHGIYGGS---DAGIVVENIG------------  268
139  V-TT----SIYALRLN-EVD------GFHGIVGGE---AAGLKVEEIG------------  265
140  K-KA----PIYALHLS-EEN------GVTGLYGGD---NAGIVVESIG------------  262
141  A-TT----SVYALRLN-EDD------GLHGLHGGE---AAGIRLEALG------------  267
142  E-TC----SIYALRLN-EAD------GLHGIYGGH---SAGVRMEEIG------------  265
143  K-KA----PIYALHLS-EEN------GVTGLYGGD---NAGIVVESIG------------  262
144  A-TC----SIYALRLN-EAD------GFHGIYGGA---NAGVQLEDIG------------  265
145  D-LT----SIYALRLN-ETD------GFHMLYGGE---SAGIVVENVG------------  265
146  K-TA----SIYAVRFN-EAD------GMHGIFGGP---QAGLRVSHVG------------  265
147  D-SA----NIYCLHLS-EEN------GVTGLYGGE---NAGIVVENIG------------  259
148  D-SA----NIYCLHLS-EEN------GVTGLYGGE---NAGIVVENIG------------  259
149  D-SA----NIYCLHLS-EEN------GVTGLYGGE---NAGIVVENIG------------  259
150  T-TA----NIYCLHLS-EEN------GVTGLYGGE---NAGIVVENIG------------  259
151  G-TA----NIYCLHLS-EEN------GLTGLYGGD---NAGIVVENIG------------  272
152  T-TA----NIYCLHLS-EEN------GVTGLYGGE---NAGIVVENIG------------  259
153  A-TC----SIYALRLN-EAD------GFHGIYGGA---NAGVQLEDIG------------  265
154  D-AA----NIYCLHLS-EEN------GVTGLYGGE---NAGIVVENIG------------  259
155  G-TA----NIYCLHLS-EEN------GLTGLYGGD---NAGIVVENIG------------  272
156  T-TA----NIYCLHLS-EEN------GVTGLYGGE---NVGIVVENIG------------  259
157  T-SA----NIYCLHLS-EEN------GVTGLYGGE---NAGIVVENIG------------  259
158  G-TA----NIYCLHLS-EEN------GLTGLYGGD---NAGIVVENIG------------  272
159  G-TA----NIYCLHLS-EEN------GLTGLYGGD---NAGIVVENIG------------  272
160  G-TA----NIYCLHLS-EEN------GLTGLYGGD---NAGIVVENIG------------  272
161  K-TC----SIYAIRAN-EVD------GLHGIYGGG---PAGIVVENIG------------  265
162  V-TT----SVYALRLN-EYD------GLHGLYGGG---TAGVVVEDIG------------  272
163  G-TA----NIYCLHLS-EEN------GLTGLYGGD---NAGIVVENIG------------  272
164  T-AA----NIYCLHLS-EEN------GVTGLYGGE---NAGIVVENIG------------  259
165  T-GA----NIYCLHLS-EEN------GVTGLYGGE---NAGIVVENIG------------  259
166  T-AA----NIYCLHLS-EEN------GVTGLYGGE---NAGIVVENIG------------  259
167  D-SA----NIYCLHLS-EEN------GVTGLYGGE---NAGIVVENIG------------  259
168  T-GA----NIYCLHLS-EEN------GVTGLYGGE---NAGIVVENIG------------  259
169  D-SA----NIYCLHLS-EEN------GVTGLYGGE---NAGIVVENIG------------  259
170  G-KA----SIYCLHLS-EEN------GVTGLYGGD---NAGIVVENIG------------  257
171  T-GA----NIYCLHLS-EEN------GVTGLYGGE---NAGIVVENIG------------  259
172  G-TA----NIYCLHLS-EEN------GLTGLYGGD---NAGIVVENIG------------  272
173  S-KA----SIYCLHLS-EEN------GVTGLYGGD---NAGIVVENIG------------  257
174  G-KA----SIYCLHLS-EEN------GVTGLYGGD---NAGIVVENIG------------  257
175  G-KA----SIYCLHLS-EEN------GVTGLYGGD---NAGIVVENIG------------  257
176  D-SA----NIYCLHLS-EEN------GVTGLYGGE---NAGIVVENIG------------  259
```

```
177  G-TA----NIYCLHLS-EEN------GLTGLYGGD---NAGIVVENIG------------  272
178  G-TA----NIYCLHLS-EEN------GLTGLYGGD---NAGIVVENIG------------  272
179  G-KA----SIYCLHLS-EEN------GVTGLYGGD---NAGIVVENIG------------  257
180  T-GA----NIYCLHLS-EEN------GVTGLYGGE---NAGIVVENIG------------  259
181  G-KA----SIYCLHLS-EEN------GVTGLYGGD---NAGIVVENIG------------  257
182  G-KA----SIYCLHLS-EEN------GVTGLYGGD---NAGIVVESIG------------  257
183  G-KA----SIYCLHLS-EEN------GVTGLYGGD---NAGIVVENIG------------  257
184  G-KA----SIYCLHLS-EEN------GVTGLYGGD---NAGIVVENIG------------  257
185  G-TA----NIYCLHLS-EEN------GLTGLYGGD---NAGIVVENIG------------  272
186  G-KA----SIYCLHLS-EEN------GVTGLYGGD---NAGIVVENIG------------  257
187  G-KA----SIYCLHLS-EEN------GVTGLYGGD---NAGIVVENIG------------  257
188  G-KA----SIYCLHLS-EEN------GVTGLYGGD---NAGIVVENIG------------  257
189  G-KA----SIYCLHLS-EEN------GVTGLYGGD---NAGIVVENIG------------  257
190  D-CT----SIWFIQFG-MMR------GVIGLE------NGGIVHEDVG------------  261
191  G-TA----NIYCLHLS-EEN------GLTGLYGGD---NAGIVVENIG------------  272
192  K-TT----SIYALRLN-EAD------GFHGLWAGA---QAGIRYEHLG------------  264
193  G-KA----SIYCLHLS-EEN------GVTGLYGGD---NAGIVVENIG------------  257
194  G-KA----SIYCLHLS-EEN------GVTGLYGGD---NAGIVVENIG------------  257
195  G-KA----SIYCLHLS-EEN------GVTGLYGGD---NAGIVVENIG------------  257
196  G-KA----SIYCLHLS-EEN------GVTGLYGGD---NAGIVVENIG------------  257
197  G-KA----SIYCLHLS-EEN------GVTGLYGGD---NAGIVVENIG------------  257
198  G-KA----SIYCLHLS-EEN------GVTGLYGGD---NAGIVVENIG------------  257
199  G-KA----SIYCLHLS-EEN------GVTGLYGGD---NAGIVVENIG------------  257
200  G-KA----SIYCLHMS-EEN------GVTGLYGGD---NAGIVVENIG------------  257
201  G-KA----SIYCLHLS-EEN------GVTGLYGGD---NAGIVVENIG------------  257
202  E-TC----SIYALRLN-ETD------GFHGIYGGP---TAGVQFEDIG------------  264
203  G-KA----SIYCLHLS-EEN------GVTGLYGGD---NAGIVVENIG------------  257
204  GNTC----SVYAARLN-EVD------GLHGLYGGD---NAGLVVENIG------------  267
205  G-KA----SIYCLHLS-EEN------GVTGLYGGD---NAGIVVENIG------------  257
206  G-KA----SIYCLHLS-EEN------GVTGLYGGD---NAGIVVENIG------------  257
207  G-KA----SIYCLHLS-EEN------GVTGLYGGD---NAGIVVENIG------------  257
208  G-KA----SIYCLHLS-EEN------GVTGLYGGD---NAGIVVENIG------------  257
209  G-KA----SIYCLHLS-EEN------GVTGLYGGD---NAGIVVENIG------------  257
210  G-KA----SIYCLHLS-EEN------GVTGLYGGD---NAGIVVENIG------------  257
211  G-KA----SIYCLHLS-EEN------GVTGLYGGD---NAGIVVENIG------------  257
212  G-KA----SIYCLHLS-EEN------GVTGLYGGD---NAGIVVENIG------------  257
213  G-KA----SIYCLHLS-EEN------GVTGLYGGD---NAGIVVENIG------------  257
214  G-TA----NIYCLHLS-EEN------GLTGLYGGD---NAGIVVENIG------------  272
215  G-KA----SIYCLHLS-EEN------GVTGLYGGD---NAGIVVENIG------------  257
216  G-KA----SIYCLHLS-EEN------GVTGLYGGD---NAGIVVENIG------------  257
217  G-KA----SIYCLHLS-EES------GVTGLYGGD---NAGIVVENIG------------  257
218  G-KA----SIYCLHLS-EEN------GVTGLYGGD---NAGIVVENIG------------  257
219  G-KA----SIYCLHLS-EEN------GVTGLYGGD---NAGIVVENIG------------  257
220  G-KA----SIYCLHLS-EEN------GVTGLYGGD---NAGIVVENIG------------  257
221  G-KA----SIYCLHLS-EEN------GVTGLYGGD---NAGIVVENIG------------  257
222  G-KA----SIYCLHLS-EEN------GVTGLYGGD---NAGIVVENIG------------  257
223  G-KA----SIYCLHLS-EEN------GVTGLYGGD---NAGIVVENIG------------  257
224  G-KA----SIYCLHLS-EEN------GVTGLYGGD---NAGIVVENIG------------  257
225  G-KA----SIYCLHLS-EEN------GVTGLYGGE---NAGIVVENIG------------  257
226  G-KA----SIYCLHLS-EEN------GVTGLYGGD---NAGIVVENIG------------  257
227  G-KA----SIYCLHLS-EEN------GVTGLYGGD---NAGIVVENIG------------  257
228  --TC----PIYAIRAN-ELD------GLHGLFGGD---NAGIVVENLG------------  258
229  G-KA----SIYCLHLS-EEN------GVTGLYGGD---NAGIVVENIG------------  257
230  --TC----QIYAIRAN-ELD------GLHGLFGGD---NAGIVVENLG------------  258
231  G-KA----SIYCLHLS-EEN------GVTGLYGGD---NAGIVVENIG------------  257
232  G-TA----NIYCLHLS-EEN------GLTGLYGGD---NAGIVVENIG------------  272
233  G-KA----SIYCLHLS-EEN------GVTGLYGGD---NAGIVVENIG------------  257
234  G-KA----SIYCLHLS-EEN------GVTGLYGGD---NAGIVVENIG------------  257
235  G-KA----SIYCLHLS-EEN------GVTGLYGGD---NAGIVVENIG------------  257
236  G-KA----SIYCLHLS-EEN------GVTGLYGGD---NAGIVVENIG------------  257
237  G-KA----SIYCLHLS-EEN------GVTGLYGGD---NAGIVVENIG------------  257
238  G-KA----SIYCLHMS-EEN------GVTGLYGGD---NAGIVVENIG------------  257
239  N-LS----SIYAVKFG--PS------GLMGLE------NGGITVEEVG------------  260
240  G-TA----NIYCLHLS-EEN------GLTGLYGGD---NAGIVVENIG------------  272
241  G-KA----SIYCLHLS-EEN------GVTGLYGGE---NAGIVVENIG------------  257
242  G-KA----SIYCLHLS-EEN------GVTGLYGGD---NAGIVVENIG------------  257
243  G-KA----SIYCLHLS-EEN------GVTGLYGGD---NAGIVVENIG------------  257
```

244	G-KA----SIYCLHLS-EEN------GVTGLYGGD---NAGIVVENIG------------	257
245	G-KA----SIYCLHLS-EEN------GVTGLYGGD---NAGIVVENIG------------	257
246	G-KA----SIYCLHLS-EEN------GVTGLYGGD---NAGIVVENIG------------	257
247	G-KA----SIYCLHLS-EEN------GVTGLYGGD---NAGIVVENIG------------	257
248	G-KA----SIYCLHLS-EEN------GVTGLYGGD---NAGIVVENIG------------	257
249	G-KA----SIYCLHLS-EEN------GVTGLYGGD---NAGIVVENIG------------	257
250	G-IA----SIYCLHLS-EEN------GVTGLYGGD---NAGIVVENIG------------	257
251	G-KA----SIYCLHLS-EEN------GVTGLYGGD---NAGIVVENIG------------	257
252	G-KA----SIYCLHLS-EEN------GVTGLYGGD---NAGIVVENIG------------	257
253	G-KA----SIYCLHLS-EEN------GVTGLYGGD---NAGIVVENIG------------	257
254	G-KA----SIYCLHLS-EEN------GVTGLYGGD---NAGIVVENIG------------	257
255	G-KA----SIYCLHLS-EEN------GVTGLYGGD---NAGIVVENIG------------	257
256	G-KA----SIYCLHLS-EEN------GVTGLYGGE---NAGIVVENIG------------	257
257	G-KA----SIYCLHLS-EEN------GVTGLYGGE---NAGIVVENIG------------	257
258	G-KA----SIYCLHLS-EEN------GVTGLYGGD---NAGIVVENIG------------	257
259	G-KA----SIYCLHLS-EEN------GVTGLYGGD---NAGIVVENIG------------	257
260	G-KA----SIYCLHLS-EEN------GVTGLYGGD---NAGIVVENIG------------	257
261	T-KA----DIYCLHLS-EEN------GVTGLYGGE---NAGIVVENIG------------	258
262	G-KA----SIYCLHLS-EEN------GVTGLYGGD---NAGIVVENIG------------	257
263	D-AA----DIYCLHLS-EEN------GVTGLYGGE---NAGIVVENIG------------	259
264	T-AA----DIYCLHLS-EEN------GVTGLYGGE---NAGIVVENIG------------	259
265	T-KA----DIYCLHLS-EEN------GVTGLYGGE---NAGIVVENIG------------	258
266	G-KA----SIYCLHLS-EEN------GVTGLYGGD---NAGIVVENIG------------	257
267	T-KA----DIYCLHLS-EEN------GVTGLYGGK---NAGIVVENIG------------	258
268	T-KA----DIYCLHLS-EEN------GVTGLYGGE---NAGIVVENIG------------	258
269	G-KA----SIYCLHLS-EEN------GVTGLYGGE---NAGIVVENIG------------	257
270	--TC----QIYALRAN-ELD------GLHGLYGGE---NAGIVVENIG------------	258
271	G-KA----SIYCLHLS-EEN------GVTGLYGGD---NAGIVVENIG------------	257
272	G-KA----SIYCLHLS-EEN------GVTGLYGGD---NAGIVVENIG------------	257
273	T-NA----DIYCLHLS-EEN------GVTGLYGGE---NAGIVVENIG------------	258
274	T-KA----DIYCLHLS-EEN------GVTGLYGGE---NAGIVVENIG------------	258
275	G-KA----SIYCLHLS-EEN------GVTGLYGGD---NAGIVVENIG------------	257
276	G-KA----SIYCLHLS-EEN------GVTGLYGGD---NAGIVVENIG------------	257
277	G-KA----SIYCLHLS-EEN------GVTGLYGGD---NAGIVVENIG------------	257
278	T-KA----DIYCLHLS-EEN------GVTGLYGGE---NAGIVVENIG------------	258
279	G-KA----SIYCLHLS-EEN------GVTGLYGGD---NAGIVVENIG------------	257
280	G-KA----SIYCLHLS-EEN------GVTGLYGGD---NAGIVVENIG------------	257
281	K-TT----SIYAVRLN-EVD------GLHGLYGGG---NAGIVVEDIG------------	265
282	T-NA----DIYCLHLS-EEN------GVTGLYGGE---NAGIVVENIG------------	258
283	G-KA----SIYCLHLS-EEN------GVTGLYGGD---NAGIVVENIG------------	257
284	G-KA----SIYCLHLS-EEN------GVTGLYGGD---NAGIVVENIG------------	257
285	G-KA----SIYCLHLS-EEN------GVTGLYGGD---NAGIVVENIG------------	257
286	G-KA----SIYCLHLS-EEN------GVTGLYGGE---NAGIVVENIG------------	257
287	T-KA----DIYCLHLS-EEN------GVTGLYGGD---NAGIVVENIG------------	258
288	G-KA----NIYCLHLS-EEN------GVTGLYGGE---NAGIVVENIG------------	257
289	T-KA----DIYCLHLS-EEN------GVTGLYGGE---NAGIVVENIG------------	258
290	G-KA----SIYCLHLS-EEN------GVTGLYGGD---NAGIVVENIG------------	257
291	G-KA----SIYCLHLS-EEN------GVTGLYGGD---NAGIVVENIG------------	257
292	G-KA----SIYCLHLS-EEN------GVTGLYGGD---NAGIVVENIG------------	257
293	G-KA----SIYCLHMS-EEN------GVTGLYGGD---NAGIVVENIG------------	257
294	G-KA----SIYCLHLS-EEN------GVTGLYGGD---NAGIVVENIG------------	257
295	T-KA----DIYCLHLS-EEN------GVTGLYGGE---NAGIVVENIG------------	258
296	T-KA----DIYCLHLS-EEN------GVTGLYGGE---NAGIVVENIG------------	258
297	T-KA----DIYCLHLS-EEN------GVTGLYGGE---NAGIVVENIG------------	258
298	G-KA----SIYCLHLS-EEN------GVTGLYGGD---NAGIVVENIG------------	257
299	T-KA----DIYCLHLS-EEN------GVTGLYGGE---NAGIVVENIG------------	258
300	G-KA----SIYCLHLS-EEN------GVTGLYGGD---NAGIVVENIG------------	251
301	T-KA----DIYCLHLS-EEN------GVTGLYGGE---NAGIVVENIG------------	258
302	T-KA----DIYCLHLS-EEN------GVTGLYGGE---NAGIVVENIG------------	258
303	G-KA----SIYCLHLS-EEN------GVTGLYGGE---NAGIVVENIG------------	257
304	T-KA----DIYCLHLS-EEN------GVTGLYGGD---NAGIVVENIG------------	258
305	T-KA----DIYCLHLS-EEN------GVTGLYGGE---NAGIVVENIG------------	258
306	T-KA----DIYCLHLS-EEN------GVTGLYGGE---NAGIVVENIG------------	258
307	G-KA----SIYCLHLS-EEN------GVTGLYGGE---NAGIVVENIG------------	257
308	T-KA----DIYCLHLS-EEN------GVTGLYGGE---NAGIVVENIG------------	258
309	G-KA----SIYCLHLS-EEN------GVTGMYGGE---NAGIVVENIG------------	257
310	G-KA----SIYCLHLS-EEN------GVTGLYGGE---NAGIVVENIG------------	257

```
311 G-KA----SIYCLHLS-EEN------GVTGLYGGE---NAGIVVENIG------------ 257
312 T-KA----DIYCLHLS-EEN------GVTGLYGGD---NAGIVVENIG------------ 258
313 G-KA----SIYCLHLS-EEN------GVTGLYGGD---NAGIVVENIG------------ 257
314 G-KA----SIYCLHLS-EEN------GVTGLYGGE---NAGIVVENIG------------ 257
315 G-KA----SIYCLHLS-EEN------GVTGLYGGD---NAGIVVENIG------------ 257
316 T-KA----DIYCLHLS-EEN------GVTGLYGGE---NAGIVVENIG------------ 258
317 T-AA----DIYCLHLS-EEN------GVTGLYGGE---NAGIVVENIG------------ 259
318 T-KA----DIYCLHLS-EEN------GVTGLYGGE---NAGIVVENIG------------ 258
319 T-KA----DIYCLHLS-EEN------GVTGLYGGE---NAGIVVENIG------------ 258
320 G-KA----SIYCLHLS-EEN------GVTGLYGGE---NAGIVVENIG------------ 257
321 T-KA----DIYCLHLS-EEN------GVTGLYGGE---NAGIVVENIG------------ 258
322 G-KA----SIYCLHLS-EEN------GVTGLYGGE---NAGIVVENIG------------ 257
323 G-KA----SIYCLHLS-EEN------GVTGLYGGE---NAGIVVENIG------------ 257
324 G-KA----SIYCLHLS-EEN------GVTGLYGGD---NAGIVVENIG------------ 257
325 G-KA----SIYCLHLS-EEN------GVTGLYGGD---NAGIVVENIG------------ 257
326 T-KA----DIYCLHLS-EEN------GVTGLYGGE---NAGIVVENIG------------ 258
327 T-KA----DIYCLHLS-EEN------GVTGLYGGE---NAGIVVENIG------------ 258
328 T-KA----DIYCLHLS-EEN------GVTGLYGGE---NAGIVVENIG------------ 258
329 G-KA----SIYCLHLS-EEN------GVTGLYGGE---NAGIVVENIG------------ 257
330 G-KA----SIYCLHLS-EEN------GVTGLYGGE---NAGIVVENIG------------ 257
331 T-KA----DIYCLHLS-EEN------GVTGLYGGE---NAGIVVENIG------------ 258
332 G-KA----SIYCLHLS-EEN------GVTGLYGGE---NAGIVVENIG------------ 257
333 G-KA----SIYCLHLS-EEN------GVTGLYGGE---NAGIVVENIG------------ 257
334 T-AA----DIYCLHLS-EEN------GVTGLYGGE---NAGIVVENIG------------ 259
335 G-KA----SIYCLHLS-EEN------GVTGLYGGE---NAGIVVENIG------------ 257
336 G-KA----SIYCLHLS-EEN------GVTGLYGGD---NAGIVVENIG------------ 257
337 G-KA----SIYCLHLS-EEN------GVTGLYGGE---NAGIVVENIG------------ 257
338 T-KA----DIYCLHLS-EEN------GVTGLYGGE---NAGIVVENIG------------ 258
339 G-KA----SIYCLHLS-EEN------GVTGLYGGE---NAGIVVENIG------------ 257
340 T-KA----DIYCLHLS-EEN------GVTGLYGGE---NAGIVVENIG------------ 258
341 G-KA----SIYCLHLS-EEN------GVTGLYGGE---NAGIVVENIG------------ 257
342 G-TA----NIYCLHLS-EEN------GLTGLYGGD---NAGIVVENIG------------ 272
343 G-KA----SIYCLHLS-EEN------GVTGLYGGE---NAGIVVENIG------------ 257
344 G-KA----SIYCLHMS-EEN------GVTGLYGGE---NAGIVVENIG------------ 257
345 T-KA----DIYCLHLS-EEN------GVTGLYGGE---NAGIVVENIG------------ 258
346 G-KA----SIYCLHLS-EEN------GVTGLYGGD---NAGIVVENIG------------ 257
347 G-KA----SIYCLHLS-EEN------GVTGLYGGD---NAGIVVENIG------------ 257
348 S-AA----DIYGLHLS-EEN------GVTGLYGGE---NAGIVVENIG------------ 259
349 G-KA----SIYCLHLS-EEN------GVTGLYGGE---NAGIVVENIG------------ 257
350 G-KA----SIYCLHLS-EEN------GVTGLYGGE---NAGIVVENIG------------ 257
351 K-TT----SIYAVRLN-EVD------GLHGLYGGG---NAGIVVEDIG------------ 246
352 G-KA----SIYCLHLS-EEN------GVTGLYGGE---NAGIVVENIG------------ 257
353 T-CT----SVVAGTLD-DGSM---QSGIAGLTAMD---ASGIQVEEVG------------ 272
354 T-KA----DIYCLHLS-EEN------GVTGLYGGE---NAGIVVENIG------------ 255
355 T-AA----DIYCLHLS-EEN------GVTGLYGGE---NAGIVVENIG------------ 259
356 G-KA----SIYCLHLS-EEN------GVTGLYGGE---NAGIVVENIG------------ 257
357 T-KA----DIYCLHLS-EEN------GVTGLYGGE---NAGIVVENIG------------ 258
358 G-KA----SIYCLHLS-EEN------GVTGLYGGE---NAGIVVENIG------------ 257
359 T-KA----DIYCLHLS-EEN------GVTGLYGGE---NAGIVVENIG------------ 258
360 G-KA----SIYCLHLS-EEN------GVTGLYGGE---NAGIVVENIG------------ 257
361 D-AA----DIYCLHLS-EEN------GVTGLYGGD---NAGIVVENIG------------ 259
362 T-KA----DIYCLHLS-EEN------GVTGLYGGE---NAGIVVENIG------------ 258
363 G-KA----SIYCLHLS-EEN------GVTGLYGGE---NAGIVVENIG------------ 257
364 G-KA----SIYCLHLS-EEN------GVTGLYGGE---NAGIVVENIG------------ 257
365 G-KA----SIYCLHLS-EEN------GVTGLYGGE---NAGIVVENIG------------ 257
366 G-KA----SIYCLHLS-EEN------GVTGLYGGE---NAGIVVENIG------------ 257
367 A-AA----DIYGLHLS-EEN------GVTGLYGGE---NAGIVVENIG------------ 259
368 A-AA----DIYGLHLS-EEN------GVTGLYGGE---NAGIVVENIG------------ 259
369 G-KA----SIYCLHLS-EEN------GVTGLYGGE---NAGIVVENIG------------ 257
370 G-KA----SIYCLHLS-EEN------GVTGLYGGE---NAGIVVENIG------------ 257
371 G-KA----SIYCLHLS-EEN------GVTGLYGGE---NAGIVVENIG------------ 257
372 G-KA----SIYCLHLS-EEN------GVTGLYGGE---NAGIVVENIG------------ 257
373 G-KA----SIYCLHLS-EEN------GVTGLYGGE---NAGIVVENIG------------ 257
374 G-KA----SIYCLHLS-EEN------GVTGLYGGD---NAGIVVENIG------------ 257
375 G-KA----SIYCLHLS-EEN------GVTGLYGGE---NAGIVVENIG------------ 257
376 G-KA----SIYCLHLS-EEN------GVTGLYGGD---NAGIVVENIG------------ 257
377 G-LS----SIYAVKWG--PS------GLMGLE------NGGIVVEEVG------------ 260
```

```
378  G-KA----SIYCLHLS-EEN------GVTGLYGGE---NAGIVVENIG------------  257
379  G-KA----SIYCLHLS-EEN------GVTGLYGGE---NAGIVVENIG------------  257
380  S-AA----DIYGLHLS-EEN------GVTGLYGGE---NAGIVVENIG------------  259
381  S-AA----DIYGLHLS-EEN------GVTGLYGGE---NAGIVVENIG------------  259
382  A-CT----TIFAGVFD-DGDM---KTGLLGLTAAN---AYGMHVVDVG------------  271
383  A-AA----DIYGLHLS-EEN------GVTGLYGGE---NAGIVVENIG------------  259
384  A-AA----DIYGLHLS-EEN------GVTGLYGGE---NAGIVVENIG------------  259
385  G-KA----SIYCLHLS-EEN------GVTGLYGGE---NAGIVVENIG------------  257
386  G-KA----SIYCLHLS-EEN------GVTGLYGGD---NAGIVVENIG------------  257
387  L-TGGSFRSVYAMRLN-EAD------GLFGVYGGS--EAAGFTVEDIG------------  272
388  S-AA----DIYGLHLS-EEN------GVTGLYGGE---NAGIVVENIG------------  259
389  T-KA----DIYCLHLS-EEN------GVTGLYGGE---NAGIVVENIG------------  258
390  N-CT----TILAGVLD-DGSR---KVGVTGLTAKN---AYGLAVEPVG------------  271
391  A-AA----DIYGLHLS-EEN------GVTGLYGGE---NAGIVVENIG------------  259
392  G-KA----SIYCLHLS-EEN------GVTGLYGGE---NAGIVVENIG------------  257
393  G-KA----SIYCLHLS-EEN------GVTGLYGGE---NAGIVVENIG------------  257
394  A-AA----DIYGLHLS-EEN------GVTGLYGGE---NAGIVVENIG------------  259
395  A-AA----DIYGLHLS-EEN------GVTGLYGGE---NAGIVVENIG------------  259
396  G-TA----NIYCLHLS-EEN------GLTGLYGGE---NAGIVVENIG------------  271
397  N-AT----SVFAGTFD-DGSR---THGIAGLTAER---QAGMHIKYVG------------  275
398  G-KA----SIYCLHLS-EEN------GVTGLYGGD---NAGIVVENIG------------  257
399  S-TA----DIFCLHLS-EEN------GVTGLYGGE---NAGIVVENIG------------  259
400  T-AA----DIYGLHLS-EEN------GVTGLYGGE---NAGIVVENIG------------  259
401  S-AA----DIYGLHLS-EEN------GVTGLYGGE---NAGIVVENIG------------  259
402  S-AA----DIYGLHLS-EEN------GVTGLYGGE---NAGIVVENIG------------  259
403  T-KA----DIYCLHLS-EEN------GVTGLYGGE---NAGIVVENIG------------  258
404  T-KA----DIYCLHLS-EEN------GVTGLYGGE---NAGIVVENIG------------  258
405  G-KA----SIYCLHLS-EEN------GVTGLYGGD---NAGIVVENIG------------  257
406  A-AA----DIYGLHLS-EEN------GVTGLYGGE---NAGIVVENIG------------  259
407  T-KA----DIYCLHLS-EEN------GVTGLYGGE---NAGIVVENIG------------  258
408  G-KA----SIYCLHLS-EEN------GVTGLYGGE---NAGIVVENIG------------  257
409  A-AA----DIYGLHLS-EEN------GVTGLYGGE---NAGIVVENIG------------  259
410  A-AA----DIYGLHLS-EEN------GVTGLYGGE---NAGIVVENIG------------  259
411  A-AA----DIYGLHLS-EEN------GVTGLYGGE---NAGIVVENIG------------  259
412  A-AA----DIYGLHLS-EEN------GVTGLYGGE---NAGIVVENIG------------  259
413  G-KA----SIYCLHLS-EEN------GVTGLYGGD---NAGIVVENIG------------  257
414  A-AA----DIYGLHLS-EEN------GVTGLYGGE---NAGIVVENIG------------  259
415  A-AA----DIYGLHLS-EEN------GVTGLYGGE---NAGIVVENIG------------  259
416  A-AA----DIYGLHLS-EEN------GVTGLYGGE---NAGIVVENIG------------  259
417  G-TA----NIYCLHLS-EEN------GLTGLYGGE---NAGIVVENIG------------  271
418  G-KA----SIYCLHLS-EEN------GVTGLYGGE---NAGIVVENIG------------  257
419  A-TT----SVYALRLN-EID------GFHGLYGGN---AAGIRIDDLG------------  265
420  A-AA----DIYGLHLS-EEN------GVTGLYGGE---NAGIVVENIG------------  259
421  G-KA----SIYCLHLS-EEN------GVTGLYGGD---NAGIVVENIG------------  257
422  G-TA----NIYCLHLS-EEN------GLTGLYGGD---NAGIVVENIG------------  272
423  G-TA----NIYCLHLS-EEN------GLTGLYGGE---NAGIVVENIG------------  271
424  A-AA----DIYGLHLS-EEN------GVTGLYGGE---NAGIVVENIG------------  259
425  G-TA----NIYCLHLS-EEN------GLTGLYGGD---NAGIVVENIG------------  272
426  G-TA----NIYCLHLS-EEN------GLTGLYGGD---NAGIVVENIG------------  272
427  G-TA----NIYCLHLS-EEN------GLTGLYGGD---NAGIVVENIG------------  272
428  S-AA----DIYGLHLS-EEN------GVTGLYGGE---NAGIVVENIG------------  259
429  G-KA----SIYCLHLS-EEN------GVTGLYGGD---NAGIVVENIG------------  257
430  G-TA----NIYCLHLS-EEN------GLTGLYGGD---NAGIVVENIG------------  272
431  A-AA----DIYGLHLS-EEN------GVTGLYGGE---NAGIVVENIG------------  259
432  A-QT----TIFAGTLD-DGSR---THGIAGLTAAN---DAGIVIEDVG------------  271
433  G-KA----SIYCLHLS-EEN------GVTGLYGGD---NAGIVVENIG------------  257
434  T-KA----DIYCLHLS-EEN------GVTGLYGGE---NAGIVVENIG------------  258
435  T-KA----DIYCLHLS-EEN------GVTGLYGGE---NAGIVVENIG------------  258
436  A-AA----DIYGLHLS-EEN------GVTGLYGGE---NAGIVVENIG------------  259
437  G-KA----SIYGLHMS-EEN------GLTGLYGGD---NAGIVVENIG------------  257
438  A-AA----DIYGLHLS-EEN------GVTGLYGGE---NAGIVVENIG------------  259
439  --TC----QIFALRAN-ELD------GLHGLYGGE---NAGIVVENIG------------  258
440  G-KA----SIYCLHLS-EEN------GVTGLYGGD---NAGIVVENIG------------  257
441  G-LS----SIYAVKWG--PS------GLMGLE------NGGIVVEEVG------------  260
442  Q-KA----DIYCLHMS-EEN------GLTGLYGGD---TAGIVVEDIG------------  260
443  G-KA----SIYCLHLS-EEN------GVTGLYGGE---NAGIVVENIG------------  257
444  T-CT----SIIAGCLD-DGSR---KVGIAGLHAKK---AMGIQVKALG------------  271
```

```
445   A-QT----TVFAGVFD-DGDR---KTGLAGLTATN---AFGLHVERVG------------   271
446   G-KA----SIYCLHLS-EEN------GVTGLYGGD---NAGIVVENIG------------   257
447   G-KA----SIYCLHLS-EEN------GVTGLYGGD---NAGIVVENIG------------   257
448   N-LS----SIYAVKFG--SA------GVMGLE------NGGIQVVRVG------------   258
449   G-KA----SIYCLHLS-EEN------GVTGLYGGD---NAGIVVENIG------------   257
450   G-KA----SIYCLHLS-EEN------GVTGLYGGD---NAGIVVENIG------------   257
451   N-LS----SIYAVKFG--PS------GLMGLE------NGGITVEEVG------------   260
452   N-LS----SIYAVKFG--PS------GVMGIE------NGGIQIEAIG------------   260
453   A-AA----DIYGLHLS-EEN------GVTGLYGGE---NAGIVVENIG------------   259
454   N-AT----SVFAGTFD-DGSR---THGIAGLTAER---QAGMHIKYVG------------   275
455   S-TC----SIYAVRLN-ELD------GFHGLWGGK---DAGIRVEDVG------------   265
456   T-KA----DIYCLHLS-EEN------GVTGLYGGE---NAGIVVENIG------------   258
457   G-KA----SIYCLHLS-EEN------GVTGLYGGD---NAGIVVENIG------------   257
458   A-AA----DIYGLHLS-EEN------GVTGLYGGE---NAGIVVENIG------------   259
459   N-LA----SVYAVRAN-ELD------GFHGLYGGD---AAGVRVEAIG------------   265
460   G-KA----SIYCLHLS-EEN------GVTGLYGGD---NAGIVVENIG------------   257
461   N-AT----SVFAGTFD-DGSR---THGIAGLTAER---QAGMHVKYVG------------   275
462   G-KA----SIYCLHLS-EEN------GVTGLYGGD---NAGIVVENIG------------   257
463   A-NR----RIWFMVLG-DDGGKGPTRGLSRIVPAG--MERRPFVVRMTNGVPDF------   191
464   G-TA----NIYCLHLS-EEN------GLTGLYGGD---NAGIVVENIG------------   272
465   G-KA----SIYCLHLS-EEN------GVTGLYGGD---NAGIVVENIG------------   257
466   G-KA----SIYCLHLS-EEN------GVTGLYGGD---NAGIVVENIG------------   257
467   G-KA----SIYCLHLS-EEN------GVTGLYGGD---NAGIVVENIG------------   257
468   G-KA----SIYCLHLS-EEN------GVTGLYGGD---NAGIVVENIG------------   257
469   K-SA----SIYCLHMS-EEN------GLTGLYGGD---NAGIVVEDIG------------   260
470   G-KA----SIYCLHLS-EEN------GVTGLYGGD---NAGIVVENIG------------   257
471   N-CT----TVFAGTFD-DGSR---THGIAGLTAEE---AAGIQIEDVG------------   272
472   A-TA----SVYALRLN-EQD------GLHGLYGGD---NAGIVVENIG------------   275
473   N-CT----TVFAGTFD-DGSR---THGIAGLTAEE---AAGIQIEDVG------------   272
474   G-KA----SIYCLHLS-EEN------GVTGLYGGD---NAGIVVENIG------------   257
475   G-KA----SIYCLHLS-EEN------GVTGLYGGD---NAGIVVENIG------------   257
476   N-AT----SVFAGTFD-DGSR---THGIAGLTAER---QAGMHVKYVG------------   275
477   N-TT----TLFAGTLD-DGSG---KHGIAGLTAAG---EAGIRVTEIG------------   271
478   N-CT----SIIAGCLD-DGSQ---RVGIAGLHAKK---AMGIQVKDIG------------   271
479   N-AT----SVFAGTFD-DGSR---THGIAGLTAAT---EAGVKIKYVG------------   275
480   N-TT----SIFAGTLD-DGSR---THGLAGLTAEE---AAGIRVEDVG------------   272
481   G-KA----SIYCLHLS-EEN------GVTGLYGGD---NAGIVVENIG------------   257
482   G-KA----SIYCLHLS-EEN------GVTGLYGGD---NAGIVVENIG------------   257
483   K-KS----DIFCLHMS-EEN------GLTGLYGGE---NAGIVVEDIG------------   260
484   G-KA----SIYCLHLS-EEN------GVTGLYGGD---NAGIVVENIG------------   257
485   N-LS----SIYAVKFG--PS------GVMGIE------NGGIQIEAIG------------   260
486   K-KS----DIYCLHMS-EEN------GLTGLYGGD---TAGIVVEDIG------------   260
487   A-AA----DIYGLHLS-EEN------GVTGLYGGE---NAGIVVENIG------------   259
488   Q-KA----DIYCLHLS-EEN------GLTGLYGGD---NAGIVVEDIG------------   260
489   K-KA----DIYCLHLS-EEN------GLTGLYGGD---NAGIVVESIG------------   260
490   N-AT----SVFAGTFD-DGSR---THGIAGLTAEK---QAGMHVKYVG------------   275
491   G-KA----SIYCLHLS-EEN------GVTGLYGGD---NAGIVVENIG------------   257
492   T-ST----SIVAGCLD-DGSQ---RVGIAGLHAKK---AMGIQVKNIG------------   271
493   T-KA----DIYCLHLS-EEN------GVTGLYGGD---NAGIVVENIG------------   258
494   G-LS----SIYAVKFG--PA------GLMGLE------HGGIQVERVG------------   260
495   T-KA----DIYCLHLS-EEN------GVTGLYGGE---NAGIVVENIG------------   258
496   T-KA----DIYCLHLS-EEN------GVTGLYGGE---NAGIVVENIG------------   258
497   N-CT----TVFAGTFD-DGSG---MHGVSGITPTM---NAGMSVTEIG------------   272
498   G-KA----SIYCLHLS-EEN------GVTGLYGGD---NAGIVVENIG------------   257
499   Q-KA----DIYCLHLS-EEN------GLTGLYGGD---NAGIVVEDIG------------   260
500   N-AT----SVFAGTFD-DGSR---THGIAGLTAEK---QAGMHVKYVG------------   275
```

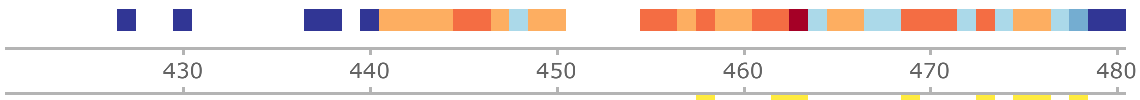


```
1     --------------------IRTEGSEIVV----HKTWPVGLALGSQAALSSLKNVAI--   288
2     --------------------IRTEGSEIVV----HKTWPVGLALGSQAALSSLKNVAI--   288
3     --------------------LRTEGSEIVV----HKTWPVGLALGSQAALASVKNVAI--   288
4     --------------------IRTEGSEIVV----HKTWPVGLALGSQAALASVKNVAI--   288
5     --------------------VRTEGSEIVV----HKTWPVGLALGSQAALASVKNVAI--   288
```

```
6   --------------------IRTEGSEIVV----HKTWPVGLALGSQAALASVKNVAI--  288
7   --------------------IRTEGSEIVV----HKTWPVGLALGSQAALASLKNVAI--  286
8   --------------------VRTEGSEIVV----HKTWPVGLALGSQAALASVKNVAI--  286
9   --------------------IRTEGSEIVV----HKTWPVGLALGSQAALASLKNVAI--  286
10  --------------------VRTEGSEIVV----HKTWPVGLALGSQAALASVKNVAI--  286
11  --------------------IRTEGSEIIL----HKTWPVGLALGSQAALASVNNVAI--  286
12  --------------------LRTEGSEIVV----HKTWPVGLALGSQAALASVKNVAI--  286
13  --------------------VRTEGSEIVV----HKTWPVGLALGSQAALASVKNVAIQ-  287
14  --------------------SQADQSQTVV----SVTWPVGLALGSQGALSGILNVGIN-  287
15  --------------------IQADASTLVY----HLTMPVGIALGSACALAVIKNATIPK  327
16  --------------------ILADASTLVY----HLSMPAGIALGSSAALSVIRNATIPV  327
17  --------------------ILADASTLVY----HLSMPAGIALGSSAALSVIRNATIPV  327
18  --------------------ILADASTLVY----HLAMPVGIALGSAAALSVIKNVTIPK  327
19  --------------------MLPDGSTLVY----HVTMPVSIALGSASSLAVIKNVAIPS  326
20  --------------------ILADASTLVY----HMMMPVGIALGSACALSVIKNATIPR  328
21  --------------------MLPDGSTLVY----HVTMPASIALGSASSLAVIKNAVIPA  326
22  --------------------MLPDGSTLVY----HVTMPASIALGSASSLAVIKNAVIPA  326
23  --------------------MLPDGSTLVY----HVTMPASIALGSASSLAVIKNAVIPA  327
24  --------------------MLPDGSTLVY----HVTMPASIALGSVSSLAVIKNAVIPA  326
25  --------------------MLPDGSTLVY----HVTMPASIALGSASSLAVIKNALIPA  326
26  --------------------MLPDGSTLVY----HVTMPASIALGSASSLAVIKNAVIPA  326
27  --------------------ILADASTLVY----HLAMPVGIALGSSAALSVIRHATIPG  327
28  --------------------MLPDGSTLVY----HVTMPASIALGSVSSLAVIKNAVIPA  326
29  SQQVLVPDVPTTITVNGATITPSLNAVTTN----WVSWPCGMAMGAQGSLSILKGFGL-V  342
30  SQQVLVPDVPTTITVNGATITPSLNAVTTN----WVSWPCGMAMGAQGSLSILKGFGL-V  348
31  ----EVPEAP------------YLKAITNT----WVSWPCGMAMGAQGSLSILKGFNL-V  323
32  ----EVPEAP------------YLKAITNT----WVSWPCGMAMGAQGSLSILKGFNL-V  323
33  ---VTVPEVPTTIQPTG---SVTVNAITDT----WVSMPCGLAVGAQGSLSILKGFNL-V  338
34  --------------------AASSTSQINV----DYTFPVATAMGSSGALAILEGVD--V  332
35  --------------------SAATTSRINV----TYTFPVATAMGTQGALAILENVL--V  332
36  --------------------VRTEGSEIVV----HKTWPVGLALGSQAALASVKNVAI--  209
37  --------------------SAHSTSLINV----DYTFPVAIAMGSSGALSILDQVD--V  328
38  ---APDPGKPF------TVQLPTLPTL-DT----WVSWPTGVAMGSQGSLSILSGFAL-V  332
39  --------------------TMPDGSETVG----QIFLPIGLALATESALAGITNIA---  300
40  --------------------STSTKSRICV----TYTFPVATAVGSSGALSVLEGVD--V  320
41  --------------------YDPDNGTIIV----FVTWPVSISLATKAALSAVVNIAITP  286
42  ---LDIPSVPFIGSVLAPVTIPTLPTV-DT----WVSWPTGVAMGSQGSLSILRGFPL-V  340
43  ---LDIPSVPFIGTAEGAVTVPTLPTV-DT----WASWPTGIAMGSQGSLSILKGFNL-V  339
44  ---LDIASVPFIGSVQAPVTIPTLPTV-DT----WVSWPTGVAMGSQGSLSILKGFNL-V  340
45  ---VPDPGNPI------TVQLPTLPTL-DT----WVSWPTGIAMGSQGSLRAYP--KI-P  330
46  ---------------------TIDGPGEDT----FVTMPAGIAVGSQGALSIIQNYTT-V  309
47  ---------------A-----AESQPGEET----WVTMPAGLALGSQGALSIIENYTI-I  309
48  ---------------------TIDGPGEDT----FVTMPAGIAVGSQGALSIIQNYTT-A  307
49  ---------------------TIDGPGEDT----FVTMPAGIAVGSQGALSIIQNYTT-V  309
50  ---------------------PNDGPGEDT----FVTMPAGIAVGSQGALSIIQNYTT-A  307
51  ---------------AGGPGSPGMQPGIDV----WVSMPAGVAVGSQGALSIIRNFTT-V  312
52  -------------------ANGEVNMARNV----WLTMPAGLAMGSQGSLSILTNFEN-V  312
53  --------------------LVDGKGQDCW----VAYWPVGVALESQGGVARLQQVG---  309
54  --------------------SGATSSQMRV----TYTFPVATAMGTGSALAVLKNVV--V  313
55  --------------------LVDGKAQDNL----TVHWAVGLALASDQAIARLKQVAK-A  313
56  ------------------------------------------------------------  258
57  ---------------VGQ-----TLPVRDM----WVSFPSGLAIGSQGAISMIQNFEPLV  314
58  --------------------SAATTSRMNV----TYTYPTATAMGCASALAILENVL--V  317
59  --------------------DVDGKGQRSW----VVSWPVSVALESQSGVARLQKVG---  309
60  -------------------ANQTVNMTKDI----WLTMPAGLALGSQGALSMLTNFEH-V  313
61  --------------------LIDGKAQDTN----TLYWAVGLALAAEQAVARLQKVS---  311
62  ---------------I----QPDAGPGEDT----FVTMPAGIAVGSQGALSIIQNYTT-V  312
63  -------------------ANAEVNMARNI----WLTMPAGLALGSQGCLSILTNFAN-V  312
64  --------------------LIDGKGQDGW----IAYWPVGVALESQAGVARLQQVG---  309
65  --------------------LVDGKAQDQQ----IVSWAVGLALASEQAIARLEKVAS-S  313
66  ---------------GGG-GQPAMLPGVDV----WVSTPAGYAVGSQGALSIIRNFTP-V  311
67  ---------------AGI---GDPIAADDV----FLGWPVGTAVGSPAAISLLQNFSI-A  317
68  --------------------TLSQGSGIRY----DVVFPVAISVPAVSDIAVISNVLV--  307
69  --------------------TLSQGSGTRY----DVVFPVAISVPAVTDIAVISNVLV--  323
70  --------------------PSADAGTLSSRNAVSVIWPMGIAYGSAGAVSRLANITP-L  323
71  --------------------RGADTGTLASRHAVSVIWPMGIAYGSAGAVSRLANITP-L  374
72  --------------------RGADTGTLASRHAVSVIWPMGIAYGSAGAVSRLANITP-L  369
```

```
73   --------------------RLADAGTLSSRQAISCIWPMGIAYGSAGAVSRLANITP-L  323
74   --------------------TVQTKDASRT----RLKWYAALALKSTKSLARIKGVTN-S  300
75   ---------------------------------------------HQGSG----------  259
76   SQQVLVPDVPTTITVNGATITPSLNAVTTN----WVSWPCGMAMGAQGSLSILKGFGL-V  277
77   -------------------ANQTVNMT---------------------------------  285
78   --------------------THATKDAMSF----RMKWYVGTALKATHAVARLKGITN-I  299
79   ------------------------------------------------------------  223
80   --------------------TVQNKDATRT----RVKWYCGLALKSTRSLARVRGVTN-V  300
81   --------------------TVQNKDATRT----RLKWYCGTALKSTKSLARIQGIQN-I  301
82   --------------------TVQNKDAQRF----RMKWYVGTALKSTKSLARLKGVTT-V  300
83   --------------------TVQNKDASRY----RVKWYAGTALKATHSVARLKGILN-I  300
84   --------------------TIQNKDASRY----RVKWYAGTALKATHSVARLKGILN-I  300
85   --------------------TIQNKDASRY----RVKWYAGTALKATHSVARLKGILN-I  300
86   --------------------TIQNKDASRY----RVKWYAGTALKATHSVARLKGILN-I  300
87   --------------------SLTPTSTITR----ALHWPVGYALGSMGAVSMLENITP-L  310
88   --------------------SGATTSQTRV----TYTFPVATAMGTGSALAILKNVV--V  264
89   --------------------TIQNKDASRY----RVKWYAGTALKATHSVARLKGILN-I  300
90   --------------------TIQNKDAVRY----RVKWYVGTALKATHSVARLQGVTN-V  300
91   ---------------AGA-GAPDMLPGVDT----WVSMPAGLALGSQGALSIIENYTT-V  272
92   --------------------SLTPTSTITR----ALHWPVGYALGSMGAVSMLENITP-L  310
93   --------------------SLMPTSTITR----ALHWPVGYALGSMGAVSMLENITP-L  310
94   --------------------TVQNKDAIRY----RVKWYAGLALKSTKSLAKVADVAM-G  297
95   --------------------TIQNKDASRY----RVKWYAATALKATHSVARLKGILN-I  300
96   --------------------TVQNKDATRI----RMKWYTGLALKSTKSVARLKGVTN-I  300
97   --------------------KLQGKDAQRW----RLKWYCGTALKATHSVARLKGITN-V  300
98   --------------------TVQNKDATRT----RLKWYCGLALKSTKSLAAVKGITN-V  300
99   --------------------KLEGKDAQRW----RLKWYAGTVLRATHSVARLKGITN-V  300
100  --------------------TVQNKDATRT----RLKWYVGLALKSTHSLAAIKGITN-V  300
101  --------------------LVQNKDARRT----RLKWYCGIALKSTKSLARIKGITN-I  300
102  -------------------EGASGASELNV----DYIFTAGVALGQEMALSIMRNARE--  321
103  --------------------TVQNKDATRT----RLKWYCGLALKSTKSLAAVKGITN-V  300
104  --------------------TVQNKDATRI----RLKWYVGLVLKSTKSLARLKGVTN-I  300
105  --------------------TVQNKDATRT----RIKWYCGLALKSTRSIAAIRGVTN-I  300
106  --------------------TVQNKDATRT----RIKWYCGLALKSTRSIAAIRGVTN-I  298
107  --------------------TVQNKDATRI----RLKWYAGLALKSTRSIARLKGVTN-I  295
108  --------------------TVQNKDATRT----RIKWYCGLALKSTRSIAAIRGVT---  298
109  --------------------TVQNKDATRT----RIKWYCGLALKSTRSIAAIRGVTN--  299
110  --------------------TVQNKDATRT----RIKWYCGLALKSTRSIAAIRGVTN-I  300
111  --------------------TVQNKDATRT----RLKWYTATALKSTKSLARLRGITN-I  299
112  --------------------TVQNKDATRT----RIKWYCGLALKSTRSIAAIRGVTN-I  302
113  --------------------TVQNKDATRT----RIKWYCGLALKSTRSIAAIRGVTN-I  300
114  --------------------TVQNKDATRT----RIKWYCGLALKSTRSIAAIRGV----  297
115  --------------------TVQNKDATRT----RLKWYCGLALKATKSLAAIRGVTN-V  301
116  --------------------TVQNKDATRT----RIKWYCGLALKSTKSLAALKGVPN-I  297
117  --------------------TVQNKDATRI----RLKWYVGLVLKSTKSLARLKGVTN-I  300
118  --------------------TVQNKDATRI----RLKWYVGLVLKSTKSLARLKGVTN-I  300
119  --------------------TVQNKDATRT----RIKWYCGLALKSTKSLAALKGVPN-I  297
120  --------------------TVQNKDAIRT----RLKWYCGLALKSTKSLARLAGVTN-V  301
121  --------------------TVQNKDATRT----RIKWYCGLALKSTRSIAAIRG-----  296
122  --------------------TVQNKDATRT----RIKWYCGLALKSTRSIAAIRGVTN-I  300
123  --------------------TVQNKDATRI----RLKWYCGLALKSTKSIARLKGVTN-I  300
124  --------------------TVQNKDATRT----RIKWYCGLALKSTRSIAAIRGVTN-I  300
125  --------------------TVQNKDATRT----RIKWYCGLALKSTRSIAAIRGVTN-I  300
126  --------------------TVQNKDATRT----RIKWYCGLALKSTRSIAAIR------  295
127  --------------------KLEGKDAQRW----RLKWYCGTALKRTTSVARLKGVTN-I  300
128  --------------------KLEGKDAQRW----RLKWYCGTALKRTTSVARLKGVTN-I  300
129  --------------------TVQNKDADRI----RVKWYCGTALKSTRSLARLRGVTN-I  300
130  --------------------TVQNKDADRI----RVKWYCGTALKSTRSLARLRGVTN-I  300
131  --------------------TVQNKDADRI----RVKWYCGTALKSTRSLARLRGVTN-I  300
132  --------------------TVQNKDATRT----RIKWYCGLALKSTRSI----------  291
133  --------------------VHQTKDERIW----RVKWYCGLALFSQLGVASADGIRD--  305
134  --------------------TVQNKDADRI----RVKWYCGTALKSTRSLARLRGVTN-I  300
135  --------------------TVQNKDATRT----RIKWYCGLALKSTR------------  289
136  --------------------TVQNKDAIRT----RLKWYCGLALKSTKSLAALTNVPN-V  296
137  --------------------TVQNKDVERY----RMKWYCGTALKSTRSLARLKGITN-I  300
138  --------------------TVQNKDATRI----RLKWYTGLALKSTRSIARLKGVTN-I  303
139  --------------------TIQNKDAVRY----RVKWYVGTALKATHSVARLAGITN-I  300
```

```
140  --------------------TVQNKDAVRT----RVKWYCGLANKHDYAIARIDNVKF--  296
141  --------------------TVQNKDAWRY----RLKWYVGLALKSTRSMARLKGITN-V  302
142  --------------------TLEDKDATRY----RLKWYAGLVLKATHGVARLKGLTN-I  300
143  --------------------TVQNKDAVRT----RVKWYCGLANKHDYAIARIDNVKF--  296
144  --------------------THHTKDARKW----RVKWYAGTALKATHSVARLKGITN-I  300
145  --------------------TVQNKDAERW----RVKWYVGSALKATHSVARLRAISN-V  300
146  --------------------TVQNKDVERY----RMKWYVGTALKSTKSLARLKGITN-I  300
147  --------------------TVQNKDATRT----RVKWYCSLANKHDKAIAALTNVKI--  293
148  --------------------TVQNKDATRT----RVKWYCSLANKHDKAIAALTNVKI--  293
149  --------------------TVQNKDATRT----RVKWYCSLANKHDKAIAALTNVKI--  293
150  --------------------TVQNKDATRT----RVKWYCSLANKHDKAIAALTNVKI--  293
151  --------------------TVQNKDAVRT----RVKWYCSLANKHDKAIAALNNVKI--  306
152  --------------------TVQNKDATRT----RVKWYCSLANKHDKAIAALTNVKI--  293
153  --------------------THHTKDARKW----RVKWYAGTALKATHSVARLKGITN-I  300
154  --------------------TVQNKDAVRT----RVKWYCSLANKHDKAIAALTNVKI--  293
155  --------------------TVQNKDAVRT----RVKWYCSLANKHDKAIAALNNVKI--  306
156  --------------------TVQNKDATRT----RVKWYCSLANKHDKAIAALTNVKI--  293
157  --------------------TVQNKDATRT----RVKWYCSLANKHDKAIAALTNVKI--  293
158  --------------------TVQNKDAVRT----RVKWYCSLANKHDKAIAALNNVKI--  306
159  --------------------TVQNKDAVRT----RVKWYCSLANKHDKAIAALNNVKI--  306
160  --------------------TVQNKDAVRT----RVKWYCSLANKHDKAIAALNNVKI--  306
161  --------------------TVQNKDATRI----RLKWYVGLVLKSTK------------  289
162  --------------------TVQTKDSSRT----RLKWYCGLALKSTKSMARLKGLTN-V  307
163  --------------------TVQNKDAVRT----RVKWYCSLANKHDKAIAALNNVKI--  306
164  --------------------TVQNKDATRT----RVKWYCSLANKHDKAIAALTNVKI--  293
165  --------------------TVQNKDATRT----RVKWYCSLANKHDKAIAALTNVKI--  293
166  --------------------TVQNKDATRT----RVKWYCSLANKHDKAIAALTNVKI--  293
167  --------------------TVQNKDATRT----RVKWYCSLANKHDKAIAALTNVKI--  293
168  --------------------TVQNKDATRT----RVKWYCSLANKHDKAIAALTNVKI--  293
169  --------------------TVQNKDATRT----RVKWYCSLANKHDKAIAALTNVKI--  293
170  --------------------TVQNKDAVRT----RVKWYCSLANKHDKAIAALTNVKI--  291
171  --------------------TVQNKDATRT----RVKWYCSLANKHDKAIAALTNVKI--  293
172  --------------------TVQNKDAVRT----RVKWYCSLANKHDKAIAALNNVKI--  306
173  --------------------TVQNKDAVRT----RVKWYCSLANKHDKAIAALTNVKI--  291
174  --------------------TVQNKDAVRT----RVKWYCSLANKHDKAIAALTNVKI--  291
175  --------------------TVQNKDAVRT----RVKWYCSLANKHDKAIAALTNVKI--  291
176  --------------------TVQNKDATRT----RVKWYCSLANKHDKAIAALTNVKI--  293
177  --------------------TVQNKDAVRT----RVKWYCSLANKHDKAIATLNNVKI--  306
178  --------------------TVQNKDAVRT----RVKWYCSLANKHDKAIAALNNV----  304
179  --------------------TVQNKDATRT----RVKWYCSLANKHDKAIAALTNVKI--  291
180  --------------------TVQNKDATRT----RVKWYCSLANKHDKAIAALTNVKI--  293
181  --------------------TVQNKDAVRT----RVKWYCSLANKHDKAIAALTNVKI--  291
182  --------------------TVQNKDAVRT----RVKWYCSLANKHDKAIAALTNVKI--  291
183  --------------------TVQNKDAVRT----RVKWYCSLANKHDKAIAALTNVKI--  291
184  --------------------TVQNKDAVRT----RVKWYCSLANKHDKAIAALTNVKI-Q  292
185  --------------------TVQNKDAVRT----RVKWYCSLANKHDKAIATLNNVKI--  306
186  --------------------TVQNKDAVRT----RVKWYCSLANKHDKAIAALTNVKI--  291
187  --------------------TVQNKDAVRT----RVKWYCSLANKHDKAIAALTNVKI--  291
188  --------------------TVQNKDAVRT----RVKWYCSLANKHDKAIAALTNVKI--  291
189  --------------------TVQNKDAVRT----RVKWYCSLANKHDKAIAALTNVKI--  291
190  --------------------PMETKDARRH----RIKWYCGLALFRKAALARLKGIRP-L  296
191  --------------------TVQNKDAVRT----RVKWYCSLANKHDKAIAALNNVKI--  306
192  --------------------TSHVKDADLH----RLKWYCGTALKATHALARLKGVTN-I  299
193  --------------------TVQNKDAVRT----RVKWYCSLANKHDKAIAALTNVKI--  291
194  --------------------TVQNKDAVRT----RVKWYCSLANKHDKAIAALTNVKI--  291
195  --------------------TVQNKDAVRT----RVKWYCSLANKHDKAIAALTNVKI--  291
196  --------------------TVQNKDAVRT----RVKWYCSLANKHDKAIAALTNVKI--  291
197  --------------------TVQNKDAVRT----RVKWYCSLANKHDKAIAALTNVKI--  291
198  --------------------TVQNKDAVRT----RVKWYCSLANKHDKAIAALTNVKI--  291
199  --------------------TVQNKDAVRT----RVKWYCSLANKHDKAIAALTNVKI--  291
200  --------------------TVQNKDAVRT----RVKWYCSLANKHDKAIAALTNVKI--  291
201  --------------------TVQNKDAVRT----RVKWYCSLANKHDKAIAALTNVKI--  291
202  --------------------THATKDAHKW----RLKWYAGTALKATHSVARLKGILN-I  299
203  --------------------TVQNKDAVRT----RVKWYCSLANKHDKAIAALTNVKI--  291
204  --------------------TVQNKDATRI----RLKWYAGLALKSTKSLARLKGVTN-I  302
205  --------------------TVQNKDAVRT----RVKWYCSLANKHDKAIAALTNVKI--  291
206  --------------------TVQNKDAVRT----RVKWYCSLANKHDKAIAALTNVKI--  291
```

```
207 --------------------TVQNKDAVRT----RVKWYCSLANKHDKAIAALTNVKI-- 291
208 --------------------TVQNKDAVRT----RVKWYCSLANKHDKAIAALTNVKI-- 291
209 --------------------TVQNKDAVRT----RVKWYCSLANKHDKAIAALTNVKI-- 291
210 --------------------TVQNKDAVRT----RVKWYCSLANKHDKAIAALTNVKI-- 291
211 --------------------TVQNKDAVRT----RVKWYCSLANKHDKAIAALTNVKI-- 291
212 --------------------TVQNKDAVRT----RVKWYCSLANKHDKAIAALTNVKI-- 291
213 --------------------TVQNKDAVRT----RVKWYCSLANKHDKAIAALTNVKV-- 291
214 --------------------TVQNKDAVRT----RVKWYCSLANKHDKAIAALNNVKI-- 306
215 --------------------TVQNKDAVRT----RVKWYCSLANKHDKAIAALTNVKI-- 291
216 --------------------TVQNKDAVRT----RVKWYCSLANKHDKAIAALTNVKI-- 291
217 --------------------TVQNKDAVRT----RVKWYCSLANKHDKAIAALTNVKI-- 291
218 --------------------TVQNKDAVRT----RVKWYCSLANKHDKAIAALTNVKI-- 291
219 --------------------TVQNKDAVRT----RVKWYCSLANKHDKAIAALTNVKI-- 291
220 --------------------TVQNKDAVRT----RVKWYCSLANKHDKAIAALTNVKI-- 291
221 --------------------TVQNKDAVRT----RVKWYCSLANKHDKAIAALTNVKI-- 291
222 --------------------TVQNKDAVRT----RVKWYCSLANKHDKAIAALTNVKI-- 291
223 --------------------TVQNKDAVRT----RVKWYCSLANKHDKAIAALTNVKI-- 291
224 --------------------TVQNKDAVRT----RVKWYCSLANKHDKAIAALTNVKI-- 291
225 --------------------TVQNKDAVRT----RVKWYCSLANKHDKAIAALTNVKI-- 291
226 --------------------TVQNKDAVRT----RVKWYCSLANKHDKAIAALTNVK--- 290
227 --------------------TVQNKDAVRT----RVKWYCSLANKHDKAIAALTNVKI-- 291
228 --------------------TVQNKDAIRT----RLKWYCGLALKSTKSLACLKNVQI-G 293
229 --------------------TVQNKDAVRT----RVKWYCSLANKHDKAIAALTNVKI-- 291
230 --------------------TVQNKDAIRT----RLKWYCGLALKSTKSLACLKNVQI-G 293
231 --------------------TVQNKDAVRT----RVKWYCSLANKHDKAIAALTNVKI-- 291
232 --------------------TVQNKDAVRT----RVKWYCSLANKHDKAIAALNNVKI-- 306
233 --------------------TVQNKDAVRT----RVKWYCSLANKHDKAIAALTNVKI-- 291
234 --------------------TVQNKDAVRT----RVKWYCSLANKHDKAIAALTNVKI-- 291
235 --------------------TVQNKDAVRT----RVKWYCSLANKHDKAIAALTNVKI-- 291
236 --------------------TVQNKDAVRT----RVKWYCSLANKHDKAIAALTNVKI-- 291
237 --------------------TVQNKDAVRT----RVKWYCSLANKHDKAIATLTNVKI-- 291
238 --------------------TVQNKDAVRT----RVKWYCSLANKHDKAIAALTNVKI-- 291
239 --------------------ALETKNARRW----RVRWYVGLALFSTVAVAQLQGITA-N 295
240 --------------------TVQNKDAVRT----RVKWYCSLANKHDKAIAALNN----- 303
241 --------------------TVQNKDATRT----RVKWYCSLANKHDKAIAALTNVKI-- 291
242 --------------------TVQNKDAVRT----RVKWYCSLANKHDKAIAALTNVKI-- 291
243 --------------------TVQNKDAVRT----RVKWYCSLANKHDKAIAALTNVKI-- 291
244 --------------------TVQNKDAVRT----RVKWYCSLANKHDKAIAALTNVKI-- 291
245 --------------------TVQNKDAVRT----RVKWYCSLANKHDKAIAALTNVKI-- 291
246 --------------------TVQNKDAVRT----RVKWYCSLANKHDKAIAALTNVKI-- 291
247 --------------------TVQNKDAVRT----RVKWYCSLANKHDKAIAALTNVKI-- 291
248 --------------------TVQNKDAVRT----RVKWYCSLANKHDKAIAALTNV---- 289
249 --------------------TVQNKDAVRT----RVKWYCSLANKHDKAIAALTNVKI-- 291
250 --------------------TVQNKDAVRT----RVKWYCSLANKHDKAIAALTNVKI-- 291
251 --------------------TVQNKDAVRT----RVKWYCSLANKHDKAIAALTNVKI-- 291
252 --------------------TVQNKDAVRT----RVKWYCSLANKHDKAIAALTNVKI-- 291
253 --------------------TVQNKDAVRT----RVKWYCSLANKHDKAIAALTNVKI-- 291
254 --------------------TVQNKDAVRT----RVKWYCSLANKHDKAIAALTNVK--- 290
255 --------------------TVQNKDAVRT----RVKWYCSLANKHDKAIAALTNVKI-- 291
256 --------------------TVQNKDATRT----RVKWYCSLANKHDKAIAALTNVKI-- 291
257 --------------------TVQNKDATRT----RVKWYCSLANKHDKAIAALTNVKI-- 291
258 --------------------TVQNKDAVRT----RVKWYCSLANKHDKAIAALTNVKI-- 291
259 --------------------TVQNKDAVRT----RVKWYCSLANKHDKAIAALTNVKI-- 291
260 --------------------TVQNKDAVRT----RVKWYCSLANKHDKAIAALTNVKI-- 291
261 --------------------TVQNKDAVRT----RVKWYCSLANKHDKAIAALTNVKI-- 292
262 --------------------TVQNKDAVRT----RVKWYCSLANKHDKAIAALTNVKI-- 291
263 --------------------TVQNKDAVRT----RVKWYCSLANKHDKAIAALKNVKI-- 293
264 --------------------TVQNKDAVRT----RVKWYCSLANKHDKAIAALTNVKI-- 293
265 --------------------TVQNKDAVRT----RVKWYCSLANKHDKAIAALTNVKI-- 292
266 --------------------TVQNKDAVRT----RVKWYCSLANKHDKAIAALTNVK--- 290
267 --------------------TVQNKDAVRT----RVKWYCSLANKHDKAIAALTNVKI-- 292
268 --------------------TVQNKDAVRT----RVKWYCSLANKHDKAIAALTNVKI-- 292
269 --------------------TVQNKDATRT----RVKWYCSLANKHDKAIAALTNVKI-- 291
270 --------------------TVQNKDAIRT----RLKWYCGLALKSTKSLACLKNVQI-G 293
271 --------------------TVQNKDAVRT----RVKWYCSLANKHDKAIAALTNVKI-- 291
272 --------------------TVQNKDAVRT----RVKWYCSLANKHDKAIAALTNVKI-- 291
273 --------------------TVQNKDAVRT----RVKWYCSLANKHDKAIAALTNVKI-- 292
```

```
274  --------------------TVQNKDAVRT----RVKWYCSLANKHDKAIAALTNVKI--  292
275  --------------------TVQNKDAVRT----RVKWYCSLANKHDKAIAALTNVKI--  291
276  --------------------TVQNKDAVRT----RVKWYCSLANKHDKAIAALTNVKI--  291
277  --------------------TVQNKDAVRT----RVKWYCSLANKHDKAIAALTNVKI--  291
278  --------------------TVQNKDAVRT----RVKWYCSLANKHDKAIAALTNVKI--  292
279  --------------------TVQNKDAVRT----RVKWYCSLANKHDKAIAALTNVKI--  291
280  --------------------TVQNKDAVRT----RVKWYCSLANKHDKAIAALTNVKI--  291
281  --------------------TVQNKDATRT----RIKWYCG-------------------  282
282  --------------------TVQNKDAVRT----RVKWYCSLANKHDKAIAALTNVKI--  292
283  --------------------TVQNKDAVRT----RVKWYCSLANKHDKAIAALTNVKI--  291
284  --------------------TVQNKDAVRT----RVKWYCSLANKHDKAIAALTNVKI--  291
285  --------------------TVQNKDAVRT----RVKWYCSLANKHDKAIAALTNV----  289
286  --------------------TVQNKDATRT----RVKWYCSLANKHDKAIAALTNVKI--  291
287  --------------------TVQNKDAVRT----RVKWYCSLANKHDKAIAALTNVKI--  292
288  --------------------TVQNKDATRT----RVKWYCSLANKHDKAIAALTNVKI--  291
289  --------------------TVQNKDAVRT----RVKWYCSLANKHDKAIAALTNVKI--  292
290  --------------------TVQNKDAVRT----RVKWYCSLANKHDKAIAALTNVKI--  291
291  --------------------TVQNKDAVRT----RVKWYCSLANKHDKAIAALTNVKI--  291
292  --------------------TVQNKDAVRT----RVKWYCSLANKHDKAIAALTNVKI--  291
293  --------------------TVQNKDAVRT----RVKWYCSLANKHDKAIAALTNVKI--  291
294  --------------------TVQNKDAVRT----RVKWYCSLANKHDKAIAALTNVKI--  291
295  --------------------TVQNKDAVRT----RVKWYCSLANKHDKAIAALTNVKI--  292
296  --------------------TVQNKDAVRT----RVKWYCSLANKHDKAIAALTNVKI--  292
297  --------------------TVQNKDAVRT----RVKWYCSLANKHDKAIAALTNVKI--  292
298  --------------------TVQNKDAVRT----RVKWYCSLANKHDKAIAALTNV----  289
299  --------------------TVQNKDAVRT----RVKWYCSLANKHDKAIAALTNVKI--  292
300  --------------------TVQNKDAVRT----RVKWYCSLANKHDKAIAALTNVKI--  285
301  --------------------TVQNKDAVRT----RVKWYCSLANKHDKAIAALTNVKI--  292
302  --------------------TVQNKDAVRT----RVKWYCSLANKHDKAIAALTNVKI--  292
303  --------------------TVQNKDATRT----RVKWYCSLANKHDKAIAALTNVKI--  291
304  --------------------TVQNKDAVRT----RVKWYCSLANKHDKAIAALTNVKI--  292
305  --------------------TVQNKDAVRT----RVKWYCSLANKHDKAIAALTNVKI--  292
306  --------------------TVQNKDAVRT----RVKWYCSLANKHDKAIAALTNVKI--  292
307  --------------------TVQNKDATRT----RVKWYCSLANKHDKAIAALTNVKI--  291
308  --------------------TVQNKDAVRT----RVKWYCSLANKHDKAIAALTNVKI--  292
309  --------------------TVQNKDATRT----RVKWYCSLANKHDKAIAALTNVKI--  291
310  --------------------TVQNKDATRT----RVKWYCSLANKHDKAIAALTNVKI--  291
311  --------------------TVQNKDATRT----RVKWYCSLANKHDKAIAALTNVKI--  291
312  --------------------TVQNKDAVRT----RVKWYCSLANKHDKAIAALTNVKI--  292
313  --------------------TVQNKDAVRT----RVKWYCSLANKHDKAIAALTNVKI--  291
314  --------------------TVQNKDATRT----RVKWYCSLANKHDKAIAALTNVKI--  291
315  --------------------TVQNKDAVRT----RVKWYCSLANKHDKAIAALTDVKI--  291
316  --------------------TVQNKDAVRT----RVKWYCSLANKHDKAIAALTNVKI--  292
317  --------------------TVQNKDAVRT----RVKWYCSLANKHDKAIAALTNVKI--  293
318  --------------------TVQNKDAVRT----RVKWYCSLANKHDKAIAALTNVKI--  292
319  --------------------TVQNKDAVRT----RVKWYCSLANKHDKAIAALTNVKI--  292
320  --------------------TVQNKDATRT----RVKWYCSLANKHDKAIAALTNVKI--  291
321  --------------------TVQNKDAVRT----RVKWYCSLANKHDKAIAALTNVKI--  292
322  --------------------TVQNKDATRT----RVKWYCSLANKHDKAIAALTNVKI--  291
323  --------------------TVQNKDATRT----RVKWYCSLANKHDKAIAALTNVKI--  291
324  --------------------TVQNKDAVRT----RVKWYCSLANKHDKAIAALTN-----  288
325  --------------------TVQNKDAVRT----RVKWYCSLANKHDKAIAALTNVKI--  291
326  --------------------TVQNKDAVRT----RVKWYCSLANKHDKAIAALTNVKI--  292
327  --------------------TVQNKDAVRT----RVKWYCSLANKHDKAIAALTNVKI--  292
328  --------------------TVQNKDAVRT----RVKWYCSLANKHDKAIAALTNVKI--  292
329  --------------------TVQNKDATRT----RVKWYCSLANKHDKAIAALTNVKI--  291
330  --------------------TVQNKDATRT----RVKWYCSLANKHDKAIAALTNVKI--  291
331  --------------------TVQNKDAVRT----RVKWYCSLANKHDKAIAALTNVKI--  292
332  --------------------TVQNKDATRT----RVKWYCSLANKHDKAIAALTNVKI--  291
333  --------------------TVQNKDATRT----RVKWYCSLANKHDKAIAALTNVKI--  291
334  --------------------TVQNKDAVRT----RVKWYCSLANKHDKAIAALTNVKI--  293
335  --------------------TVQNKDATRT----RVKWYCSLANKHDKAIAALTNVKI--  291
336  --------------------TVQNKDAVRT----RVKWYCSLANKHDKAIAALTNV----  289
337  --------------------TVQNKDATRT----RVKWYCSLANKHDKAIAALTNVKI--  291
338  --------------------TVQNKDAVRT----RVKWYCSLANKHDKAIAALTNVKI--  292
339  --------------------TVQNKDATRT----RVKWYCSLANKHDKAIAALTNVKI--  291
340  --------------------TVQNKDAVRT----RVKWYCSLANKHDKAIAALTNVKR--  292
```

```
341  --------------------TVQNKDATRT----RVKWYCSLANKHDKAIAALTNVKI--  291
342  --------------------TVQNKDAVRT----RVKWYCSLANKHDKAIAAL-------  301
343  --------------------TVQNKDATRT----RVKWYCSLANKHDKAIAALTNVKI--  291
344  --------------------TVQNKDATRT----RVKWYCSLANKHDKAIAALTNVKI--  291
345  --------------------TVQNKDAVRT----RVKWYCSLANKHDKAIAALTNVK---  291
346  --------------------TVQNKDAVRT----RVKWYCSLANKHDKAIAALTNVKI--  291
347  --------------------TVQNKDAVRT----RVKWYCSLANKHDKAIAALTN-----  288
348  --------------------TVQNKDAVRT----RVKWYCSLANKHDKAIAALTNVKI--  293
349  --------------------TVQNKDATRT----RVKWYCSLANKHDKAIAALTNVKI--  291
350  --------------------TVQNKDATRT----RVKWYCSLANKHDKAIAALTNVK---  290
351  --------------------TVQNKDATRT----RIKWYCGLALKSTRSIAAIRGVTN-I  281
352  --------------------TVQNKDATRT----RVKWYCSLANKHDKAIAALTNVKI--  291
353  --------------------TSETKDETIT----RVKWYCGLALFSEKGLAALTGITN--  306
354  --------------------TVQNKDAVRT----RVKWYCSLANKHDKAIAALTNV----  287
355  --------------------TVQNKDAVRT----RVKWYCSLANKHDKAIAALTNVKI--  293
356  --------------------TVQNKDATRT----RVKWYCSLANKHDKAIAALTNVKI--  291
357  --------------------TVQNKDAVRT----RVKWYCSLANKHDKAIAALTNV----  290
358  --------------------TVQNKDATRT----RVKWYCSLANKHDKAIAALTNVKI--  291
359  --------------------TVQNKDAVRT----RVKWYCSLANKHDKAIAALTNVKI--  292
360  --------------------TVQNKDATRT----RVKWYCSLANKHDKAIAALTNVKI--  291
361  --------------------TVQNKDATRT----RVKWYCSLANKHDKAIAALTNVKI--  293
362  --------------------TVQNKDAVRT----RVKWYCSLANKHDKAIAALTNVKI--  292
363  --------------------TVQNKDATRT----RVKWYCSLANKHDKAIAALTNVKT--  291
364  --------------------TVQNKDATRT----RVKWYCSLANKHDKAIAALTNVKI--  291
365  --------------------TVQNKDATRT----RVKWYCSLANKHDKAIAALTNVKI--  291
366  --------------------TVQNKDATRT----RVKWYCSLANKHDRAIAALTNVKI--  291
367  --------------------TVQNKDAVRT----RVKWYCSLANKHDKAIAALTNVKI--  293
368  --------------------TVQNKDAVRT----RVKWYCSLANKHDKAIAALTNVKI--  293
369  --------------------TVQNKDATRT----RVKWYCSLANKHDKAIAALTNVKI--  291
370  --------------------TVQNKDATRT----RVKWYCSLANKHDKAIAALTNVKI--  291
371  --------------------TVQNKDATRT----RVKWYCSLANKHDKAIAALTNVKI--  291
372  --------------------TVQNKDATRT----RVKWYCSLANKHDKAIAALTNVKI--  291
373  --------------------TVQNKDATRT----RVKWYCSLANKHDKAIAALTNVKI--  291
374  --------------------TVQNKDAVRT----RVKWYCSLANKHDKAIAALTNVKI--  291
375  --------------------TVQNKDATRT----RVKWYCSLANKHDKAIAALTNVKI--  291
376  --------------------TVQNKDAVRT----RVKWYCSLANKHDKAIVALTNVKI--  291
377  --------------------ALETKNARRW----RVRWYCGLALFSTVAVARLQGIAA-N  295
378  --------------------TVQNKDATRT----RVKWYCSLANKHDKAIAALTNVKI--  291
379  --------------------TVQNKDATRT----RVKWYCSLANKHDKAIAALTNVKI--  291
380  --------------------TVQNKDAVRT----RVKWYCSLANKHDKAIAALTNVKI--  293
381  --------------------TVQNKDAVRT----RVKWYCSLANKHDKAIAALTNVKI--  293
382  --------------------EAEAKDEHIW----RVKWYCGLALFSELGLSAAEGILN--  305
383  --------------------TVQNKDAVRT----RVKWYCSLANKHDKAIAALTNVKI--  293
384  --------------------TVQNKDAVRT----RVKWYCSLANKHDKAIAALTNVKI--  293
385  --------------------TVQNKDATRT----RVKWYCSLANKHDKAIAALTNVKI--  291
386  --------------------TVQNKDAVRT----RVKWYCSLANKHDKAIAALTNVKI--  291
387  --------------------TVQNKDATRT----RLKWYCNLALKSTKSLARLYGVSN--  306
388  --------------------TVQNKDAVRT----RVKWYCSLANKHDKAIAALTNVKI--  293
389  --------------------TVQNKDAVRT----RVKWYCSLANKHDKAIAALTNVKI--  292
390  --------------------VHQSKDERIW----RVKWYCGLALFSQLGIAAADGIRN--  305
391  --------------------TVQNKDAVRT----RVKWYCSLANKHDKAIAALTNVKI--  293
392  --------------------TVQNKDATRT----RVKWYCSLANKHDKAIAALTKVKI--  291
393  --------------------TVQNKDATRT----RVKWYCSLANKHDKAIAALTNVKI--  291
394  --------------------TVQNKDAVRT----RVKWYCSLANKHDKAIAALTNVKI--  293
395  --------------------TVQNKDAVRT----RVKWYCSLANKHDKAIAALTNVKI--  293
396  --------------------TVQNKDAVRT----RVKWYCSLANKHDKAIAALTNVKI--  305
397  --------------------EKEDADESIT----RISWYCGLALFSEKGLAVLKGVIP-G  310
398  --------------------TVQNKDAVRT----RVKWYCSLANKHDKAIAALT------  287
399  --------------------TVQNKDAVRT----RVKWYCSLANKHDKAIAALTNVKI--  293
400  --------------------TVQNKDAVRT----RVKWYCSLANKHDKAIAALTNVKI--  293
401  --------------------TVQNKDAVRT----RVKWYCSLANKHDKAIAALTNVKI--  293
402  --------------------TVQNKDAVRT----RVKWYCSLANKHDKAIAALTNVKI--  293
403  --------------------TVQNKDAVRT----RVKWYCSLANKHDKAIAALTN-----  289
404  --------------------TVQNKDAVRT----RVKWYCSLANKHDKAIAALTNVKI--  292
405  --------------------TVQNKDAVRT----RVKWYCSLANKHDKAIAAL-------  286
406  --------------------TVQNKDAVRT----RVKWYCSLANKHDKAIAALTNVKI--  293
407  --------------------TVQNKDAVRT----RVKWYCSLANKHDKAIAALTNV----  290
```

```
408 --------------------TVQNKDATRT----RVKWYCSLANKHDKAIAALTNVKI-- 291
409 --------------------TVQNKDAVRT----RVKWYCSLANKHDKAIAALTNVKI-- 293
410 --------------------TVQNKDAVRT----RVKWYCSLANKHDKAIAALTNVKI-- 293
411 --------------------TVQNKDAVRT----RVKWYCSLANKHDKAIAALTNVKI-- 293
412 --------------------TVQNKDAVRT----RVKWYCSLANKHDKAIAALTNVKI-- 293
413 --------------------TVQNKDAVRT----RVKWYCSLANKHDKAIAALT------ 287
414 --------------------TVQNKDAVRT----RVKWYCSLANKHDKAIAALTNVKI-- 293
415 --------------------TVQNKDAVRT----RVKWYCSLANKHDKAIAALTNVKI-- 293
416 --------------------TVQNKDAVRT----RVKWYCSLANKHDKAIAALTNVKI-- 293
417 --------------------TVQNKDAVRT----RVKWYCSLANKHDKAIAALANVKI-- 305
418 --------------------TVQNKDATRT----RVKWYCSLANKHDKAIAALTNVKI-- 291
419 --------------------LLEKRDARRW----RLRWYCGAALKATHSLARLKGVTN-V 300
420 --------------------TVQNKDAVRT----RVKWYCSLANKHNKAIAALTNVKI-- 293
421 --------------------TVQNKDAVRT----RVKWYCSLANKHDKAIAALT------ 287
422 --------------------TVQNKDAVRT----RVKWYCSLANKHD------------- 295
423 --------------------TVQNKDAVRT----RVKWYCSLANKHDKAIAALANVKI-- 305
424 --------------------TVQNKDAVRT----RVKWYCSLANKHDKAIAALTNVKI-- 293
425 --------------------TVQNKDAVRT----RVKWYCSLANK--------------- 293
426 --------------------TVQNKDAVRT----RVKWYCSLANKHD------------- 295
427 --------------------TVQNKDAVRT----RVKWYCSLA----------------- 291
428 --------------------TVQNKDAVRT----RVKWYCSLANKHDKAIAALTNV---- 291
429 --------------------TVQNKDAVRT----RVKWYCSLANKHDKAIAAL------- 286
430 --------------------TVQNKDAVRT----RVKWYCSLANKHDKAI---------- 298
431 --------------------TVQNKDAVRT----RVKWYCSLANKHDKAIAALTNVKI-- 293
432 --------------------IHQSKDEHIT----RVKWYCGLALFSLKGLAAADGITN-- 305
433 --------------------TVQNKDAVRT----RVKWYCSLANKHDKAIAAL------- 286
434 --------------------TVQNKDAVRT----RVKWYCSLANKHDKAIAALTNVKI-- 292
435 --------------------TVQNKDAVRT----RVKWYCSLANKHDKAIAALTNVKI-- 292
436 --------------------TVQNKDAVRT----RVKWYCSLANKHDKAIAALTNVKI-- 293
437 --------------------TVQNKDATRT----RVKWYVSLANKHDKAIASLNNVKI-- 291
438 --------------------TVQNKDAVRT----RVKWYCSLANKHDKAIAALTNVKI-- 293
439 --------------------TVQNKDAIRT----RLKWYCGLALKSTKSLACLKNVQI-G 293
440 --------------------TVQNKDAVRT----RVKWYCSLANKHDKAIAALT------ 287
441 --------------------ALETKNARRW----RVRWYCGLALFSTVAVARLQGIAA-N 295
442 --------------------TVQNKDSTRT----RVKWYCSLANKHDKAISALTGVAL-- 294
443 --------------------TVQNKDATRT----RVKWYCSLANKHDKAIAALTNVKI-- 291
444 --------------------ESESKDESIT----RVKFYCGLACYSDLALAVVKGISN-- 305
445 --------------------ISETKDETIW----RVKWYCGLALFSELGLAAAEGITN-- 305
446 --------------------TVQNKDAVRT----RVKWYCSLANKHDKAIA--------- 284
447 --------------------TVQNKDAVRT----RVKWYCSLANKHDKAIAA-------- 285
448 --------------------DLETKDASRW----RVKWYVSLAVFSELGVARLQGITA-N 293
449 --------------------TVQNKDAVRT----RVKWYCSLANKHDKAIA--------- 284
450 --------------------TVQNKDAVRT----RVKWYCSLANKHDKAIAAL------- 286
451 --------------------ALETKNARRW----RVRWYVGLALFSTVAVARLQGITA-N 295
452 --------------------ELETKDASRH----RIKWYAGLALFSELGVARLRGIEG-A 295
453 --------------------TVQNKDAVRT----RVKWYCSLANKHDKAIAALTKVKI-- 293
454 --------------------EKEDADESIT----RISWYCGLALFSEKGLAVLKGVIP-G 310
455 --------------------TVQNKDADRI----RVKWYCGTALKSTRSLARLQGITN-I 300
456 --------------------TVQNKDAVRT----RVKWYCSLANKHDKAIAALT------ 288
457 --------------------TVQNKDAVRT----RVKWYCSLANKHDKA----------- 282
458 --------------------TVQNKDAVRT----RVKWYCSLANKHDKAIAALTN----- 290
459 --------------------TVQNKDATRT----RLKWYVGAALKSTKSVARLEAVTN-V 300
460 --------------------TVQNKDAVRT----RVKWYCSLANKHDKAI---------- 283
461 --------------------EKEDADESIT----RISWYCGLALFSEKGLAVLKGVTP-G 310
462 --------------------TVQNKDAVRT----RVKWYCSLANK--------------- 278
463 -------------------VNQTVNMTKDI----WVSMPAGLALGSQAALSLLTNFEH-V 227
464 --------------------TVQNKDAVRT----RVKWYCSL------------------ 290
465 --------------------TVQNKDAVRT----RVKWYCSLANKHDKAIAA-------- 285
466 --------------------TVQNKDAVRT----RVKWYCSLA----------------- 276
467 --------------------TVQNKDAVRT----RVKWYCSLANKHD------------- 280
468 --------------------TVQNKDAVRT----RVKWYCSLANKHDK------------ 281
469 --------------------TVQNKDATRT----RVKWYCGLANKHEKAIAALTDVQL-- 294
470 --------------------TVQNKDAVRT----RVKWYCSLANKHDKA----------- 282
471 --------------------ASETKDNYIT----RVKWYCGLALFSEKGLAALPGVTN-- 306
472 --------------------TVQNKDATRI----RLKWYTSLALKSTRSIAALQGVTN-I 310
473 --------------------ASETKDNYIT----RVKWYCGLALFSEKGLAALPGVTN-- 306
474 --------------------TVQNKDAVRT----RVKWYCSLA----------------- 276
```

```
475   --------------------TVQNKDAVRT----RVKWYCSLANKHDKA-----------   282
476   --------------------EKEDADESIT----RISWYCGLALFSEKGLAVLKGVTP-G   310
477   --------------------ESETKDETIT----RVKMYCGLANFSQLGLYAMPGITN--   305
478   --------------------ESETKDESIT----RVKFYCGLAVYSDLALAVAQGVKN--   305
479   --------------------EKEDADESIT----RITWYCGLALFSEKGLAVLEGVTP--   309
480   --------------------PAEDKDNYIT----RVKWYCGLALFSEKGLAMADGITN--   306
481   --------------------TVQNKDAVRT----RVKWYCSLANK---------------   278
482   --------------------TVQNKDAVRT----RVKWYCSLA-----------------   276
483   --------------------TVQNKDSTRT----RVKWYCSLANKHDKALAALTGVAL--   294
484   --------------------TVQNKDAVRT----RVKWYCSLAN----------------   277
485   --------------------ELETKDASRH----RIKWYAGLALFSELGVARLRGIEG-A   295
486   --------------------TVQNKDSTRT----RVKWYCSLANKHDKAISALTGVAL--   294
487   --------------------TVQNKDAVRT----RVKWYCSLANKHDKAIAAL-------   288
488   --------------------TVQNKDATRT----RVKWYCSLANKHDKAIAALTGVAL--   294
489   --------------------TVQNKDATRT----RVKWYCSLANKHDKAISALTGVAL--   294
490   --------------------EKEDADESIT----RISWYCGLALFSEKGLAVLKGVTP-A   310
491   --------------------TVQNKDAVRT----RVKWYCSL------------------   275
492   --------------------ESETKDESIT----RVKFYCGLAVYSDLALAVAKGVTN--   305
493   --------------------TVQNKDAVRT----RVKWYCSLANK---------------   279
494   --------------------ALETKDATRW----RVKWYVGLALASDLGVARLQGITA-N   295
495   --------------------TVQNKDAVRT----RVKWYCSLA-----------------   277
496   --------------------TVQNKDAVRT----RVKWYCSLAN----------------   278
497   --------------------ESETKDNTIT----RVKMYTGMALFSELGLAAMTGVTN--   306
498   --------------------TVQNKDAVRT----RVKWYCSL------------------   275
499   --------------------TVQNKDATRT----RVKWYCSLANKHNKAIAALTGVAL--   294
500   --------------------EKEDADESIT----RISWYCGLALFSEKGLAVLKGVTP-A   310

               490       500       510
1     ---------------------------------------   288
2     ---------------------------------------   288
3     ---------------------------------------   288
4     ---------------------------------------   288
5     ---------------------------------------   288
6     ---------------------------------------   288
7     ---------------------------------------   286
8     ---------------------------------------   286
9     ---------------------------------------   286
10    ---------------------------------------   286
11    ---------------------------------------   286
12    ---------------------------------------   286
13    ---------------------------------------   287
14    ---------------------------------------   287
15    GDGY---------SSKRKG--------------------   337
16    CGK------------------------------------   330
17    CGK------------------------------------   330
18    CR-------------------------------------   329
19    SRIPL-------INFENHGA-------------------   339
20    G--------------------------------------   329
21    QRSPL-------IDFDIRGA-------------------   339
22    HRSPL-------IDFDIRGA-------------------   339
23    QRSPL-------IDFDIRGA-------------------   340
24    HRSPL-------IDFDIRGA-------------------   339
25    HRSPL-------IDFDIRRA-------------------   339
26    HRGPL-------IDFDIQGA-------------------   339
27    CR-------------------------------------   329
28    HRSPL-------IDFDIRGA-------------------   339
29    ANLT--------------TT-------------------   348
30    ANLT--------------TT-------------------   354
31    ADLP--------------TT-------------------   329
32    ADLP--------------TT-------------------   329
33    ANLA--------------AT-------------------   344
34    VDFA-----------------------------------   336
35    ---------------------------------------   332
```

36	---------------------------------------	209
37	AAYT-----------------------------------	332
38	ANLP--------------VT-------------------	338
39	---------------------------------------	300
40	SNVA-----------------------------------	324
41	---------------------------------------	286
42	ADLP--------------TT-------------------	346
43	ADLP--------------TT-------------------	345
44	ADLP--------------TT-------------------	346
45	AHL------------------------------------	333
46	GSCG--------------GG-------------------	315
47	GPCV--------------PDPA-----------------	317
48	ASCG--------------GG-------------------	313
49	ASCG--------------GGGG-----------------	317
50	ASCG--------------GGT------------------	314
51	ADCG--------------GG-------------------	318
52	GECA--------------GIVP-----------------	320
53	---------------------------------------	309
54	---------------------------------------	313
55	---------------------------------------	313
56	---------------------------------------	258
57	EDCN--------------GAELAMEAGATSAKSKSKRSK	339
58	---------------------------------------	317
59	---------------------------------------	309
60	GECA-------------GGPPPA----------------	323
61	---------------------------------------	311
62	ASCG--------------GGGG-----------------	320
63	GECT--------------GIVP-----------------	320
64	---------------------------------------	309
65	---------------------------------------	313
66	SDCS--------------VG-------------------	317
67	GPCP--------------TITPA----------------	326
68	----------QPL--------------------------	310
69	----------QPL--------------------------	326
70	---------------------------------------	323
71	---------------------------------------	374
72	---------------------------------------	369
73	---------------------------------------	323
74	GGQ----------DQSGTGKPTLV---------------	314
75	---------------------------------------	259
76	ANLT--------------TT-------------------	283
77	---------------------------------------	285
78	---------------------------------------	299
79	---------------------------------------	223
80	---------------------------------------	300
81	---------------------------------------	301
82	---------------------------------------	300
83	---------------------------------------	300
84	---------------------------------------	300
85	---------------------------------------	300
86	---------------------------------------	300
87	---------------------------------------	310
88	---------------------------------------	264
89	---------------------------------------	300
90	---------------------------------------	300
91	ASCG--------------G--------------------	277
92	---------------------------------------	310
93	---------------------------------------	310
94	----------------ESGGPLA----------------	304
95	---------------------------------------	300
96	---------------------------------------	300
97	---------------------------------------	300
98	---------------------------------------	300
99	---------------------------------------	300
100	---------------------------------------	300
101	---------------------------------------	300
102	------------------GA-------------------	323

103	---------------------------------------	300
104	---------------------------------------	300
105	---------------------------------------	300
106	---------------------------------------	298
107	---------------------------------------	295
108	---------------------------------------	298
109	---------------------------------------	299
110	---------------------------------------	300
111	---------------------------------------	299
112	---------------------------------------	302
113	---------------------------------------	300
114	---------------------------------------	297
115	---------------------------------------	301
116	---------------------------------------	297
117	---------------------------------------	300
118	---------------------------------------	300
119	---------------------------------------	297
120	---------------------------------------	301
121	---------------------------------------	296
122	---------------------------------------	300
123	---------------------------------------	300
124	---------------------------------------	300
125	---------------------------------------	300
126	---------------------------------------	295
127	---------------------------------------	300
128	---------------------------------------	300
129	---------------------------------------	300
130	---------------------------------------	300
131	---------------------------------------	300
132	---------------------------------------	291
133	---------------------------------------	305
134	---------------------------------------	300
135	---------------------------------------	289
136	---------------------------------------	296
137	---------------------------------------	300
138	---------------------------------------	303
139	---------------------------------------	300
140	---------------------------------------	296
141	---------------------------------------	302
142	---------------------------------------	300
143	---------------------------------------	296
144	---------------------------------------	300
145	---------------------------------------	300
146	---------------------------------------	300
147	---------------------------------------	293
148	---------------------------------------	293
149	---------------------------------------	293
150	---------------------------------------	293
151	---------------------------------------	306
152	---------------------------------------	293
153	---------------------------------------	300
154	---------------------------------------	293
155	---------------------------------------	306
156	---------------------------------------	293
157	---------------------------------------	293
158	---------------------------------------	306
159	---------------------------------------	306
160	---------------------------------------	306
161	---------------------------------------	289
162	A--------------------------------------	308
163	---------------------------------------	306
164	---------------------------------------	293
165	---------------------------------------	293
166	---------------------------------------	293
167	---------------------------------------	293
168	---------------------------------------	293
169	---------------------------------------	293

170	--	291
171	--	293
172	--	306
173	--	291
174	--	291
175	--	291
176	--	293
177	--	306
178	--	304
179	--	291
180	--	293
181	--	291
182	--	291
183	--	291
184	SV--------------------------------------	294
185	--	306
186	--	291
187	--	291
188	--	291
189	--	291
190	S---------------------------------------	297
191	--	306
192	--	299
193	--	291
194	--	291
195	--	291
196	--	291
197	--	291
198	--	291
199	--	291
200	--	291
201	--	291
202	--	299
203	--	291
204	--	302
205	--	291
206	--	291
207	--	291
208	--	291
209	--	291
210	--	291
211	--	291
212	--	291
213	--	291
214	--	306
215	--	291
216	--	291
217	--	291
218	--	291
219	--	291
220	--	291
221	--	291
222	--	291
223	--	291
224	--	291
225	--	291
226	--	290
227	--	291
228	ASA-------------------------------------	296
229	--	291
230	AGA-------------------------------------	296
231	--	291
232	--	306
233	--	291
234	--	291
235	--	291
236	--	291

237	---------------------------------------	291
238	---------------------------------------	291
239	---------------------------------------	295
240	---------------------------------------	303
241	---------------------------------------	291
242	---------------------------------------	291
243	---------------------------------------	291
244	---------------------------------------	291
245	---------------------------------------	291
246	---------------------------------------	291
247	---------------------------------------	291
248	---------------------------------------	289
249	---------------------------------------	291
250	---------------------------------------	291
251	---------------------------------------	291
252	---------------------------------------	291
253	---------------------------------------	291
254	---------------------------------------	290
255	---------------------------------------	291
256	---------------------------------------	291
257	---------------------------------------	291
258	---------------------------------------	291
259	---------------------------------------	291
260	---------------------------------------	291
261	---------------------------------------	292
262	---------------------------------------	291
263	---------------------------------------	293
264	---------------------------------------	293
265	---------------------------------------	292
266	---------------------------------------	290
267	---------------------------------------	292
268	---------------------------------------	292
269	---------------------------------------	291
270	EKPTTGGSGSTGGNQGGTGGGN-----------------	315
271	---------------------------------------	291
272	---------------------------------------	291
273	---------------------------------------	292
274	---------------------------------------	292
275	---------------------------------------	291
276	---------------------------------------	291
277	---------------------------------------	291
278	---------------------------------------	292
279	---------------------------------------	291
280	---------------------------------------	291
281	---------------------------------------	282
282	---------------------------------------	292
283	---------------------------------------	291
284	---------------------------------------	291
285	---------------------------------------	289
286	---------------------------------------	291
287	---------------------------------------	292
288	---------------------------------------	291
289	---------------------------------------	292
290	---------------------------------------	291
291	---------------------------------------	291
292	---------------------------------------	291
293	---------------------------------------	291
294	---------------------------------------	291
295	---------------------------------------	292
296	---------------------------------------	292
297	---------------------------------------	292
298	---------------------------------------	289
299	---------------------------------------	292
300	---------------------------------------	285
301	---------------------------------------	292
302	---------------------------------------	292
303	---------------------------------------	291

304	---------------------------------------	292
305	---------------------------------------	292
306	---------------------------------------	292
307	---------------------------------------	291
308	---------------------------------------	292
309	---------------------------------------	291
310	---------------------------------------	291
311	---------------------------------------	291
312	---------------------------------------	292
313	---------------------------------------	291
314	---------------------------------------	291
315	---------------------------------------	291
316	---------------------------------------	292
317	---------------------------------------	293
318	---------------------------------------	292
319	---------------------------------------	292
320	---------------------------------------	291
321	---------------------------------------	292
322	---------------------------------------	291
323	---------------------------------------	291
324	---------------------------------------	288
325	---------------------------------------	291
326	---------------------------------------	292
327	---------------------------------------	292
328	---------------------------------------	292
329	---------------------------------------	291
330	---------------------------------------	291
331	---------------------------------------	292
332	---------------------------------------	291
333	---------------------------------------	291
334	---------------------------------------	293
335	---------------------------------------	291
336	---------------------------------------	289
337	---------------------------------------	291
338	---------------------------------------	292
339	---------------------------------------	291
340	---------------------------------------	292
341	---------------------------------------	291
342	---------------------------------------	301
343	---------------------------------------	291
344	---------------------------------------	291
345	---------------------------------------	291
346	---------------------------------------	291
347	---------------------------------------	288
348	---------------------------------------	293
349	---------------------------------------	291
350	---------------------------------------	290
351	---------------------------------------	281
352	---------------------------------------	291
353	---------------------------------------	306
354	---------------------------------------	287
355	---------------------------------------	293
356	---------------------------------------	291
357	---------------------------------------	290
358	---------------------------------------	291
359	---------------------------------------	292
360	---------------------------------------	291
361	---------------------------------------	293
362	---------------------------------------	292
363	---------------------------------------	291
364	---------------------------------------	291
365	---------------------------------------	291
366	---------------------------------------	291
367	---------------------------------------	293
368	---------------------------------------	293
369	---------------------------------------	291
370	---------------------------------------	291

```
371  ---------------------------------------  291
372  ---------------------------------------  291
373  ---------------------------------------  291
374  ---------------------------------------  291
375  ---------------------------------------  291
376  ---------------------------------------  291
377  ---------------------------------------  295
378  ---------------------------------------  291
379  ---------------------------------------  291
380  ---------------------------------------  293
381  ---------------------------------------  293
382  ---------------------------------------  305
383  ---------------------------------------  293
384  ---------------------------------------  293
385  ---------------------------------------  291
386  ---------------------------------------  291
387  ---------------------------------------  306
388  ---------------------------------------  293
389  ---------------------------------------  292
390  ---------------------------------------  305
391  ---------------------------------------  293
392  ---------------------------------------  291
393  ---------------------------------------  291
394  ---------------------------------------  293
395  ---------------------------------------  293
396  ---------------------------------------  305
397  ---------------------------------------  310
398  ---------------------------------------  287
399  ---------------------------------------  293
400  ---------------------------------------  293
401  ---------------------------------------  293
402  ---------------------------------------  293
403  ---------------------------------------  289
404  ---------------------------------------  292
405  ---------------------------------------  286
406  ---------------------------------------  293
407  ---------------------------------------  290
408  ---------------------------------------  291
409  ---------------------------------------  293
410  ---------------------------------------  293
411  ---------------------------------------  293
412  ---------------------------------------  293
413  ---------------------------------------  287
414  ---------------------------------------  293
415  ---------------------------------------  293
416  ---------------------------------------  293
417  ---------------------------------------  305
418  ---------------------------------------  291
419  ---------------------------------------  300
420  ---------------------------------------  293
421  ---------------------------------------  287
422  ---------------------------------------  295
423  ---------------------------------------  305
424  ---------------------------------------  293
425  ---------------------------------------  293
426  ---------------------------------------  295
427  ---------------------------------------  291
428  ---------------------------------------  291
429  ---------------------------------------  286
430  ---------------------------------------  298
431  ---------------------------------------  293
432  ---------------------------------------  305
433  ---------------------------------------  286
434  ---------------------------------------  292
435  ---------------------------------------  292
436  ---------------------------------------  293
437  ---------------------------------------  291
```

```
438    ---------------------------------------    293
439    AKPTTGGS---GGTQGSTGGGN-----------------    312
440    ---------------------------------------    287
441    ---------------------------------------    295
442    ---------------------------------------    294
443    ---------------------------------------    291
444    ---------------------------------------    305
445    ---------------------------------------    305
446    ---------------------------------------    284
447    ---------------------------------------    285
448    ---------------------------------------    293
449    ---------------------------------------    284
450    ---------------------------------------    286
451    ---------------------------------------    295
452    ---------------------------------------    295
453    ---------------------------------------    293
454    ---------------------------------------    310
455    ---------------------------------------    300
456    ---------------------------------------    288
457    ---------------------------------------    282
458    ---------------------------------------    290
459    ---------------------------------------    300
460    ---------------------------------------    283
461    ---------------------------------------    310
462    ---------------------------------------    278
463    GECA--------------GPVPA----------------    236
464    ---------------------------------------    290
465    ---------------------------------------    285
466    ---------------------------------------    276
467    ---------------------------------------    280
468    ---------------------------------------    281
469    ---------------------------------------    294
470    ---------------------------------------    282
471    ---------------------------------------    306
472    ---------------------------------------    310
473    ---------------------------------------    306
474    ---------------------------------------    276
475    ---------------------------------------    282
476    ---------------------------------------    310
477    ---------------------------------------    305
478    ---------------------------------------    305
479    ---------------------------------------    309
480    ---------------------------------------    306
481    ---------------------------------------    278
482    ---------------------------------------    276
483    ---------------------------------------    294
484    ---------------------------------------    277
485    ---------------------------------------    295
486    ---------------------------------------    294
487    ---------------------------------------    288
488    ---------------------------------------    294
489    ---------------------------------------    294
490    ---------------------------------------    310
491    ---------------------------------------    275
492    ---------------------------------------    305
493    ---------------------------------------    279
494    ---------------------------------------    295
495    ---------------------------------------    277
496    ---------------------------------------    278
497    ---------------------------------------    306
498    ---------------------------------------    275
499    ---------------------------------------    294
500    ---------------------------------------    310
```

Compare to: the consensus

Amino acids that match the reference are marked with yellow highlighting.

Created: 4 Aug 2022

Last Modified: 4 Aug 2022

Feature	Location	Size	⇄	Type
